# Characterizing the Impact of Cyanobacterial Blooms on the Photoreactivity of Surface Waters from New York Lakes: A Combined Statewide Survey and Laboratory Investigation

**DOI:** 10.1021/acs.est.3c09448

**Published:** 2024-04-17

**Authors:** Joseph Wasswa, MaryGail Perkins, David A. Matthews, Teng Zeng

**Affiliations:** † Department of Civil and Environmental Engineering, Syracuse University, Syracuse, New York 13244, United States; ‡ Upstate Freshwater Institute, Syracuse, New York 13206, United States

**Keywords:** harmful algal blooms, DOM, photochemistry, reactive intermediates, inland waters

## Abstract

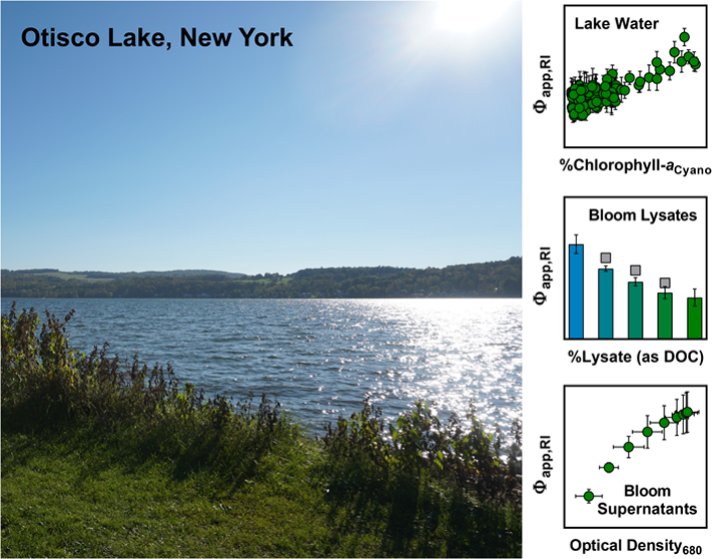

Cyanobacterial blooms introduce autochthonous dissolved organic matter (DOM) into aquatic environments, but their impact on surface water photoreactivity has not been investigated through collaborative field sampling with comparative laboratory assessments. In this work, we quantified the apparent quantum yields (Φ_app,RI_) of reactive intermediates (RIs), including excited triplet states of dissolved organic matter (^3^DOM*), singlet oxygen (^1^O_2_), and hydroxyl radicals (^•^OH), for whole water samples collected by citizen volunteers from more than 100 New York lakes. Multiple comparisons tests and orthogonal partial least-squares analysis identified the level of cyanobacterial chlorophyll *a* as a key factor in explaining the enhanced photoreactivity of whole water samples sourced from bloom-impacted lakes. Laboratory recultivation of bloom samples in bloom-free lake water demonstrated that apparent increases in Φ_app,RI_ during cyanobacterial growth were likely driven by the production of photoreactive moieties through the heterotrophic transformation of freshly produced labile bloom exudates. Cyanobacterial proliferation also altered the energy distribution of ^3^DOM* and contributed to the accelerated transformation of protriptyline, a model organic micropollutant susceptible to photosensitized reactions, under simulated sunlight conditions. Overall, our study provides insights into the relationship between the photoreactivity of surface waters and the limnological characteristics and trophic state of lakes and highlights the relevance of cyanobacterial abundance in predicting the photoreactivity of bloom-impacted surface waters.

## Abstract 2

Cyanobacterial blooms affect the photoproduction of reactive intermediates and lead to changes in the potential for photosensitized reactions in sunlit surface waters.

## Introduction

Cyanobacterial blooms not only exert profound impacts on ecosystem functioning and biogeochemical cycling across the freshwater–marine continuum but also pose a major challenge for water quality management in a changing climate.^1−3^ Cyanobacteria photosynthetically fix CO_2_ and synthesize diverse organic compounds (e.g., carbohydrates, nitrogenous substances, lipids, and organic acids) spanning a spectrum of composition and reactivity.^4−6^ Multiple factors, such as phylogenetic diversity, physiological traits, and environmental conditions, influence the amount and character of compounds synthesized by cyanobacteria.^7−9^ Once released into the water column through active exudation or passive leakage from the cells,^7^ labile compounds are either respired to CO_2_, transformed into biomass, or further processed by co-occurring heterotrophs,^10−12^ contributing to the autochthonous fraction of dissolved organic matter (DOM).^13−16^ Together, autochthonous DOM (e.g., derived from cyanobacteria and their interactions with the associated microbiome) and allochthonous DOM (e.g., of terrestrial origin) coregulate primary productivity and ultimately the stability of aquatic food webs.^17,18^ Within the photic zone of sunlit surface waters, DOM generates reactive intermediates (RIs), such as excited triplet states of DOM (^3^DOM*), singlet oxygen (^1^O_2_), and hydroxyl radicals (^•^OH), via photochemical reactions, which modulate carbon and nutrient cycling as well as contaminant transformations and pathogen inactivation.^19−21^

To date, the impact of cyanobacterial blooms on surface water photoreactivity has primarily been investigated through the analysis of operationally defined extracellular and intracellular organic matter (i.e., EOM and IOM) extracted from commercially available or field-isolated strains cultivated in standard growth media.^22−26^ Conflicting data, however, exist concerning the photoreactivity of cyanobacteria-derived DOM fractions, with some attributing the photosensitizing capacity of IOM to accessory pigments^22,27,28^ whereas others demonstrating greater formation efficiencies of RIs from EOM than IOM.^26^ Of further note, segregating EOM and IOM for evaluation does not reflect field relevant scenarios in which cyanobacteria-derived DOM mixes with the existing pool of DOM in surface waters.^29^ Cultivating cyanobacteria in growth media has also overlooked the potential significance of their interactions with heterotrophs in natural assemblages (e.g., the turnover of labile DOM).^6,30,31^ Overall, isolating the contribution of cyanobacteria-derived DOM to the photoreactivity of bloom-impacted surface waters has proven to be challenging, and assessing the photochemical effects attributable to cyanobacterial blooms may benefit from the analysis of field-collected samples.

Over the past decade, inland lakes, reservoirs, and ponds in New York have seen a steady increase in the number of bloom reports.^32^ Current reporting of bloom events largely relies on a network of volunteers participating in the Citizens Statewide Lake Assessment Program (CSLAP),^33^ which is a longstanding water quality monitoring program established to inform lake management plans and support public education and outreach in New York.^34^ Working collaboratively with CSLAP volunteers, surface water samples were collected from more than 100 lakes across New York for organic micropollutant analysis as part of a statewide occurrence survey.^35^ Considering the varying limnological conditions and trophic status of these lakes, this set of samples also presented a unique opportunity to investigate the potential impact of cyanobacterial blooms on surface water photoreactivity over a broad geographic scale. Our primary objective of this study was (i) to characterize the photoreactivity of whole water samples from CSLAP lakes via measuring the apparent quantum yields of RIs (Φ_app,RI_) and to explore the significance of water quality indicators and cyanobacterial abundance in explaining the interlake variability in Φ_app,RI_. Two additional objectives were further addressed in the context of outcomes from the statewide survey by laboratory recultivation of bloom samples sourced from a subset of CSLAP lakes (ii) to evaluate the photoreactivity of cell lysates extracted from recultivated bloom samples under hypothetical scenarios simulating the mixing of fresh lysates with allochthonous DOM or bloom-free lake water and (iii) to examine the effects of cyanobacterial proliferation on the photoreactivity of supernatants harvested from recultivated bloom samples and the photochemical transformation kinetics of model organic micropollutants commonly detected in CLSAP lakes under simulated sunlight conditions.

## Materials and Methods

Chemical sources and reagent preparation are described in the Supporting Information.

### Field Sampling

Whole water samples (*n* = 257) were collected from 111 lakes (Figure S1) by CSLAP volunteers during the 2018 and 2019 sampling seasons (i.e., June to October) as detailed in our previous work.^35^ CSLAP lakes feature a wide range of morphometry (e.g., surface area of 2 to 17,300 ha with a median of 49 ha; Table S1), watershed characteristics (e.g., watershed area of 13 to 203,300 ha with a median of 746 ha; Table S1), and water quality trends (e.g., 23.9%, 26.5%, and 49.6% classified as (mes)oligotrophic, mesotrophic, and (meso)eutrophic, respectively; Table S2). Typically, samples were taken from an open water midlake location over the deepest basin of each lake with a Kemmerer bottle submerged below the water surface and shipped on ice to the Upstate Freshwater Institute (UFI). Upon arrival at UFI, samples were analyzed in a certified laboratory for total chlorophyll *a* (Chl-*a*) and cyanobacterial chlorophyll *a* (Chl-*a*_cyano_) by a bbe Moldaenke FluoroProbe III^36^ along with a suite of water quality parameters (e.g., pH, specific conductance, nitrate–nitrite nitrogen (NO_*x*_–N), total dissolved nitrogen (TDN), total dissolved phosphorus, and N:P ratio; Table S3).^33^ Samples were then transported to Syracuse University, filtered through 0.7-μm glass fiber filters followed by 0.2-μm polyethersulfone membranes, and stored in the dark at 4 °C until analyzed for dissolved organic carbon (DOC) and optical properties (e.g., specific UV absorbance at 254 nm (SUVA_254_),^37^*E2*:*E3* (the ratio of Napierian absorption coefficients at 250 and 365 nm),^38^ spectral slope coefficient *S*_290–400_,^39^ fluorescence index (FI),^40^ freshness index (β:α),^41^ and peak M:T (the ratio of microbial humic-like to protein-like DOM fluorescence);^42^Table S4). To complement the statewide survey, bloom samples (*n* = 12) were collected from a subset of CSLAP lakes with visible surface scums during the 2021 sampling season (i.e., August to September). Qualitative microscopic analyses by UFI confirmed the dominance of *Microcystis* and *Dolichospermum* (*Anabaena*), the two most prevalent bloom-forming cyanobacterial genera, as well as other genera of cyanobacteria (e.g., *Aphanizomenon*, *Planktothrix*, and *Woronichinia*) in these bloom samples. Once transferred to Syracuse University, bloom samples were recultivated under standardized conditions, as detailed below. To obtain a uniform background matrix for recultivation, bloom-free lake water was collected from Otisco Lake, which is a mesotrophic lake that serves as the drinking water source for ∼340,000 residents in central New York.

### Laboratory Recultivation

Laboratory cultivation experiments were conducted in batch mode using an Eppendorf Innova S44i biological shaker to generate samples for photochemical characterization. Freshly collected bloom samples were first cultivated in 1-L baffled shake flasks containing Bold 3N freshwater media at 20 ± 0.5 °C with orbital shaking at 100 rpm on 12:12-h light–dark cycles illuminated by photosynthetic light-emitting diode lights at 150 μmol m^–2^ s^–1^.^4^ Over the course of cultivation, aliquots were withdrawn from the cultures and analyzed for optical density at 680 nm (OD_680_) to monitor the biomass growth.^29^ Once the cultures entered the exponential growth phase, the cells were harvested by centrifuging for 10 min at 3500 rpm using an Eppendorf 5920R refrigerated centrifuge and then rinsed with ultrapure water to remove growth media constituents (e.g., nitrate, metals, and halides that may confound photochemical measurements).^43^ Washed cells were then resuspended in a new batch of shake flasks containing unfiltered Otisco Lake water (i.e., background matrix) and recultivated under the same conditions described above to a model photoautotroph–heterotroph experimental system.^6^ Subsample aliquots were centrifuged at selected time intervals during recultivation or when the cultures reached the stationary phase to collect the supernatants (designated as “bloom supernatants”), after which cell pellets were treated with multiple freeze–thaw cycles (i.e., from −70 to 35 °C) followed by ice-bath sonication to liberate the lysates (designated as “bloom lysates”).^44^ For recultivated bloom samples, the lysates and supernatants were filtered through 0.2-μm polyethersulfone membranes and stored in the dark at 4 °C until analyzed for physicochemical and optical properties (Tables S5 and S6). For bloom lysates collected when the cultures reached the stationary phase, antioxidant capacity was measured by the 2,2′-azinobis(3-ethylbenzothiazoline-6-sulfonic acid) assay.^45^ For bloom supernatants collected when the cultures reached the stationary phase, Chl-*a* and Chl-*a*_cyano_ were quantified as described above. Otisco Lake water was also incubated, sampled, and analyzed alongside bloom samples to serve as a baseline control. No attempts were made to fractionate DOM derived from freshly collected or recultivated bloom samples via selective extraction methods (e.g., solid-phase extraction^26^) because such techniques inevitably alter DOM composition and reactivity (e.g., due to the loss of biodegradable hydrophilic fractions^46^).

### Photochemistry Experiments

Steady-state photolysis experiments were performed in duplicate using an Atlas Suntest XLS+(II) solar simulator equipped with a 1700 W xenon arc lamp and a daylight glass 300 nm UV filter. The lamp irradiance was controlled at 320 W/m^2^ between 300 and 800 nm, and the solar simulator chamber temperature was maintained at 25 ± 1 °C by an Atlas SunCool chiller. Prior to irradiation, filtered whole water samples ([DOC] = 3.2 ± 1.0 mg C/L; pH 7.4 ± 0.5) or filtered bloom lysates and supernatants (standardized to [DOC] = 3.4 ± 0.7 mg C/L; pH 7.3 ± 0.3) were spiked with a specific probe compound to measure the formation of RIs, including furfuryl alcohol (FFA) for ^1^O_2_,^47,48^ 2,4,6-trimethylphenol (TMP) as an electron transfer probe for ^3^DOM* (),^49^ and terephthalic acid for ^•^OH,^50^ respectively. *trans*,*trans*-2,4-Hexadien-1-ol (*t*,*t*-HDO; sorbic alcohol) was spiked into bloom lysates and selected supernatants either as an energy transfer probe for ^3^DOM* ()^51^ or as a quencher to quantify the contribution of high-energy ^3^DOM* capable of sensitizing *t*,*t*-HDO isomerization^52,53^ to the formation of ^3^DOM* capable of generating ^1^O_2_ and/or oxidizing TMP. TMP and *t*,*t*-HDO were also spiked into pooled whole water samples, bloom lysates, or selected supernatants at varying concentrations to determine  (i.e., the second-order reaction rate constant of TMP with ^3^DOM*) and  (i.e., the second-order reaction rate constant of *t*,*t*-HDO with ^3^DOM*), respectively. For six bloom samples dominated by *Microcystis* and *Dolichospermum* (*Anabaena*), fresh lysates were mixed with either Suwannee River natural organic matter (SRNOM; 2R101N from the International Humic Substance Society) or Otisco Lake water at different DOC ratios (i.e., 25%, 50%, and 75% of lysates as DOC) to simulate the release of cellular organic matter into surface waters with allochthonous DOM input.^29^ Samples were then irradiated in quartz test tubes (100 mm × 11 mm i.d.; held at ∼30° from the horizontal) inside the solar simulator along with controls to quantify direct photolysis and any nonphotochemical loss of probe compounds. For each experiment, bimolecular *p*-nitroanisole/pyridine actinometer solutions were irradiated with samples to monitor the incident light intensity.^54,55^, , , and  for all samples were calculated over the wavelength range of 290–550 nm as detailed in the Supporting Information.

Protriptyline (a secondary amine-containing tricyclic antidepressant previously detected in CSLAP lakes with a concentration range of 49 to 1,500 ng/L^35^) and fluridone (a systemic herbicide applied to control invasive submerged aquatic vegetation in New York^56^ and previously detected in CSLAP lakes with a concentration range of 21 to 4,300 ng/L^35^) were selected as model organic micropollutants for assessment in additional photolysis tests. Samples (i.e., four selected bloom supernatants, Otisco Lake water, or SRNOM solution; [DOC] = 3.8 ± 0.7 mg C/L; pH 7.3 ± 0.3) or deionized water were spiked with 200 ng/L of protriptyline or fluridone and irradiated in quartz test tubes along with dark controls inside the solar simulator as described above. Over the course of irradiation, subsamples were withdrawn from quartz test tubes at predetermined time intervals and analyzed by online solid-phase extraction coupled with liquid chromatography–high-resolution mass spectrometry as described in our previous work.^57^

### Data Analysis

Optical indices were extracted from absorbance and fluorescence data by using *MATLAB R2019a*. Orthogonal partial least-squares (OPLS) analysis was performed using *SIMCA 17.0* (Umetrics) to explore the explanatory power of optical indices, physicochemical parameters, and lake-watershed characteristics for the photoreactivity of CSLAP lake water samples. OPLS analysis was chosen for its versatility to simultaneously model multiple response variables with multicollinear predictor variables following the removal of systematic variation in the predictor variables that is orthogonal to the response variables.^58,59^ OPLS modeling was conducted using , , and  for whole water samples as the response variables and a collection of eight optical indices, nine physicochemical parameters, and nine lake-watershed characteristics as the predictor variables, respectively. Each predictor variable was ranked by its explanatory power for Φ_app,RI_ based on its variable importance in the projection (VIP) score,^58^ with a VIP score of >1.0 indicating the most influential variables. Multiple linear regression analysis was further performed by stepwise variable selection to identify a subset of OPLS-prioritized variables that best explained the variability in Φ_app,RI_ with minimal multicollinearity effects based on their variable inflation factors (i.e., <2).^60^ Multiple comparison tests, Spearman’s correlation analysis, and regression analysis were performed using *GraphPad Prism 8.4*.

## Results and Discussion

### Statewide Survey of Lake Water Photoreactivity

 (1.5–4.1 × 10^–2^ with a median of 2.1 × 10^–2^; Table S15),  (1.5–4.2 × 10^–2^ with a median of 2.3 × 10^–2^; Table S20), and  (1.1–3.4 × 10^–5^ with a median of 1.9 × 10^–5^; Table S11) for whole water samples from CSLAP lakes were on the same order of magnitude as those reported for surface water samples from North American temperate lakes.^61−63^ was not measured for whole water samples due to limited sample volume. On average, the ratio of  to  (0.94 ± 0.06) approached the upper limit (e.g., 0.70–0.95) predicted from the O_2_-dependent quenching of ^3^DOM* based on the analysis of DOM isolates and low molecular weight organic sensitizers.^64,65^ Furthermore,  for 16 pooled CSLAP lake water samples (7.4–8.8 × 10^8^ M^–1^ s^–1^ with a median of 8.2 × 10^8^ M^–1^ s^–1^; Table S19) fell within the ranges reported by studies examining the reactivity of ^3^DOM* with TMP for DOM isolates (e.g., 5.4–12.6 × 10^8^ M^–1^ s^–1^) and whole water samples (e.g., 7.7–20 × 10^8^ M^–1^ s^–1^).^65−68^ Φ_app,RI_ showed positive correlations with the lake water residence time and the proportion of agricultural and urban/residential land use within the lake watershed but negative correlations with the proportion of forested land use and the watershed-to-surface-area ratio (Figure S4), reflecting the joint influence of lake morphometry and catchment characteristics on surface water photoreactivity across a lake-rich landscape representative of northeastern U.S. Φ_app,RI_ also exhibited positive correlations with optical indices such as FI and *S*_290–400_ (Figure S6), which corresponded to the covariation of photoreactivity with the source and transformation of DOM.

**Figure 1:**
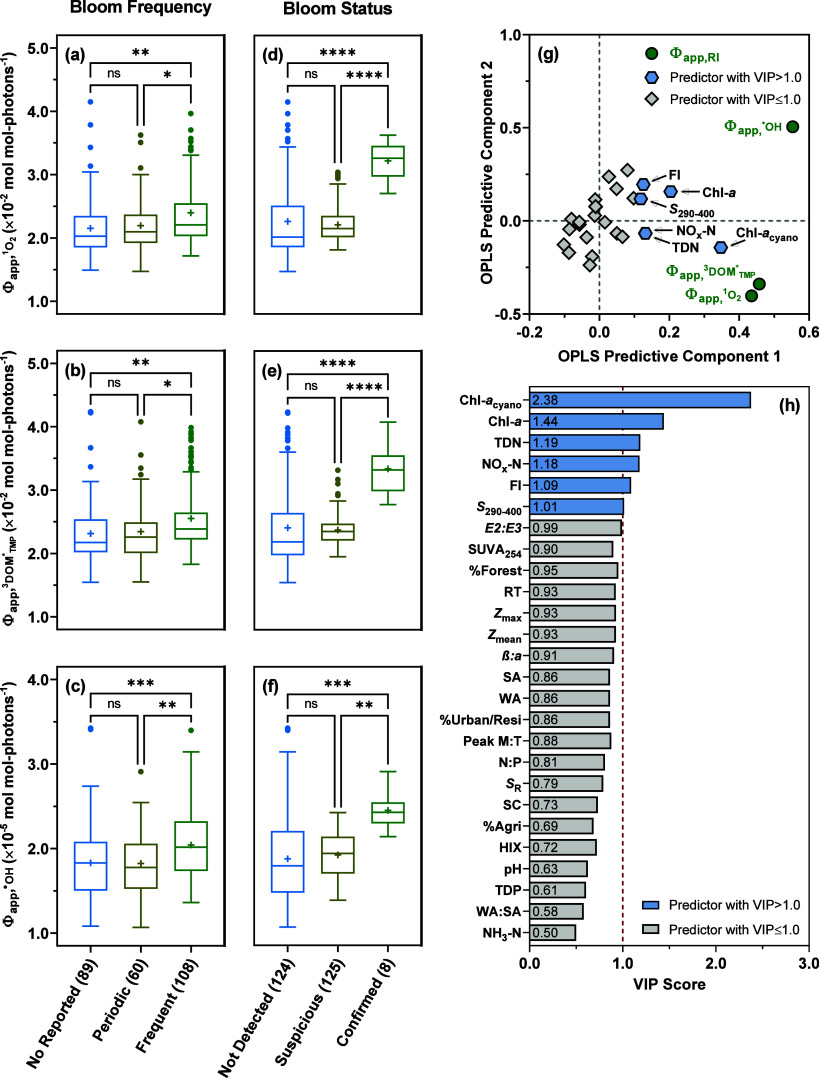
Multiple comparisons and orthogonal partial least-squares (OPLS) modeling of Φ_app,RI_ for whole water samples from CSLAP lakes: (a) Box-and-whiskers plots of  for whole water samples (*n* = 257) grouped by the bloom frequency of lakes (i.e., no reported, periodic, and frequent blooms). (b) Box-and-whiskers plots of  for whole water samples grouped by the bloom frequency of lakes. (c) Box-and-whiskers plots of  for whole water samples grouped by the bloom frequency of lakes. (d) Box-and-whiskers plots of  for whole water samples grouped by the bloom status of lakes (i.e., not detected, suspicious, and confirmed blooms) based on the concentration of cyanobacterial chlorophyll *a* (Chl-*a*_cyano_) measured at the time of sample collection. (e) Box-and-whiskers plots of  for whole water samples grouped by the bloom status of lakes. (f) Box-and-whiskers plots of  for whole water samples grouped by the bloom status of lakes. For box-and-whiskers plots, each box extends from the 25th to 75th percentiles. The whiskers extend down to the 25th percentile minus 1.5 times the interquartile range and up to the 75th percentile plus 1.5 times the interquartile range. The centerline and “+” sign mark the median and mean, respectively. Filled circles represent the outliers. The numbers in parentheses next to the *x*-axis tick labels represent the counts of samples in different categories. Significant differences between categories are denoted as “*” (*p* < 0.05), “**” (*p* < 0.01), “***” (*p* < 0.001), or “****” (*p* < 0.0001). “ns” represents no statistically significant difference. (g) Loading scatter plot of OPLS analysis for Chl-*a*_cyano_-containing whole water samples (*n* = 133), where filled green circles represent the response variables (i.e., , , and ), blue hexagons represent the six predictor variables with a VIP score of >1.0, and gray rhombi represent the remaining 20 predictor variables with a VIP score of ≤1.0. (h) Variable importance in the projection (VIP) plot of predictor variables where the red dashed line represents the VIP score threshold of 1.0. “Chl-*a*_cyano_” represents the concentration of cyanobacterial chlorophyll *a* (μg/L), “Chl-*a*” represents the concentration of chlorophyll *a* (μg/L), “TDN” represents the concentration of total dissolved nitrogen (μg/L), “NO_*x*_-N” represents the concentration of nitrate–nitrite nitrogen (μg/L), “FI” represents fluorescence index, “*S*_290–400_” represents the spectral slope coefficient from 290 to 400 nm, “*E2:E3*” represents the ratio of absorption coefficients at 250 and 365 nm, “SUVA_254_” represents the specific UV absorbance at 254 nm (L mg C^–1^ m^–1^), “%Forest” represents the percent forested land usage in the lake watershed, “RT” represents the water residence time of the lake (year), “*Z*_max_” represents the maximum depth of the lake (m), “*Z*_mean_” represents the mean depth of the lake (m), “β:α” represents freshness index, “SA” represents the lake surface area (ha), “WA” represents the lake watershed area (ha), “%Urban/Resi” represents the percent urban/residential land usage in the lake watershed, “Peak M:T” represents the ratio of microbial humic-like to protein-like DOM fluorescence, “N:P” represents the concentration ratio of total nitrogen to total phosphorus, “*S*_R_” represents the ratio of spectral slope coefficient *S*_275–295_ to *S*_350–400_, “SC” represents specific conductance (μS/cm), “%Agri” represents the percent agricultural land usage in the lake watershed, “HIX” represents humification index, “TDP” represents the concentration of total dissolved phosphorus (μg/L), “WA:SA” represents the watershed-to-surface-area ratio, and “NH_3_–N” represents the concentration of ammonia nitrogen (μg/L). Performance statistics of the OPLS model are summarized in Table S35.

Φ_app,RI_ were further grouped by the trophic state, susceptibility to blooms, or bloom frequency of CSLAP lakes to evaluate whether these management-based water quality indicators effectively reflected the photoreactivity of whole water samples. Φ_app,RI_ for samples from lakes with frequent blooms were higher than those for samples from lakes with no reported or periodic blooms (Figure 1a–c; Tukey’s multiple comparisons test *p* = 0.0008–0.0285), but Φ_app,RI_ for samples from (meso)eutrophic lakes with high susceptibility to blooms were not statistically different from those for samples from mesotrophic and (mes)oligotrophic lakes with low to moderate susceptibility to blooms (Tukey’s multiple comparisons test *p* = 0.0897–0.9619; Figures S7 and S8). Compared to the three generic indicators above, Chl-*a*_cyano_ provided a more direct assessment of bloom status as it measures the fluorescence of phycocyanin, a cyanobacterial-specific pigment.^69^ Of the CSLAP lake water samples analyzed, 52% (*n* = 133) contained 1–170 μg/L of Chl-*a*_cyano_, whereas the remaining samples (*n* = 124) did not contain quantifiable Chl-*a*_cyano_. For any sample containing Chl-*a*_cyano_, a level exceeding 25 μg/L signified a confirmed bloom, while a level below 25 μg/L was indicative of a suspicious bloom.^32^ On average, Φ_app,RI_ for samples from lakes with confirmed blooms were significantly higher than those for samples from lakes with suspicious or no blooms (Figure 1d–e; Tukey’s multiple comparisons test *p* < 0.0001–0.0012), a pattern similar to that observed upon classifying Φ_app,RI_ by the bloom frequency of lakes. Together, results from these multiple comparisons tests constituted initial evidence supporting the enhanced photoreactivity of whole water samples from CSLAP lakes experiencing frequent blooms and elevated Chl-*a*_cyano_.

To identify the main factors driving the variability in Φ_app,RI_ for whole water samples (*n* = 133) from bloom-impacted CSLAP lakes, OPLS modeling was performed using Φ_app,RI_ as the response variables and a selection of optical indices, physicochemical parameters, and lake-watershed characteristics as the predictor variables, respectively.  and  clustered together on the first predictive component axis on the OPLS loading scatter plot but were separated from  on the second predictive component axis (Figure 1g), consistent with the propositions that FFA and TMP sampled an overlapping pool of ^3^DOM*,^52,53,64^ and that the photoproduction of ^•^OH does not necessarily involve ^3^DOM*-mediated pathways.^70,71^ On the basis of VIP scores generated by OPLS modeling (Figure 1h), the six most influential predictors (i.e., those with a VIP score of >1.0) of Φ_app,RI_ followed the order of Chl-*a*_cyano_ > Chl-*a* > TDN > NO_*x*_–N > FI > *S*_290–400_. Chl-*a* has frequently been used for inferring algal abundance in large-scale lake assessment,^72,73^ whereas Chl-*a*_cyano_ represents a proxy for cyanobacterial abundance.^36^ Φ_app,RI_ for samples containing Chl-*a*_cyano_ showed stronger positive correlations with Chl-*a*_cyano_ and the proportion of Chl-*a*_cyano_ in Chl-*a* (%Chl-*a*_cyano_; an operationally defined indicator of bloom intensity^32^) than with Chl-*a* (Spearman correlation coefficient ρ = 0.423–0.669; *p* < 0.0001; Figures S9 and S10), which corroborated the prioritization of Chl-*a*_cyano_ over Chl-*a* as the top predictor of Φ_app,RI_ by OPLS modeling. Two nitrogen-related parameters, TDN and NO_*x*_-N, were also ranked as highly influential predictors, pointing to the link between Φ_app,RI_ and nitrogen loading, one of the elements implicated in the emergence of cyanobacterial blooms in lakes.^74^ Of the two remaining predictors prioritized by OPLS modeling, FI measures the relative contribution of autochthonous DOM originating from phytoplankton and bacteria versus allochthonous DOM derived from terrestrial sources,^40^ whereas *S*_290–400_ reflects the net effect of photo-biodegradation on DOM transformation.^39,75^ Multiple linear regression analyses further pinpointed Chl-*a*_cyano_ and *S*_290–400_ as the two statistically significant explanatory variables for Φ_app,RI_ (Table S36), underscoring the importance of cyanobacterial abundance and DOM turnover in assessing the impact of blooms on Φ_app,RI_ for whole water samples from CSLAP lakes. Our statewide survey of Φ_app,RI_ at best provided a snapshot of lake water photoreactivity at the time of sample collection, making it challenging to differentiate the effects of spatiotemporal shifts in watershed-scale and in-lake DOM production and processing from those attributable to cyanobacterial blooms. Complementary measurements of Φ_app,RI_ for bloom samples recultivated in a uniform background matrix (i.e., Otisco Lake water) were therefore performed to further probe the underlying mechanisms leading to the enhanced photoreactivity of bloom-impacted lake waters.

### Photoreactivity of Bloom Lysates

Given that Φ_app,RI_ for whole water samples from bloom-impacted CSLAP lakes covaried with Chl-*a*_cyano_ and, to a lesser extent, Chl-*a*, it is plausible that pigments produced by cyanobacteria served as sensitizers to enhance the photoproduction of RIs. For example, phycocyanin is a type of phycobiliprotein capable of producing reactive oxygen species (e.g., ^1^O_2_ and ^•^OH)^76−78^ via energy and electron transfer pathways^79^ and has been shown to accelerate the triplet-induced photoisomerization of microcystins when present at extremely high concentrations (e.g., >100 mg/L) in aqueous solutions.^27,28,80^ Chlorophyll *a* has also been found to catalyze the photooxidation of benzo[*a*]pyrene by ^1^O_2_ in aqueous solutions^81^ but not the photolysis of anilines and parathions in algal suspensions.^43^ Complicating matters further, phycobiliproteins, chlorophylls, and other constituents (e.g., glutathione and carotenoids) also possess antioxidant properties^82−84^ that mitigate oxidative stress to maintain redox homeostasis in cyanobacteria,^85^ and thus, they might inhibit the photoproduction of RIs. To date, the literature has reported contrasting RI formation efficiencies for cyanobacterial IOM relative to those of reference DOM isolates. For example, , , and  (i.e., the quantum yield coefficient of ^3^DOM* with TMP) for IOM extracted from a *Microcystis aeruginosa* strain from Dianchi Lake in southwestern China were 6.1–8.7 times higher than those measured for Suwannee River fulvic acid.^25^ Similarly,  for IOM extracted from bloom samples from Torrens Lake in South Australia were 2.1 times higher than that measured for Suwannee River hydrophobic acid.^24^ Somewhat in contrast to these findings, , , and  for IOM isolated from cyanobacteria from Lake Taihu in eastern China were 41 ± 7% to 67 ± 19% lower than those measured for SRNOM.^26^ Consistent with results from this latter study,  (0.8–1.3 × 10^–2^ with a median of 1.0 × 10^–2^; Table S16),  (0.9–1.3 × 10^–2^ with a median of 1.1 × 10^–2^; Table S21),  (0.6–0.9 × 10^–5^ with a median of 0.7 × 10^–5^; Table S12), and  (0.5–0.8 × 10^–2^ with a median of 0.6 × 10^–2^; Table S29) for the lysates extracted from the 12 recultivated bloom samples were 48 ± 8% to 67 ± 4% lower than those for SRNOM (Figure S11), presumably due to the lower aromaticity, higher average molecular size, and greater proteinaceous chromophore content of bloom lysates compared to SRNOM (Table S5). Φ_app,RI_ for bloom lysates also showed negative correlations with antioxidant capacity (Spearman’s ρ = −0.825 to −0.755; *p* = 0.0016–0.0062; Figure S12), supporting the notion that antioxidant constituents within the lysates likely contributed to the scavenging of ^1^O_2_ and ^•^OH and the increased probability of intramolecular charge-transfer complex formation and/or intramolecular ^3^DOM* reduction.^86^

**Figure 2:**
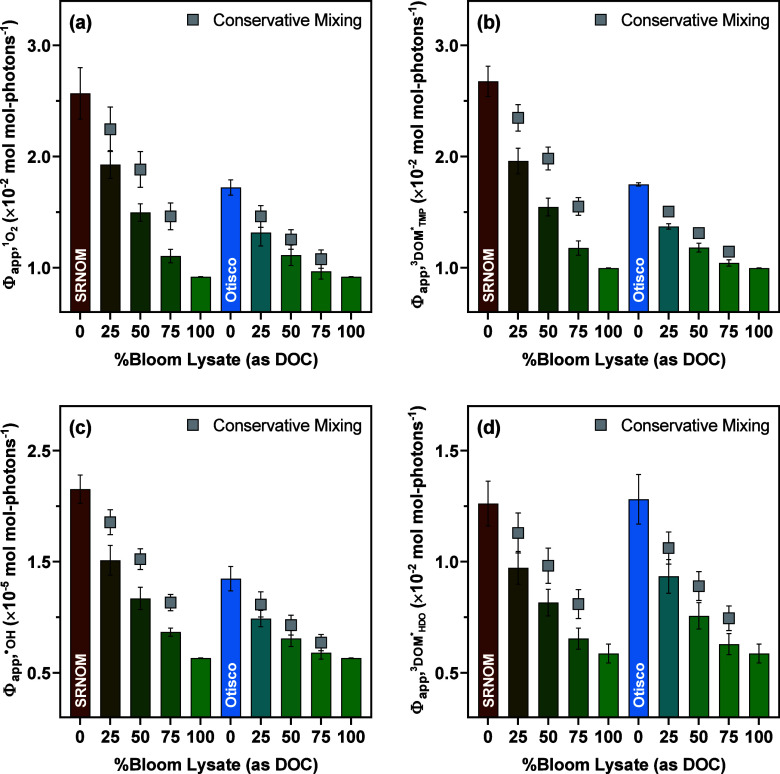
Changes in Φ_app,RI_ upon mixing the lysates extracted from six bloom samples (recultivated in Otisco Lake water until the stationary phase) with SRNOM or Otisco Lake water at different DOC ratios: (a) Comparison between  measured for the mixtures of bloom lysates with SRNOM or Otisco Lake water and  calculated assuming conservative mixing. (b) Comparison between  measured for the mixtures of bloom lysates with SRNOM or Otisco Lake water and  calculated assuming conservative mixing. (c) Comparison between  measured for the mixtures of bloom lysates with SRNOM or Otisco Lake water and  calculated assuming conservative mixing. (d) Comparison between  measured for the mixtures of bloom lysates with SRNOM or Otisco Lake water and  calculated assuming conservative mixing. Error bars represent the standard deviation of duplicate measurements for Φ_app,RI_ or the 95% confidence interval for Φ_app,RI_ calculated assuming conservative mixing; where absent, bars fall within symbols. Φ_app,RI_ profiles (Figures S14–S19) for the lysates extracted from six different bloom samples were pooled for the clarity of presentation.

Mixing the lysates extracted from six of the 12 bloom samples with SRNOM across different DOC ratios resulted in progressive decreases in Φ_app,RI_ that deviated from those derived under the assumption of conservative mixing^87^ (i.e., no interactions between the lysates and SRNOM). On average, experimentally measured Φ_app,RI_ for the mixtures of bloom lysates and SRNOM accounted for 78 ± 4% to 83 ± 4% of the values calculated by conservative mixing (Figure 2), pointing to the inhibitory effect of lysates on the photoproduction of RIs from SRNOM. Changes in  and  (i.e., the quantum yield coefficient of ^3^DOM* with *t*,*t*-HDO) upon mixing bloom lysates with SRNOM also followed trends analogous to those of  and  (Figure S13), indicating that apparent decreases in  with increasing proportions of lysates in the mixtures were not solely driven by the lower  (4.3–5.1 × 10^8^ M^–1^ s^–1^ with a median of 4.8 × 10^8^ M^–1^ s^–1^; Table S19) and  (5.2–6.3 × 10^8^ M^–1^ s^–1^ with a median of 5.8 × 10^8^ M^–1^ s^–1^; Table S28) for bloom lysates than those for SRNOM. Φ_app,RI_ for bloom lysates were 38 ± 9% to 49 ± 8% lower than those for Otisco Lake water, so the release of lysates into Otisco Lake water generated Φ_app,RI_ profiles similar to those observed with SRNOM (Figure 2). Such inhibition of RI formation qualitatively agreed with the suppression of ^1^O_2_ and ^3^DOM* production in irradiated mixtures of wastewater effluent organic matter and riverine DOM isolates^87^ as well as the reduction in photolysis rates of cyanotoxins in SRNOM solution amended with IOM extracted from a *Microcystis aeruginosa* strain.^28^ Overall, synthesizing data from current and previous work highlighted the challenge of reconciling the variability in the photoproduction efficiencies of RIs for cyanobacterial DOM fractions. Still, our mixing experiments demonstrated that bloom lysates exerted a net inhibitory effect on the photoproduction of RIs from allochthonous DOM (e.g., SRNOM), possibly due to their limited photosensitizing potential and intrinsic antioxidant capacity.

### Photoreactivity of Bloom Supernatants

Contrary to the comparatively low photoreactivity of bloom lysates,  (3.4–6.8 × 10^–2^ with a median of 5.1 × 10^–2^; Table S17),  (3.5–6.6 × 10^–2^ with a median of 4.8 × 10^–2^; Table S22),  (2.7–4.9 × 10^–5^ with a median of 3.3 × 10^–5^; Table S13), and  (3.2–4.9 × 10^–2^ with a median of 3.8 × 10^–2^; Table S30) for the supernatants harvested from the 12 recultivated bloom samples were 62 ± 28% to 206 ± 37% higher than those for SRNOM (Figure S20). Φ_app,RI_ for bloom supernatants, however, integrated the photoreactivity of native and transformed bloom exudates as well as that of Otisco Lake water. Correcting Φ_app,RI_ by subtracting the background contribution from Otisco Lake water thus provided estimates of Φ_app,RI_ attributable to bloom exudates, which were 2.6 ± 0.5 to 4.0 ± 0.4 times higher than those for the lysates extracted from the same bloom samples. Φ_app,RI_ for bloom exudates accounted for 60 ± 6% to 66 ± 4% of those for the supernatants and showed positive correlations with %Chl-*a*_cyano_ (Spearman’s ρ = 0.678–0.909; *p* = 0.0001–0.0185; Figure S21), reaffirming the association between enhanced photoreactivity and cyanobacterial abundance for whole water samples from bloom-impacted CSLAP lakes. Compared to Otisco Lake water,  (7.1–9.8 × 10^8^ M^–1^ s^–1^ with a median of 8.4 × 10^8^ M^–1^ s^–1^; Table S19) and  (8.8–9.9 × 10^8^ M^–1^ s^–1^ with a median of 9.2 × 10^8^ M^–1^ s^–1^; Table S28) for bloom supernatants were 41 ± 6% to 50 ± 15% higher and covaried with %Chl-*a*_cyano_ (Spearman’s ρ = 0.622–0.832; *p* = 0.0013–0.0347; Figure S22), suggesting that changes in cyanobacterial abundance also altered ^3^DOM* reactivity with TMP or *t*,*t*-HDO.

**Figure 3:**
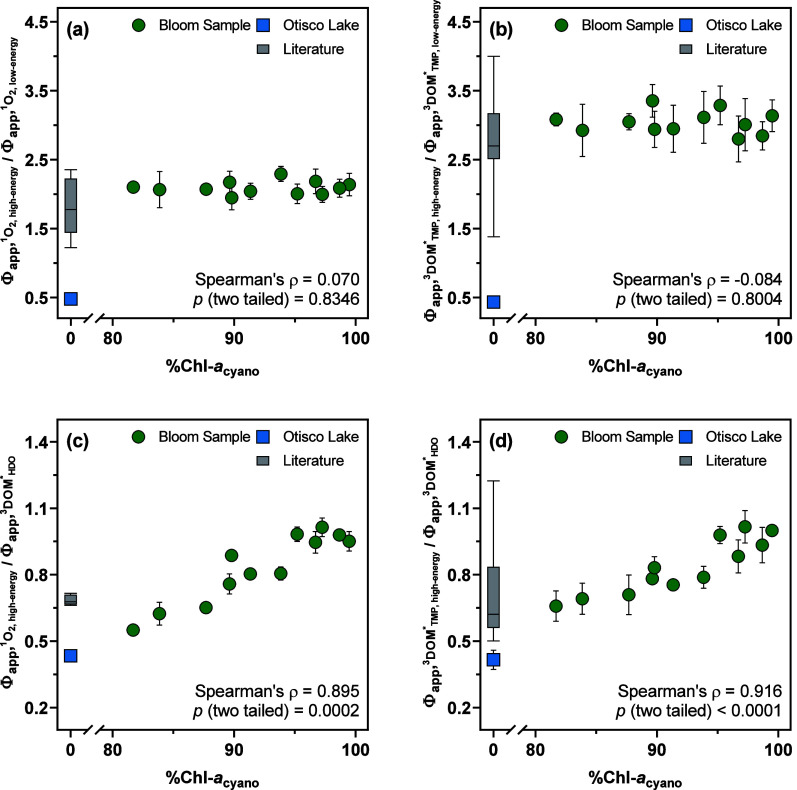
Changes in the energy distribution of ^3^DOM* with the proportion of Chl-*a*_cyano_ in Chl-*a* (%Chl-*a*_cyano_) for the supernatants harvested from bloom samples (recultivated in Otisco Lake water until the stationary phase): (a) Spearman’s correlation between the ratio of  attributable to high-energy and low-energy ^3^DOM* (minus the contribution from Otisco Lake water) and %Chl-*a*_cyano_ for bloom supernatants. Literature data included ratios measured for microbially derived DOM.^26,53,88^ (b) Spearman’s correlation between the ratio of  attributable to high-energy and low-energy ^3^DOM* (minus the contribution from Otisco Lake water) and %Chl-*a*_cyano_ for bloom supernatants. Literature data included ratios measured for microbially derived DOM.^26,53^ (c) Spearman’s correlation between the ratio of  to  (minus the contribution from Otisco Lake water) and %Chl-*a*_cyano_ for bloom supernatants. Literature data included ratios measured for eight DOM isolates from the International Humic Substance Society (IHSS).^75^ (d) Spearman’s correlation between the ratio of  to  (minus the contribution from Otisco Lake water) and %Chl-*a*_cyano_ for bloom supernatants. Literature data included ratios measured for eight IHSS DOM isolates.^75^ Error bars represent the standard deviations from duplicate measurements of Φ_app,RI_ ratios; where absent, bars fall within symbols.

Variations in  (attributable to  and ) and  (attributable to  and ) among bloom supernatants were accompanied by shifts in the relative contribution from high-energy and low-energy ^3^DOM* driven by the proliferation of cyanobacteria in Otisco Lake water. For example, with 68 ± 2% of  attributable to high-energy ^3^DOM*, the ratio of  to  (ranging from 1.9 ± 0.2 to 2.3 ± 0.1; Figure 3a) far exceeded that of Otisco Lake water and fell on the higher end of those reported for extracellular substances produced by cyanobacteria and heterotrophic bacteria (i.e., 1.4–2.4)^26,88^ as well as wastewater effluent organic matter (i.e., 1.2–2.3).^53^ With 75 ± 2% of  attributable to high-energy ^3^DOM*, the ratio of  to  (ranging from 2.8 ± 0.2 to 3.4 ± 0.2; Figure 3b) was significantly higher than that of Otisco Lake water but overlapped with those measured for Pony Lake fulvic acid (i.e., 2.7)^53^ and solid-phase extracted cyanobacterial EOM (i.e., 4.0).^26^ Only the ratio of  to  (ranging from 0.56 ± 0.13 to 1.02 ± 0.14) and the ratio of  to  (ranging from 0.66 ± 0.07 to 1.02 ± 0.07) exhibited positive correlations with %Chl-*a*_cyano_ (Figure 3c,d), indicating that bloom supernatants contained DOM enriched in moieties that were more efficient at producing high-energy ^3^DOM* capable of generating ^1^O_2_ and participating in one-electron transfer TMP oxidation than those capable of sensitizing *t*,*t*-HDO isomerization.

**Figure 4:**
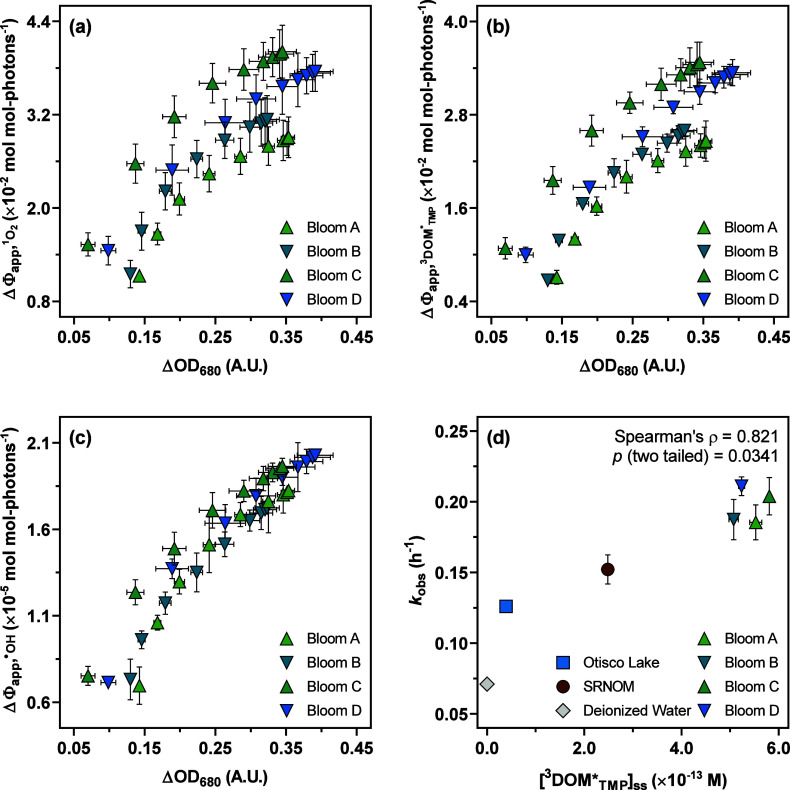
Changes in Φ_app,RI_ for the supernatants harvested from four bloom samples relative to changes in Φ_app,RI_ for Otisco Lake water per absorbance unit at 680 nm over the course of recultivation: (a) Spearman’s correlation between  and ΔOD_680_ for bloom supernatants. (b) Spearman’s correlation between  and ΔOD_680_ for bloom supernatants. (c) Spearman’s correlation between  and ΔOD_680_ for bloom supernatants. (d) Spearman’s correlation between the pseudo-first order rate constants for protriptyline photolysis (*k*_obs_) in different aqueous matrices (i.e., four bloom supernatants, Otisco Lake water, SRNOM solution, and deionized water) and the steady-state concentrations of  (i.e., ) measured in corresponding matrices under simulated sunlight conditions. Error bars represent the standard deviations from duplicate measurements of Φ_app,RI_, OD_680_, , or *k*_obs_; where absent, bars fall within symbols.

Four bloom samples with medium levels of %Chl-*a*_cyano_ were selected to further explore the evolution of Φ_app,RI_ for bloom supernatants in relation to bulk DOM character as previous work has shown that labile constituents (e.g., carbohydrates, amino acids, amino sugars)^89^ released by cyanobacteria stimulated the heterotrophic production of photoreactive moieties.^4^ Over the course of recultivation, SUVA_254_, FI, and peak M:T of bloom supernatants increased by 14 ± 1% to 34 ± 7% when the cultures reached the stationary phase, whereas *S*_290–400_ and β:α decreased by 21 ± 2% to 24 ± 5%, which was similar to the trends documented for bacterial processing of phytoplankton-derived DOM.^4,42^ Concurrently, , , and  attributable to bloom exudates increased by 163 ± 46%, 247 ± 61%, and 161 ± 39%, respectively (Figure 4a–c); in contrast, Φ_app,RI_ for Otisco Lake water underwent only minimal changes after incubation under the same conditions. Furthermore, changes in Φ_app,RI_ exhibited positive correlations with changes in SUVA_254_, FI, and peak M:T (Spearman’s ρ = 0.577–0.881; *p* < 0.0001–0.0002; Figures S23–S25) but negative correlations with changes in *S*_290–400_ and β:α (Spearman’s ρ= −0.569 to −0.833; *p* < 0.0001–0.0003; Figures S26 and S27), providing converging lines of evidence that co-occurring heterotrophs transformed the labile fraction of bloom exudates into microbial humic-like DOM characterized by an enrichment of aromatic moieties that served as precursors to ^3^DOM* as well as the photoproduction sites of ^•^OH.

Hypothetically, the increases in Φ_app,RI_ for bloom supernatants should lead to the accelerated transformation of contaminants susceptible to photosensitized reactions but exert minimal effects on the photochemical fate of contaminants primarily undergoing direct photolysis. Protriptyline and fluridone were selected as two representative organic micropollutants to test this hypothesis given their occurrence in CSLAP lakes.^35^ Indeed, the pseudo-first-order photolysis rate constants of protriptyline in bloom supernatants increased by 47 ± 14% to 67 ± 10% relative to those in Otisco Lake water and exhibited a positive correlation with  (Figure 4d), as expected from the reactivity of its secondary amine moiety with ^3^DOM* via the electron transfer mechanism.^90^ In contrast, the pseudo-first-order photolysis rate constants of fluridone in bloom supernatants were not statistically different from those in Otisco Lake water or deionized water (Figure S29), which agreed with prior work reporting comparable photolysis rates of fluridone in eutrophic lake water and distilled water.^91^ Together, our data illustrated the increased potential for photosensitized reactions in bloom-impacted lake water under simulated sunlight conditions, although the field relevance of these results warrants further investigation.

### Environmental Implications

This work showcased the feasibility of collaborating with a citizen volunteer water quality monitoring program (i.e., CSLAP) to achieve a broad-scale photochemical characterization of whole water samples from limnologically and geographically diverse lakes with varying degrees of anthropogenic influence. Coupling Φ_app,RI_ measurements with citizen science-based water quality monitoring enabled us to identify Chl-*a*_cyano_ as a key factor in explaining the enhanced photoreactivity of bloom-impacted lake waters. Laboratory recultivation of bloom samples in bloom-free lake water supported the attribution of apparent increases in Φ_app,RI_ observed during cyanobacterial growth to the production of photoreactive moieties through the heterotrophic transformation of freshly produced, labile bloom exudates rather than to the release of bloom lysates. Collectively, our results indicate that cyanobacterial proliferation enhances the photoproduction of RIs and increases the potential for photosensitized reactions in sunlit lake waters, although the generalizability of these observations should be evaluated for lakes in other regions. With recent advances in environmental data analytics, the photochemical, optical, and water chemistry data collected for CSLAP lakes may contribute to improving the predictive modeling of Φ_app,RI_ (e.g., via machine learning^92^) for bloom-impacted surface waters. Our work also highlighted areas that merit additional consideration in future research. For example, our study design did not fully replicate the complex and dynamic nature of field conditions, so additional investigations are required to gain insights from photochemical tests conducted with heterogeneous systems (e.g., algal suspensions^43^) and mechanistic models that account for chemical exchanges, microbial interactions, and hydrodynamic processes within and outside the phycosphere.^18^ From a methodological perspective, incorporating cyanobacteria-specific spectral evaluations from satellite remote sensing^62,93^ presents a promising approach for (re)constructing regional, long-term photochemical data sets at scales challenging for ground-based lake monitoring initiatives such as CSLAP. High-resolution sampling at specific lakes of interest,^94,95^ on the other hand, should provide a more integrated picture of how transient spatiotemporal heterogeneity and cyanobacterial community succession shape the photoreactivity of DOM. Overall, our work represents a step forward in understanding the implications of cyanobacterial blooms for surface water photoreactivity in the context of a changing climate.^96,97^

## Supporting Information Available

Physicochemical and optical properties of whole water and bloom samples; photochemistry experimental details and summary of photochemical parameters; correlation analysis of Φ_app,RI_ for whole water samples with lake-watershed characteristics; correlation analysis of Φ_app,RI_ with cyanobacterial abundance for whole water samples and bloom supernatants; correlation analysis of Φ_app,RI_ with antioxidant capacity for bloom lysates; changes in Φ_app,RI_ upon mixing bloom lysates with SRNOM or Otisco Lake water; changes in Φ_app,R_ with optical indices for bloom supernatants during recultivation; performance statistics of the OPLS and multiple linear regression models; photolysis kinetics of protriptyline and fluridone in bloom supernatants under simulated sunlight conditions (PDF)
