# Supplementary material for: Characterizing the Impact of Cyanobacterial Blooms on the Photoreactivity of Surface Waters from New York Lakes: A Combined Statewide Survey and Laboratory Investigation
Source: Environ Sci Technol. 2024 Apr 17;58(18):8020–31. doi: 10.1021/acs.est.3c09448 (PMC11080073; doi:10.1021/acs.est.3c09448)
Supplement: Supplementary file 1 — es3c09448_si_001.pdf [file es3c09448_si_001.pdf]

Supporting Information for

Characterizing the Impact of Cyanobacterial Blooms on the Photoreactivity of Surface Waters from New York Lakes: A Combined Statewide Survey and Laboratory Investigation

Joseph Wasswa[1], MaryGail Perkins[2], David A. Matthews[2], Teng Zeng[,1]*

[1]Department of Civil and Environmental Engineering, Syracuse University, 151 Link Hall, Syracuse, New York 13244, United States

[2]Upstate Freshwater Institute, 224 Midler Park Drive, Syracuse, New York 13206, United States

*Corresponding Author: Teng Zeng: Email: tezeng@syr.edu; Phone: +1-315-443-1099

(Total 149 pages, 9 texts, 37 tables, 30 figures)

Table of Contents

1. Chemicals, reagents, and glassware S3

2. Map of CSLAP lakes S5

3. Characteristics of CSLAP lakes S6

4. Physicochemical and optical properties of whole water and bloom samples S12

5. Analytical methods for photochemistry experiments S32

6. *p*-Nitroanisole/pyridine actinometry S33

7. Terephthalic acid (TPA) as a probe for $^{\bullet}OH$ S40

8. Furfuryl alcohol (FFA) as a probe for $^1O_2$ S48

9. 2,4,6-Trimethylphenol (TMP) as an electron transfer probe for $^3DOM^*$ S58

10. *trans,trans*-2,4-Hexadien-1-ol (*t,t*-HDO) as an energy transfer probe for $^3DOM^*$ S77

11. Correlation matrices of $\Phi_{app,RI}$ for whole water samples S88

12. Comparisons of $\Phi_{app,RI}$ for whole water samples S91

13. Performance statistics of the OPLS and MLR models of $\Phi_{app,RI}$ for whole water samples S93

14. Correlations between $\Phi_{app,RI}$ and Chl-a_{cyano}, Chl-a, or %Chl-a_{cyano} for whole water samples S95

15. Comparisons of $\Phi_{app,RI}$ for bloom lysates S97

16. Correlations between $\Phi_{app,RI}$ and AOC for bloom lysates S98

17. Changes in $\Phi_{app,RI}$ upon mixing bloom lysates with SRNOM or Otisco Lake water S99

18. Comparisons of $\Phi_{app,RI}$ for bloom supernatants S106

19. Correlations between $\Phi_{app,RI}$ and %Chl-a_{cyano} for bloom supernatants S107

20. Correlations between $k_{TMP,\ ^3DOM^*_{TMP}}$ or $k_{t,t\text{-HDO},\ ^3DOM^*_{HDO}}$ and %Chl-a_{cyano} for bloom supernatants S108

21. Changes in $\Phi_{app,RI}$ with optical indices for bloom supernatants during recultivation S109

22. Photolysis of protriptyline and fluridone in bloom supernatants under simulated sunlight conditions S114

23. Summary of literature data on the apparent quantum yields of RIs S117

References S142

1. Chemicals, reagents, and glassware

Chemicals and reagents were used as received without further purification unless otherwise noted. Methanol (MeOH; HPLC grade), acetonitrile (ACN; HPLC grade), water (HPLC grade), sodium hydroxide solution (NaOH; 0.1 N certified), sulfuric acid solution (H_2SO_4; 0.1 N certified), hydrochloric acid solution (HCl; 0.1 N certified), *o*-phosphoric acid (H_3PO_4; certified ACS grade), acetic acid (HPLC grade), trifluoroacetic acid (TFA; ≥98.5%), ammonium acetate (≥97%), and sodium sulfate (Na_2SO_4; ≥99.0%) were purchased from Fisher Scientific. Sodium chloride (NaCl; 99.5%), sodium nitrite ($NaNO_2$; ≥99.0%), sodium nitrate ($NaNO_3$; 99+%), sodium dihydrogen phosphate monohydrate ($NaH_2PO_4 \bullet H_2O$; 99+%), sodium phosphate dibasic heptahydrate ($Na_2HPO_4 \bullet 7H_2O$; 99+%), terephthalic acid (TPA; 99+%), furfuryl alcohol (FFA; 98%), 2,4,6-trimethylphenol (TMP; 99%), *p*-nitroanisole (PNA; 99+%; recrystallized[1]), and pyridine (pyr; 99+%) were purchased from ACROS Organics. 2-Hydroxyterephthalic acid (hTPA; 97%), *trans*,*trans*-2,4-hexadien-1-ol (*t,t*-HDO; sorbic alcohol; 97%), (±)-6-hydroxy-2,5,7,8-tetramethylchromane-2-carboxylic acid (Trolox; 97%), and 2,2'-azino-bis(3-ethylbenzothiazoline-6-sulfonic acid) diammonium salt (≥98%) were purchased from Sigma-Aldrich. Potassium hydrogen phthalate solution (certified carbon standard, 1000 ppm) was purchased from LabChem. pH buffer solutions (pH 4.01, 7.00, and 10.01) and conductivity calibration solution (1413 μS/cm) were purchased from Mettler Toledo. Suwannee River natural organic matter (SRNOM; 2R101N) was purchased from the International Humic Substance Society (IHSS). Bold 3N media were purchased from the Culture Collection of Algae at the University of Texas at Austin (UTEX).

Stock solutions were prepared by dissolving or diluting a gravimetrically weighted amount of solid or liquid standards into HPLC grade water. Bimolecular PNA/pyr actinometer solutions (10 μM PNA/5 mM pyr) were prepared freshly on the day of experimentation by mixing 10 mM of PNA and 12.36 M of pyridine stock solutions at a predetermined volumetric ratio.[2, 3] Working solutions and calibration standards were prepared by diluting predetermined volumes of stock solutions into ultrapure water (resistivity 18.2 MΩ•cm) produced by a Thermo Scientific Barnstead MicroPure UV/UF water purification system. Stock and working solutions were stored at 4 °C. Mobile phases for HPLC analysis were prepared using HPLC grade water and solvents.

Non-volumetric glassware was rinsed 5 times with HPLC grade methanol, followed by 5 times with ultrapure water, and combusted at 450 °C in a Thermo Scientific Lindberg/Blue M Moldatherm box furnace for a minimum of 5 h. Volumetric glassware, quartz tubes, and microsyringes were rinsed with HPLC grade methanol and ultrapure water and dried at 70 °C in a Fisherbrand Isotemp general purpose heating and drying oven.

2. Map of CSLAP lakes

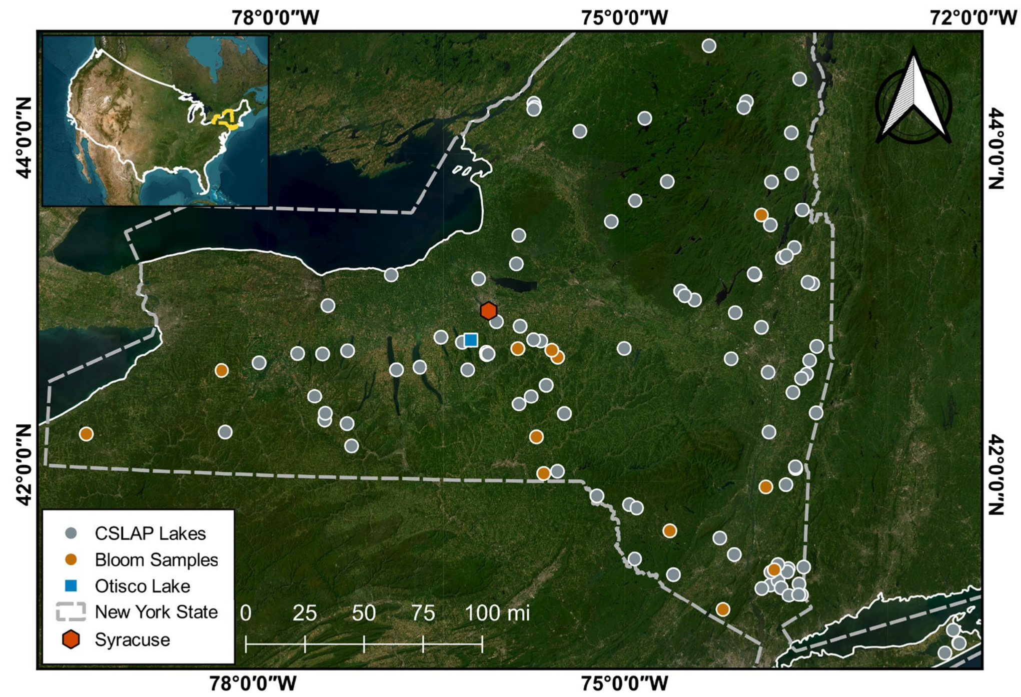

Figure S1. Map of CSLAP lakes. Whole water samples were collected from all CSLAP lakes that participated in this study, whereas bloom samples were collected from a subset of 12 lakes. Otisco Lake water was collected for use in the laboratory recultivation of bloom samples. Further details about the morphometry, watershed characteristics, and water quality status of lakes are summarized in Tables S1 and S2. Satellite Image Source: Esri, Maxar, GeoEye, Earthstar Geographics, CNES/Airbus DS, USDA, USGS, AeroGRID, IGN, and the GIS User Community.

3. Characteristics of CSLAP lakes

Table S1. Morphometry and watershed characteristics of CSLAP lakes

Lake ID	Watershed Area (ha)	Surface Area (ha)	Maximum Depth (m)	Mean Depth (m)	Residence Time (year)	Watershed Area : Surface Area	Agricultural Land Use (%)	Urban & Residential Land Use (%)	Forested Land Use (%)
2	3405	78	4.0	2.4	0.09	44	2.8	22.5	65.1
6	3448	170	4.0	2.0	0.10	20	12.8	8.8	68.8
8	2970	130	15.0	5.6	0.49	23	0.5	21.6	64.3
12	1720	407	14.5	4.4	0.69	4	9.7	3.3	52.5
13	11270	106	25.9	15.6	0.37	106	0.1	2.7	78.8
17	903	47	20.6	4.9	0.40	19	30.8	3.6	49.3
18	2113	101	13.0	5.5	0.66	21	41.0	2.0	47.7
21	1340	124	12.7	4.3	1.00	11	20.3	3.7	63.0
22	10850	1287	18.0	11.5	3.30	8	40.3	6.2	42.7
23	6606	184	14.9	5.2	0.33	36	22.6	2.4	71.6
25	145	49	5.1	3.7	0.33	3	0.0	3.4	60.0
29	120	18	11.5	4.6	2.10	7	0.0	4.0	72.0
31	500	16	4.7	2.1	0.16	31	42.9	2.9	46.5
33	46700	5434	13.3	7.8	0.69	9	22.0	7.5	55.0
34	136000	1671	44.0	17.0	0.69	81	0.1	2.5	87.8
37	50	10	10.0	3.7	1.91	5	47.4	2.6	37.4
38	1289	36	14.0	6.0	0.60	36	32.1	0.9	57.0
39	1804	109	13.0	6.2	0.93	17	18.5	0.7	67.8
40	1011	225	16.0	7.2	4.01	4	17.2	4.9	52.9
41	2230	479	14.0	7.1	3.81	5	20.2	7.1	45.4
45	410	26	6.5	2.9	0.36	16	32.1	3.8	55.1
49	13485	1359	14.0	3.9	1.10	10	38.1	6.7	36.2
50	4770	521	22.0	9.4	1.84	9	1.2	1.7	63.4
52	220	52	12.0	5.6	2.64	4	3.6	8.5	70.0
53	80	16	14.0	7.0	2.50	5	0.0	1.0	78.0
57	17500	767	13.4	10.1	0.90	23	45.9	5.9	31.2
58	224	8	6.0	3.0	0.36	28	4.8	34.0	56.6
61	52480	2745	50.6	29.1	3.10	19	48.6	4.5	35.6
66	131	47	5.1	2.3	0.45	3	28.9	3.9	53.6
68	2392	210	6.3	2.6	0.45	11	9.9	5.9	60.5
69	1305	41	18.3	8.5	0.67	32	14.1	1.4	73.9
72	1236	18	2.9	1.4	0.03	69	0.3	0.1	83.7
73	286	23	7.5	3.7	0.47	12	1.8	43.6	36.8
74	890	52	14.2	7.3	0.76	17	0.3	21.2	56.2
75	286	34	10.0	4.0	1.00	8	6.0	10.0	59.0
77	2560	112	35.4	6.8	0.75	23	23.6	15.3	34.9
78	136	16	5.5	2.3	0.59	9	0.7	2.0	88.6

Table S1. Morphometry and watershed characteristics of CSLAP lakes (continued)									
Lake ID	Watershed Area (ha)	Surface Area (ha)	Maximum Depth (m)	Mean Depth (m)	Residence Time (year)	Watershed Area : Surface Area	Agricultural Land Use (%)	Urban & Residential Land Use (%)	Forested Land Use (%)
82	1440	184	8.9	4.2	0.69	8	2.2	4.1	76.3
88	5140	1134	50.0	8.8	4.86	5	0.0	3.6	80.0
89	51	16	3.5	1.4	0.48	3	5.5	7.1	54.3
90	434	31	3.1	1.3	0.23	14	0.0	3.3	77.0
92	183100	17259	198.4	88.6	16.70	11	40.1	6.4	38.1
93	176	16	2.1	0.9	0.14	11	0.0	12.7	71.4
96	3060	267	7.5	3.7	0.69	11	20.7	5.6	56.6
99	598	114	3.0	1.4	0.35	5	0.0	0.0	84.5
100	4960	554	10.7	5.8	0.85	9	0.0	1.6	75.9
102	131	8	3.5	2.0	0.19	16	0.0	80.8	14.0
103	398	49	5.5	2.6	0.36	8	3.7	0.6	71.1
107	560	54	8.0	3.8	0.91	10	0.0	0.8	88.1
108	780	34	8.5	5.5	0.69	23	0.0	2.5	75.7
109	2703	166	6.4	2.5	0.27	16	9.2	17.1	39.4
115	10800	702	9.8	5.3	0.69	15	11.8	3.7	73.2
117	5230	329	11.6	7.2	0.69	16	62.3	7.1	12.1
120	1134	153	6.4	3.0	1.06	7	0.3	6.8	84.3
126	2269	111	11.7	5.0	0.48	20	0.9	2.8	87.7
130	90	13	5.3	2.5	0.94	7	15.3	4.9	62.3
132	111	13	4.0	2.0	0.40	9	2.0	15.0	71.0
133	746	23	6.5	3.1	0.25	32	30.5	10.2	46.0
135	4000	292	7.7	3.0	0.69	14	0.0	3.6	84.6
136	602	67	13.0	6.1	1.79	9	0.0	3.4	74.7
137	907	205	23.2	12.8	12.47	4	4.0	1.0	60.1
138	149	10	9.0	4.2	0.58	15	15.4	5.5	72.3
139	19100	3522	90.5	44.2	16.10	5	35.8	5.4	36.9
145	1440	127	22.6	10.4	0.69	11	0.4	5.4	74.2
147	270	21	5.5	2.6	0.44	13	26.9	6.6	8.6
149	400	49	18.0	4.2	1.04	8	0.0	27.1	51.7
153	106	21	13.0	6.1	2.66	5	8.7	5.3	69.3
164	444	101	9.0	3.7	1.50	4	18.0	20.0	34.9
166	63	10	9.7	4.6	1.50	6	0.0	33.6	17.0
169	997	171	12.8	5.8	1.82	6	0.0	1.4	79.5
176	10857	52	28.0	21.3	0.13	209	0.0	1.4	81.8
177	308	18	3.3	1.6	0.18	17	0.6	3.5	91.3
178	350	52	27.0	14.0	4.50	7	5.0	1.0	66.0
182	203300	17326	132.6	54.5	9.29	12	46.3	6.5	33.3
183	85	8	4.7	2.2	0.41	11	0.0	0.2	79.3

Table S1. Morphometry and watershed characteristics of CSLAP lakes (continued)									
Lake ID	Watershed Area (ha)	Surface Area (ha)	Maximum Depth (m)	Mean Depth (m)	Residence Time (year)	Watershed Area : Surface Area	Agricultural Land Use (%)	Urban & Residential Land Use (%)	Forested Land Use (%)
190	143	18	8.0	3.8	1.25	8	59.6	5.3	20.6
192	3150	26	3.0	1.4	0.02	121	3.4	1.1	90.1
194	213	29	19.0	8.9	2.44	7	41.2	3.0	46.0
195	227	13	3.1	1.5	0.23	17	2.2	4.0	88.2
199	60372	11401	60.0	14.5	6.67	5	0.8	4.9	71.7
203	85	3	3.0	2.0	0.10	28	0.5	22.0	72.5
205	645	23	11.5	6.8	0.44	28	1.4	6.4	72.3
209	42	11	3.0	1.0	0.10	4	1.0	17.0	70.0
210	98	5	6.1	2.9	0.53	20	0.5	5.7	80.7
212	255	44	8.6	4.0	1.10	6	26.1	2.8	55.0
213	555	29	7.5	3.5	0.36	19	16.2	21.2	46.7
215	132	16	1.7	0.4	0.07	8	1.3	2.4	86.8
221	1568	5	4.6	2.2	0.01	314	0.2	10.5	72.1
223	3650	132	19.0	8.9	0.81	28	37.4	14.8	34.2
225	466	47	4.3	2.0	0.35	10	0.1	10.8	71.2
229	166	29	12.0	4.9	1.40	6	1.2	20.0	58.7
230	1053	49	7.0	3.1	0.24	21	3.5	25.3	56.3
231	64	2	1.5	0.9	0.07	32	0.0	99.3	0.7
234	3937	93	9.0	4.7	10.58	42	36.6	9.0	41.0
235	235	13	8.2	4.5	0.41	18	1.2	7.7	77.2
236	69	8	10.0	4.7	0.87	9	0.0	1.3	86.1
238	5350	88	7.4	3.2	0.09	61	2.0	16.8	71.3
239	695	92	5.0	3.0	0.70	8	0.2	53.2	31.9
245	11292	80	8.8	4.0	0.05	141	35.6	5.4	53.4
246	186	6	2.1	1.2	0.10	31	5.3	33.7	57.1
247	1124	51	21.0	9.0	0.09	22	5.0	7.0	77.0
248	13	4	12.0	6.0	6.24	3	0.0	1.4	66.0
249	460	11	2.5	1.2	0.04	42	37.6	8.9	38.7
250	712	23	19.7	9.0	0.48	31	41.2	9.4	36.0
251	57	3	1.8	1.0	0.05	19	46.5	3.3	38.7
253	48173	4260	83.5	38.8	11.07	11	35.8	6.8	45.4
256	10981	894	20.1	10.2	1.74	12	42.0	4.4	41.6
257	434	12	4.1	1.8	0.17	36	3.0	16.8	53.2
258	73	6	14.0	6.0	1.00	12	4.0	25.0	52.0
259	437	11	3.0	1.0	0.10	40	22.0	1.0	73.0
260	1513	19	3.9	1.8	0.03	80	1.1	8.7	77.7
261	65	7	3.0	2.5	0.33	9	19.3	10.8	58.3
262	32116	2184	27.0	13.4	1.29	15	0.0	1.0	80.0

**Table S2.** Water quality status of CSLAP lakes			
Lake ID	Trophic State	Bloom Susceptibility	Bloom Frequency
2	Eutrophic	High susceptibility	Frequent blooms
6	Eutrophic	High susceptibility	Frequent blooms
8	Mesoligotrophic	Low susceptibility	No reported blooms
12	Mesoeutrophic	Moderate susceptibility	Frequent blooms
13	Mesoeutrophic	Low susceptibility	No reported blooms
17	Mesotrophic	Low susceptibility	No reported blooms
18	Mesoeutrophic	Low susceptibility	Frequent blooms
21	Mesotrophic	Low susceptibility	Periodic blooms
22	Mesotrophic	Low susceptibility	Frequent blooms
23	Mesoeutrophic	Moderate susceptibility	Periodic blooms
25	Mesoeutrophic	Moderate susceptibility	Frequent blooms
29	Mesoligotrophic	Low susceptibility	No reported blooms
31	Mesoeutrophic	Moderate susceptibility	Frequent blooms
33	Mesoeutrophic	High susceptibility	Frequent blooms
34	Mesoligotrophic	Low susceptibility	Periodic blooms
37	Mesotrophic	Low susceptibility	Frequent blooms
38	Mesotrophic	Low susceptibility	No reported blooms
39	Mesoligotrophic	Low susceptibility	No reported blooms
40	Mesotrophic	Moderate susceptibility	Frequent blooms
41	Mesotrophic	Low susceptibility	Frequent blooms
45	Mesoeutrophic	Moderate susceptibility	Frequent blooms
49	Mesoeutrophic	Moderate susceptibility	Frequent blooms
50	Mesoligotrophic	Low susceptibility	No reported blooms
52	Mesoligotrophic	Low susceptibility	No reported blooms
53	Mesoligotrophic	Low susceptibility	Periodic blooms
57	Mesotrophic	Low susceptibility	Periodic blooms
58	Eutrophic	Moderate susceptibility	Periodic blooms
61	Mesotrophic	Low susceptibility	Frequent blooms
66	Eutrophic	High susceptibility	Periodic blooms
68	Mesotrophic	Low susceptibility	No reported blooms
69	Mesoligotrophic	Low susceptibility	No reported blooms
72	Mesoeutrophic	Low susceptibility	Periodic blooms
73	Mesoeutrophic	Moderate susceptibility	Frequent blooms
74	Mesoeutrophic	Moderate susceptibility	Frequent blooms
75	Mesotrophic	Low susceptibility	No reported blooms
77	Mesoeutrophic	Moderate susceptibility	Frequent blooms
78	Eutrophic	High susceptibility	Frequent blooms

**Table S2.** Water quality status of CSLAP lakes (continued)			
Lake ID	Trophic State	Bloom Susceptibility	Bloom Frequency
82	Mesoligotrophic	Low susceptibility	Frequent Blooms
88	Oligotrophic	Low susceptibility	Periodic blooms
89	Eutrophic	High susceptibility	Frequent blooms
90	Eutrophic	Moderate susceptibility	Periodic blooms
92	Mesotrophic	Low susceptibility	Frequent blooms
93	Eutrophic	Moderate susceptibility	Periodic Blooms
96	Mesoeutrophic	Moderate susceptibility	Frequent blooms
99	Mesoeutrophic	Low susceptibility	No reported blooms
100	Mesoligotrophic	Low susceptibility	No reported blooms
102	Eutrophic	High susceptibility	Frequent blooms
103	Mesotrophic	Low susceptibility	No reported blooms
107	Mesoligotrophic	Low susceptibility	No reported blooms
108	Mesoligotrophic	Low susceptibility	No reported blooms
109	Eutrophic	High susceptibility	Frequent blooms
115	Eutrophic	High susceptibility	Frequent blooms
117	Mesotrophic	Low susceptibility	Periodic blooms
120	Mesoeutrophic	Low susceptibility	No reported blooms
126	Mesoligotrophic	Low susceptibility	No reported blooms
130	Mesotrophic	Low susceptibility	No reported blooms
132	Mesoeutrophic	Moderate susceptibility	No reported blooms
133	Mesoeutrophic	Moderate susceptibility	No reported blooms
135	Mesoligotrophic	Low susceptibility	No reported blooms
136	Mesotrophic	Low susceptibility	Periodic blooms
137	Oligotrophic	Low susceptibility	No reported blooms
138	Mesotrophic	Low susceptibility	Periodic Blooms
139	Oligotrophic	Low susceptibility	Periodic blooms
145	Mesoligotrophic	Low susceptibility	No reported blooms
147	Eutrophic	High susceptibility	Frequent Blooms
149	Oligotrophic	Low susceptibility	No reported blooms
153	Mesotrophic	Low susceptibility	Frequent blooms
164	Eutrophic	Moderate Susceptibility	Frequent blooms
166	Mesoligotrophic	Low susceptibility	Frequent blooms
169	Oligotrophic	Low susceptibility	No reported blooms
176	Mesoligotrophic	Low susceptibility	No reported blooms
177	Mesotrophic	Low susceptibility	No reported blooms
178	Oligotrophic	Low susceptibility	Periodic blooms
182	Mesotrophic	Low susceptibility	Frequent blooms
183	Mesoligotrophic	Low susceptibility	No reported blooms

**Table S2.** Water quality status of CSLAP lakes (continued)			
Lake ID	Trophic State	Bloom Susceptibility	Bloom Frequency
190	Eutrophic	High susceptibility	Frequent blooms
192	Eutrophic	Moderate susceptibility	No reported blooms
194	Mesotrophic	Moderate susceptibility	Periodic blooms
195	Eutrophic	Moderate susceptibility	No reported blooms
199	Oligotrophic	Low susceptibility	No reported blooms
203	Eutrophic	Moderate susceptibility	No reported blooms
205	Mesoeutrophic	Moderate susceptibility	Periodic blooms
209	Mesoeutrophic	Moderate susceptibility	Periodic blooms
210	Mesoeutrophic	Moderate susceptibility	No reported blooms
212	Mesotrophic	Low susceptibility	Frequent blooms
213	Mesoeutrophic	Moderate susceptibility	No reported blooms
215	Mesoeutrophic	Moderate susceptibility	Frequent blooms
221	Eutrophic	Moderate susceptibility	Frequent Blooms
223	Mesoeutrophic	Moderate susceptibility	Frequent blooms
225	Mesotrophic	Moderate susceptibility	Frequent blooms
229	Eutrophic	High susceptibility	Periodic blooms
230	Mesoeutrophic	Moderate susceptibility	Frequent blooms
231	Eutrophic	Moderate susceptibility	Frequent blooms
234	Mesotrophic	Low susceptibility	Periodic blooms
235	Mesotrophic	Low susceptibility	Periodic blooms
236	Mesotrophic	Low Susceptibility	Frequent blooms
238	Eutrophic	Moderate susceptibility	No reported blooms
239	Eutrophic	High susceptibility	Frequent blooms
245	Mesotrophic	Moderate susceptibility	Frequent blooms
246	Eutrophic	High susceptibility	No reported blooms
247	Mesotrophic	Low susceptibility	No reported blooms
248	Eutrophic	Moderate susceptibility	No reported blooms
249	Mesoeutrophic	Low susceptibility	No reported blooms
250	Mesotrophic	Low susceptibility	No reported blooms
251	Eutrophic	High susceptibility	No reported blooms
253	Mesoligotrophic	Low susceptibility	Frequent blooms
256	Mesotrophic	Low susceptibility	Periodic blooms
257	Eutrophic	High susceptibility	Periodic blooms
258	Mesoeutrophic	Moderate susceptibility	Periodic blooms
259	Eutrophic	High susceptibility	Periodic blooms
260	Mesoeutrophic	High susceptibility	No reported blooms
261	Mesoeutrophic	High susceptibility	Periodic blooms
262	Mesoligotrophic	Low susceptibility	Periodic blooms

4. Physicochemical and optical properties of whole water and bloom samples

For whole water samples, pH, specific conductance, and the concentrations of nitrate-nitrite nitrogen (NO_x-N), ammonia nitrogen (NH_3-N), total dissolved nitrogen (TDN), total dissolved phosphorus (TDP), total chlorophyll *a* (Chl-*a*), and cyanobacterial chlorophyll *a* (Chl-a_{cyano}) were measured at the Upstate Freshwater Institute using standard methods.[4-6] Upon transfer to Syracuse University, dissolved organic carbon (DOC) was measured by high-temperature catalytic combustion using a Teledyne-Tekmar Torch total organic carbon analyzer. UV-visible absorbance spectra were recorded in a Starna Cells 1-I-10 quartz cuvette (1-cm pathlength) from 200 to 700 nm in 1-nm increments using a Thermo Scientific Evolution 201 UV-visible spectrophotometer and corrected for blank and long-wavelength baseline.[7] Fluorescence excitation-emission matrices (EEMs) were measured in a Starna Cells 3-Q-10 quartz cuvette (1-cm pathlength) using a Horiba Scientific Aqualog spectrofluorometer.[7] Lamp, cuvette, and Raman water scans were checked prior to each sample queue. EEMs were recorded across an excitation wavelength range of 240 to 550 nm in 2-nm increments and an emission wavelength range of 247.68 to 830.02 nm in 2.33-nm increments with an integration time of 1 s and a medium CCD gain. Prior to data analysis, EEMs were corrected for instrument-specific correction factors[8] and inner filter effects,[9, 10] blank subtracted, and normalized against the Raman peak area of a Starna Cells RM-H_2O Raman water fluorescence reference standard.[11, 12] Optical indices, such as Napierian absorption coefficient (*a*) at 440 nm,[13] $SUVA_{254}$ (the specific UV absorbance at 254 nm),[14] *E2*:*E3* (the ratio of Napierian absorption coefficients at 250 and 365 nm),[15] spectral slope coefficients (e.g., $S_{275\text{-}295}$, $S_{350\text{-}400}$, $S_{290\text{-}400}$),[16, 17] spectral slope ratio (S_R; the ratio of $S_{275\text{-}295}$ to $S_{350\text{-}400}$),[18] fluorescence index (FI),[19] humification index (HIX),[20] freshness index (β:α),[21] and peak M:T (the ratio of microbial humic-like to protein-like DOM fluorescence)[22] were extracted from the absorbance and EEM fluorescence data using *MATLAB*.[7]

For bloom samples recultivated in unfiltered Otisco Lake water, optical density at 680 nm (OD_{680}) was measured using the Thermo Scientific Evolution 201 UV-visible spectrophotometer over the course of recultivation or when the cultures reached the stationary phase. Following the collection of bloom lysates and supernatants, pH, DOC, $SUVA_{254}$, *E2*:*E3*, $S_{290\text{-}400}$, FI, HIX, β:α, and peak M:T were measured as described

above. For bloom lysates collected when the cultures reached the stationary phase, antioxidant capacity (AOC) was determined by the 2,2'-azinobis-(3-ethylbenzothiazoline-6-sulfonic acid) (ABTS) assay.[23, 24] Briefly, the $ABTS^{\bullet+}$ solution was prepared by reacting 7 mM of 2,2'-azino-bis(3-ethylbenzothiazoline-6-sulfonic acid) diammonium salt with 2.45 mM of potassium persulfate and diluted with HPLC grade water and methanol (50:50 v/v) to an absorbance of 0.70±0.01 at 734 nm. Each sample (0.5 mL) was mixed with $ABTS^{\bullet+}$ solution (2.4 mL), diluted by HPLC grade water (2 mL), thoroughly vortexed, and left to stand in the dark for 6 min. AOC (using Trolox as a redox standard and expressed in mmol e^-/g C)[25] was then measured spectrophotometrically at 734 nm using the Evolution 201 UV-visible spectrophotometer. For bloom supernatants collected when the cultures reached the stationary phase, the concentrations of Chl-*a* and Chl-a_{cyano} were measured by a bbe Moldaenke FluoroProbe III.[5]

Major physicochemical and optical properties of whole water and bloom samples are summarized in **Tables S3-S6**.

**Table S3.** Physicochemical properties of whole water samples										
Sample ID [Lake ID Sampling Date]	pH	Specific Conductance (µS/cm)	NO_x-N (µg/L)	NH_3-N (µg/L)	TDN (µg/L)	TDP (µg/L)	N:P	Chl-*a* (µg/L)	Chl-a_{cyano} (µg/L)	DOC (mg C/L)
Lake 2 06/08/2019	7.1	403	115	46	366	15	10	35.1	1.0	3.87
Lake 2 08/31/2019	7.5	475	60	96	477	15	13	41.6	1.0	4.08
Lake 6 06/18/2019	6.7	203	184	59	369	32	6	70.1	1.0	4.49
Lake 6 08/28/2019	7.1	197	289	102	539	32	15	80.4	69.0	5.08
Lake 8 08/05/2019	7.1	433	61	55	247	4	31	2.4	<1.0	3.07
Lake 12 06/08/2019	6.7	160	180	42	341	6	23	3.0	<1.0	3.74
Lake 12 09/01/2019	7.2	168	117	27	402	6	26	33.1	12.0	3.74
Lake 13 07/08/2018	6.6	61	111	32	342	5	54	3.3	2.0	4.50
Lake 13 07/22/2018	6.9	62	66	21	322	7	59	3.2	1.0	3.94
Lake 13 08/06/2018	7.2	43	102	40	337	5	60	1.9	1.0	3.01
Lake 13 08/21/2018	7.6	56	36	30	216	2	47	1.5	1.0	3.79
Lake 13 09/02/2018	6.5	44	51	36	184	4	33	2.2	1.0	2.56
Lake 13 06/10/2019	6.8	86	97	27	282	4	42	5.8	<1.0	4.16
Lake 13 08/31/2019	6.9	70	64	16	227	4	31	4.9	1.0	4.17
Lake 17 07/01/2018	8.2	140	181	59	430	6	46	15.6	1.0	2.59
Lake 17 07/15/2018	7.9	139	84	55	215	8	23	12.4	1.0	2.83
Lake 17 07/28/2018	7.9	143	94	32	301	3	38	22.8	3.0	3.17
Lake 17 08/12/2018	7.5	116	205	58	417	5	76	22.7	8.0	3.19
Lake 17 08/26/2018	8.4	140	116	32	211	2	30	17.3	6.0	2.03
Lake 17 07/15/2019	7.1	174	63	111	303	5	25	3.8	<1.0	2.57
Lake 17 08/12/2019	7.5	159	58	45	305	5	26	24.0	3.0	2.88
Lake 18 08/05/2018	7.7	235	153	32	328	7	40	22.6	2.0	2.33
Lake 18 08/18/2018	7.8	235	142	25	353	6	33	32.7	4.0	2.39
Lake 18 09/09/2018	7.1	216	99	39	320	4	27	29.7	5.0	2.59
Lake 18 09/30/2018	7.1	247	166	41	323	6	23	30.4	9.0	2.67
Lake 18 06/15/2019	7.5	303	560	21	691	6	51	25.6	<1.0	2.59
Lake 18 08/12/2019	7.1	199	187	62	263	6	18	23.3	1.0	2.74
Lake 21 07/02/2019	7.4	204	74	63	272	4	47	2.0	<1.0	2.71
Lake 21 08/13/2019	7.3	165	68	72	181	4	19	2.3	<1.0	2.60
Lake 22 06/23/2019	7.6	480	130	58	407	9	32	54.8	1.0	2.71
Lake 22 06/23/2019	7.5	496	60	40	438	7	32	48.6	1.0	2.73
Lake 22 09/16/2019	7.3	402	79	26	303	9	23	64.3	2.0	2.62
Lake 22 09/16/2019	7.3	400	83	15	372	7	23	55.4	2.0	3.02
Lake 23 06/12/2019	6.7	134	171	46	422	6	29	74.4	1.0	3.04
Lake 23 09/17/2019	7.3	123	93	26	305	6	33	95.4	29.0	3.45
Lake 25 07/08/2018	8.6	339	703	222	976	13	62	37.0	7.0	3.35
Lake 25 07/29/2018	8.2	313	590	190	868	8	42	80.9	2.0	3.04
Lake 25 06/09/2019	7.5	344	746	59	1180	4	142	93.8	2.0	2.80

**Table S3.** Physicochemical properties of whole water samples (continued)										
Sample ID [Lake ID Sampling Date]	pH	Specific Conductance (µS/cm)	NO_x-N (µg/L)	NH_3-N (µg/L)	TDN (µg/L)	TDP (µg/L)	N:P	Chl-*a* (µg/L)	Chl-a_{cyano} (µg/L)	DOC (mg C/L)
Lake 29 06/02/2019	7.0	117	27	20	205	4	29	1.6	<1.0	2.11
Lake 29 08/12/2019	7.0	111	37	32	205	4	24	2.0	<1.0	2.40
Lake 31 06/15/2019	6.5	99	117	47	390	7	26	5.8	<1.0	4.86
Lake 31 08/27/2019	7.0	86	16	276	309	7	20	38.0	3.0	4.60
Lake 33 08/18/2019	7.3	196	222	51	338	17	14	126.9	12.0	2.74
Lake 33 09/01/2019	7.5	197	145	37	614	14	17	180.3	58.0	3.20
Lake 34 06/04/2019	7.0	80	39	22	244	3	35	3.4	<1.0	3.85
Lake 34 06/04/2019	6.7	71	40	22	192	3	36	2.2	<1.0	4.30
Lake 37 06/23/2019	7.2	328	677	68	881	3	94	2.9	<1.0	2.13
Lake 37 08/19/2019	7.8	223	235	56	382	3	39	34.6	5.0	2.44
Lake 38 06/17/2019	7.9	206	259	34	397	13	33	5.8	<1.0	2.87
Lake 39 07/20/2019	7.3	157	133	25	181	5	27	1.6	<1.0	2.65
Lake 40 06/27/2018	7.4	205	201	36	430	6	45	0.4	<1.0	3.07
Lake 40 07/14/2018	7.6	195	150	24	310	14	26	1.3	<1.0	2.37
Lake 40 08/01/2018	7.7	141	143	71	382	6	38	3.6	<1.0	2.38
Lake 40 08/12/2018	6.8	126	173	20	322	6	36	1.0	<1.0	2.79
Lake 40 08/29/2018	7.1	130	124	59	353	6	23	11.4	11.0	3.74
Lake 40 06/19/2019	7.7	163	56	48	308	5	26	2.3	<1.0	2.08
Lake 41 07/07/2018	7.5	266	132	60	397	7	35	1.4	<1.0	2.59
Lake 41 07/17/2018	7.7	307	278	56	382	11	22	13.8	1.0	2.57
Lake 41 07/29/2018	7.6	210	203	51	424	7	43	1.7	<1.0	2.91
Lake 41 08/19/2018	7.5	282	106	31	359	10	30	3.7	<1.0	2.56
Lake 45 08/18/2019	7.6	215	358	143	542	5	28	57.9	6.0	2.65
Lake 49 07/08/2019	8.1	358	109	39	380	7	15	4.5	<1.0	2.76
Lake 50 06/10/2019	7.3	213	16	38	183	3	27	2.4	<1.0	3.12
Lake 52 06/30/2019	7.5	321	43	35	188	3	29	1.2	<1.0	1.99
Lake 53 06/09/2019	6.3	86	88	45	268	252	44	1.6	<1.0	2.49
Lake 57 06/29/2019	7.6	290	52	20	199	5	15	223.8	1.0	3.11
Lake 57 09/08/2019	7.2	294	95	37	271	5	21	280.6	1.0	2.94
Lake 58 09/10/2019	6.1	87	548	20	687	14	44	120.9	97.0	8.33
Lake 61 07/09/2018	7.5	315	589	189	1110	6	136	4.5	<1.0	3.05
Lake 61 07/19/2018	7.6	296	689	152	973	10	125	2.9	<1.0	2.66
Lake 61 08/19/2018	7.5	283	228	83	485	6	94	3.4	<1.0	2.12
Lake 61 09/02/2018	7.7	327	508	103	917	1	157	6.3	<1.0	2.42
Lake 61 09/22/2018	8.0	296	269	40	366	5	89	0.1	<1.0	2.99
Lake 61 07/21/2019	7.5	294	1010	34	1070	4	113	2.3	<1.0	2.73
Lake 61 07/23/2019	7.6	305	752	55	1090	4	116	4.3	<1.0	2.53
Lake 66 08/16/2019	7.1	289	302	25	564	12	19	22.2	17.0	3.32

**Table S3.** Physicochemical properties of whole water samples (continued)										
Sample ID [Lake ID Sampling Date]	pH	Specific Conductance (µS/cm)	NO_x-N (µg/L)	NH_3-N (µg/L)	TDN (µg/L)	TDP (µg/L)	N:P	Chl-*a* (µg/L)	Chl-a_{cyano} (µg/L)	DOC (mg C/L)
Lake 68 09/17/2019	7.6	223	68	29	214	4	24	125.8	2.0	3.43
Lake 69 06/09/2019	6.2	49	55	44	257	3	32	1.9	<1.0	3.31
Lake 69 08/19/2019	7.4	44	47	36	204	3	25	62.9	1.0	4.71
Lake 72 07/12/2019	7.2	47	312	20	452	8	33	19.7	1.0	7.68
Lake 73 07/20/2019	7.4	545	216	78	317	10	15	48.2	3.0	3.16
Lake 73 08/24/2019	7.5	499	147	170	416	10	19	31.5	11.0	3.64
Lake 74 06/08/2019	7.4	250	122	29	352	7	20	84.0	<1.0	3.65
Lake 74 08/18/2019	7.5	203	76	87	258	7	15	120.6	1.0	3.65
Lake 75 06/02/2019	7.3	284	116	26	336	5	37	1.1	<1.0	2.91
Lake 77 06/16/2019	7.2	527	176	46	348	12	17	8.5	1.0	5.21
Lake 77 06/16/2019	7.6	493	162	35	348	14	13	6.3	<1.0	4.80
Lake 77 09/22/2019	7.1	400	156	19	351	12	16	20.9	4.0	4.23
Lake 77 09/22/2019	7.2	422	113	35	357	14	11	45.3	3.0	4.64
Lake 78 06/02/2019	6.3	44	11	53	379	13	16	7.2	<1.0	2.91
Lake 88 06/24/2019	6.7	31	72	25	239	2	62	2.7	<1.0	3.16
Lake 88 08/21/2019	7.1	27	50	54	148	2	60	21.0	1.0	2.42
Lake 89 07/10/2019	6.8	27	139	33	361	5	20	16.5	<1.0	2.91
Lake 90 07/01/2018	7.2	45	216	66	454	9	22	18.5	1.0	3.43
Lake 90 08/12/2018	7.2	49	159	46	476	11	28	25.8	5.0	4.74
Lake 90 09/11/2018	6.8	44	177	41	334	7	19	20.0	2.0	5.57
Lake 90 06/08/2019	6.3	56	137	27	258	9	16	3.4	<1.0	3.93
Lake 90 08/17/2019	6.9	53	175	105	305	9	21	2.4	<1.0	4.16
Lake 92 08/06/2018	7.3	718	219	52	424	70	7	4.8	<1.0	2.29
Lake 92 08/19/2018	7.8	647	204	75	417	10	32	5.9	<1.0	2.37
Lake 92 09/03/2018	7.5	689	322	46	531	5	39	6.3	<1.0	2.53
Lake 92 06/04/2019	7.6	753	346	28	577	4	44	2.9	<1.0	2.19
Lake 92 06/17/2019	7.9	624	353	22	608	5	63	6.3	<1.0	2.59
Lake 92 08/11/2019	7.7	621	358	26	484	5	50	2.3	<1.0	2.40
Lake 92 09/01/2019	7.5	663	280	26	432	5	47	8.5	<1.0	2.04
Lake 96 08/05/2019	7.7	195	101	29	379	10	18	235.7	14.0	2.62
Lake 99 08/10/2019	7.1	144	70	30	244	5	23	39.4	1.0	5.34
Lake 100 06/23/2019	7.5	57	70	32	203	2	43	4.2	<1.0	3.46
Lake 100 08/18/2019	7.1	46	36	26	118	2	25	34.2	1.0	3.60
Lake 102 06/16/2019	7.7	817	236	157	803	24	19	13.0	<1.0	3.08
Lake 102 09/22/2019	7.6	829	351	271	739	24	19	50.6	16.0	3.61
Lake 103 06/09/2019	7.3	101	55	116	223	6	23	3.9	<1.0	5.35
Lake 107 08/15/2019	7.4	80	101	23	217	3	41	2.0	<1.0	2.52
Lake 108 08/17/2019	6.7	135	46	25	199	4	30	8.4	<1.0	2.98

**Table S3.** Physicochemical properties of whole water samples (continued)										
Sample ID [Lake ID Sampling Date]	pH	Specific Conductance (µS/cm)	NO_x-N (µg/L)	NH_3-N (µg/L)	TDN (µg/L)	TDP (µg/L)	N:P	Chl-*a* (µg/L)	Chl-a_{cyano} (µg/L)	DOC (mg C/L)
Lake 109 07/08/2019	7.6	368	140	58	442	7	18	81.8	23.0	7.23
Lake 109 08/18/2019	8.4	254	502	77	900	7	30	97.7	89.0	6.20
Lake 115 07/08/2019	7.5	307	234	24	370	11	21	54.4	2.0	2.68
Lake 115 07/08/2019	7.6	297	298	27	492	11	26	50.1	2.0	2.83
Lake 115 09/09/2019	7.2	272	215	46	302	11	16	60.1	3.0	3.23
Lake 115 09/09/2019	7.6	263	207	101	345	11	17	29.0	3.0	3.02
Lake 117 08/22/2019	7.3	190	14	26	161	5	21	5.3	<1.0	3.13
Lake 117 09/05/2019	6.3	199	17	30	165	5	22	7.1	<1.0	2.60
Lake 120 06/10/2019	6.9	269	112	19	161	8	14	3.8	<1.0	2.92
Lake 120 08/19/2019	7.2	236	124	33	178	8	13	16.9	3.0	2.95
Lake 126 07/08/2018	8.0	65	33	51	195	5	38	5.2	1.0	2.68
Lake 126 07/21/2018	7.3	65	118	33	257	4	42	6.5	1.0	2.43
Lake 126 08/17/2019	7.3	88	26	38	123	3	22	3.2	1.0	2.44
Lake 130 08/25/2019	7.5	57	115	55	370	6	30	161.5	2.0	2.38
Lake 130 09/22/2019	6.6	53	114	22	365	6	30	3.6	<1.0	2.43
Lake 132 06/10/2019	6.8	182	86	26	262	7	16	11.4	<1.0	2.84
Lake 132 09/03/2019	7.1	148	203	19	474	7	29	148.8	10.0	2.79
Lake 133 06/04/2019	7.6	350	599	30	747	9	29	10.5	<1.0	2.77
Lake 133 08/19/2019	7.7	334	216	71	648	9	22	36.1	2.0	2.66
Lake 135 05/27/2019	6.2	98	42	13	194	3	35	2.8	<1.0	3.52
Lake 135 08/31/2019	7.3	176	60	34	201	3	30	1.9	<1.0	3.46
Lake 136 06/08/2019	6.7	149	98	31	263	6	29	41.8	1.0	2.61
Lake 136 09/20/2019	7.2	127	147	71	317	6	32	20.1	7.0	2.61
Lake 137 08/18/2019	7.5	103	20	19	182	3	25	0.7	<1.0	2.36
Lake 139 08/06/2018	6.9	282	161	52	725	3	242	0.6	<1.0	2.08
Lake 139 09/19/2018	7.1	258	91	46	455	1	130	3.6	1.0	1.90
Lake 145 06/06/2019	6.2	106	46	23	168	2	44	13.3	<1.0	4.17
Lake 145 08/15/2019	7.0	99	19	34	188	2	38	3.7	<1.0	4.19
Lake 149 06/10/2019	7.1	220	24	29	174	3	30	1.2	<1.0	3.01
Lake 149 09/21/2019	6.7	145	34	16	134	3	29	31.5	1.0	2.37
Lake 153 08/25/2019	7.0	174	69	20	164	2	19	1.5	<1.0	2.64
Lake 153 09/08/2019	7.2	106	32	26	151	2	17	3.0	<1.0	2.53
Lake 164 06/23/2019	7.9	339	18	66	548	7	22	47.1	1.0	3.38
Lake 164 08/27/2019	7.6	358	313	36	478	7	19	35.8	5.0	3.93
Lake 166 07/14/2019	7.5	409	236	25	365	4	31	0.9	<1.0	2.96
Lake 166 09/29/2019	7.2	388	168	21	369	4	33	34.5	2.0	3.00
Lake 169 07/22/2019	7.4	203	25	15	205	2	36	0.9	<1.0	2.86
Lake 169 08/18/2019	7.5	172	32	28	190	2	34	2.8	<1.0	3.17

Table S3. Physicochemical properties of whole water samples (continued)										
Sample ID [Lake ID Sampling Date]	pH	Specific Conductance (µS/cm)	NO_x-N (µg/L)	NH_3-N (µg/L)	TDN (µg/L)	TDP (µg/L)	N:P	Chl-*a* (µg/L)	Chl-a_{cyano} (µg/L)	DOC (mg C/L)
Lake 176 07/15/2018	7.5	42	124	25	314	6	66	1.6	<1.0	3.42
Lake 176 06/15/2019	7.0	54	123	58	306	2	72	2.8	<1.0	4.25
Lake 176 08/19/2019	6.7	44	62	14	223	2	57	3.6	<1.0	4.10
Lake 177 08/19/2019	7.4	141	72	25	204	5	19	4.0	<1.0	3.38
Lake 178 08/06/2019	7.2	110	141	17	247	2	52	1.0	<1.0	3.31
Lake 182 07/17/2018	8.6	492	706	194	1070	5	80	5.2	<1.0	2.30
Lake 182 07/31/2018	8.3	480	650	176	1180	9	60	5.1	<1.0	2.83
Lake 182 08/14/2018	8.0	475	595	64	861	10	62	6.9	<1.0	2.65
Lake 182 09/11/2018	7.0	506	516	59	638	3	57	12.9	1.0	2.28
Lake 182 06/18/2019	8.0	420	822	45	1220	7	81	6.6	<1.0	2.05
Lake 182 06/18/2019	7.5	342	686	35	1010	5	100	4.2	<1.0	2.39
Lake 182 07/01/2019	7.6	452	893	201	1350	5	91	17.5	1.0	2.54
Lake 182 07/08/2019	7.5	448	808	34	1070	6	53	13.3	1.0	2.31
Lake 182 08/12/2019	7.5	452	765	33	981	7	68	14.5	1.0	2.32
Lake 182 08/12/2019	7.9	355	633	28	763	5	72	6.2	<1.0	2.19
Lake 182 08/27/2019	7.9	408	902	78	1452	5	98	19.5	1.0	2.50
Lake 183 08/25/2019	6.8	25	59	37	207	3	25	4.7	<1.0	2.56
Lake 183 09/10/2019	6.8	27	82	13	223	3	27	5.8	<1.0	3.17
Lake 190 06/17/2019	7.8	216	757	67	916	16	25	73.6	49.0	4.64
Lake 190 08/19/2019	7.8	189	622	46	952	16	25	36.7	35.0	4.48
Lake 192 09/25/2019	5.9	67	10	37	335	15	13	29.7	2.0	4.25
Lake 194 06/26/2018	7.4	73	122	65	327	5	45	12.0	1.0	3.50
Lake 194 07/09/2018	7.5	71	81	29	257	6	47	14.6	1.0	3.58
Lake 194 07/23/2018	7.2	72	157	39	292	6	46	11.8	1.0	2.74
Lake 194 08/07/2018	7.3	72	134	65	370	5	63	12.1	1.0	3.12
Lake 194 08/20/2018	6.7	71	129	22	246	4	43	16.6	2.0	3.07
Lake 194 09/09/2018	6.9	72	241	53	396	2	52	14.0	2.0	3.04
Lake 194 09/22/2019	7.3	70	100	36	264	4	26	23.8	8.0	3.27
Lake 195 06/03/2019	6.1	162	18	23	199	13	8	3.5	<1.0	5.58
Lake 199 06/19/2018	7.8	140	169	14	283	2	76	0.4	<1.0	2.52
Lake 199 07/03/2018	7.4	127	129	22	216	4	34	0.5	<1.0	2.02
Lake 199 07/18/2018	7.5	101	68	17	187	2	70	0.9	<1.0	1.83
Lake 199 07/31/2018	7.3	125	14	19	152	1	58	1.0	<1.0	2.22
Lake 199 08/14/2018	7.5	124	34	20	102	1	53	0.9	<1.0	2.06
Lake 199 08/28/2018	7.3	125	44	24	211	2	46	0.8	<1.0	2.19
Lake 199 09/11/2018	6.9	119	42	32	205	3	72	1.5	<1.0	1.81
Lake 199 06/23/2019	7.5	146	24	16	152	2	26	1.2	<1.0	1.83
Lake 199 07/08/2019	7.1	149	21	22	136	2	28	0.7	<1.0	2.20

**Table S3.** Physicochemical properties of whole water samples (continued)										
Sample ID [Lake ID Sampling Date]	pH	Specific Conductance (µS/cm)	NO_x-N (µg/L)	NH_3-N (µg/L)	TDN (µg/L)	TDP (µg/L)	N:P	Chl-*a* (µg/L)	Chl-a_{cyano} (µg/L)	DOC (mg C/L)
Lake 199 07/30/2019	6.7	154	14	24	157	2	28	1.1	<1.0	2.48
Lake 199 08/18/2019	7.3	134	14	25	119	2	21	1.1	<1.0	1.95
Lake 199 09/15/2019	7.3	132	12	38	105	2	23	1.8	<1.0	2.22
Lake 203 06/16/2019	7.6	686	183	116	319	14	14	1.9	<1.0	3.38
Lake 203 09/22/2019	7.0	646	178	93	442	14	15	4.0	<1.0	3.56
Lake 205 06/08/2019	7.0	209	12	30	316	7	26	44.2	1.0	3.58
Lake 205 08/06/2019	7.4	195	181	24	379	7	22	29.8	2.0	3.63
Lake 209 08/18/2019	7.6	121	118	40	217	5	18	5.6	<1.0	2.73
Lake 210 06/02/2019	6.8	148	959	91	1130	5	71	3.9	<1.0	4.10
Lake 210 08/18/2019	7.2	171	635	163	923	5	61	2.5	<1.0	4.84
Lake 212 06/16/2018	8.1	199	152	34	502	6	49	3.2	1.0	2.15
Lake 212 07/14/2018	7.9	198	101	25	350	8	32	2.9	1.0	3.73
Lake 212 07/28/2018	7.9	209	226	42	501	5	47	3.0	1.0	3.56
Lake 212 08/12/2018	7.2	203	77	64	421	5	39	5.0	1.0	3.77
Lake 212 06/17/2019	7.0	222	127	28	438	6	31	14.7	<1.0	4.12
Lake 213 08/18/2019	7.4	366	160	126	484	6	28	54.6	9.0	2.82
Lake 215 06/09/2019	7.1	178	45	27	236	5	25	1.6	<1.0	3.94
Lake 215 09/15/2019	7.0	185	35	31	267	5	28	3.3	<1.0	3.91
Lake 223 08/19/2019	7.8	295	155	28	355	7	18	164.9	4.0	4.33
Lake 225 07/28/2019	7.3	369	107	30	275	6	28	39.2	1.0	3.76
Lake 229 06/29/2019	7.9	202	98	44	303	7	26	1.4	<1.0	2.53
Lake 230 06/08/2019	7.1	443	81	13	285	11	16	38.0	4.0	3.19
Lake 231 06/04/2019	7.7	1424	449	29	627	29	12	1.8	<1.0	3.99
Lake 234 06/20/2018	7.6	526	288	48	683	5	66	6.2	<1.0	2.48
Lake 234 07/15/2018	8.2	534	275	51	447	4	47	3.1	<1.0	2.42
Lake 234 06/09/2019	7.1	559	660	40	970	5	71	4.0	<1.0	2.80
Lake 234 08/12/2019	7.7	514	185	71	381	5	27	10.9	1.0	2.25
Lake 235 07/14/2019	6.8	178	47	48	233	5	26	4.6	<1.0	4.50
Lake 236 07/14/2019	6.8	158	49	30	208	4	18	2.1	<1.0	2.70
Lake 236 09/15/2019	6.5	149	24	28	202	4	23	19.0	6.0	2.96
Lake 238 08/12/2019	6.2	158	103	21	401	12	32	50.3	6.0	4.52
Lake 239 06/09/2019	7.4	594	218	35	366	25	8	30.3	6.0	3.20
Lake 239 08/18/2019	7.5	527	29	197	607	25	11	31.7	8.0	3.14
Lake 245 09/22/2019	7.4	481	274	31	319	7	24	1.2	<1.0	2.39
Lake 246 08/05/2019	7.0	415	158	22	715	30	16	42.4	8.0	5.45
Lake 247 06/08/2019	6.4	135	46	34	182	4	22	122.6	1.0	3.56
Lake 247 09/20/2019	6.6	125	67	32	217	4	26	178.3	3.0	3.45
Lake 248 08/18/2019	7.2	90	290	328	655	225	4	62.2	2.0	5.27

**Table S3.** Physicochemical properties of whole water samples (continued)										
Sample ID [Lake ID Sampling Date]	pH	Specific Conductance (µS/cm)	NO_x-N (µg/L)	NH_3-N (µg/L)	TDN (µg/L)	TDP (µg/L)	N:P	Chl-*a* (µg/L)	Chl-a_{cyano} (µg/L)	DOC (mg C/L)
Lake 249 07/08/2019	7.8	341	260	64	456	6	20	2.6	<1.0	3.00
Lake 249 09/01/2019	7.9	371	83	71	375	6	16	23.5	1.0	3.46
Lake 250 07/08/2019	7.9	426	385	23	457	6	37	3.7	<1.0	2.71
Lake 250 08/18/2019	7.7	360	227	37	331	6	27	4.2	<1.0	2.99
Lake 251 08/27/2019	7.4	202	2410	44	3520	92	31	63.0	5.0	5.04
Lake 251 09/17/2019	6.9	204	981	42	1810	92	16	45.0	12.0	5.52
Lake 253 07/02/2019	7.6	434	232	34	423	3	75	1.4	<1.0	2.29
Lake 253 08/25/2019	7.8	380	199	17	251	3	45	14.1	1.0	2.19
Lake 253 09/15/2019	7.6	365	208	34	452	3	68	53.9	2.0	2.28
Lake 256 07/05/2018	7.7	359	223	33	663	4	74	5.8	4.0	2.75
Lake 256 07/16/2018	7.8	362	325	64	740	6	49	5.6	3.0	2.07
Lake 256 07/31/2018	8.5	412	361	40	674	5	38	4.3	1.0	2.43
Lake 256 08/15/2018	8.0	351	222	58	362	7	22	5.1	2.0	2.27
Lake 256 08/29/2018	7.2	365	326	49	492	8	33	4.8	2.0	3.05
Lake 256 09/14/2018	7.8	318	163	57	501	3	29	4.0	1.0	2.46
Lake 256 09/28/2018	7.7	250	123	35	320	5	31	5.9	1.0	2.39
Lake 256 06/18/2019	7.6	361	652	50	910	4	56	4.8	<1.0	2.74
Lake 256 06/18/2019	7.8	363	633	57	908	4	56	2.4	<1.0	2.82
Lake 256 08/13/2019	7.7	361	641	25	757	4	43	4.4	1.0	2.92
Lake 257 08/24/2019	7.3	499	677	47	912	16	26	58.6	20.0	8.51
Lake 258 06/16/2019	6.7	263	11	80	492	9	27	19.8	3.0	3.39
Lake 258 08/11/2019	6.4	270	105	35	461	9	22	17.9	5.0	3.87
Lake 259 09/01/2019	6.7	93	530	72	1000	25	18	193.5	170.0	5.58
Lake 260 06/01/2019	7.4	271	27	19	269	13	10	3.5	<1.0	3.66
Lake 260 08/12/2019	7.5	363	173	44	401	13	14	53.5	4.0	3.82
Lake 261 06/17/2019	7.1	63	171	21	227	10	7	30.6	3.0	3.67
Lake 261 08/12/2019	7.3	79	227	28	382	10	12	33.1	9.0	3.49
Lake 262 06/17/2019	6.4	36	80	35	341	3	87	2.3	<1.0	6.44
Lake 262 09/21/2019	7.2	35	50	31	171	3	55	3.1	<1.0	5.38

NO_x-N = the concentration of nitrate-nitrite nitrogen; NH_3-N = the concentration of ammonia nitrogen; TDN = the concentration of total dissolved nitrogen; TDP = the concentration of total dissolved phosphorus; N:P = the concentration ratio of total nitrogen to total phosphorus; Chl-*a* = the concentration of total chlorophyll *a*; Chl-a_{cyano} = the concentration of cyanobacterial chlorophyll *a*; DOC = the concentration of dissolved organic carbon.

**Table S4.** Optical properties of whole water samples											
Sample ID [Lake ID Sampling Date]	a_{440} (m^{-1})	*E2:E3*	$S_{275–295}$ (μm^{-1})	$S_{350–400}$ (μm^{-1})	$S_{290–400}$ (μm^{-1})	S_R	$SUVA_{254}$ (L mg C^{-1}•m^{-1})	FI	HIX	$\beta:\alpha$	Peak M:T
Lake 2 06/08/2019	1.14	5.97	17.3	18.4	17.5	0.94	2.89	1.58	0.92	0.57	2.88
Lake 2 08/31/2019	0.83	7.88	21.1	20.2	19.6	1.05	3.01	1.59	0.91	0.64	3.44
Lake 6 06/18/2019	1.66	5.69	17.1	17.3	16.7	0.99	3.12	1.50	0.91	0.54	2.93
Lake 6 08/28/2019	1.42	6.98	19.5	18.5	18.3	1.06	3.21	1.63	0.91	0.59	3.15
Lake 8 08/05/2019	0.65	7.26	21.4	17.9	18.4	1.19	2.39	1.55	0.86	0.62	2.19
Lake 12 06/08/2019	0.83	6.93	19.9	19.6	18.8	1.02	2.92	1.53	0.91	0.57	2.86
Lake 12 09/01/2019	0.89	7.83	22.3	17.8	18.6	1.26	2.80	1.64	0.90	0.63	2.70
Lake 13 07/08/2018	2.89	4.91	17.4	14.3	14.5	1.22	3.19	1.48	0.82	0.52	0.84
Lake 13 07/22/2018	0.76	6.92	19.9	20.5	19.1	0.97	2.92	1.46	0.87	0.52	2.64
Lake 13 08/06/2018	0.32	9.47	18.5	24.6	22.1	0.75	2.37	1.62	0.93	0.51	5.86
Lake 13 08/21/2018	0.89	6.78	19.7	19.4	18.3	1.02	2.83	1.52	0.91	0.53	3.41
Lake 13 09/02/2018	0.08	10.90	26.8	27.6	24.9	0.97	1.97	1.74	0.86	0.71	2.20
Lake 13 06/10/2019	1.50	5.41	16.1	17.9	16.7	0.90	3.04	1.43	0.92	0.44	3.73
Lake 13 08/31/2019	1.19	5.95	17.6	18.3	17.4	0.96	2.92	1.51	0.92	0.47	4.29
Lake 17 07/01/2018	0.29	8.03	22.5	18.8	19.2	1.20	1.78	1.70	0.86	0.74	2.82
Lake 17 07/15/2018	0.84	7.06	22.4	15.9	17.1	1.41	2.45	1.69	0.87	0.64	2.29
Lake 17 07/28/2018	0.92	6.86	22.2	15.3	16.6	1.45	2.49	1.63	0.85	0.61	2.31
Lake 17 08/12/2018	0.18	9.18	24.3	24.5	22.5	0.99	2.32	1.62	0.87	0.65	2.41
Lake 17 08/26/2018	0.01	10.92	29.1	28.4	25.1	1.02	1.26	1.70	0.78	0.71	1.53
Lake 17 07/15/2019	0.44	8.33	23.1	19.6	19.8	1.18	2.38	1.65	0.85	0.66	1.85
Lake 17 08/12/2019	0.56	7.13	22.0	16.3	17.4	1.36	2.00	1.63	0.84	0.60	1.73
Lake 18 08/05/2018	0.17	10.05	25.0	20.5	21.1	1.22	1.71	1.69	0.86	0.73	2.41
Lake 18 08/18/2018	0.21	9.10	23.9	21.3	20.9	1.12	1.77	1.65	0.87	0.71	2.44
Lake 18 09/09/2018	0.55	7.05	21.8	16.0	17.2	1.36	1.99	1.76	0.88	0.70	2.86
Lake 18 09/30/2018	0.50	6.52	21.3	19.4	18.2	1.10	2.07	1.76	0.97	0.70	5.38
Lake 18 06/15/2019	0.23	7.55	21.4	21.0	20.1	1.02	1.89	1.78	0.85	0.68	1.69
Lake 18 08/12/2019	0.40	8.49	23.8	16.8	18.7	1.42	1.83	1.72	0.84	0.72	1.90
Lake 21 07/02/2019	0.32	8.56	23.0	18.6	19.6	1.24	1.84	1.62	0.88	0.64	2.14
Lake 21 08/13/2019	0.29	9.11	24.0	19.3	20.2	1.24	1.84	1.69	0.86	0.66	2.14
Lake 22 06/23/2019	0.38	10.14	24.9	20.1	21.2	1.24	2.40	1.56	0.85	0.72	1.91
Lake 22 06/23/2019	0.45	8.15	22.7	19.6	19.7	1.16	2.34	1.74	0.85	0.71	1.79
Lake 22 09/16/2019	0.41	10.00	25.0	19.2	20.5	1.30	2.22	1.67	0.85	0.69	1.90
Lake 22 09/16/2019	0.48	9.51	24.9	18.0	19.7	1.38	2.26	1.55	0.88	0.67	1.96
Lake 23 06/12/2019	0.89	6.05	17.8	16.8	16.8	1.06	2.49	1.54	0.89	0.56	2.33
Lake 23 09/17/2019	0.83	7.56	20.3	18.4	18.7	1.11	2.70	1.60	0.89	0.62	2.86
Lake 25 07/08/2018	0.59	8.92	23.2	19.5	20.0	1.19	2.61	1.72	0.76	0.71	0.98
Lake 25 07/29/2018	0.23	9.91	23.3	23.7	22.5	0.98	2.39	1.76	0.90	0.69	3.12
Lake 25 06/09/2019	0.26	10.05	22.7	22.7	22.2	1.00	2.31	1.73	0.89	0.67	2.79

**Table S4.** Optical properties of whole water samples (continued)											
Sample ID [Lake ID Sampling Date]	a_{440} (m^{-1})	*E2:E3*	$S_{275–295}$ (μm^{-1})	$S_{350–400}$ (μm^{-1})	$S_{290–400}$ (μm^{-1})	S_R	$SUVA_{254}$ (L mg C^{-1}•m^{-1})	FI	HIX	$\beta:\alpha$	Peak M:T
Lake 29 06/02/2019	0.23	7.62	23.6	20.3	19.6	1.17	1.61	1.56	0.86	0.60	1.57
Lake 29 08/12/2019	0.37	7.82	23.9	17.0	18.2	1.40	1.76	1.59	0.83	0.63	1.90
Lake 31 06/15/2019	1.61	5.79	16.9	17.9	17.1	0.94	3.18	1.52	0.92	0.50	3.64
Lake 31 08/27/2019	1.30	6.77	19.1	19.3	18.4	0.99	3.21	1.53	0.93	0.53	4.07
Lake 33 08/18/2019	0.43	8.45	22.9	18.7	19.4	1.22	2.14	1.70	0.87	0.69	2.20
Lake 33 09/01/2019	0.70	7.46	21.7	17.2	18.2	1.26	2.37	1.75	0.89	0.68	2.22
Lake 34 06/04/2019	1.24	5.73	16.3	18.8	17.4	0.87	2.97	1.52	0.92	0.46	3.52
Lake 34 06/04/2019	1.68	5.34	16.0	16.8	16.1	0.96	2.97	1.45	0.95	0.44	4.64
Lake 37 06/23/2019	0.09	9.44	24.4	25.3	23.2	0.97	1.84	1.62	0.85	0.67	1.63
Lake 37 08/19/2019	0.49	8.45	25.3	16.4	18.6	1.54	2.03	1.85	0.83	0.64	1.45
Lake 38 06/17/2019	0.33	7.37	20.9	19.7	19.2	1.07	1.86	1.65	0.87	0.62	1.96
Lake 39 07/20/2019	0.17	9.60	24.1	22.3	21.7	1.08	1.82	1.64	0.88	0.65	2.56
Lake 40 06/27/2018	0.66	6.74	19.0	20.9	19.1	0.91	2.63	1.47	0.86	0.48	1.62
Lake 40 07/14/2018	0.01	11.83	26.8	33.0	27.7	0.81	1.75	1.67	0.87	0.70	2.45
Lake 40 08/01/2018	0.15	8.87	25.0	21.6	20.8	1.16	1.76	1.66	0.89	0.71	2.54
Lake 40 08/12/2018	0.07	11.16	26.5	25.2	23.8	1.05	1.68	1.70	0.87	0.71	2.25
Lake 40 08/29/2018	0.79	6.68	19.5	19.8	18.6	0.99	2.82	1.50	0.91	0.54	3.75
Lake 40 06/19/2019	0.84	10.45	26.2	31.6	26.3	0.83	1.73	1.55	0.85	0.64	1.69
Lake 41 07/07/2018	0.20	10.31	26.1	21.9	21.9	1.19	2.00	1.65	0.83	0.69	2.21
Lake 41 07/17/2018	0.01	11.99	27.0	29.2	25.9	0.92	1.98	1.63	0.83	0.71	2.00
Lake 41 07/29/2018	0.08	11.48	26.9	25.4	24.0	1.06	1.94	1.70	0.87	0.72	2.41
Lake 41 08/19/2018	0.06	11.69	26.4	26.5	24.6	1.00	1.90	1.66	0.86	0.70	2.47
Lake 45 08/18/2019	0.45	7.76	21.6	20.4	19.9	1.05	2.27	1.78	0.89	0.60	2.33
Lake 49 07/08/2019	0.24	9.47	24.0	21.0	21.1	1.14	2.01	1.67	0.85	0.65	1.79
Lake 50 06/10/2019	0.51	7.58	20.8	21.0	20.0	0.99	2.59	1.56	0.89	0.54	2.65
Lake 52 06/30/2019	0.00	8.92	24.9	27.6	23.7	0.90	1.50	1.59	0.77	0.71	1.11
Lake 53 06/09/2019	0.52	7.02	21.7	16.5	17.4	1.31	1.90	1.44	0.85	0.55	1.51
Lake 57 06/29/2019	0.21	8.19	22.1	23.3	21.6	0.95	2.03	1.60	0.88	0.66	2.28
Lake 57 09/08/2019	0.30	9.61	24.0	21.0	21.3	1.14	2.10	1.74	0.87	0.69	2.20
Lake 58 09/10/2019	4.42	4.78	15.0	17.2	15.7	0.87	3.85	1.49	0.92	0.44	3.28
Lake 61 07/09/2018	0.34	8.66	23.1	18.9	19.7	1.22	1.93	1.67	0.88	0.68	2.30
Lake 61 07/19/2018	1.01	5.73	20.2	10.6	13.5	1.91	2.07	1.73	0.85	0.74	1.54
Lake 61 08/19/2018	0.29	8.27	23.2	19.6	19.7	1.19	1.88	1.62	0.87	0.69	2.52
Lake 61 09/02/2018	0.01	11.12	24.2	29.2	25.8	0.83	1.81	1.65	0.88	0.74	2.43
Lake 61 09/22/2018	0.50	7.89	23.1	19.2	19.2	1.20	2.36	1.69	0.87	0.67	2.43
Lake 61 07/21/2019	0.14	10.31	22.8	23.5	23.0	0.97	1.95	1.63	0.89	0.65	2.46
Lake 61 07/23/2019	0.19	10.32	23.6	22.0	22.3	1.07	1.94	1.62	0.87	0.68	2.30
Lake 66 08/16/2019	0.68	9.13	24.0	19.5	20.1	1.24	2.76	1.70	0.86	0.67	2.10

**Table S4.** Optical properties of whole water samples (continued)											
Sample ID [Lake ID Sampling Date]	a_{440} (m^{-1})	*E2:E3*	$S_{275–295}$ (μm^{-1})	$S_{350–400}$ (μm^{-1})	$S_{290–400}$ (μm^{-1})	S_R	$SUVA_{254}$ (L mg C^{-1}•m^{-1})	FI	HIX	$\beta:\alpha$	Peak M:T
Lake 68 09/17/2019	0.48	9.28	23.4	21.6	21.1	1.08	2.70	1.53	0.87	0.61	2.28
Lake 69 06/09/2019	0.83	6.09	17.3	19.2	17.9	0.90	2.66	1.46	0.93	0.49	3.50
Lake 69 08/19/2019	1.54	6.13	17.9	18.1	17.4	0.99	3.15	1.49	0.92	0.49	3.71
Lake 72 07/12/2019	3.26	5.47	16.2	18.1	16.7	0.90	3.75	1.50	0.95	0.46	5.81
Lake 73 07/20/2019	0.61	8.05	22.2	19.6	19.6	1.13	2.69	1.58	0.89	0.64	2.45
Lake 73 08/24/2019	0.69	8.11	22.9	18.4	19.1	1.24	2.61	1.69	0.87	0.68	2.37
Lake 74 06/08/2019	0.64	7.07	20.6	19.6	19.0	1.05	2.70	1.54	0.88	0.63	2.10
Lake 74 08/18/2019	0.52	8.73	23.8	19.6	20.0	1.22	2.51	1.66	0.86	0.65	2.30
Lake 75 06/02/2019	0.19	8.47	24.4	21.3	20.6	1.14	1.74	1.49	0.82	0.68	1.36
Lake 77 06/16/2019	1.32	6.62	18.2	19.3	18.5	0.95	3.27	1.59	0.91	0.56	2.87
Lake 77 06/16/2019	2.44	5.54	16.7	15.9	15.9	1.05	3.37	1.57	0.91	0.55	2.78
Lake 77 09/22/2019	0.92	7.97	21.1	20.1	19.7	1.05	3.10	1.59	0.90	0.61	2.86
Lake 77 09/22/2019	0.71	8.34	21.0	21.7	20.7	0.96	3.09	1.61	0.91	0.61	3.13
Lake 78 06/02/2019	0.72	6.04	19.8	16.2	16.6	1.23	2.13	1.51	0.86	0.58	1.65
Lake 88 06/24/2019	0.27	7.57	21.0	22.9	20.6	0.92	2.28	1.48	0.90	0.50	2.98
Lake 88 08/21/2019	0.37	8.38	23.2	19.9	19.8	1.17	2.09	1.51	0.86	0.53	2.43
Lake 89 07/10/2019	0.50	8.24	23.7	20.0	19.7	1.19	2.47	1.57	0.82	0.65	1.53
Lake 90 07/01/2018	0.64	7.33	22.3	17.6	18.1	1.27	2.34	1.67	0.89	0.66	2.89
Lake 90 08/12/2018	1.22	6.51	19.3	19.9	18.4	0.97	3.22	1.53	0.94	0.51	4.67
Lake 90 09/11/2018	1.79	6.04	17.8	18.9	17.6	0.94	3.44	1.54	0.94	0.50	4.92
Lake 90 06/08/2019	1.37	5.88	17.1	18.2	17.2	0.94	3.04	1.46	0.94	0.47	3.66
Lake 90 08/17/2019	1.15	7.19	21.1	18.5	18.4	1.14	3.05	1.54	0.91	0.52	3.29
Lake 92 08/06/2018	0.01	8.85	32.1	28.2	25.9	1.14	1.64	1.95	0.79	0.82	1.60
Lake 92 08/19/2018	0.04	13.39	29.3	25.4	24.9	1.15	1.52	1.95	0.84	0.76	2.05
Lake 92 09/03/2018	0.29	8.57	23.8	19.9	20.0	1.20	1.93	1.68	0.89	0.64	3.17
Lake 92 06/04/2019	0.01	12.14	28.0	36.5	29.5	0.77	1.44	1.68	0.83	0.77	1.50
Lake 92 06/17/2019	0.01	11.22	25.6	26.3	24.7	0.97	1.56	1.70	0.82	0.71	1.56
Lake 92 08/11/2019	0.19	11.47	28.8	18.7	21.2	1.54	1.58	1.76	0.82	0.74	1.48
Lake 92 09/01/2019	0.34	8.85	27.0	14.2	17.5	1.90	1.51	1.72	0.81	0.73	1.44
Lake 96 08/05/2019	0.50	7.95	23.0	16.7	18.1	1.38	2.02	1.73	0.85	0.69	1.89
Lake 99 08/10/2019	1.94	5.98	17.8	18.5	17.4	0.96	3.43	1.49	0.93	0.45	4.62
Lake 100 06/23/2019	0.99	5.87	17.3	19.3	17.7	0.89	2.83	1.47	0.94	0.46	4.41
Lake 100 08/18/2019	0.88	6.80	20.0	18.1	17.9	1.11	2.62	1.58	0.91	0.52	4.25
Lake 102 06/16/2019	0.61	7.49	20.1	18.3	18.6	1.10	2.45	1.71	0.85	0.67	1.82
Lake 102 09/22/2019	1.30	7.03	20.4	16.3	17.4	1.25	2.95	1.79	0.83	0.79	1.34
Lake 103 06/09/2019	2.66	5.01	16.3	15.1	15.1	1.08	3.29	1.51	0.92	0.50	3.42
Lake 107 08/15/2019	0.42	8.08	22.8	20.0	19.7	1.14	2.22	1.59	0.85	0.61	2.02
Lake 108 08/17/2019	0.32	9.16	25.0	20.2	20.4	1.24	2.07	1.67	0.86	0.59	2.28

Table S4. Optical properties of whole water samples (continued)											
Sample ID [Lake ID Sampling Date]	a_{440} (m^{-1})	$E2{:}E3$	$S_{275–295}$ (μm^{-1})	$S_{350–400}$ (μm^{-1})	$S_{290–400}$ (μm^{-1})	S_R	$SUVA_{254}$ (L mg C^{-1}•m^{-1})	FI	HIX	$\beta{:}\alpha$	Peak M:T
Lake 109 07/08/2019	1.89	6.70	19.0	20.2	18.9	0.94	3.69	1.57	0.93	0.52	3.35
Lake 109 08/18/2019	1.71	7.67	21.3	19.4	19.4	1.10	3.55	1.63	0.90	0.59	2.75
Lake 115 07/08/2019	0.16	8.98	23.6	24.9	22.4	0.95	2.33	1.70	0.89	0.61	2.01
Lake 115 07/08/2019	0.38	8.91	23.2	21.0	20.7	1.11	2.35	1.61	0.88	0.66	2.14
Lake 115 09/09/2019	0.78	7.82	22.8	16.3	17.8	1.40	2.41	1.66	0.88	0.67	2.58
Lake 115 09/09/2019	0.80	7.70	22.6	16.1	17.7	1.40	2.41	1.65	0.89	0.69	2.60
Lake 117 08/22/2019	0.56	7.44	21.6	19.1	18.9	1.13	2.39	1.55	0.87	0.54	2.45
Lake 117 09/05/2019	0.43	7.70	21.9	19.9	19.3	1.10	2.25	1.58	0.87	0.56	2.62
Lake 120 06/10/2019	0.50	7.48	20.7	20.0	19.5	1.03	2.40	1.55	0.89	0.56	2.44
Lake 120 08/19/2019	0.50	8.83	23.9	18.9	19.9	1.26	2.37	1.59	0.85	0.61	2.22
Lake 126 07/08/2018	0.19	9.25	24.4	22.4	21.6	1.09	1.80	1.57	0.89	0.62	2.86
Lake 126 07/21/2018	0.12	7.28	16.5	25.4	21.8	0.65	2.24	1.70	0.92	0.63	2.98
Lake 126 08/17/2019	0.44	7.88	23.3	18.1	18.8	1.29	2.03	1.65	0.86	0.59	2.21
Lake 130 08/25/2019	0.45	8.53	24.3	18.3	19.2	1.33	2.16	1.63	0.84	0.65	2.12
Lake 130 09/22/2019	0.35	8.87	24.1	20.3	20.4	1.18	2.21	1.57	0.82	0.66	1.64
Lake 132 06/10/2019	0.64	6.01	19.7	17.6	17.0	1.12	2.17	1.63	0.86	0.59	1.60
Lake 132 09/03/2019	0.60	7.40	23.1	16.1	17.5	1.43	2.09	1.75	0.82	0.83	1.57
Lake 133 06/04/2019	0.69	8.77	27.1	16.1	18.3	1.68	2.49	1.60	0.74	0.77	0.91
Lake 133 08/19/2019	0.44	11.42	29.0	18.4	20.7	1.58	2.41	1.69	0.79	0.77	1.40
Lake 135 05/27/2019	0.85	6.23	17.9	19.2	18.0	0.94	2.70	1.46	0.94	0.47	3.53
Lake 135 08/31/2019	0.47	8.44	22.5	20.9	20.4	1.08	2.52	1.55	0.90	0.56	3.50
Lake 136 06/08/2019	0.69	7.43	21.4	17.5	18.2	1.22	2.42	1.54	0.86	0.59	1.97
Lake 136 09/20/2019	0.48	9.23	24.4	18.7	19.8	1.31	2.34	1.60	0.85	0.67	1.83
Lake 137 08/18/2019	0.13	13.13	29.7	21.8	23.1	1.36	1.75	1.61	0.80	0.62	1.48
Lake 139 08/06/2018	0.61	5.08	19.5	11.7	13.0	1.67	1.44	1.85	0.78	0.56	1.68
Lake 139 09/19/2018	0.39	6.29	24.5	12.8	14.6	1.91	1.03	1.42	0.76	0.78	2.27
Lake 145 06/06/2019	1.26	5.83	16.8	18.7	17.4	0.90	3.05	1.49	0.92	0.44	3.55
Lake 145 08/15/2019	1.16	6.59	19.3	18.8	18.2	1.03	3.02	1.46	0.91	0.49	3.75
Lake 149 06/10/2019	0.50	7.34	20.9	19.9	19.1	1.05	2.37	1.52	0.86	0.54	2.17
Lake 149 09/21/2019	0.10	11.67	26.4	25.1	23.9	1.05	1.91	1.59	0.82	0.58	1.82
Lake 153 08/25/2019	0.53	8.04	23.3	17.7	18.6	1.32	2.14	1.53	0.87	0.59	2.31
Lake 153 09/08/2019	0.71	7.20	22.3	15.4	17.0	1.44	2.12	1.67	0.88	0.62	2.46
Lake 164 06/23/2019	0.55	8.03	23.0	19.2	19.6	1.19	2.47	1.75	0.78	0.69	1.14
Lake 164 08/27/2019	0.91	5.96	16.0	20.6	18.5	0.78	3.00	1.62	0.95	0.55	4.92
Lake 166 07/14/2019	0.29	9.95	26.1	20.1	21.1	1.30	2.11	1.68	0.77	0.70	1.57
Lake 166 09/29/2019	0.18	10.61	25.8	23.1	22.6	1.12	2.14	1.67	0.81	0.71	1.62
Lake 169 07/22/2019	0.31	8.90	23.5	21.7	21.1	1.08	2.35	1.55	0.85	0.59	2.32
Lake 169 08/18/2019	0.31	9.58	25.0	21.1	21.2	1.19	2.23	1.57	0.84	0.59	2.49

**Table S4.** Optical properties of whole water samples (continued)											
Sample ID [Lake ID Sampling Date]	a_{440} (m^{-1})	*E2:E3*	$S_{275–295}$ (μm^{-1})	$S_{350–400}$ (μm^{-1})	$S_{290–400}$ (μm^{-1})	S_R	$SUVA_{254}$ (L mg C^{-1}•m^{-1})	FI	HIX	$\beta:\alpha$	Peak M:T
Lake 176 07/15/2018	0.77	6.45	19.1	19.4	18.2	0.98	2.66	1.48	0.92	0.49	4.70
Lake 176 06/15/2019	1.43	5.47	16.0	18.4	16.9	0.87	3.04	1.44	0.94	0.42	4.81
Lake 176 08/19/2019	1.25	6.02	17.8	18.6	17.6	0.95	2.99	1.49	0.93	0.44	5.01
Lake 177 08/19/2019	0.81	6.64	19.4	19.1	18.4	1.01	2.68	1.56	0.90	0.56	3.26
Lake 178 08/06/2019	0.50	9.28	25.0	18.7	19.9	1.34	2.38	1.56	0.87	0.67	2.26
Lake 182 07/17/2018	0.09	11.77	24.8	24.5	23.9	1.01	1.76	1.74	0.89	0.71	2.74
Lake 182 07/31/2018	0.08	9.82	22.6	26.2	23.6	0.86	2.16	1.74	0.77	0.64	0.93
Lake 182 08/14/2018	0.09	10.05	23.8	25.9	23.7	0.92	1.90	1.58	0.88	0.69	2.57
Lake 182 09/11/2018	0.01	11.48	24.4	33.0	27.6	0.74	1.80	1.65	0.89	0.67	2.98
Lake 182 06/18/2019	0.24	9.32	24.9	19.2	20.3	1.30	1.72	1.72	0.81	0.69	1.24
Lake 182 06/18/2019	0.37	8.50	24.0	16.9	18.8	1.42	1.77	1.71	0.83	0.70	1.29
Lake 182 07/01/2019	0.22	7.79	20.7	23.2	21.0	0.89	2.21	1.64	0.91	0.60	2.76
Lake 182 07/08/2019	0.28	9.76	25.6	19.6	20.6	1.31	2.02	1.63	0.84	0.69	1.58
Lake 182 08/12/2019	0.16	12.17	26.5	21.8	22.9	1.22	1.74	1.84	0.83	0.74	1.77
Lake 182 08/12/2019	0.28	10.17	26.0	18.0	20.3	1.44	1.74	1.73	0.84	0.70	1.87
Lake 182 08/27/2019	0.49	7.89	21.9	16.7	18.2	1.31	1.90	1.92	0.87	0.69	2.24
Lake 183 08/25/2019	0.54	7.40	21.5	18.8	18.6	1.14	2.24	1.58	0.89	0.53	3.02
Lake 183 09/10/2019	0.55	7.84	22.3	19.6	19.2	1.14	2.43	1.59	0.88	0.57	2.85
Lake 190 06/17/2019	1.21	7.24	20.1	19.4	19.0	1.03	3.22	1.90	0.91	0.58	2.44
Lake 190 08/19/2019	1.24	8.33	22.8	18.1	19.2	1.26	3.20	1.64	0.85	0.65	1.73
Lake 192 09/25/2019	1.53	5.95	17.4	18.4	17.4	0.95	3.13	1.53	0.90	0.50	2.62
Lake 194 06/26/2018	0.77	6.86	20.8	19.3	18.4	1.08	2.81	1.52	0.91	0.60	2.93
Lake 194 07/09/2018	0.80	6.61	19.4	20.7	18.9	0.93	2.92	1.46	0.90	0.51	3.35
Lake 194 07/23/2018	0.34	8.62	22.6	21.1	20.5	1.07	2.19	1.65	0.90	0.64	3.24
Lake 194 08/07/2018	0.30	8.23	22.3	22.5	20.8	0.99	2.27	1.59	0.90	0.63	3.14
Lake 194 08/20/2018	0.46	7.81	20.7	20.8	19.8	1.00	2.42	1.65	0.91	0.61	3.30
Lake 194 09/09/2018	0.35	8.10	21.3	22.1	20.7	0.96	2.40	1.61	0.92	0.59	3.73
Lake 194 09/22/2019	0.40	9.23	22.7	22.1	21.5	1.03	2.58	1.61	0.87	0.62	2.05
Lake 195 06/03/2019	2.99	7.34	18.4	13.2	14.7	1.39	3.44	2.39	0.82	0.43	3.57
Lake 199 06/19/2018	0.71	6.61	21.1	13.3	15.7	1.59	1.93	1.72	0.88	0.69	2.68
Lake 199 07/03/2018	0.01	22.68	25.7	42.9	34.0	0.19	0.88	1.93	0.92	0.53	4.61
Lake 199 07/18/2018	0.09	10.67	28.2	21.7	21.9	1.30	1.27	1.63	0.78	0.71	1.37
Lake 199 07/31/2018	0.29	6.47	23.7	15.5	16.2	1.53	1.40	1.66	0.81	0.68	1.51
Lake 199 08/14/2018	0.22	8.07	26.4	18.1	18.7	1.46	1.36	1.65	0.74	0.74	1.39
Lake 199 08/28/2018	0.06	10.12	26.5	25.9	23.7	1.02	1.81	1.62	0.84	0.71	1.95
Lake 199 09/11/2018	0.01	10.90	28.2	30.1	25.9	0.94	1.26	1.64	0.79	0.70	1.84
Lake 199 06/23/2019	0.01	11.33	27.6	35.0	21.1	0.21	0.88	1.37	0.91	0.55	1.60
Lake 199 07/08/2019	0.12	9.58	25.8	21.0	21.1	1.23	1.40	1.50	0.75	0.67	1.50

**Table S4.** Optical properties of whole water samples (continued)											
Sample ID [Lake ID Sampling Date]	a_{440} (m^{-1})	$E2:E3$	$S_{275–295}$ (μm^{-1})	$S_{350–400}$ (μm^{-1})	$S_{290–400}$ (μm^{-1})	S_R	$SUVA_{254}$ (L mg C^{-1}•m^{-1})	FI	HIX	$\beta:\alpha$	Peak M:T
Lake 199 07/30/2019	0.37	7.83	24.9	14.6	17.1	1.70	1.47	1.56	0.81	0.60	1.66
Lake 199 08/18/2019	0.10	11.71	30.0	20.8	22.0	1.44	1.25	1.44	0.78	0.66	1.84
Lake 199 09/15/2019	0.01	18.84	32.1	38.0	32.3	0.84	1.22	1.62	0.73	0.66	1.83
Lake 203 06/16/2019	0.98	6.42	20.1	17.0	17.2	1.18	2.66	1.67	0.88	0.62	2.19
Lake 203 09/22/2019	0.63	8.40	22.9	19.1	19.5	1.20	2.63	1.62	0.87	0.68	2.27
Lake 205 06/08/2019	1.11	6.08	18.6	17.6	17.3	1.06	2.83	1.50	0.90	0.54	2.46
Lake 205 08/06/2019	1.06	7.00	21.2	17.6	18.0	1.20	2.86	1.56	0.88	0.60	2.18
Lake 209 08/18/2019	0.64	6.01	18.2	18.9	17.7	0.96	2.29	1.63	0.91	0.55	3.08
Lake 210 06/02/2019	1.33	5.57	17.3	17.0	16.5	1.02	2.89	1.53	0.90	0.53	2.41
Lake 210 08/18/2019	1.97	5.53	17.4	16.2	16.1	1.07	3.17	1.55	0.90	0.53	2.64
Lake 212 06/16/2018	0.05	11.32	27.4	24.0	23.4	1.14	1.31	1.65	0.82	0.69	1.99
Lake 212 07/14/2018	0.65	8.86	24.5	19.9	19.9	1.23	2.73	1.58	0.90	0.63	3.24
Lake 212 07/28/2018	0.62	8.68	23.7	19.9	20.0	1.19	2.73	1.61	0.90	0.64	2.85
Lake 212 08/12/2018	0.47	9.20	24.3	21.3	20.8	1.14	2.69	1.62	0.90	0.62	3.18
Lake 212 06/17/2019	1.37	6.35	20.3	16.4	16.8	1.24	2.99	1.60	0.88	0.58	2.18
Lake 213 08/18/2019	0.58	7.50	22.1	17.5	18.3	1.26	2.29	1.80	0.85	0.64	1.74
Lake 215 06/09/2019	1.19	5.52	16.4	17.7	16.8	0.93	2.83	1.43	0.90	0.49	2.53
Lake 215 09/15/2019	1.47	5.84	17.3	17.2	16.8	1.01	2.96	1.47	0.90	0.50	2.93
Lake 223 08/19/2019	0.98	8.45	21.8	18.9	19.5	1.16	3.02	1.60	0.90	0.64	2.92
Lake 225 07/28/2019	1.15	6.55	19.8	17.9	17.7	1.11	2.93	1.52	0.91	0.55	3.18
Lake 229 06/29/2019	0.48	7.08	22.0	19.0	18.7	1.16	2.24	1.58	0.88	0.62	2.02
Lake 230 06/08/2019	0.62	6.84	19.2	21.0	19.6	0.92	2.78	1.61	0.90	0.61	2.43
Lake 231 06/04/2019	0.93	7.59	20.9	18.4	18.8	1.13	2.86	1.58	0.84	0.74	1.85
Lake 234 06/20/2018	0.16	10.47	23.6	22.8	22.5	1.03	1.88	1.70	0.89	0.66	3.07
Lake 234 07/15/2018	0.10	10.62	25.1	24.8	23.4	1.01	1.81	1.85	0.84	0.72	2.34
Lake 234 06/09/2019	0.60	6.35	20.3	17.5	18.0	1.16	2.09	1.89	0.81	0.63	1.20
Lake 234 08/12/2019	0.48	7.71	22.3	16.8	18.2	1.33	1.97	1.70	0.82	0.67	1.60
Lake 235 07/14/2019	1.46	6.21	18.6	18.4	17.6	1.01	3.18	1.48	0.92	0.50	3.28
Lake 236 07/14/2019	0.13	8.62	24.1	25.6	22.6	0.94	2.11	1.53	0.87	0.60	2.36
Lake 236 09/15/2019	0.59	7.22	22.1	17.1	17.7	1.30	2.13	1.62	0.83	0.61	1.70
Lake 238 08/12/2019	1.30	7.10	20.1	19.1	18.7	1.05	3.21	1.54	0.91	0.56	3.22
Lake 239 06/09/2019	0.49	7.15	20.8	20.0	19.2	1.04	2.44	1.68	0.84	0.68	1.44
Lake 239 08/18/2019	0.71	8.16	22.9	17.8	18.8	1.29	2.65	1.76	0.86	0.69	1.76
Lake 245 09/22/2019	0.50	8.16	21.6	18.4	19.2	1.18	2.12	1.63	0.87	0.65	2.52
Lake 246 08/05/2019	2.26	5.94	17.7	17.1	16.8	1.03	3.43	1.64	0.90	0.64	2.62
Lake 247 06/08/2019	0.75	6.62	19.6	18.8	18.2	1.05	2.55	1.54	0.90	0.55	2.52
Lake 247 09/20/2019	0.46	8.19	21.9	21.2	20.4	1.03	2.55	1.58	0.89	0.59	2.63
Lake 248 08/18/2019	2.10	5.86	18.5	16.5	16.4	1.12	3.30	1.59	0.91	0.60	2.99

**Table S4.** Optical properties of whole water samples (continued)											
Sample ID [Lake ID Sampling Date]	a_{440} (m^{-1})	*E2:E3*	$S_{275–295}$ (μm^{-1})	$S_{350–400}$ (μm^{-1})	$S_{290–400}$ (μm^{-1})	S_R	$SUVA_{254}$ (L mg C^{-1}•m^{-1})	FI	HIX	*β:α*	Peak M:T
Lake 249 07/08/2019	0.47	7.40	22.0	18.9	18.8	1.17	2.22	1.76	0.83	0.67	1.69
Lake 249 09/01/2019	0.71	7.00	21.3	17.6	17.9	1.21	2.46	1.71	0.88	0.69	2.70
Lake 250 07/08/2019	0.16	8.01	24.0	24.4	21.5	0.98	2.06	1.61	0.75	0.71	1.33
Lake 250 08/18/2019	0.56	7.77	23.5	16.5	18.0	1.42	2.13	1.72	0.77	0.69	1.44
Lake 251 08/27/2019	1.36	7.31	20.5	18.7	18.7	1.10	3.28	1.73	0.89	0.66	2.72
Lake 251 09/17/2019	2.30	6.66	20.9	15.3	16.6	1.37	3.37	1.74	0.81	0.72	1.25
Lake 253 07/02/2019	0.01	14.79	28.4	29.1	27.2	0.98	1.72	1.64	0.81	0.68	1.86
Lake 253 08/25/2019	0.35	9.85	27.3	15.9	19.1	1.72	1.75	1.67	0.81	0.71	1.53
Lake 253 09/15/2019	0.03	16.22	30.3	28.1	27.3	1.08	1.64	1.70	0.78	0.76	1.09
Lake 256 07/05/2018	0.12	10.68	25.1	23.8	23.0	1.05	1.82	1.72	0.87	0.72	2.35
Lake 256 07/16/2018	0.24	10.03	24.9	19.7	20.7	1.27	1.77	1.64	0.88	0.73	2.49
Lake 256 07/31/2018	0.16	10.59	25.6	21.5	21.9	1.19	1.82	1.68	0.88	0.72	2.63
Lake 256 08/15/2018	0.20	8.68	23.9	21.2	20.6	1.13	1.83	1.71	0.88	0.73	2.65
Lake 256 08/29/2018	0.67	6.66	19.4	19.5	18.4	0.99	2.51	1.46	0.92	0.54	4.47
Lake 256 09/14/2018	0.10	9.97	24.8	25.1	23.2	0.98	1.86	1.74	0.87	0.72	2.74
Lake 256 09/28/2018	0.01	11.22	25.3	32.1	26.8	0.79	1.77	1.70	0.99	0.73	4.95
Lake 256 06/18/2019	0.29	8.13	20.9	19.5	20.0	1.07	1.83	1.68	0.85	0.69	1.56
Lake 256 06/18/2019	0.57	6.35	20.0	15.5	16.5	1.29	1.91	1.66	0.85	0.67	1.74
Lake 256 08/13/2019	0.27	9.46	24.1	19.9	20.6	1.21	1.82	1.69	0.88	0.72	2.30
Lake 257 08/24/2019	3.35	6.05	18.4	18.2	17.4	1.01	3.83	1.58	0.93	0.56	3.83
Lake 258 06/16/2019	0.89	6.79	20.9	17.4	17.7	1.20	2.63	1.58	0.89	0.62	2.44
Lake 258 08/11/2019	1.54	5.81	20.0	14.6	15.6	1.37	2.81	1.59	0.87	0.62	2.06
Lake 259 09/01/2019	1.93	6.60	19.6	18.4	18.0	1.06	3.46	1.60	0.87	0.56	2.06
Lake 260 06/01/2019	1.13	5.58	16.4	18.5	17.3	0.88	2.87	1.53	0.92	0.50	3.05
Lake 260 08/12/2019	1.08	6.81	19.8	18.9	18.3	1.05	2.99	1.59	0.91	0.60	3.10
Lake 261 06/17/2019	0.86	6.50	19.3	19.3	18.2	1.00	2.79	1.59	0.88	0.57	2.10
Lake 261 08/12/2019	0.87	7.32	21.4	17.7	18.1	1.21	2.72	1.61	0.87	0.62	1.97
Lake 262 06/17/2019	2.62	5.31	15.5	18.0	16.6	0.86	3.55	1.40	0.94	0.39	5.03
Lake 262 09/21/2019	1.65	6.17	17.8	19.4	18.0	0.91	3.39	1.45	0.93	0.45	4.29

a_{440} = Napierian absorption coefficient at 440 nm; *E2:E3* = the ratio of absorption coefficients at 250 and 365 nm; $S_{275–295}$ = the spectral slope coefficient from 275 to 295 nm; $S_{350–400}$ = the spectral slope coefficient from 350 to 400 nm; $S_{290–400}$ = the spectral slope coefficient from 290 to 400 nm; S_R = the ratio of $S_{275-295}$ to $S_{350-400}$; $SUVA_{254}$ = specific UV absorbance at 254 nm; FI = fluorescence index; HIX = humification index; *β:α* = freshness index; Peak M:T = the ratio of microbial humic-like to protein-like DOM fluorescence.

**Table S5.** Physicochemical and optical properties of bloom lysates												
Sample ID	Bloom ID	OD_{680} (A.U.)	pH	*E2:E3*	$S_{290–400}$ (μm^{-1})	$SUVA_{254}$ (L mg C^{-1}•m^{-1})	FI	HIX	*β:α*	Peak M:T	AOC (mmol e^{-}/g C)	DOC (mg C/L)
Lake 261 09/04/2021	A	0.376±0.009	7.1	2.93±0.02	7.5±0.1	0.68±0.01	2.24±0.05	0.48±0.03	0.94±0.01	0.12±0.01	0.82±0.09	3.02±0.08
Lake 238 09/07/2021	B	0.349±0.018	7.2	2.88±0.09	7.4±0.2	0.65±0.03	2.24±0.09	0.42±0.01	0.88±0.01	0.11±0.01	0.86±0.06	3.02±0.05
Lake 147 08/30/2021	C	0.370±0.019	7.9	2.72±0.41	7.2±1.4	0.67±0.03	2.14±0.02	0.41±0.01	0.89±0.02	0.09±0.01	0.78±0.09	3.01±0.07
Lake 138 09/07/2021	D	0.417±0.026	7.3	2.98±0.05	8.1±0.2	0.57±0.01	2.20±0.07	0.42±0.01	0.93±0.04	0.13±0.02	0.72±0.06	3.05±0.05
Lake 33 09/04/2021	E	0.368±0.018	7.4	2.76±0.20	6.6±0.1	0.65±0.02	2.49±0.01	0.40±0.01	0.90±0.04	0.15±0.01	0.80±0.08	3.04±0.07
Lake 37 08/20/2021	F	0.267±0.013	7.8	2.91±0.09	7.6±0.1	0.50±0.03	2.11±0.04	0.53±0.01	0.94±0.01	0.14±0.01	0.83±0.13	3.05±0.06
Lake 38 08/29/2021	G	0.430±0.022	7.6	2.91±0.07	8.0±0.3	0.73±0.02	2.12±2.12	0.43±0.43	0.92±0.92	0.10±0.01	0.72±0.06	3.03±0.03
Lake 40 09/16/2021	H	0.392±0.020	7.9	2.75±0.17	7.2±0.6	0.61±0.03	2.12±0.05	0.48±0.01	0.87±0.03	0.13±0.01	0.65±0.07	3.02±0.08
Lake 78 08/22/2021	I	0.410±0.020	7.9	2.85±0.01	7.7±0.1	0.54±0.01	2.23±0.05	0.53±0.01	0.92±0.01	0.12±0.02	0.68±0.05	3.03±0.03
Lake 82 08/17/2021	J	0.475±0.024	7.5	2.80±0.27	7.6±0.7	0.46±0.02	2.37±0.03	0.49±0.04	0.91±0.03	0.14±0.01	0.75±0.06	3.01±0.07
Lake 93 08/29/2021	K	0.245±0.012	7.1	3.01±0.03	8.3±0.2	0.46±0.01	2.15±0.03	0.47±0.01	0.89±0.03	0.17±0.01	0.91±0.11	3.02±0.07
Lake 221 09/06/2021	L	0.542±0.027	7.7	2.84±0.16	7.4±0.5	0.68±0.03	2.14±0.03	0.46±0.01	0.92±0.01	0.13±0.01	0.61±0.04	3.02±0.08
Lake 256 10/12/2021	Otisco	0.026±0.001	7.2	8.20±0.02	18.8±0.1	1.61±0.08	1.75±0.09	0.78±0.04	0.95±0.05	3.11±0.02	1.31±0.11	2.76±0.14
Lysate 25% + Otisco 75%	A	-	7.8	6.58±0.07	15.5±0.1	1.26±0.01	1.86±0.01	0.69±0.01	0.95±0.01	1.27±0.02	-	3.03±0.04
Lysate 50% + Otisco 50%	A	-	7.6	5.16±0.13	12.5±0.1	1.06±0.01	2.00±0.01	0.59±0.01	0.95±0.01	0.54±0.01	-	3.04±0.03
Lysate 75% + Otisco 25%	A	-	6.8	3.89±0.18	9.6±0.1	0.86±0.01	2.20±0.01	0.49±0.01	0.95±0.01	0.23±0.02	-	3.01±0.07
Lysate 25% + Otisco 75%	B	-	7.9	6.95±0.07	16.5±0.1	1.22±0.02	1.78±0.01	0.77±0.01	0.95±0.01	1.21±0.01	-	3.05±0.03
Lysate 50% + Otisco 50%	B	-	7.4	5.65±0.12	13.9±0.1	0.99±0.02	1.82±0.01	0.76±0.01	0.95±0.01	0.50±0.01	-	3.02±0.04
Lysate 75% + Otisco 25%	B	-	7.2	4.31±0.13	11.1±0.1	0.75±0.03	1.89±0.03	0.72±0.01	0.95±0.01	0.22±0.03	-	3.03±0.06
Lysate 25% + Otisco 75%	C	-	7.3	6.66±0.03	15.8±0.1	1.26±0.02	1.81±0.01	0.74±0.02	0.95±0.01	1.12±0.02	-	3.04±0.05
Lysate 50% + Otisco 50%	C	-	7.1	5.29±0.03	13.0±0.1	1.07±0.01	1.90±0.01	0.67±0.03	0.95±0.01	0.46±0.01	-	3.02±0.04
Lysate 75% + Otisco 25%	C	-	6.9	4.05±0.01	10.3±0.1	0.88±0.01	2.02±0.01	0.59±0.04	0.95±0.01	0.19±0.02	-	3.01±0.04
Lysate 25% + Otisco 75%	D	-	7.7	6.65±0.07	15.8±0.1	1.26±0.04	1.79±0.01	0.76±0.01	0.95±0.01	1.22±0.03	-	3.03±0.06
Lysate 50% + Otisco 50%	D	-	7.1	5.27±0.11	13.0±0.2	1.05±0.03	1.86±0.02	0.71±0.01	0.95±0.01	0.51±0.01	-	3.04±0.04
Lysate 75% + Otisco 25%	D	-	7.0	4.01±0.11	10.2±0.2	0.85±0.04	1.97±0.04	0.63±0.01	0.94±0.02	0.20±0.01	-	3.05±0.07
Lysate 25% + Otisco 75%	E	-	7.2	6.51±0.30	15.6±0.6	1.27±0.03	1.78±0.01	0.75±0.01	0.95±0.01	1.31±0.01	-	3.01±0.04
Lysate 50% + Otisco 50%	E	-	7.0	5.07±0.42	12.8±1.0	1.07±0.02	1.83±0.01	0.69±0.01	0.95±0.02	0.57±0.01	-	3.02±0.03
Lysate 75% + Otisco 25%	E	-	6.9	3.82±0.45	10.0±1.2	0.87±0.03	1.91±0.01	0.60±0.01	0.95±0.02	0.24±0.01	-	3.02±0.05
Lysate 25% + Otisco 75%	F	-	7.1	6.85±0.01	16.3±0.1	1.24±0.02	1.78±0.01	0.75±0.01	0.95±0.01	1.31±0.02	-	3.04±0.03
Lysate 50% + Otisco 50%	F	-	7.0	5.53±0.02	13.8±0.1	1.02±0.01	1.83±0.01	0.70±0.01	0.95±0.01	0.55±0.01	-	3.03±0.04
Lysate 75% + Otisco 25%	F	-	6.8	4.24±0.03	11.1±0.1	0.80±0.01	1.92±0.02	0.61±0.01	0.95±0.02	0.22±0.01	-	3.02±0.03

**Table S5.** Physicochemical and optical properties of bloom lysates (continued)												
Sample ID	Bloom ID	OD_{680} (A.U.)	pH	*E2:E3*	$S_{290–400}$ (μm^{-1})	$SUVA_{254}$ (L mg $C^{-1}\bullet m^{-1}$)	FI	HIX	$\beta:\alpha$	Peak M:T	AOC (mmol e^-/g C)	DOC (mg C/L)
SRNOM	SRNOM	-	7.8	4.69±0.01	15.5±0.1	3.97±0.05	1.37±0.01	0.96±0.01	0.34±0.01	15.85±4.56	2.20±0.32	3.03±0.04
Lysate 25% + SRNOM 75%	A	-	7.8	4.53±0.01	14.7±0.1	3.15±0.02	1.57±0.12	0.74±0.10	0.37±0.03	0.68±0.47	-	3.02±0.03
Lysate 50% + SRNOM 50%	A	-	7.6	4.27±0.05	13.4±0.1	2.31±0.01	1.79±0.19	0.60±0.10	0.44±0.05	0.26±0.11	-	3.04±0.04
Lysate 75% + SRNOM 25%	A	-	6.8	3.81±0.10	11.3±0.1	1.49±0.01	1.71±0.32	0.67±0.20	0.43±0.09	0.57±0.55	-	3.01±0.03
Lysate 25% + SRNOM 75%	B	-	7.9	4.58±0.01	14.9±0.1	3.08±0.05	1.41±0.01	0.95±0.01	0.35±0.01	0.93±0.02	-	3.05±0.05
Lysate 50% + SRNOM 50%	B	-	7.4	4.39±0.03	14.1±0.2	2.23±0.01	1.47±0.01	0.92±0.01	0.37±0.01	0.31±0.01	-	3.04±0.01
Lysate 75% + SRNOM 25%	B	-	7.2	4.02±0.06	12.4±0.2	1.38±0.02	1.60±0.03	0.85±0.01	0.43±0.01	0.17±0.01	-	3.01±0.02
Lysate 25% + SRNOM 75%	C	-	7.3	4.54±0.01	14.8±0.1	3.16±0.04	1.45±0.01	0.88±0.02	0.35±0.01	0.83±0.03	-	3.02±0.05
Lysate 50% + SRNOM 50%	C	-	7.1	4.31±0.01	13.7±0.1	2.32±0.02	1.57±0.02	0.78±0.04	0.37±0.01	0.28±0.01	-	3.03±0.04
Lysate 75% + SRNOM 25%	C	-	6.9	3.90±0.01	11.8±0.1	1.50±0.01	1.77±0.02	0.65±0.05	0.44±0.02	0.14±0.01	-	3.04±0.03
Lysate 25% + SRNOM 75%	D	-	7.7	4.54±0.01	14.8±0.2	3.12±0.01	1.42±0.01	0.92±0.01	0.35±0.01	0.94±0.04	-	3.05±0.01
Lysate 50% + SRNOM 50%	D	-	7.1	4.31±0.03	13.7±0.2	2.32±0.07	1.50±0.01	0.85±0.01	0.38±0.01	0.31±0.01	-	3.02±0.08
Lysate 75% + SRNOM 25%	D	-	7.0	3.88±0.06	11.7±0.2	1.49±0.05	1.68±0.02	0.72±0.01	0.44±0.02	0.14±0.01	-	3.02±0.07
Lysate 25% + SRNOM 75%	E	-	7.2	4.51±0.06	14.7±0.1	3.14±0.04	1.41±0.01	0.90±0.01	0.37±0.01	1.07±0.02	-	3.03±0.04
Lysate 50% + SRNOM 50%	E	-	7.0	4.24±0.14	13.5±0.4	2.31±0.03	1.48±0.01	0.82±0.01	0.42±0.01	0.39±0.01	-	3.04±0.03
Lysate 75% + SRNOM 25%	E	-	6.9	3.77±0.26	11.5±0.8	1.48±0.02	1.62±0.02	0.68±0.01	0.52±0.01	0.17±0.01	-	3.04±0.02
Lysate 25% + SRNOM 75%	F	-	7.1	4.57±0.01	14.9±0.1	3.13±0.01	1.41±0.01	0.91±0.01	0.35±0.01	1.03±0.05	-	3.02±0.01
Lysate 50% + SRNOM 50%	F	-	7.0	4.37±0.01	14.0±0.1	2.29±0.05	1.47±0.01	0.83±0.01	0.37±0.01	0.34±0.02	-	3.01±0.07
Lysate 75% + SRNOM 25%	F	-	6.8	3.99±0.02	12.4±0.1	1.42±0.02	1.61±0.01	0.69±0.01	0.43±0.01	0.16±0.02	-	3.03±0.06

OD_{680} = optical density at 680 nm; *E2:E3* = the ratio of absorption coefficients at 250 and 365 nm; $S_{290–400}$ = the spectral slope coefficient from 290 to 400 nm; $SUVA_{254}$ = specific UV absorbance at 254 nm; FI = fluorescence index; HIX = humification index; $\beta:\alpha$ = freshness index; Peak M:T = the ratio of microbial humic-like to protein-like DOM fluorescence; AOC = antioxidant capacity.

**Table S6.** Physicochemical and optical properties of bloom supernatants													
Sample ID [Lake ID Sampling Date]	Bloom ID	OD_{680} (A.U.)	pH	*E2:E3*	$S_{290–400}$ (μm^{-1})	$SUVA_{254}$ (L mg C^{-1}•m^{-1})	FI	HIX	*β:α*	Peak M:T	Chl-*a* (µg/L)	Chl-a_{cyano} (µg/L)	DOC (mg C/L)
Lake 261 09/04/2021	A	0.151±0.003	7.3	5.10±0.11	13.6±0.1	1.88±0.14	1.98±0.14	0.85±0.06	0.91±0.07	1.15±0.05	-	-	3.15±0.23
	A	0.182±0.006	7.3	4.90±0.08	13.3±0.1	1.92±0.01	2.01±0.01	0.86±0.01	0.84±0.01	1.20±0.01	-	-	3.22±0.02
	A	0.215±0.008	7.0	4.62±0.07	12.8±0.1	1.98±0.08	2.04±0.09	0.87±0.04	0.82±0.03	1.29±0.05	-	-	3.33±0.14
	A	0.259±0.009	7.2	4.07±0.06	11.7±0.1	2.10±0.14	2.12±0.14	0.90±0.06	0.81±0.06	1.35±0.05	-	-	3.59±0.24
	A	0.306±0.009	7.2	3.62±0.05	10.7±0.1	2.18±0.04	2.14±0.04	0.90±0.02	0.77±0.01	1.39±0.04	-	-	3.86±0.07
	A	0.347±0.009	7.1	3.34±0.04	10.0±0.1	2.21±0.11	2.19±0.11	0.92±0.05	0.75±0.04	1.47±0.12	-	-	4.08±0.20
	A	0.370±0.009	7.1	3.25±0.04	9.7±0.1	2.22±0.15	2.20±0.15	0.93±0.06	0.73±0.05	1.50±0.09	-	-	4.15±0.28
	A	0.376±0.009	7.1	3.23±0.04	9.7±0.1	2.21±0.04	2.20±0.04	0.93±0.02	0.71±0.01	1.55±0.06	-	-	4.17±0.07
	A	0.379±0.009	7.2	3.20±0.04	9.6±0.1	2.22±0.02	2.24±0.02	0.94±0.01	0.69±0.01	1.56±0.11	504.4	460.7	4.20±0.04
Lake 238 09/07/2021	B	0.138±0.003	7.2	5.75±0.05	14.7±0.1	1.93±0.11	1.94±0.11	0.84±0.05	0.88±0.05	0.94±0.08	-	-	3.21±0.18
	B	0.159±0.006	7.2	5.63±0.05	14.5±0.1	1.95±0.04	2.00±0.04	0.85±0.02	0.86±0.02	1.04±0.09	-	-	3.25±0.07
	B	0.195±0.009	7.0	5.26±0.04	13.9±0.1	2.01±0.09	2.02±0.09	0.86±0.04	0.83±0.04	1.10±0.01	-	-	3.38±0.15
	B	0.242±0.009	7.1	4.71±0.03	12.9±0.1	2.10±0.10	2.10±0.10	0.89±0.04	0.82±0.04	1.17±0.05	-	-	3.62±0.17
	B	0.283±0.014	7.1	4.26±0.03	12.1±0.1	2.16±0.12	2.12±0.12	0.89±0.05	0.80±0.04	1.18±0.12	-	-	3.86±0.21
	B	0.321±0.015	7.2	3.95±0.03	11.4±0.1	2.18±0.11	2.18±0.11	0.91±0.05	0.77±0.04	1.28±0.04	-	-	4.07±0.21
	B	0.338±0.017	7.0	3.76±0.02	11.0±0.1	2.18±0.06	2.18±0.06	0.92±0.02	0.75±0.02	1.31±0.04	-	-	4.21±0.11
	B	0.345±0.017	7.1	3.74±0.02	10.9±0.1	2.19±0.10	2.19±0.10	0.92±0.04	0.73±0.03	1.33±0.09	-	-	4.23±0.20
	B	0.349±0.018	7.1	3.68±0.02	10.8±0.1	2.19±0.13	2.22±0.14	0.93±0.06	0.70±0.04	1.34±0.05	581.3	522.0	4.28±0.26
Lake 147 08/30/2021	C	0.078±0.009	7.1	5.35±0.09	13.1±0.1	1.93±0.10	1.94±0.10	0.80±0.04	0.95±0.05	1.24±0.10	-	-	3.64±0.18
	C	0.150±0.011	7.0	5.18±0.08	12.8±0.1	1.96±0.05	1.97±0.05	0.82±0.02	0.93±0.02	1.29±0.08	-	-	3.71±0.10
	C	0.208±0.016	7.3	4.89±0.08	12.3±0.1	2.03±0.06	1.99±0.06	0.83±0.02	0.90±0.03	1.41±0.10	-	-	3.83±0.11
	C	0.264±0.018	7.0	4.50±0.07	11.6±0.1	2.10±0.08	2.07±0.07	0.86±0.03	0.89±0.03	1.48±0.02	-	-	4.02±0.14
	C	0.310±0.020	7.2	4.21±0.06	11.0±0.1	2.16±0.08	2.09±0.08	0.87±0.03	0.86±0.03	1.51±0.07	-	-	4.19±0.15
	C	0.340±0.019	7.1	4.03±0.06	10.7±0.1	2.19±0.17	2.15±0.17	0.89±0.07	0.84±0.06	1.60±0.02	-	-	4.30±0.33
	C	0.354±0.019	7.2	3.95±0.05	10.5±0.1	2.20±0.08	2.15±0.07	0.90±0.03	0.81±0.03	1.61±0.02	-	-	4.35±0.15
	C	0.366±0.019	7.2	3.94±0.05	10.4±0.1	2.21±0.12	2.16±0.12	0.91±0.05	0.78±0.04	1.65±0.01	-	-	4.36±0.23
	C	0.370±0.019	7.2	3.92±0.05	10.4±0.1	2.21±0.10	2.19±0.10	0.92±0.04	0.76±0.03	1.66±0.06	602.2	573.2	4.38±0.19
Lake 138 09/07/2021	D	0.107±0.011	7.0	5.55±0.09	13.3±0.1	1.90±0.07	1.87±0.07	0.80±0.03	0.92±0.03	1.17±0.03	-	-	3.71±0.13
	D	0.202±0.024	7.2	5.37±0.09	13.0±0.1	1.93±0.07	1.90±0.07	0.81±0.03	0.90±0.03	1.16±0.08	-	-	3.78±0.14
	D	0.280±0.029	7.4	5.04±0.08	12.5±0.1	1.98±0.04	1.92±0.04	0.82±0.02	0.87±0.02	1.22±0.03	-	-	3.93±0.09
	D	0.326±0.028	7.1	4.69±0.07	11.9±0.1	2.04±0.14	2.00±0.14	0.84±0.06	0.85±0.06	1.28±0.03	-	-	4.11±0.28
	D	0.365±0.023	7.2	4.41±0.06	11.3±0.1	2.07±0.04	2.02±0.04	0.85±0.02	0.83±0.02	1.32±0.07	-	-	4.27±0.08
	D	0.389±0.025	7.0	4.31±0.06	11.1±0.1	2.08±0.08	2.04±0.08	0.88±0.04	0.80±0.03	1.40±0.01	-	-	4.34±0.17
	D	0.402±0.024	7.1	4.25±0.06	11.0±0.1	2.09±0.11	2.06±0.11	0.88±0.05	0.78±0.04	1.41±0.03	-	-	4.38±0.24
	D	0.412±0.026	7.2	4.23±0.06	11.0±0.1	2.09±0.14	2.08±0.14	0.90±0.06	0.76±0.05	1.43±0.04	-	-	4.39±0.30
	D	0.417±0.026	7.2	4.22±0.06	11.0±0.1	2.09±0.12	2.12±0.12	0.91±0.05	0.74±0.04	1.43±0.04	736.3	716.1	4.40±0.25

Table S6. Physicochemical and optical properties of bloom supernatants (continued)

Sample ID [Lake ID Sampling Date]	Bloom ID	OD_{680} (A.U.)	pH	*E2:E3*	$S_{290–400}$ (μm^{-1})	$SUVA_{254}$ (L mg C^{-1}•m^{-1})	FI	HIX	$\beta:\alpha$	Peak M:T	Chl-*a* (µg/L)	Chl-a_{cyano} (µg/L)	DOC (mg C/L)
Lake 33 09/04/2021	E	0.368±0.018	7.2	3.96±0.06	13.1±0.1	2.24±0.04	1.95±0.03	0.69±0.01	0.96±0.02	1.56±0.08	410.7	360.1	4.22±0.07
Lake 37 08/20/2021	F	0.267±0.013	7.6	4.12±0.07	11.6±0.1	2.08±0.07	1.88±0.06	0.85±0.03	0.87±0.03	1.24±0.05	750.4	610.6	3.69±0.12
Lake 38 08/29/2021	G	0.430±0.022	7.5	3.93±0.06	9.4±0.1	2.24±0.05	1.92±0.04	0.75±0.02	0.93±0.02	1.40±0.01	866.6	776.7	5.38±0.12
Lake 40 09/16/2021	H	0.392±0.020	7.3	3.89±0.06	11.1±0.1	2.23±0.05	1.88±0.04	0.83±0.02	0.86±0.02	1.51±0.03	518.2	501.2	5.13±0.10
Lake 78 08/22/2021	I	0.410±0.020	7.4	4.16±0.07	11.7±0.1	2.34±0.02	1.92±0.02	0.80±0.01	0.89±0.01	1.53±0.01	682.2	640.2	5.24±0.06
Lake 82 08/17/2021	J	0.475±0.024	7.9	4.01±0.07	10.6±0.1	2.27±0.04	1.94±0.03	0.70±0.01	0.93±0.02	1.33±0.11	1204.4	1188.4	5.72±0.10
Lake 93 08/29/2021	K	0.245±0.012	7.2	4.06±0.07	13.3±0.1	2.14±0.17	1.90±0.15	0.82±0.06	0.90±0.07	1.40±0.05	182.3	152.9	3.86±0.30
Lake 221 09/06/2021	L	0.542±0.027	7.5	3.78±0.06	10.1±0.1	2.45±0.21	1.94±0.17	0.74±0.06	0.93±0.08	1.32±0.01	2270.3	2258.5	5.90±0.51
Lake 256 10/12/2021	Otisco	0.008±0.001	7.1	7.01±0.11	17.5±0.1	1.78±0.13	1.96±0.14	0.91±0.07	0.68±0.05	3.39±0.17	-	-	2.95±0.21
	Otisco	0.013±0.001	7.2	7.10±0.12	17.6±0.1	1.77±0.12	1.95±0.13	0.91±0.06	0.72±0.05	3.31±0.13	-	-	2.93±0.19
	Otisco	0.016±0.001	7.2	7.30±0.12	17.8±0.1	1.74±0.09	1.88±0.10	0.86±0.05	0.80±0.04	3.28±0.04	-	-	2.89±0.15
	Otisco	0.018±0.002	7.1	7.50±0.13	18.0±0.1	1.72±0.07	1.88±0.07	0.84±0.03	0.89±0.03	3.22±0.05	-	-	2.84±0.11
	Otisco	0.020±0.002	7.0	7.75±0.13	18.3±0.1	1.69±0.12	1.86±0.13	0.82±0.06	0.89±0.06	3.22±0.12	-	-	2.79±0.19
	Otisco	0.022±0.002	7.2	7.91±0.14	18.5±0.2	1.67±0.08	1.86±0.08	0.82±0.04	0.90±0.04	3.19±0.12	-	-	2.76±0.13
	Otisco	0.023±0.002	7.1	8.01±0.14	18.6±0.2	1.65±0.07	1.84±0.08	0.81±0.03	0.92±0.04	3.15±0.06	-	-	2.74±0.11
	Otisco	0.024±0.002	7.1	8.11±0.14	18.7±0.2	1.64±0.01	1.77±0.01	0.80±0.01	0.95±0.01	3.14±0.05	-	-	2.72±0.02
	Otisco	0.025±0.002	7.2	8.19±0.15	18.8±0.2	1.63±0.13	1.75±0.14	0.79±0.06	0.96±0.08	3.12±0.07	4.5	<1.0	2.71±0.22
Lake 256 10/12/2021	Otisco	0.009±0.001	7.1	6.93±0.11	17.4±0.1	1.75±0.10	1.96±0.12	0.90±0.05	0.67±0.04	3.38±0.06			3.08±0.18
	Otisco	0.014±0.001	7.2	7.02±0.11	17.5±0.1	1.74±0.13	1.95±0.14	0.90±0.07	0.73±0.05	3.34±0.03	-	-	3.06±0.22
	Otisco	0.017±0.001	7.1	7.21±0.12	17.7±0.1	1.71±0.13	1.88±0.14	0.85±0.07	0.82±0.06	3.32±0.23	-	-	3.01±0.23
	Otisco	0.019±0.001	7.2	7.43±0.13	18.0±0.1	1.69±0.10	1.88±0.11	0.84±0.05	0.85±0.05	3.30±0.29	-	-	2.96±0.17
	Otisco	0.021±0.002	7.0	7.65±0.13	18.2±0.1	1.66±0.05	1.86±0.06	0.81±0.02	0.86±0.03	3.21±0.28	-	-	2.91±0.09
	Otisco	0.023±0.002	7.1	7.85±0.14	18.4±0.2	1.64±0.03	1.86±0.03	0.80±0.01	0.87±0.02	3.20±0.34	-	-	2.86±0.05
	Otisco	0.024±0.002	7.2	8.01±0.14	18.6±0.2	1.61±0.02	1.84±0.02	0.80±0.01	0.89±0.01	3.06±0.23	-	-	2.84±0.03
	Otisco	0.025±0.002	7.2	8.15±0.14	18.8±0.2	1.60±0.07	1.77±0.08	0.79±0.03	0.91±0.04	3.05±0.12	-	-	2.82±0.12
	Otisco	0.026±0.002	7.1	8.22±0.15	18.8±0.2	1.59±0.02	1.75±0.02	0.78±0.01	0.94±0.01	3.09±0.18	4.1	<1.0	2.80±0.04

OD_{680} = optical density at 680 nm; *E2:E3* = the ratio of absorption coefficients at 250 and 365 nm; $S_{290–400}$ = the spectral slope coefficient from 290 to 400 nm; $SUVA_{254}$ = specific UV absorbance at 254 nm; FI = fluorescence index; HIX = humification index; $\beta:\alpha$ = freshness index; Peak M:T = the ratio of microbial humic-like to protein-like DOM fluorescence.

5. Analytical methods for photochemistry experiments

Over the course of irradiation, 500 µL of sample aliquots were withdrawn from quartz test tubes at predetermined time intervals and analyzed for the concentrations of hTPA, FFA, TMP, four sorbic alcohol isomers (i.e., *cis*,*cis*-2,4-hexadien-1-ol, *cis*,*trans*-2,4-hexadien-1-ol, *trans*,*cis*-2,4-hexadien-1-ol, *trans*,*trans*-2,4-hexadien-1-ol), or PNA by an Agilent 1260 Infinity II high-performance liquid chromatograph with a variable wavelength detector and a fluorescence detector with instrument configurations and settings detailed in **Table S7**. The formation of hTPA from TPA,[89, 90] the loss of FFA,[26, 27] the loss of TMP,[28, 29] and the isomerization of *t,t*-HDO[30] were monitored as a function of time following the methods established in our previous work.[7]

Table S7. High-performance liquid chromatography methods

Analyte	Mobile Phase	Analytical Column	Detector	Retention Time
PNA	25% Water<br>75% MeOH<br>Isocratic flow rate: 0.5 mL/min	Agilent Poroshell 120 EC-C18<br>4.6 × 100 mm, 2.7 µm<br>Column temperature: 30 °C	VWD absorbance<br>λ_{UV} = 316 nm	3.2 min
hTPA	75% 10 mM Phosphate buffer<br>25% MeOH<br>Isocratic flow rate: 0.5 mL/min	Agilent Poroshell 120 EC-C18<br>4.6 × 100 mm, 2.7 µm<br>Column temperature: 30 °C	FLD fluorescence<br>$\lambda_{excitation}$ = 250 nm<br>$\lambda_{emission}$ = 410 nm	3.6 min
FFA	80% 10 mM Phosphate buffer<br>20% MeOH<br>Isocratic flow rate: 0.5 mL/min	Agilent Poroshell 120 EC-C18<br>4.6 × 100 mm, 2.7 µm<br>Column temperature: 30 °C	VWD absorbance<br>λ_{UV} = 219 nm	4.2 min
TMP	30% 10 mM Phosphate buffer<br>70% MeOH<br>Isocratic flow rate: 0.5 mL/min	Agilent Poroshell 120 EC-C18<br>4.6 × 100 mm, 2.7 µm<br>Column temperature: 30 °C	FLD fluorescence<br>$\lambda_{excitation}$ = 230 nm<br>$\lambda_{emission}$ = 325 nm	4.6 min
t,t-HDO	70% Water with 0.05% TFA<br>30% ACN<br>Isocratic flow rate: 0.7 mL/min	Phenomenex Luna C18<br>4.6 × 250 mm, 5 µm<br>Column temperature: 10 °C	VWD absorbance<br>λ_{UV} = 230 nm	*c,c*-HDO 13.6 min<br>*c,t*-HDO 14.1 min<br>*t,c*-HDO 14.6 min<br>*t,t*-HDO 15.3 min

VWD = variable wavelength detection; FLD = fluorescence detection; *c,c*-HDO = *cis,cis*-2,4-hexadien-1-ol; *c,t*-HDO = *cis,trans*-2,4-hexadien-1-ol; *t,c*-HDO = *trans,cis*-2,4-hexadien-1-ol; *t,t*-HDO = *trans,trans*-2,4-hexadien-1-ol. The limit of detection for PNA, hTPA, FFA, TMP, and *t,t*-HDO was 0.04 µM, 0.5 nM, 0.06 µM, 0.01 µM, and 0.02 µM, respectively.

6. *p*-Nitroanisole/pyridine actinometry

For each sample, the rate of light absorption R_a (mol-photons L^{-1} s^{-1} or Einstein L^{-1} s^{-1}) per unit sample volume was calculated as described in our previous work.[7] For each set of photochemistry experiments, the loss of PNA in 10 μM PNA/5 mM pyr actinometer solutions was monitored in the solar simulator to determine the pseudo-first order rate constant for the loss of PNA, $k_{obs,PNA}$ (s^{-1}):[2, 31, 32]

$$R_{loss,\,PNA} = -\frac{d[PNA]}{dt} = k_{obs,\,PNA}[PNA] = \Phi_{PNA}\sum_{\lambda\,=\,290\text{ nm}}^{550\text{ nm}} \frac{W_\lambda \varepsilon_\lambda [1-10^{-\alpha_{D(\lambda)}z}]}{z\alpha_\lambda}[PNA]$$
$$\approx \Phi_{PNA}\sum_{\lambda\,=\,290\text{ nm}}^{550\text{ nm}} I_\lambda \frac{\varepsilon_\lambda[1-10^{-\alpha_\lambda z}]}{\alpha_\lambda}[PNA] \qquad \text{(S1)}$$

where $R_{loss,\,PNA}$ (M s^{-1}) is the loss rate of PNA, [PNA] is the concentration of PNA, Φ_{PNA} (1.74×10^{-3} mol mol-photons^{-1} or mol Einstein^{-1}; calculated from Φ_{PNA} = 0.29 [pyr] + 0.00029) is the quantum yield for the loss of PNA at a given pyridine concentration (i.e., [pyr] = 5×10^{-3} M),[3] W_λ (10^{-3} mol-photons cm^{-2} s^{-1} nm^{-1} or milliEinstein cm^{-2} s^{-1} nm^{-1}) is the incident light intensity at a given wavelength λ, ε_λ (M^{-1} cm^{-1}) is the decadic molar absorption coefficient of PNA at a given wavelength λ,[3] $\alpha_{D(\lambda)}$ (cm^{-1}) is the apparent (or diffuse) attenuation coefficient ($\alpha_{D(\lambda)}\approx\alpha_\lambda$ where the distribution function D(λ) is ~1.0 for the quartz test tube[32]), z (1.12 cm) is the optical pathlength for the quartz test tube,[31] α_λ (cm^{-1}) is the decadic absorption (or attenuation) coefficient (i.e., the absorbance divided by the optical pathlength of the quartz cuvette), I_λ (10^{-3} mol-photons cm^{-3} s^{-1} nm^{-1} or milliEinstein cm^{-3} s^{-1} nm^{-1} or mol-photons L^{-1} s^{-1} nm^{-1}) is the incident light intensity at a given wavelength λ per unit volume, and 290-550 nm is the integration range chosen based on the wavelength dependence of $\Phi_{app,RI}$.[33, 34]

Equation S1 was re-written given that I_λ can be approximated by multiplying the fractional spectral intensity of the xenon arc lamp, ρ_λ (nm^{-1}), with the total incident light intensity from 290 to 550 nm per unit volume, I_0 (mol-photons L^{-1} s^{-1}; the wavelength range was selected to account for PNA absorbance past 400 nm[35]):[3, 36, 37]

$$k_{obs,\,PNA} = 2.303z\Phi_{PNA}\sum_{\lambda\,=\,290\text{ nm}}^{550\text{ nm}} \rho_\lambda I_0 \varepsilon_\lambda = 2.303z\Phi_{PNA}I_0\sum_{\lambda\,=\,290\text{ nm}}^{550\text{ nm}} \rho_\lambda \varepsilon_\lambda \qquad \text{(S2)}$$

Equation S2 was further re-arranged to calculate I_0, assuming that I_0 measured by the PNA/pyr actinometer represented I_0 through a given sample:[3, 36, 37]

$$I_0 = \frac{k_{\text{obs, PNA}}}{2.303 z \Phi_{\text{PNA}} \sum_{\lambda\,=\,290\text{ nm}}^{550\text{ nm}} \rho_\lambda \varepsilon_\lambda} \tag{S3}$$

Equation S3 was used to calculate R_a using the sample-specific α_λ (m^{-1}):[36, 37]

$$R_a = \sum_{\lambda\,=\,290\text{ nm}}^{550\text{ nm}} \frac{W_\lambda (1 - 10^{-\alpha_{\text{D},\lambda} z})}{z} \approx \sum_{\lambda\,=\,290\text{ nm}}^{550\text{ nm}} \rho_\lambda I_0 \, (1 - 10^{-\alpha_\lambda z}) \tag{S4}$$

R_a for the calculations of $\Phi_{app,RI}$ for whole water and bloom samples are summarized in **Tables S8-S10**.

**Table S8.** Summary of R_a for whole water samples			
Sample ID	R_a ($\times 10^{-6}$ mol-photons L^{-1} s^{-1})	Sample ID	R_a ($\times 10^{-6}$ mol-photons L^{-1} s^{-1})
Lake 2 06/08/2019	2.19±0.10	Lake 40 08/29/2018	1.69±0.08
Lake 2 08/31/2019	1.77±0.08	Lake 40 06/19/2019	0.52±0.02
Lake 6 06/18/2019	2.96±0.14	Lake 41 07/07/2018	0.55±0.03
Lake 6 08/28/2019	2.72±0.13	Lake 41 07/17/2018	0.57±0.03
Lake 8 08/05/2019	1.27±0.06	Lake 41 07/29/2018	0.52±0.02
Lake 12 06/08/2019	1.78±0.08	Lake 41 08/19/2018	0.49±0.02
Lake 12 09/01/2019	1.74±0.08	Lake 45 08/18/2019	0.96±0.04
Lake 13 07/08/2018	4.48±0.21	Lake 49 07/08/2019	0.64±0.03
Lake 13 07/22/2018	1.78±0.08	Lake 50 06/10/2019	1.18±0.05
Lake 13 08/06/2018	0.84±0.04	Lake 52 06/30/2019	0.46±0.02
Lake 13 08/21/2018	1.79±0.08	Lake 53 06/09/2019	0.95±0.04
Lake 13 09/02/2018	0.60±0.03	Lake 57 06/29/2019	0.69±0.03
Lake 13 06/10/2019	2.80±0.13	Lake 57 09/08/2019	0.68±0.03
Lake 13 08/31/2019	2.31±0.11	Lake 58 09/10/2019	7.67±0.35
Lake 17 07/01/2018	0.70±0.03	Lake 61 07/09/2018	0.68±0.03
Lake 17 07/15/2018	1.51±0.07	Lake 61 07/19/2018	1.66±0.08
Lake 17 07/28/2018	1.60±0.07	Lake 61 08/19/2018	0.74±0.03
Lake 17 08/12/2018	0.86±0.04	Lake 61 09/02/2018	0.55±0.03
Lake 17 08/26/2018	0.32±0.01	Lake 61 09/22/2018	1.02±0.05
Lake 17 07/15/2019	0.94±0.04	Lake 61 07/21/2019	0.60±0.03
Lake 17 08/12/2019	1.02±0.05	Lake 61 07/23/2019	0.62±0.03
Lake 18 08/05/2018	0.44±0.02	Lake 66 08/16/2019	1.37±0.06
Lake 18 08/18/2018	0.60±0.03	Lake 68 09/17/2019	1.09±0.05
Lake 18 09/09/2018	0.98±0.05	Lake 69 06/09/2019	1.66±0.08
Lake 18 09/30/2018	0.87±0.04	Lake 69 08/19/2019	2.89±0.13
Lake 18 06/15/2019	0.74±0.03	Lake 72 07/12/2019	5.90±0.27
Lake 18 08/12/2019	0.73±0.03	Lake 73 07/20/2019	1.29±0.06
Lake 21 07/02/2019	0.64±0.03	Lake 73 08/24/2019	1.38±0.06
Lake 21 08/13/2019	0.61±0.03	Lake 74 06/08/2019	1.44±0.07
Lake 22 06/23/2019	0.93±0.04	Lake 74 08/18/2019	1.08±0.05
Lake 22 06/23/2019	0.96±0.04	Lake 75 06/02/2019	0.52±0.02
Lake 22 09/16/2019	0.79±0.04	Lake 77 06/16/2019	4.19±0.19
Lake 22 09/16/2019	0.82±0.04	Lake 77 06/16/2019	1.94±0.09
Lake 23 06/12/2019	1.65±0.08	Lake 77 09/22/2019	2.67±0.12
Lake 23 09/17/2019	1.62±0.07	Lake 77 09/22/2019	1.74±0.08
Lake 25 07/08/2018	1.19±0.05	Lake 78 06/02/2019	1.28±0.06
Lake 25 07/29/2018	0.86±0.04	Lake 88 06/24/2019	0.97±0.04
Lake 25 06/09/2019	0.76±0.04	Lake 88 08/21/2019	0.76±0.04
Lake 29 06/02/2019	0.51±0.02	Lake 89 07/10/2019	1.03±0.05
Lake 29 08/12/2019	0.69±0.03	Lake 90 07/01/2018	1.16±0.05
Lake 31 06/15/2019	2.97±0.14	Lake 90 08/12/2018	2.53±0.12
Lake 31 08/27/2019	2.59±0.12	Lake 90 09/11/2018	3.50±0.16
Lake 33 08/18/2019	0.85±0.04	Lake 90 06/08/2019	2.57±0.12
Lake 33 09/01/2019	1.29±0.06	Lake 90 08/17/2019	2.20±0.10
Lake 34 06/04/2019	2.38±0.11	Lake 92 08/06/2018	0.56±0.03
Lake 34 06/04/2019	3.00±0.14	Lake 92 08/19/2018	0.54±0.02
Lake 37 06/23/2019	0.56±0.03	Lake 92 09/03/2018	0.63±0.03
Lake 37 08/19/2019	0.90±0.04	Lake 92 06/04/2019	0.40±0.02
Lake 38 06/17/2019	0.68±0.03	Lake 92 06/17/2019	0.42±0.02
Lake 39 07/20/2019	0.56±0.03	Lake 92 08/11/2019	0.39±0.02
Lake 40 06/27/2018	1.47±0.07	Lake 92 09/01/2019	0.58±0.03
Lake 40 07/14/2018	0.50±0.02	Lake 96 08/05/2019	0.93±0.04
Lake 40 08/01/2018	0.65±0.03	Lake 99 08/10/2019	3.68±0.17
Lake 40 08/12/2018	0.44±0.02	Lake 100 06/23/2019	1.99±0.09

**Table S8.** Summary of R_a for whole water samples (continued)			
Sample ID	R_a ($\times10^{-6}$ mol-photons L^{-1} s^{-1})	Sample ID	R_a ($\times10^{-6}$ mol-photons L^{-1} s^{-1})
Lake 100 08/18/2019	1.68±0.08	Lake 182 06/18/2019	0.63±0.03
Lake 102 06/16/2019	1.23±0.06	Lake 182 07/01/2019	0.92±0.04
Lake 102 09/22/2019	2.31±0.11	Lake 182 07/08/2019	0.92±0.04
Lake 103 06/09/2019	4.40±0.20	Lake 182 08/12/2019	0.69±0.03
Lake 107 08/15/2019	0.88±0.04	Lake 182 08/12/2019	0.56±0.03
Lake 108 08/17/2019	0.70±0.03	Lake 182 08/27/2019	0.62±0.03
Lake 109 07/08/2019	3.96±0.18	Lake 183 08/25/2019	1.09±0.05
Lake 109 08/18/2019	3.37±0.16	Lake 183 09/10/2019	1.13±0.05
Lake 115 07/08/2019	0.88±0.04	Lake 190 06/17/2019	2.45±0.11
Lake 115 07/08/2019	1.42±0.07	Lake 190 08/19/2019	2.35±0.11
Lake 115 09/09/2019	0.89±0.04	Lake 192 09/25/2019	2.84±0.13
Lake 115 09/09/2019	1.45±0.07	Lake 194 06/26/2018	1.65±0.08
Lake 117 08/22/2019	1.14±0.05	Lake 194 07/09/2018	1.84±0.08
Lake 117 09/05/2019	0.92±0.04	Lake 194 07/23/2018	0.78±0.04
Lake 120 06/10/2019	1.07±0.05	Lake 194 08/07/2018	0.93±0.04
Lake 120 08/19/2019	1.00±0.05	Lake 194 08/20/2018	1.03±0.05
Lake 126 07/08/2018	0.51±0.02	Lake 194 09/09/2018	0.98±0.05
Lake 126 07/21/2018	1.09±0.05	Lake 194 09/22/2019	1.05±0.05
Lake 126 08/17/2019	0.88±0.04	Lake 195 06/03/2019	4.69±0.22
Lake 130 08/25/2019	0.90±0.04	Lake 199 06/19/2018	0.42±0.02
Lake 130 09/22/2019	0.81±0.04	Lake 199 07/03/2018	1.13±0.05
Lake 132 06/10/2019	1.18±0.05	Lake 199 07/18/2018	0.17±0.01
Lake 132 09/03/2019	1.07±0.05	Lake 199 07/31/2018	0.28±0.01
Lake 133 06/04/2019	1.22±0.06	Lake 199 08/14/2018	0.61±0.03
Lake 133 08/19/2019	0.84±0.04	Lake 199 08/28/2018	0.49±0.02
Lake 135 05/27/2019	1.70±0.08	Lake 199 09/11/2018	0.57±0.03
Lake 135 08/31/2019	1.06±0.05	Lake 199 06/23/2019	0.44±0.02
Lake 136 06/08/2019	1.30±0.06	Lake 199 07/08/2019	0.64±0.03
Lake 136 09/20/2019	0.96±0.04	Lake 199 07/30/2019	0.26±0.01
Lake 137 08/18/2019	0.37±0.02	Lake 199 08/18/2019	0.26±0.01
Lake 139 08/06/2018	0.92±0.04	Lake 199 09/15/2019	0.23±0.01
Lake 139 09/19/2018	0.54±0.02	Lake 203 06/16/2019	1.80±0.08
Lake 145 06/06/2019	2.48±0.11	Lake 203 09/22/2019	1.26±0.06
Lake 145 08/15/2019	2.26±0.10	Lake 205 06/08/2019	2.07±0.10
Lake 149 06/10/2019	1.05±0.05	Lake 205 08/06/2019	2.01±0.09
Lake 149 09/21/2019	0.51±0.02	Lake 209 08/18/2019	1.29±0.06
Lake 153 08/25/2019	1.27±0.06	Lake 210 06/02/2019	2.42±0.11
Lake 153 09/08/2019	0.96±0.04	Lake 210 08/18/2019	3.43±0.16
Lake 164 06/23/2019	1.14±0.05	Lake 212 06/16/2018	0.41±0.02
Lake 164 08/27/2019	2.24±0.10	Lake 212 07/14/2018	1.28±0.06
Lake 166 07/14/2019	0.62±0.03	Lake 212 07/28/2018	1.31±0.06
Lake 166 09/29/2019	0.67±0.03	Lake 212 08/12/2018	1.07±0.05
Lake 169 07/22/2019	0.90±0.04	Lake 212 06/17/2019	2.41±0.11
Lake 169 08/18/2019	0.71±0.03	Lake 213 08/18/2019	1.17±0.05
Lake 176 07/15/2018	1.57±0.07	Lake 215 06/09/2019	2.26±0.10
Lake 176 06/15/2019	2.74±0.13	Lake 215 09/15/2019	2.64±0.12
Lake 176 08/19/2019	2.42±0.11	Lake 223 08/19/2019	1.94±0.09
Lake 177 08/19/2019	1.63±0.08	Lake 225 07/28/2019	2.16±0.10
Lake 178 08/06/2019	0.99±0.05	Lake 229 06/29/2019	0.99±0.05
Lake 182 07/17/2018	0.52±0.02	Lake 230 06/08/2019	1.58±0.07
Lake 182 07/31/2018	0.41±0.02	Lake 231 06/04/2019	1.80±0.08
Lake 182 08/14/2018	0.46±0.02	Lake 234 06/20/2018	0.69±0.03
Lake 182 09/11/2018	0.78±0.04	Lake 234 07/15/2018	0.65±0.03
Lake 182 06/18/2019	0.67±0.03	Lake 234 06/09/2019	1.10±0.05

**Table S8.** Summary of R_a for whole water samples (continued)			
Sample ID	R_a ($\times 10^{-6}$ mol-photons L^{-1} s^{-1})	Sample ID	R_a ($\times 10^{-6}$ mol-photons L^{-1} s^{-1})
Lake 234 08/12/2019	0.89±0.04	Lake 256 07/05/2018	0.50±0.02
Lake 235 07/14/2019	2.79±0.13	Lake 256 07/16/2018	0.51±0.02
Lake 236 07/14/2019	0.74±0.03	Lake 256 07/31/2018	0.55±0.03
Lake 236 09/15/2019	1.07±0.05	Lake 256 08/15/2018	0.63±0.03
Lake 238 08/12/2019	2.53±0.12	Lake 256 08/29/2018	1.36±0.06
Lake 239 06/09/2019	1.17±0.05	Lake 256 09/14/2018	0.74±0.03
Lake 239 08/18/2019	1.37±0.06	Lake 256 09/28/2018	0.63±0.03
Lake 245 09/22/2019	0.96±0.04	Lake 256 06/18/2019	0.99±0.05
Lake 246 08/05/2019	4.02±0.19	Lake 256 06/18/2019	0.56±0.03
Lake 247 06/08/2019	1.48±0.07	Lake 256 08/13/2019	0.64±0.03
Lake 247 09/20/2019	1.10±0.05	Lake 257 08/24/2019	6.10±0.28
Lake 248 08/18/2019	3.68±0.17	Lake 258 06/16/2019	1.62±0.07
Lake 249 07/08/2019	0.96±0.04	Lake 258 08/11/2019	2.56±0.12
Lake 249 09/01/2019	1.38±0.06	Lake 259 09/01/2019	3.63±0.17
Lake 250 07/08/2019	0.72±0.03	Lake 260 06/01/2019	2.20±0.10
Lake 250 08/18/2019	1.05±0.05	Lake 260 08/12/2019	2.11±0.10
Lake 251 08/27/2019	2.66±0.12	Lake 261 06/17/2019	1.71±0.08
Lake 251 09/17/2019	3.90±0.18	Lake 261 08/12/2019	1.63±0.08
Lake 253 07/02/2019	0.33±0.02	Lake 262 06/17/2019	4.74±0.22
Lake 253 08/25/2019	0.44±0.02	Lake 262 09/21/2019	3.26±0.15
Lake 253 09/15/2019	0.62±0.03		

**Table S9.** Summary of R_a for bloom lysates							
Sample ID	Bloom ID	OD_{680} (A.U.)	R_a ($\times10^{-6}$ mol-photons L^{-1} s^{-1})	Sample ID	Bloom ID	OD_{680} (A.U.)	R_a ($\times10^{-6}$ mol-photons L^{-1} s^{-1})
Lake 261 09/04/2021	A	0.379±0.009	1.53±0.03				
Lake 238 09/07/2021	B	0.349±0.018	1.52±0.11				
Lake 147 08/30/2021	C	0.370±0.019	1.61±0.27				
Lake 138 09/07/2021	D	0.417±0.026	1.23±0.01				
Lake 33 09/04/2021	E	0.368±0.018	1.55±0.08				
Lake 37 08/20/2021	F	0.267±0.013	1.07±0.08				
Lake 38 08/29/2021	G	0.430±0.022	1.63±0.02				
Lake 40 09/16/2021	H	0.392±0.020	1.44±0.05				
Lake 78 08/22/2021	I	0.410±0.020	1.21±0.01				
Lake 82 08/17/2021	J	0.475±0.024	1.03±0.09				
Lake 93 08/29/2021	K	0.245±0.012	0.91±0.02				
Lake 221 09/06/2021	L	0.542±0.027	1.57±0.03				
Lake 256 10/12/2021	Otisco	0.026±0.001	0.71±0.01	SRNOM	SRNOM	-	2.97±0.02
Lysate 25% + Otisco 75%	A	-	0.92±0.02	Lysate 25% + SRNOM 75%	A	-	2.27±0.05
Lysate 50% + Otisco 50%	A	-	1.13±0.04	Lysate 50% + SRNOM 50%	A	-	1.91±0.07
Lysate 75% + Otisco 25%	A	-	1.34±0.06	Lysate 75% + SRNOM 25%	A	-	2.50±0.03
Lysate 25% + Otisco 75%	B	-	0.80±0.02	Lysate 25% + SRNOM 75%	B	-	2.03±0.05
Lysate 50% + Otisco 50%	B	-	0.89±0.04	Lysate 50% + SRNOM 50%	B	-	1.56±0.06
Lysate 75% + Otisco 25%	B	-	0.98±0.06	Lysate 75% + SRNOM 25%	B	-	2.62±0.01
Lysate 25% + Otisco 75%	C	-	0.92±0.01	Lysate 25% + SRNOM 75%	C	-	2.26±0.01
Lysate 50% + Otisco 50%	C	-	1.12±0.02	Lysate 50% + SRNOM 50%	C	-	1.90±0.02
Lysate 75% + Otisco 25%	C	-	1.33±0.03	Lysate 75% + SRNOM 25%	C	-	2.61±0.04
Lysate 25% + Otisco 75%	D	-	0.91±0.03	Lysate 25% + SRNOM 75%	D	-	2.25±0.06
Lysate 50% + Otisco 50%	D	-	1.12±0.06	Lysate 50% + SRNOM 50%	D	-	1.89±0.09
Lysate 75% + Otisco 25%	D	-	1.32±0.08	Lysate 75% + SRNOM 25%	D	-	2.63±0.05
Lysate 25% + Otisco 75%	E	-	0.94±0.07	Lysate 25% + SRNOM 75%	E	-	2.30±0.12
Lysate 50% + Otisco 50%	E	-	1.16±0.13	Lysate 50% + SRNOM 50%	E	-	1.96±0.20
Lysate 75% + Otisco 25%	E	-	1.38±0.20	Lysate 75% + SRNOM 25%	E	-	2.54±0.02
Lysate 25% + Otisco 75%	F	-	0.84±0.01	Lysate 25% + SRNOM 75%	F	-	2.11±0.02
Lysate 50% + Otisco 50%	F	-	0.97±0.01	Lysate 50% + SRNOM 50%	F	-	1.67±0.01
Lysate 75% + Otisco 25%	F	-	1.10±0.01	Lysate 75% + SRNOM 25%	F	-	2.97±0.02

Table S10. Summary of R_a for bloom supernatants

Sample ID	Bloom ID	OD_{680} (A.U.)	R_a ($\times10^{-6}$ mol-photons L^{-1} s^{-1})	Sample ID	Bloom ID	OD_{680} (A.U.)	R_a ($\times10^{-6}$ mol-photons L^{-1} s^{-1})
Lake 261 09/04/2021	A	0.151±0.003	1.49±0.06	Lake 33 09/04/2021	E	0.368±0.018	2.82±0.16
	A	0.182±0.006	1.63±0.09	Lake 37 08/20/2021	F	0.267±0.013	3.09±0.18
	A	0.215±0.008	1.87±0.10	Lake 38 08/29/2021	G	0.430±0.022	5.50±0.32
	A	0.259±0.009	2.50±0.12	Lake 40 09/16/2021	H	0.392±0.020	4.95±0.29
	A	0.306±0.009	3.24±0.15	Lake 78 08/22/2021	I	0.410±0.020	4.81±0.28
	A	0.347±0.009	3.86±0.17	Lake 82 08/17/2021	J	0.475±0.024	5.32±0.30
	A	0.370±0.009	4.07±0.17	Lake 93 08/29/2021	K	0.245±0.012	2.39±0.13
	A	0.376±0.009	4.13±0.18	Lake 221 09/06/2021	L	0.542±0.027	6.33±0.36
	A	0.379±0.009	4.20±0.18				
Lake 238 09/07/2021	B	0.138±0.003	1.39±0.04	Lake 256 10/12/2021	Otisco	0.008±0.001	1.02±0.06
	B	0.159±0.006	1.47±0.04		Otisco	0.013±0.001	0.99±0.06
	B	0.195±0.009	1.71±0.05		Otisco	0.016±0.001	0.93±0.05
	B	0.242±0.009	2.20±0.06		Otisco	0.018±0.002	0.87±0.05
	B	0.283±0.014	2.74±0.06		Otisco	0.020±0.002	0.81±0.05
	B	0.321±0.015	3.22±0.07		Otisco	0.022±0.002	0.77±0.05
	B	0.338±0.017	3.56±0.08		Otisco	0.023±0.002	0.75±0.04
	B	0.345±0.017	3.61±0.08		Otisco	0.024±0.002	0.72±0.04
	B	0.349±0.018	3.73±0.08		Otisco	0.025±0.002	0.71±0.04
Lake 147 08/30/2021	C	0.078±0.009	1.76±0.10	Lake 256 10/12/2021	Otisco	0.009±0.001	1.06±0.06
	C	0.150±0.011	1.90±0.10		Otisco	0.014±0.001	1.03±0.06
	C	0.208±0.016	2.17±0.11		Otisco	0.017±0.001	0.97±0.06
	C	0.264±0.018	2.62±0.13		Otisco	0.019±0.001	0.90±0.05
	C	0.310±0.020	3.05±0.15		Otisco	0.021±0.002	0.84±0.05
	C	0.340±0.019	3.35±0.16		Otisco	0.023±0.002	0.79±0.05
	C	0.354±0.019	3.50±0.16		Otisco	0.024±0.002	0.75±0.05
	C	0.366±0.019	3.53±0.17		Otisco	0.025±0.002	0.73±0.04
	C	0.370±0.019	3.56±0.17		Otisco	0.026±0.002	0.71±0.04
Lake 138 09/07/2021	D	0.107±0.011	1.78±0.10				
	D	0.202±0.024	1.93±0.11				
	D	0.280±0.029	2.22±0.12				
	D	0.326±0.028	2.62±0.13				
	D	0.365±0.023	3.00±0.15				
	D	0.389±0.025	3.15±0.15				
	D	0.402±0.024	3.25±0.16				
	D	0.412±0.026	3.28±0.16				
	D	0.417±0.026	3.29±0.16				

7. Terephthalic acid (TPA) as a probe for $^{\bullet}$OH

TPA was spiked into samples to measure the photoproduction of $^{\bullet}$OH, although other lower-energy hydroxylating species[38-41] might also contribute to the observed TPA loss. For each sample, the *net* formation of TPA hydroxylation product, hTPA, was monitored by considering the production of hTPA from TPA and the concurrent loss of hTPA via direct photolysis and its negligible reactions with $^{\bullet}$OH and $^{1}O_2$:[42, 43]

$$R_{\mathrm{f,\,hTPA}} = \frac{\mathrm{d[hTPA]}}{\mathrm{dt}} = R_{\mathrm{prod,\,hTPA}} - R_{\mathrm{loss,\,hTPA}}$$

$$= k_{\mathrm{TPA},\,^{\bullet}\mathrm{OH}}[\mathrm{TPA}][^{\bullet}\mathrm{OH}]_{\mathrm{ss}} Y_{\mathrm{hTPA}} - k_{\mathrm{direct\ photolysis,\,hTPA}}[\mathrm{hTPA}] SF_{\Sigma\lambda} - k_{\mathrm{hTPA},\,^{\bullet}\mathrm{OH}}[\mathrm{hTPA}][^{\bullet}\mathrm{OH}]_{\mathrm{ss}} \quad \text{(S5)}$$

$$- k_{\mathrm{hTPA},\,^{1}\mathrm{O_2}}[\mathrm{hTPA}][^{1}\mathrm{O_2}]_{\mathrm{ss}}$$

$$\approx k_{\mathrm{TPA},\,^{\bullet}\mathrm{OH}}[\mathrm{TPA}][^{\bullet}\mathrm{OH}]_{\mathrm{ss}} Y_{\mathrm{hTPA}} - k_{\mathrm{direct\ photolysis,\,hTPA}}[\mathrm{hTPA}] SF_{\Sigma\lambda}$$

where $R_{\mathrm{f,\,hTPA}}$ (M s^{-1}) is the formation rate of hTPA, $R_{\mathrm{prod,\,hTPA}}$ (M s^{-1}) is the production rate of hTPA from TPA (note that the production rate of hTPA from TPA attributable to $^{\bullet}$OH formed via NO_3^- photolysis[44-46] or the photo-Fenton[47-49] pathway was minimal given the low concentrations of NO_x-N and Fe in samples), $R_{\mathrm{loss,\,hTPA}}$ (M s^{-1}) is the loss rate of hTPA, $k_{\mathrm{TPA},\,^{\bullet}\mathrm{OH}}$ (4.2±(0.3)×10^9 M^{-1} s^{-1}) is the second-order reaction rate constant of TPA with $^{\bullet}$OH,[42, 50] [TPA] is the initial concentration of TPA (10 μM), $[^{\bullet}\mathrm{OH}]_{\mathrm{ss}}$ is the steady-state concentration of $^{\bullet}$OH, Y_{hTPA} is the formation yield hTPA from the reaction of TPA with $^{\bullet}$OH, $k_{\mathrm{direct\ photolysis,\,hTPA}}$ (s^{-1}) is the experimentally determined direct photolysis rate constant of hTPA, [hTPA] is the concentration of hTPA, $SF_{\Sigma\lambda}$ is the sample-specific light screening factor,[51] $k_{\mathrm{hTPA},\,^{\bullet}\mathrm{OH}}$ (6.3±(0.1)×10^9 M^{-1} s^{-1}) is the second-order reaction rate constant of hTPA with $^{\bullet}$OH,[42] $k_{\mathrm{hTPA},\,^{1}\mathrm{O_2}}$ (5.0±(0.1)×10^4 M^{-1} s^{-1}) is the second-order reaction rate constant of hTPA with $^{1}O_2$,[42] and $[^{1}\mathrm{O_2}]_{\mathrm{ss}}$ is the steady-state concentration of $^{1}O_2$.

For each sample, a nonlinear least squares regression of hTPA data in the initial rate kinetics regime was performed using Equation S5 (i.e., $dx/dt = a + bx$ where dx/dt = d[hTPA]/dt and x = [hTPA]) to solve for the formation rate of $^{\bullet}$OH, $R_{\mathrm{f},\,^{\bullet}\mathrm{OH}}$ (M s^{-1}):[43, 52]

$$R_{\mathrm{f},\,^{\bullet}\mathrm{OH}} = \frac{R_{\mathrm{prod,\,hTPA}}}{Y_{\mathrm{hTPA}}} \quad \text{(S6)}$$

To account for the effects of pH and temperature on $R_{\text{f, hTPA}}$, a pH- and temperature-adjusted Y_{hTPA} of 0.37±0.01 was derived by averaging the values predicted from $Y_{\text{hTPA}} = [(30 + 0.43 \times \text{pH}) / 100]_{293\text{ K}} \times [(0.0059 \pm 0.0011) \times T - (1.50 \pm 0.31)] / [(0.0059 \pm 0.0011) \times 293\text{ K} - (1.50 \pm 0.31)]$ using T = 298 K (i.e., the chamber temperature of the solar simulator was controlled at 25±1 °C throughout the irradiation period) and an average sample pH of 7.3±0.4.[42, 50, 53-55]

The steady-state concentration of $^{\bullet}$OH in the presence of TPA, $[^{\bullet}\text{OH}]_{\text{ss}}$, was calculated as:[36, 43]

$$[^{\bullet}\text{OH}]_{\text{ss}} = \frac{R_{\text{f},\,^{\bullet}\text{OH}}}{k'_{\text{q},\,^{\bullet}\text{OH}}} = \frac{R^{\text{corr}}_{\text{prod, hTPA}}}{(k_{^{\bullet}\text{OH, HCO}_3^-}[\text{HCO}_3^-] + k_{^{\bullet}\text{OH, CO}_3^{2-}}[\text{CO}_3^{2-}] + k_{^{\bullet}\text{OH, DOM}}[\text{DOM}] + k_{\text{TPA},\,^{\bullet}\text{OH}}[\text{TPA}])Y_{\text{hTPA}}}$$
$$\approx \frac{R^{\text{corr}}_{\text{prod, hTPA}}}{(k_{^{\bullet}\text{OH, DOM}}[\text{DOM}] + k_{\text{TPA},\,^{\bullet}\text{OH}}[\text{TPA}])Y_{\text{hTPA}}} \quad \text{(S7)}$$

where $k'_{\text{q},\,^{\bullet}\text{OH}}$ (s^{-1}) is the pseudo-first order rate constant for $^{\bullet}$OH quenching, $k_{^{\bullet}\text{OH, HCO}_3^-}$ (8.5×10^6 M^{-1} s^{-1}) is the second-order reaction rate constant of $^{\bullet}$OH with HCO_3^-,[56] $[HCO_3^-]$ is the concentration of HCO_3^-, $k_{^{\bullet}\text{OH, CO}_3^{2-}}$ (3.9×10^8 M^{-1} s^{-1}) is the second-order reaction rate constant of $^{\bullet}$OH with CO_3^{2-},[56] $[CO_3^{2-}]$ is the concentration of CO_3^{2-}, $k_{^{\bullet}\text{OH, DOM}}$ (2.7(±1.5)×10^4 (mg C/L)$^{-1}$ s^{-1} or 3.3(±1.8)×10^8 (mol C/L)$^{-1}$ s^{-1} for whole water samples, 4.8(±1.6)×10^4 (mg C/L)$^{-1}$ s^{-1} or 5.8(±1.9)×10^8 (mol C/L)$^{-1}$ s^{-1} for bloom lysates, and 3.8(±1.9)×10^4 (mg C/L)$^{-1}$ s^{-1} or 4.6(±2.3)×10^8 (mol C/L)$^{-1}$ s^{-1} for bloom supernatants, respectively) is the average second-order reaction rate constant of $^{\bullet}$OH with DOM compiled from the literature,[42, 44, 46-48, 57-69] and [DOM] is the concentration of DOC.

The apparent quantum yield of $^{\bullet}$OH attributable to DOM, $\Phi_{\text{app},\,^{\bullet}\text{OH}}$ (mol mol-photons^{-1}), was calculated as:[36, 37]

$$\Phi_{\text{app},\,^{\bullet}\text{OH}} = \frac{R_{\text{f},\,^{\bullet}\text{OH}}}{R_{\text{a}}} = \frac{R^{\text{corr}}_{\text{prod, hTPA}}}{R_{\text{a}} Y_{\text{hTPA}}} \quad \text{(S8)}$$

$\Phi_{\text{app},\,^{\bullet}\text{OH}}$ for whole water and bloom samples **Tables S11-S14**.

Table S11. $\Phi_{app, \cdot OH}$ for whole water samples			
Sample ID	$\Phi_{app, \cdot OH}$ ($\times 10^{-5}$ mol mol-photons^{-1})	Sample ID	$\Phi_{app, \cdot OH}$ ($\times 10^{-5}$ mol mol-photons^{-1})
Lake 2 06/08/2019	1.41±0.03	Lake 40 08/29/2018	2.42±0.16
Lake 2 08/31/2019	1.85±0.10	Lake 40 06/19/2019	2.47±0.08
Lake 6 06/18/2019	1.85±0.10	Lake 41 07/07/2018	2.21±0.07
Lake 6 08/28/2019	2.33±0.11	Lake 41 07/17/2018	2.16±0.09
Lake 8 08/05/2019	1.50±0.08	Lake 41 07/29/2018	2.55±0.14
Lake 12 06/08/2019	1.36±0.04	Lake 41 08/19/2018	2.57±0.14
Lake 12 09/01/2019	2.09±0.06	Lake 45 08/18/2019	2.32±0.08
Lake 13 07/08/2018	1.99±0.11	Lake 49 07/08/2019	2.07±0.15
Lake 13 07/22/2018	1.65±0.08	Lake 50 06/10/2019	1.89±0.07
Lake 13 08/06/2018	1.92±0.08	Lake 52 06/30/2019	2.52±0.08
Lake 13 08/21/2018	1.96±0.12	Lake 53 06/09/2019	1.44±0.09
Lake 13 09/02/2018	2.20±0.11	Lake 57 06/29/2019	2.13±0.08
Lake 13 06/10/2019	1.23±0.04	Lake 57 09/08/2019	2.20±0.14
Lake 13 08/31/2019	1.54±0.07	Lake 58 09/10/2019	2.54±0.13
Lake 17 07/01/2018	1.83±0.04	Lake 61 07/09/2018	1.89±0.12
Lake 17 07/15/2018	1.43±0.11	Lake 61 07/19/2018	1.41±0.04
Lake 17 07/28/2018	1.87±0.10	Lake 61 08/19/2018	1.86±0.10
Lake 17 08/12/2018	2.17±0.11	Lake 61 09/02/2018	2.72±0.15
Lake 17 08/26/2018	1.96±0.16	Lake 61 09/22/2018	1.52±0.07
Lake 17 07/15/2019	1.98±0.10	Lake 61 07/21/2019	2.32±0.17
Lake 17 08/12/2019	2.00±0.10	Lake 61 07/23/2019	2.42±0.12
Lake 18 08/05/2018	1.74±0.07	Lake 66 08/16/2019	2.18±0.16
Lake 18 08/18/2018	2.35±0.10	Lake 68 09/17/2019	1.95±0.09
Lake 18 09/09/2018	1.86±0.09	Lake 69 06/09/2019	1.46±0.08
Lake 18 09/30/2018	2.04±0.09	Lake 69 08/19/2019	1.92±0.09
Lake 18 06/15/2019	1.90±0.11	Lake 72 07/12/2019	1.77±0.11
Lake 18 08/12/2019	1.66±0.07	Lake 73 07/20/2019	2.19±0.13
Lake 21 07/02/2019	1.92±0.16	Lake 73 08/24/2019	2.37±0.09
Lake 21 08/13/2019	1.91±0.08	Lake 74 06/08/2019	1.66±0.10
Lake 22 06/23/2019	1.88±0.08	Lake 74 08/18/2019	1.74±0.09
Lake 22 06/23/2019	1.57±0.10	Lake 75 06/02/2019	2.00±0.08
Lake 22 09/16/2019	1.88±0.12	Lake 77 06/16/2019	1.39±0.10
Lake 22 09/16/2019	1.84±0.10	Lake 77 06/16/2019	2.20±0.18
Lake 23 06/12/2019	1.60±0.11	Lake 77 09/22/2019	1.87±0.16
Lake 23 09/17/2019	2.14±0.14	Lake 77 09/22/2019	1.68±0.08
Lake 25 07/08/2018	2.28±0.12	Lake 78 06/02/2019	1.60±0.10
Lake 25 07/29/2018	1.94±0.07	Lake 88 06/24/2019	2.02±0.06
Lake 25 06/09/2019	1.94±0.07	Lake 88 08/21/2019	1.51±0.08
Lake 29 06/02/2019	1.80±0.09	Lake 89 07/10/2019	1.82±0.11
Lake 29 08/12/2019	1.46±0.06	Lake 90 07/01/2018	1.69±0.05
Lake 31 06/15/2019	1.73±0.07	Lake 90 08/12/2018	2.04±0.08
Lake 31 08/27/2019	2.03±0.13	Lake 90 09/11/2018	1.73±0.10
Lake 33 08/18/2019	2.05±0.15	Lake 90 06/08/2019	1.39±0.06
Lake 33 09/01/2019	2.28±0.13	Lake 90 08/17/2019	1.59±0.07
Lake 34 06/04/2019	1.43±0.04	Lake 92 08/06/2018	2.73±0.14
Lake 34 06/04/2019	1.19±0.05	Lake 92 08/19/2018	2.60±0.11
Lake 37 06/23/2019	2.45±0.09	Lake 92 09/03/2018	1.99±0.10
Lake 37 08/19/2019	2.16±0.07	Lake 92 06/04/2019	3.40±0.12
Lake 38 06/17/2019	1.77±0.08	Lake 92 06/17/2019	2.59±0.12
Lake 39 07/20/2019	2.26±0.21	Lake 92 08/11/2019	2.14±0.10
Lake 40 06/27/2018	1.73±0.08	Lake 92 09/01/2019	1.47±0.06
Lake 40 07/14/2018	3.14±0.12	Lake 96 08/05/2019	2.06±0.12
Lake 40 08/01/2018	2.11±0.15	Lake 99 08/10/2019	1.38±0.06
Lake 40 08/12/2018	2.53±0.16	Lake 100 06/23/2019	1.47±0.04

Table S11. $\Phi_{app, \cdot OH}$ for whole water samples (continued)			
Sample ID	$\Phi_{app, \cdot OH}$ ($\times 10^{-5}$ mol mol-photons^{-1})	Sample ID	$\Phi_{app, \cdot OH}$ ($\times 10^{-5}$ mol mol-photons^{-1})
Lake 100 08/18/2019	1.50±0.07	Lake 182 06/18/2019	2.20±0.14
Lake 102 06/16/2019	1.63±0.11	Lake 182 07/01/2019	1.73±0.13
Lake 102 09/22/2019	2.15±0.09	Lake 182 07/08/2019	1.61±0.08
Lake 103 06/09/2019	1.08±0.06	Lake 182 08/12/2019	1.66±0.03
Lake 107 08/15/2019	1.83±0.09	Lake 182 08/12/2019	2.03±0.12
Lake 108 08/17/2019	1.99±0.14	Lake 182 08/27/2019	1.97±0.10
Lake 109 07/08/2019	2.34±0.07	Lake 183 08/25/2019	1.50±0.07
Lake 109 08/18/2019	2.55±0.11	Lake 183 09/10/2019	1.85±0.08
Lake 115 07/08/2019	1.83±0.09	Lake 190 06/17/2019	2.43±0.14
Lake 115 07/08/2019	2.10±0.08	Lake 190 08/19/2019	2.42±0.13
Lake 115 09/09/2019	1.73±0.09	Lake 192 09/25/2019	1.82±0.13
Lake 115 09/09/2019	1.83±0.09	Lake 194 06/26/2018	1.95±0.08
Lake 117 08/22/2019	1.59±0.08	Lake 194 07/09/2018	1.66±0.08
Lake 117 09/05/2019	1.78±0.10	Lake 194 07/23/2018	1.55±0.08
Lake 120 06/10/2019	1.79±0.05	Lake 194 08/07/2018	1.59±0.10
Lake 120 08/19/2019	1.90±0.12	Lake 194 08/20/2018	1.87±0.06
Lake 126 07/08/2018	1.60±0.07	Lake 194 09/09/2018	1.84±0.08
Lake 126 07/21/2018	1.64±0.09	Lake 194 09/22/2019	2.28±0.14
Lake 126 08/17/2019	1.98±0.11	Lake 195 06/03/2019	1.11±0.08
Lake 130 08/25/2019	2.17±0.10	Lake 199 06/19/2018	2.11±0.06
Lake 130 09/22/2019	1.94±0.04	Lake 199 07/03/2018	1.12±0.06
Lake 132 06/10/2019	1.35±0.09	Lake 199 07/18/2018	3.42±0.17
Lake 132 09/03/2019	1.94±0.12	Lake 199 07/31/2018	2.27±0.11
Lake 133 06/04/2019	1.58±0.06	Lake 199 08/14/2018	1.18±0.06
Lake 133 08/19/2019	2.08±0.09	Lake 199 08/28/2018	1.55±0.06
Lake 135 05/27/2019	1.48±0.13	Lake 199 09/11/2018	2.52±0.16
Lake 135 08/31/2019	1.98±0.12	Lake 199 06/23/2019	2.74±0.18
Lake 136 06/08/2019	1.69±0.08	Lake 199 07/08/2019	1.30±0.05
Lake 136 09/20/2019	2.06±0.13	Lake 199 07/30/2019	2.30±0.08
Lake 137 08/18/2019	2.41±0.09	Lake 199 08/18/2019	2.21±0.08
Lake 139 08/06/2018	1.07±0.04	Lake 199 09/15/2019	3.41±0.13
Lake 139 09/19/2018	1.51±0.08	Lake 203 06/16/2019	1.39±0.07
Lake 145 06/06/2019	1.43±0.08	Lake 203 09/22/2019	1.70±0.08
Lake 145 08/15/2019	1.52±0.09	Lake 205 06/08/2019	1.49±0.05
Lake 149 06/10/2019	1.75±0.11	Lake 205 08/06/2019	1.66±0.07
Lake 149 09/21/2019	2.18±0.09	Lake 209 08/18/2019	1.41±0.07
Lake 153 08/25/2019	1.38±0.07	Lake 210 06/02/2019	1.16±0.07
Lake 153 09/08/2019	1.62±0.08	Lake 210 08/18/2019	1.11±0.07
Lake 164 06/23/2019	2.09±0.12	Lake 212 06/16/2018	2.00±0.14
Lake 164 08/27/2019	2.27±0.07	Lake 212 07/14/2018	1.65±0.08
Lake 166 07/14/2019	2.16±0.07	Lake 212 07/28/2018	1.66±0.10
Lake 166 09/29/2019	1.84±0.06	Lake 212 08/12/2018	1.75±0.12
Lake 169 07/22/2019	2.08±0.11	Lake 212 06/17/2019	1.51±0.05
Lake 169 08/18/2019	2.21±0.10	Lake 213 08/18/2019	2.23±0.14
Lake 176 07/15/2018	1.51±0.11	Lake 215 06/09/2019	1.64±0.09
Lake 176 06/15/2019	1.36±0.09	Lake 215 09/15/2019	1.95±0.08
Lake 176 08/19/2019	1.49±0.05	Lake 223 08/19/2019	1.71±0.06
Lake 177 08/19/2019	1.64±0.08	Lake 225 07/28/2019	1.43±0.05
Lake 178 08/06/2019	1.94±0.10	Lake 229 06/29/2019	1.58±0.10
Lake 182 07/17/2018	2.00±0.09	Lake 230 06/08/2019	2.35±0.09
Lake 182 07/31/2018	2.30±0.08	Lake 231 06/04/2019	1.56±0.10
Lake 182 08/14/2018	2.53±0.18	Lake 234 06/20/2018	2.40±0.13
Lake 182 09/11/2018	2.52±0.23	Lake 234 07/15/2018	2.51±0.16
Lake 182 06/18/2019	2.52±0.13	Lake 234 06/09/2019	1.46±0.08

Table S11. $\Phi_{app, \cdot OH}$ for whole water samples (continued)			
Sample ID	$\Phi_{app, \cdot OH}$ ($\times 10^{-5}$ mol mol-photons^{-1})	Sample ID	$\Phi_{app, \cdot OH}$ ($\times 10^{-5}$ mol mol-photons^{-1})
Lake 234 08/12/2019	1.46±0.08	Lake 256 07/05/2018	2.34±0.14
Lake 235 07/14/2019	1.45±0.06	Lake 256 07/16/2018	2.02±0.07
Lake 236 07/14/2019	2.11±0.10	Lake 256 07/31/2018	1.72±0.08
Lake 236 09/15/2019	2.42±0.09	Lake 256 08/15/2018	1.69±0.08
Lake 238 08/12/2019	2.12±0.10	Lake 256 08/29/2018	1.85±0.08
Lake 239 06/09/2019	2.29±0.09	Lake 256 09/14/2018	2.23±0.11
Lake 239 08/18/2019	1.97±0.10	Lake 256 09/28/2018	2.40±0.12
Lake 245 09/22/2019	1.74±0.08	Lake 256 06/18/2019	1.21±0.05
Lake 246 08/05/2019	2.13±0.08	Lake 256 06/18/2019	1.66±0.11
Lake 247 06/08/2019	1.82±0.11	Lake 256 08/13/2019	1.97±0.11
Lake 247 09/20/2019	2.17±0.09	Lake 257 08/24/2019	2.20±0.14
Lake 248 08/18/2019	1.64±0.11	Lake 258 06/16/2019	2.05±0.14
Lake 249 07/08/2019	1.78±0.14	Lake 258 08/11/2019	2.01±0.14
Lake 249 09/01/2019	1.54±0.09	Lake 259 09/01/2019	2.91±0.21
Lake 250 07/08/2019	2.24±0.10	Lake 260 06/01/2019	1.40±0.06
Lake 250 08/18/2019	1.52±0.06	Lake 260 08/12/2019	1.88±0.10
Lake 251 08/27/2019	2.06±0.10	Lake 261 06/17/2019	2.04±0.12
Lake 251 09/17/2019	2.05±0.16	Lake 261 08/12/2019	2.20±0.05
Lake 253 07/02/2019	2.10±0.11	Lake 262 06/17/2019	1.19±0.07
Lake 253 08/25/2019	2.83±0.20	Lake 262 09/21/2019	1.47±0.05
Lake 253 09/15/2019	2.00±0.14		

Table S12. $\Phi_{app, \cdot OH}$ for bloom lysates

Sample ID	Bloom ID	OD_{680} (A.U.)	$\Phi_{app, \cdot OH}$ ($\times 10^{-5}$ mol mol-photons^{-1})	Sample ID	Bloom ID	OD_{680} (A.U.)	$\Phi_{app, \cdot OH}$ ($\times 10^{-5}$ mol mol-photons^{-1})
Lake 261 09/04/2021	A	0.379±0.009	0.63±0.08				
Lake 238 09/07/2021	B	0.349±0.018	0.63±0.07				
Lake 147 08/30/2021	C	0.370±0.019	0.62±0.05				
Lake 138 09/07/2021	D	0.417±0.026	0.65±0.07				
Lake 33 09/04/2021	E	0.368±0.018	0.64±0.06				
Lake 37 08/20/2021	F	0.267±0.013	0.61±0.04				
Lake 38 08/29/2021	G	0.430±0.022	0.78±0.11				
Lake 40 09/16/2021	H	0.392±0.020	0.77±0.09				
Lake 78 08/22/2021	I	0.410±0.020	0.71±0.09				
Lake 82 08/17/2021	J	0.475±0.024	0.87±0.09				
Lake 93 08/29/2021	K	0.245±0.012	0.63±0.07				
Lake 221 09/06/2021	L	0.542±0.027	0.93±0.11				
Lake 256 10/12/2021	Otisco	0.026±0.001	1.39±0.08	SRNOM	-	-	2.15±0.13
Lysate 25% + Otisco 75%	A	-	0.98±0.06	Lysate 25% + SRNOM 75%	A	-	1.50±0.14
Lysate 50% + Otisco 50%	A	-	0.81±0.07	Lysate 50% + SRNOM 50%	A	-	1.17±0.09
Lysate 75% + Otisco 25%	A	-	0.69±0.06	Lysate 75% + SRNOM 25%	A	-	0.86±0.06
Lysate 25% + Otisco 75%	B	-	1.01±0.07	Lysate 25% + SRNOM 75%	B	-	1.50±0.13
Lysate 50% + Otisco 50%	B	-	0.83±0.07	Lysate 50% + SRNOM 50%	B	-	1.12±0.10
Lysate 75% + Otisco 25%	B	-	0.68±0.05	Lysate 75% + SRNOM 25%	B	-	0.83±0.03
Lysate 25% + Otisco 75%	C	-	0.98±0.08	Lysate 25% + SRNOM 75%	C	-	1.51±0.11
Lysate 50% + Otisco 50%	C	-	0.80±0.08	Lysate 50% + SRNOM 50%	C	-	1.19±0.12
Lysate 75% + Otisco 25%	C	-	0.68±0.08	Lysate 75% + SRNOM 25%	C	-	0.88±0.05
Lysate 25% + Otisco 75%	D	-	0.97±0.07	Lysate 25% + SRNOM 75%	D	-	1.50±0.13
Lysate 50% + Otisco 50%	D	-	0.79±0.08	Lysate 50% + SRNOM 50%	D	-	1.14±0.08
Lysate 75% + Otisco 25%	D	-	0.67±0.05	Lysate 75% + SRNOM 25%	D	-	0.83±0.05
Lysate 25% + Otisco 75%	E	-	0.98±0.09	Lysate 25% + SRNOM 75%	E	-	1.52±0.13
Lysate 50% + Otisco 50%	E	-	0.80±0.08	Lysate 50% + SRNOM 50%	E	-	1.18±0.08
Lysate 75% + Otisco 25%	E	-	0.67±0.04	Lysate 75% + SRNOM 25%	E	-	0.87±0.04
Lysate 25% + Otisco 75%	F	-	1.02±0.07	Lysate 25% + SRNOM 75%	F	-	1.54±0.16
Lysate 50% + Otisco 50%	F	-	0.84±0.05	Lysate 50% + SRNOM 50%	F	-	1.22±0.12
Lysate 75% + Otisco 25%	F	-	0.71±0.05	Lysate 75% + SRNOM 25%	F	-	0.93±0.09

Table S13. $\Phi_{app, \cdot OH}$ for bloom supernatants

Sample ID	Bloom ID	OD_{680} (A.U.)	$\Phi_{app, \cdot OH}$ ($\times 10^{-5}$ mol mol-photons^{-1})	Sample ID	Bloom ID	OD_{680} (A.U.)	$\Phi_{app, \cdot OH}$ ($\times 10^{-5}$ mol mol-photons^{-1})
Lake 261 09/04/2021	A	0.151±0.003	2.09±0.18	Lake 33 09/04/2021	E	0.368±0.018	3.16±0.17
	A	0.182±0.006	2.45±0.13	Lake 37 08/20/2021	F	0.267±0.013	2.74±0.23
	A	0.215±0.008	2.68±0.15	Lake 38 08/29/2021	G	0.430±0.022	3.52±0.25
	A	0.259±0.009	2.89±0.18	Lake 40 09/16/2021	H	0.392±0.020	3.90±0.31
	A	0.306±0.009	3.05±0.11	Lake 78 08/22/2021	I	0.410±0.020	3.60±0.23
	A	0.347±0.009	3.13±0.19	Lake 82 08/17/2021	J	0.475±0.024	4.19±0.29
	A	0.370±0.009	3.16±0.14	Lake 93 08/29/2021	K	0.245±0.012	2.84±0.23
	A	0.376±0.009	3.17±0.09	Lake 221 09/06/2021	L	0.542±0.027	4.88±0.27
	A	0.379±0.009	3.17±0.14				
Lake 238 09/07/2021	B	0.138±0.003	2.13±0.19	Lake 256 10/12/2021	Otisco	0.008±0.001	1.39±0.08
	B	0.159±0.006	2.35±0.14		Otisco	0.013±0.001	1.38±0.09
	B	0.195±0.009	2.55±0.14		Otisco	0.016±0.001	1.38±0.05
	B	0.242±0.009	2.73±0.13		Otisco	0.018±0.002	1.38±0.07
	B	0.283±0.014	2.88±0.11		Otisco	0.020±0.002	1.37±0.03
	B	0.321±0.015	3.02±0.07		Otisco	0.022±0.002	1.36±0.03
	B	0.338±0.017	3.05±0.13		Otisco	0.023±0.002	1.36±0.04
	B	0.345±0.017	3.07±0.10		Otisco	0.024±0.002	1.35±0.09
	B	0.349±0.018	3.07±0.13		Otisco	0.025±0.002	1.35±0.11
Lake 147 08/30/2021	C	0.078±0.009	2.15±0.13	Lake 256 10/12/2021	Otisco	0.009±0.001	1.39±0.08
	C	0.150±0.011	2.62±0.16		Otisco	0.014±0.001	1.39±0.08
	C	0.208±0.016	2.87±0.17		Otisco	0.017±0.001	1.38±0.11
	C	0.264±0.018	3.09±0.12		Otisco	0.019±0.001	1.38±0.11
	C	0.310±0.020	3.19±0.10		Otisco	0.021±0.002	1.37±0.04
	C	0.340±0.019	3.26±0.08		Otisco	0.023±0.002	1.37±0.04
	C	0.354±0.019	3.29±0.09		Otisco	0.024±0.002	1.36±0.10
	C	0.366±0.019	3.31±0.09		Otisco	0.025±0.002	1.36±0.05
	C	0.370±0.019	3.31±0.06		Otisco	0.026±0.002	1.35±0.11
Lake 138 09/07/2021	D	0.107±0.011	2.11±0.10				
	D	0.202±0.024	2.76±0.14				
	D	0.280±0.029	3.02±0.19				
	D	0.326±0.028	3.17±0.12				
	D	0.365±0.023	3.27±0.09				
	D	0.389±0.025	3.32±0.15				
	D	0.402±0.024	3.35±0.10				
	D	0.412±0.026	3.37±0.10				
	D	0.417±0.026	3.38±0.14				

Table S14. $\Phi_{app,\,^{\cdot}OH}$ for bloom supernatants minus the contribution from Otisco Lake water

Sample ID	Bloom ID	ΔOD_{680} (A.U.)	$\Delta\Phi_{app,\,^{\cdot}OH}$ ($\times10^{-5}$ mol mol-photons^{-1})	Sample ID	Bloom ID	ΔOD_{680} (A.U.)	$\Delta\Phi_{app,\,^{\cdot}OH}$ ($\times10^{-5}$ mol mol-photons^{-1})
Lake 261 09/04/2021	A	0.143±0.007	0.70±0.11	Lake 33 09/04/2021	E	0.343±0.017	1.82±0.06
	A	0.168±0.008	1.06±0.04	Lake 37 08/20/2021	F	0.242±0.012	1.39±0.12
	A	0.199±0.010	1.30±0.07	Lake 38 08/29/2021	G	0.405±0.020	2.17±0.14
	A	0.241±0.012	1.51±0.16	Lake 40 09/16/2021	H	0.367±0.018	2.56±0.20
	A	0.286±0.014	1.68±0.07	Lake 78 08/22/2021	I	0.384±0.019	2.25±0.12
	A	0.325±0.016	1.76±0.18	Lake 82 08/17/2021	J	0.450±0.022	2.84±0.18
	A	0.346±0.017	1.80±0.11	Lake 93 08/29/2021	K	0.219±0.011	1.49±0.12
	A	0.352±0.018	1.81±0.02	Lake 221 09/06/2021	L	0.517±0.026	3.53±0.16
	A	0.354±0.018	1.82±0.03				
Lake 238 09/07/2021	B	0.130±0.006	0.73±0.12				
	B	0.146±0.007	0.96±0.05				
	B	0.179±0.009	1.17±0.06				
	B	0.224±0.011	1.35±0.11				
	B	0.263±0.013	1.51±0.07				
	B	0.299±0.015	1.65±0.06				
	B	0.315±0.016	1.69±0.10				
	B	0.320±0.016	1.71±0.03				
	B	0.323±0.016	1.72±0.02				
Lake 147 08/30/2021	C	0.070±0.003	0.75±0.06				
	C	0.137±0.007	1.24±0.07				
	C	0.192±0.010	1.49±0.10				
	C	0.246±0.012	1.71±0.10				
	C	0.290±0.015	1.82±0.06				
	C	0.318±0.016	1.89±0.07				
	C	0.331±0.017	1.93±0.05				
	C	0.341±0.017	1.95±0.02				
	C	0.345±0.017	1.96±0.05				
Lake 138 09/07/2021	D	0.099±0.005	0.71±0.02				
	D	0.189±0.009	1.37±0.06				
	D	0.264±0.013	1.63±0.11				
	D	0.308±0.015	1.79±0.10				
	D	0.345±0.017	1.90±0.05				
	D	0.367±0.018	1.96±0.14				
	D	0.379±0.019	1.99±0.07				
	D	0.388±0.019	2.02±0.03				
	D	0.391±0.020	2.03±0.03				

8. Furfuryl alcohol (FFA) as a probe for $^1O_2$

FFA was spiked into samples to measure the photoproduction of $^1O_2$. For each sample, the loss of FFA was monitored to determine the pseudo-first order rate constant for the photodegradation of FFA, $k_{\text{obs, FFA}}$ (s^{-1}), with the contributions from *apparent* direct photolysis of FFA caused by impurities[26, 70] (7.9±3.9% for whole water samples, 11.5±5.9% for bloom lysates, and 4.6±4.6% for bloom supernatants, respectively) and the reaction of FFA with $^{\bullet}OH$ (19.2±3.1% for whole water samples, 13.2±2.6% for bloom lysates, and 12.3±2.0% for bloom supernatants, respectively):[71, 72]

$$R_{\text{loss, FFA}} = -\frac{\text{d[FFA]}}{\text{dt}} = k_{\text{obs, FFA}}[\text{FFA}]$$
$$= k_{\text{FFA, }^1\text{O}_2}[\text{FFA}][^1\text{O}_2]_{\text{ss}} + k_{\text{direct photolysis, FFA}}[\text{FFA}]SF_{\Sigma\lambda} + k_{\text{FFA, }^{\bullet}\text{OH}}[\text{FFA}][^{\bullet}\text{OH}]_{\text{ss}} \quad \text{(S9)}$$

where $R_{\text{loss, FFA}}$ ($M\ s^{-1}$) is the loss rate of FFA, [FFA] is the initial concentration of FFA (10 μM), $k_{\text{FFA, }^1\text{O}_2}$ ($M^{-1}\ s^{-1}$) is the second-order reaction rate constant of FFA with $^1O_2$, and $[^1O_2]_{ss}$ is the steady-state concentration of $^1O_2$, $k_{\text{direct photolysis, FFA}}$ (s^{-1}) is the experimentally determined *apparent* direct photolysis rate constant of FFA, $SF_{\Sigma\lambda}$ is the sample-specific light screening factor, $k_{\text{FFA, }^{\bullet}\text{OH}}$ ($1.5\times10^{10}\ M^{-1}\ s^{-1}$) is the second-order reaction rate constant of FFA with $^{\bullet}OH$,[56] and $[^{\bullet}OH]_{ss}$ is the steady-state concentration of $^{\bullet}OH$ measured by TPA.

To account for the potential effect of temperature on the $^1O_2$ reaction kinetics of FFA, a temperature-adjusted $k_{\text{FFA, }^1\text{O}_2}$ of $1.06(\pm0.07)\times10^8\ M^{-1}\ s^{-1}$ was derived by substituting T = 25 °C into $k_{\text{FFA, }^1\text{O}_2} = (1.00 \pm 0.04) \times 10^8\ M^{-1}\ s^{-1} + [(2.1 \pm 0.3) \times 10^6\ M^{-1}\ s^{-1}\ °C^{-1}] \times (T - 22\ °C)$.[27]

For each sample, the steady-state concentration of $^1O_2$, $[^1O_2]_{ss}$, was calculated as:[26, 27, 36, 73]

$$[^1\text{O}_2]_{\text{ss}} = \frac{R_{\text{f, }^1\text{O}_2}}{k_{\text{d}}^{\Delta}} = \frac{(k_{\text{obs, FFA}} - k_{\text{direct photolysis, FFA}}SF_{\Sigma\lambda} - k_{\text{FFA, }^{\bullet}\text{OH}}[^{\bullet}\text{OH}]_{\text{ss}})(k_{\text{d}}^{\Delta} + k_{\text{FFA, }^1\text{O}_2}[\text{FFA}])}{k_{\text{d}}^{\Delta}k_{\text{FFA, }^1\text{O}_2}}$$
$$\approx \frac{(k_{\text{obs, FFA}} - k_{\text{direct photolysis, FFA}}SF_{\Sigma\lambda} - k_{\text{FFA, }^{\bullet}\text{OH}}[^{\bullet}\text{OH}]_{\text{ss}})}{k_{\text{FFA, }^1\text{O}_2}} \quad \text{(S10)}$$

where $R_{\text{f, }^1\text{O}_2}$ ($M\ s^{-1}$) is the formation rate of $^1O_2$ and k_{d}^{Δ} ($2.78(\pm0.03)\times10^5\ s^{-1}$; temperature-adjusted for T = 25 °C[27]) is the pseudo-first order deactivation rate constant of $^1O_2$ by water.

The formation rate of $^1O_2$, $R_{f,\,^1O_2}$ (M s^{-1}), was calculated as:

$$R_{f,\,^1O_2} = [^1O_2]_{ss} k_d^{\Delta} \quad \text{(S11)}$$

The apparent quantum yield of $^1O_2$, $\Phi_{app,\,^1O_2}$ (mol mol-photons^{-1}), was calculated as:[36, 73]

$$\Phi_{app,\,^1O_2} = \frac{R_{f,\,^1O_2}}{R_a} = \frac{(k_d^{\Delta} + k_{FFA,\,^1O_2}[FFA])[^1O_2]_{ss}}{R_a} \approx \frac{k_d^{\Delta}[^1O_2]_{ss}}{R_a} \quad \text{(S12)}$$

To determine $\Phi_{app,\,^1O_2}$ attributable to high-energy $^3DOM^*$ (i.e., $\Phi_{app,\,^1O_2,\,high\text{-}energy}$) and low-energy $^3DOM^*$ (i.e., $\Phi_{app,\,^1O_2,\,low\text{-}energy}$) for the supernatants of recultivated bloom samples, *t,t*-HDO (2 mM) was spiked into FFA-containing (10 μM) supernatants to preferentially quench high-energy $^3DOM^*$ capable of sensitizing *t,t*-HDO isomerization (i.e., $^3DOM^*$ with $E_T \geq 250$ kJ mol^{-1} [74]). The percentage contribution of $\Phi_{app,\,^1O_2,\,high\text{-}energy}$ to $\Phi_{app,\,^1O_2}$ and the percentage contribution of $\Phi_{app,\,^1O_2,\,low\text{-}energy}$ to $\Phi_{app,\,^1O_2}$ were calculated as:[75]

$$\%\Phi_{app,\,^1O_2,\,high\text{-}energy} = \%\Phi_{app,\,^1O_2\_^3DOM^*_{HDO}} = \frac{(\Phi_{app,\,^1O_2} - \Phi_{app,\,^1O_2,\,HDO})}{\Phi_{app,\,^1O_2}} \times 100\% \quad \text{(S13)}$$

$$\%\Phi_{app,\,^1O_2,\,low\text{-}energy} = \frac{\Phi_{app,\,^1O_2,\,HDO}}{\Phi_{app,\,^1O_2}} \times 100\% \quad \text{(S14)}$$

$\Phi_{app,\,^1O_2}$ for whole water and bloom samples are summarized in **Tables S15-S18**.

**Table S15.** $\Phi_{app, {}^1O_2}$ for whole water samples			
Sample ID	$\Phi_{app, {}^1O_2}$ ($\times 10^{-2}$ mol mol-photons^{-1})	Sample ID	$\Phi_{app, {}^1O_2}$ ($\times 10^{-2}$ mol mol-photons^{-1})
Lake 2 06/08/2019	1.97±0.16	Lake 40 08/29/2018	3.04±0.22
Lake 2 08/31/2019	2.09±0.15	Lake 40 06/19/2019	2.39±0.19
Lake 6 06/18/2019	1.85±0.13	Lake 41 07/07/2018	3.10±0.25
Lake 6 08/28/2019	3.31±0.25	Lake 41 07/17/2018	2.46±0.19
Lake 8 08/05/2019	1.87±0.14	Lake 41 07/29/2018	3.07±0.22
Lake 12 06/08/2019	1.93±0.15	Lake 41 08/19/2018	3.14±0.23
Lake 12 09/01/2019	2.33±0.18	Lake 45 08/18/2019	2.12±0.17
Lake 13 07/08/2018	3.01±0.23	Lake 49 07/08/2019	2.25±0.15
Lake 13 07/22/2018	2.29±0.17	Lake 50 06/10/2019	2.05±0.16
Lake 13 08/06/2018	2.65±0.20	Lake 52 06/30/2019	2.96±0.24
Lake 13 08/21/2018	2.94±0.22	Lake 53 06/09/2019	1.77±0.13
Lake 13 09/02/2018	2.73±0.20	Lake 57 06/29/2019	2.15±0.16
Lake 13 06/10/2019	1.68±0.13	Lake 57 09/08/2019	2.15±0.15
Lake 13 08/31/2019	2.06±0.16	Lake 58 09/10/2019	3.51±0.27
Lake 17 07/01/2018	2.03±0.17	Lake 61 07/09/2018	2.01±0.14
Lake 17 07/15/2018	1.85±0.13	Lake 61 07/19/2018	2.26±0.18
Lake 17 07/28/2018	2.06±0.15	Lake 61 08/19/2018	2.00±0.15
Lake 17 08/12/2018	2.29±0.17	Lake 61 09/02/2018	3.40±0.25
Lake 17 08/26/2018	2.37±0.15	Lake 61 09/22/2018	1.94±0.15
Lake 17 07/15/2019	2.06±0.15	Lake 61 07/21/2019	2.48±0.16
Lake 17 08/12/2019	2.07±0.15	Lake 61 07/23/2019	2.89±0.22
Lake 18 08/05/2018	2.11±0.16	Lake 66 08/16/2019	3.00±0.21
Lake 18 08/18/2018	2.10±0.16	Lake 68 09/17/2019	2.35±0.18
Lake 18 09/09/2018	1.94±0.15	Lake 69 06/09/2019	1.80±0.13
Lake 18 09/30/2018	2.46±0.19	Lake 69 08/19/2019	1.85±0.14
Lake 18 06/15/2019	2.11±0.15	Lake 72 07/12/2019	1.96±0.14
Lake 18 08/12/2019	1.94±0.15	Lake 73 07/20/2019	2.00±0.14
Lake 21 07/02/2019	2.01±0.12	Lake 73 08/24/2019	2.21±0.17
Lake 21 08/13/2019	2.06±0.16	Lake 74 06/08/2019	1.94±0.14
Lake 22 06/23/2019	2.04±0.16	Lake 74 08/18/2019	1.92±0.14
Lake 22 06/23/2019	2.01±0.14	Lake 75 06/02/2019	2.14±0.16
Lake 22 09/16/2019	2.11±0.15	Lake 77 06/16/2019	2.14±0.16
Lake 22 09/16/2019	2.19±0.16	Lake 77 06/16/2019	2.18±0.14
Lake 23 06/12/2019	1.93±0.13	Lake 77 09/22/2019	2.92±0.21
Lake 23 09/17/2019	2.70±0.19	Lake 77 09/22/2019	2.21±0.17
Lake 25 07/08/2018	2.30±0.17	Lake 78 06/02/2019	2.67±0.20
Lake 25 07/29/2018	2.40±0.19	Lake 88 06/24/2019	2.23±0.18
Lake 25 06/09/2019	2.35±0.18	Lake 88 08/21/2019	1.82±0.13
Lake 29 06/02/2019	2.01±0.15	Lake 89 07/10/2019	1.98±0.14
Lake 29 08/12/2019	1.85±0.14	Lake 90 07/01/2018	1.86±0.15
Lake 31 06/15/2019	2.74±0.21	Lake 90 08/12/2018	2.58±0.20
Lake 31 08/27/2019	2.13±0.15	Lake 90 09/11/2018	1.98±0.15
Lake 33 08/18/2019	2.25±0.15	Lake 90 06/08/2019	1.71±0.13
Lake 33 09/01/2019	2.90±0.21	Lake 90 08/17/2019	1.88±0.14
Lake 34 06/04/2019	1.78±0.14	Lake 92 08/06/2018	3.52±0.27
Lake 34 06/04/2019	1.61±0.12	Lake 92 08/19/2018	3.22±0.25
Lake 37 06/23/2019	2.52±0.20	Lake 92 09/03/2018	2.09±0.15
Lake 37 08/19/2019	2.03±0.17	Lake 92 06/04/2019	3.96±0.32
Lake 38 06/17/2019	1.93±0.14	Lake 92 06/17/2019	3.28±0.25
Lake 39 07/20/2019	2.50±0.15	Lake 92 08/11/2019	2.30±0.17
Lake 40 06/27/2018	1.96±0.15	Lake 92 09/01/2019	1.90±0.15
Lake 40 07/14/2018	3.70±0.29	Lake 96 08/05/2019	2.03±0.14
Lake 40 08/01/2018	2.18±0.15	Lake 99 08/10/2019	1.94±0.15
Lake 40 08/12/2018	3.16±0.22	Lake 100 06/23/2019	1.81±0.15

Table S15. $\Phi_{app, {}^1O_2}$ for whole water samples (continued)			
Sample ID	$\Phi_{app, {}^1O_2}$ ($\times10^{-2}$ mol mol-photons^{-1})	Sample ID	$\Phi_{app, {}^1O_2}$ ($\times10^{-2}$ mol mol-photons^{-1})
Lake 100 08/18/2019	1.81±0.14	Lake 182 06/18/2019	2.40±0.17
Lake 102 06/16/2019	1.90±0.13	Lake 182 07/01/2019	2.17±0.15
Lake 102 09/22/2019	2.30±0.18	Lake 182 07/08/2019	1.89±0.14
Lake 103 06/09/2019	1.58±0.12	Lake 182 08/12/2019	1.93±0.16
Lake 107 08/15/2019	2.02±0.15	Lake 182 08/12/2019	2.14±0.15
Lake 108 08/17/2019	2.15±0.15	Lake 182 08/27/2019	2.05±0.15
Lake 109 07/08/2019	2.56±0.20	Lake 183 08/25/2019	1.91±0.15
Lake 109 08/18/2019	3.19±0.25	Lake 183 09/10/2019	1.97±0.15
Lake 115 07/08/2019	2.08±0.16	Lake 190 06/17/2019	3.26±0.24
Lake 115 07/08/2019	2.31±0.18	Lake 190 08/19/2019	3.26±0.24
Lake 115 09/09/2019	1.96±0.14	Lake 192 09/25/2019	1.89±0.13
Lake 115 09/09/2019	2.03±0.15	Lake 194 06/26/2018	1.97±0.15
Lake 117 08/22/2019	1.94±0.14	Lake 194 07/09/2018	1.89±0.14
Lake 117 09/05/2019	1.95±0.14	Lake 194 07/23/2018	2.25±0.17
Lake 120 06/10/2019	2.01±0.16	Lake 194 08/07/2018	2.27±0.16
Lake 120 08/19/2019	2.14±0.15	Lake 194 08/20/2018	2.09±0.16
Lake 126 07/08/2018	1.96±0.15	Lake 194 09/09/2018	2.18±0.17
Lake 126 07/21/2018	2.12±0.16	Lake 194 09/22/2019	2.47±0.18
Lake 126 08/17/2019	2.06±0.15	Lake 195 06/03/2019	1.58±0.12
Lake 130 08/25/2019	2.04±0.15	Lake 199 06/19/2018	2.39±0.19
Lake 130 09/22/2019	2.12±0.18	Lake 199 07/03/2018	1.62±0.12
Lake 132 06/10/2019	1.71±0.12	Lake 199 07/18/2018	4.15±0.30
Lake 132 09/03/2019	2.44±0.17	Lake 199 07/31/2018	2.64±0.20
Lake 133 06/04/2019	1.89±0.15	Lake 199 08/14/2018	1.49±0.11
Lake 133 08/19/2019	2.17±0.16	Lake 199 08/28/2018	1.92±0.15
Lake 135 05/27/2019	1.89±0.12	Lake 199 09/11/2018	3.04±0.22
Lake 135 08/31/2019	2.13±0.15	Lake 199 06/23/2019	3.43±0.24
Lake 136 06/08/2019	1.92±0.15	Lake 199 07/08/2019	1.73±0.13
Lake 136 09/20/2019	2.44±0.17	Lake 199 07/30/2019	2.71±0.21
Lake 137 08/18/2019	3.13±0.24	Lake 199 08/18/2019	2.29±0.18
Lake 139 08/06/2018	1.47±0.12	Lake 199 09/15/2019	3.79±0.29
Lake 139 09/19/2018	2.38±0.18	Lake 203 06/16/2019	1.73±0.13
Lake 145 06/06/2019	1.76±0.13	Lake 203 09/22/2019	1.98±0.15
Lake 145 08/15/2019	1.84±0.14	Lake 205 06/08/2019	1.97±0.15
Lake 149 06/10/2019	1.95±0.14	Lake 205 08/06/2019	1.99±0.15
Lake 149 09/21/2019	2.42±0.19	Lake 209 08/18/2019	1.80±0.13
Lake 153 08/25/2019	1.72±0.13	Lake 210 06/02/2019	1.56±0.12
Lake 153 09/08/2019	1.92±0.14	Lake 210 08/18/2019	1.50±0.11
Lake 164 06/23/2019	2.00±0.14	Lake 212 06/16/2018	2.12±0.14
Lake 164 08/27/2019	2.03±0.16	Lake 212 07/14/2018	2.34±0.18
Lake 166 07/14/2019	2.28±0.18	Lake 212 07/28/2018	2.47±0.18
Lake 166 09/29/2019	2.01±0.16	Lake 212 08/12/2018	2.14±0.15
Lake 169 07/22/2019	2.38±0.17	Lake 212 06/17/2019	2.46±0.19
Lake 169 08/18/2019	2.35±0.18	Lake 213 08/18/2019	2.30±0.16
Lake 176 07/15/2018	1.85±0.13	Lake 215 06/09/2019	2.67±0.20
Lake 176 06/15/2019	1.72±0.12	Lake 215 09/15/2019	2.36±0.18
Lake 176 08/19/2019	1.80±0.14	Lake 223 08/19/2019	2.48±0.19
Lake 177 08/19/2019	1.89±0.14	Lake 225 07/28/2019	2.03±0.16
Lake 178 08/06/2019	2.01±0.15	Lake 229 06/29/2019	1.93±0.14
Lake 182 07/17/2018	2.10±0.16	Lake 230 06/08/2019	2.26±0.17
Lake 182 07/31/2018	2.46±0.19	Lake 231 06/04/2019	1.93±0.14
Lake 182 08/14/2018	3.38±0.24	Lake 234 06/20/2018	2.91±0.21
Lake 182 09/11/2018	3.57±0.24	Lake 234 07/15/2018	3.11±0.22
Lake 182 06/18/2019	3.25±0.24	Lake 234 06/09/2019	1.84±0.14

Table S15. $\Phi_{app, {}^1O_2}$ for whole water samples (continued)			
Sample ID	$\Phi_{app, {}^1O_2}$ ($\times 10^{-2}$ mol mol-photons^{-1})	Sample ID	$\Phi_{app, {}^1O_2}$ ($\times 10^{-2}$ mol mol-photons^{-1})
Lake 234 08/12/2019	1.93±0.14	Lake 256 07/05/2018	2.97±0.21
Lake 235 07/14/2019	1.76±0.13	Lake 256 07/16/2018	2.85±0.23
Lake 236 07/14/2019	2.86±0.22	Lake 256 07/31/2018	2.22±0.17
Lake 236 09/15/2019	2.08±0.16	Lake 256 08/15/2018	2.67±0.21
Lake 238 08/12/2019	2.53±0.19	Lake 256 08/29/2018	2.58±0.20
Lake 239 06/09/2019	2.19±0.17	Lake 256 09/14/2018	2.35±0.17
Lake 239 08/18/2019	2.28±0.17	Lake 256 09/28/2018	2.34±0.17
Lake 245 09/22/2019	2.09±0.16	Lake 256 06/18/2019	1.60±0.12
Lake 246 08/05/2019	2.03±0.16	Lake 256 06/18/2019	2.21±0.15
Lake 247 06/08/2019	1.95±0.14	Lake 256 08/13/2019	2.10±0.15
Lake 247 09/20/2019	2.27±0.17	Lake 257 08/24/2019	2.24±0.16
Lake 248 08/18/2019	1.91±0.14	Lake 258 06/16/2019	2.39±0.17
Lake 249 07/08/2019	1.91±0.13	Lake 258 08/11/2019	2.19±0.15
Lake 249 09/01/2019	1.95±0.14	Lake 259 09/01/2019	3.63±0.26
Lake 250 07/08/2019	2.35±0.18	Lake 260 06/01/2019	1.72±0.13
Lake 250 08/18/2019	1.85±0.14	Lake 260 08/12/2019	2.10±0.15
Lake 251 08/27/2019	2.42±0.18	Lake 261 06/17/2019	2.31±0.17
Lake 251 09/17/2019	2.48±0.17	Lake 261 08/12/2019	2.14±0.17
Lake 253 07/02/2019	2.40±0.17	Lake 262 06/17/2019	1.81±0.14
Lake 253 08/25/2019	3.44±0.24	Lake 262 09/21/2019	1.92±0.15
Lake 253 09/15/2019	1.98±0.13		

Table S16. $\Phi_{app, {}^1O_2}$ for bloom lysates

Sample ID	Bloom ID	OD_{680} (A.U.)	$\Phi_{app, {}^1O_2}$ ($\times 10^{-2}$ mol mol-photons^{-1})	Sample ID	Bloom ID	OD_{680} (A.U.)	$\Phi_{app, {}^1O_2}$ ($\times 10^{-2}$ mol mol-photons^{-1})
Lake 261 09/04/2021	A	0.379±0.009	0.92±0.09				
Lake 238 09/07/2021	B	0.349±0.018	0.93±0.09				
Lake 147 08/30/2021	C	0.370±0.019	1.00±0.08				
Lake 138 09/07/2021	D	0.417±0.026	0.99±0.09				
Lake 33 09/04/2021	E	0.368±0.018	0.84±0.07				
Lake 37 08/20/2021	F	0.267±0.013	0.82±0.07				
Lake 38 08/29/2021	G	0.430±0.022	1.19±0.10				
Lake 40 09/16/2021	H	0.392±0.020	1.23±0.12				
Lake 78 08/22/2021	I	0.410±0.020	1.14±0.12				
Lake 82 08/17/2021	J	0.475±0.024	1.30±0.14				
Lake 93 08/29/2021	K	0.245±0.012	0.88±0.07				
Lake 221 09/06/2021	L	0.542±0.027	1.35±0.11				
Lake 256 10/12/2021	Otisco	0.026±0.001	1.72±0.07	SRNOM	-	-	2.57±0.23
Lysate 25% + Otisco 75%	A	-	1.25±0.12	Lysate 25% + SRNOM 75%	A	-	1.90±0.12
Lysate 50% + Otisco 50%	A	-	1.03±0.09	Lysate 50% + SRNOM 50%	A	-	1.46±0.08
Lysate 75% + Otisco 25%	A	-	0.88±0.03	Lysate 75% + SRNOM 25%	A	-	1.05±0.04
Lysate 25% + Otisco 75%	B	-	1.29±0.11	Lysate 25% + SRNOM 75%	B	-	1.93±0.10
Lysate 50% + Otisco 50%	B	-	1.07±0.10	Lysate 50% + SRNOM 50%	B	-	1.44±0.07
Lysate 75% + Otisco 25%	B	-	0.89±0.09	Lysate 75% + SRNOM 25%	B	-	1.02±0.05
Lysate 25% + Otisco 75%	C	-	1.31±0.14	Lysate 25% + SRNOM 75%	C	-	1.90±0.16
Lysate 50% + Otisco 50%	C	-	1.11±0.09	Lysate 50% + SRNOM 50%	C	-	1.55±0.08
Lysate 75% + Otisco 25%	C	-	0.97±0.09	Lysate 75% + SRNOM 25%	C	-	1.15±0.04
Lysate 25% + Otisco 75%	D	-	1.37±0.13	Lysate 25% + SRNOM 75%	D	-	1.96±0.11
Lysate 50% + Otisco 50%	D	-	1.18±0.11	Lysate 50% + SRNOM 50%	D	-	1.57±0.07
Lysate 75% + Otisco 25%	D	-	1.05±0.02	Lysate 75% + SRNOM 25%	D	-	1.18±0.04
Lysate 25% + Otisco 75%	E	-	1.31±0.11	Lysate 25% + SRNOM 75%	E	-	1.90±0.11
Lysate 50% + Otisco 50%	E	-	1.11±0.06	Lysate 50% + SRNOM 50%	E	-	1.44±0.08
Lysate 75% + Otisco 25%	E	-	0.97±0.09	Lysate 75% + SRNOM 25%	E	-	1.07±0.11
Lysate 25% + Otisco 75%	F	-	1.37±0.11	Lysate 25% + SRNOM 75%	F	-	1.98±0.14
Lysate 50% + Otisco 50%	F	-	1.18±0.09	Lysate 50% + SRNOM 50%	F	-	1.53±0.10
Lysate 75% + Otisco 25%	F	-	1.04±0.09	Lysate 75% + SRNOM 25%	F	-	1.15±0.07

Table S17. $\Phi_{app, {}^1O_2}$ for bloom supernatants

Sample ID	Bloom ID	OD_{680} (A.U.)	$\Phi_{app, {}^1O_2}$ ($\times10^{-2}$ mol mol-photons^{-1})	Sample ID	Bloom ID	OD_{680} (A.U.)	$\Phi_{app, {}^1O_2}$ ($\times10^{-2}$ mol mol-photons^{-1})
Lake 261 09/04/2021	A	0.151±0.003	2.95±0.11	Lake 33 09/04/2021	E	0.368±0.018	3.84±0.25
	A	0.182±0.006	3.48±0.14	Lake 37 08/20/2021	F	0.267±0.013	3.36±0.29
	A	0.215±0.008	3.92±0.27	Lake 38 08/29/2021	G	0.430±0.022	5.10±0.31
	A	0.259±0.009	4.23±0.30	Lake 40 09/16/2021	H	0.392±0.020	5.34±0.32
	A	0.306±0.009	4.44±0.31	Lake 78 08/22/2021	I	0.410±0.020	5.01±0.32
	A	0.347±0.009	4.56±0.32	Lake 82 08/17/2021	J	0.475±0.024	5.66±0.39
	A	0.370±0.009	4.62±0.35	Lake 93 08/29/2021	K	0.245±0.012	3.48±0.18
	A	0.376±0.009	4.63±0.29	Lake 221 09/06/2021	L	0.542±0.027	6.83±0.43
	A	0.379±0.009	4.63±0.33				
Lake 238 09/07/2021	B	0.138±0.003	2.98±0.26	Lake 256 10/12/2021	Otisco	0.008±0.001	1.82±0.08
	B	0.159±0.006	3.52±0.25		Otisco	0.013±0.001	1.81±0.05
	B	0.195±0.009	4.02±0.30		Otisco	0.016±0.001	1.80±0.04
	B	0.242±0.009	4.42±0.30		Otisco	0.018±0.002	1.79±0.06
	B	0.283±0.014	4.65±0.31		Otisco	0.020±0.002	1.78±0.06
	B	0.321±0.015	4.81±0.39		Otisco	0.022±0.002	1.77±0.07
	B	0.338±0.017	4.84±0.39		Otisco	0.023±0.002	1.75±0.09
	B	0.345±0.017	4.85±0.38		Otisco	0.024±0.002	1.74±0.08
	B	0.349±0.018	4.86±0.38		Otisco	0.025±0.002	1.72±0.08
Lake 147 08/30/2021	C	0.078±0.009	3.36±0.23	Lake 256 10/12/2021	Otisco	0.009±0.001	1.83±0.08
	C	0.150±0.011	4.39±0.26		Otisco	0.014±0.001	1.81±0.06
	C	0.208±0.016	4.98±0.33		Otisco	0.017±0.001	1.80±0.08
	C	0.264±0.018	5.40±0.32		Otisco	0.019±0.001	1.79±0.06
	C	0.310±0.020	5.56±0.34		Otisco	0.021±0.002	1.78±0.09
	C	0.340±0.019	5.65±0.32		Otisco	0.023±0.002	1.77±0.07
	C	0.354±0.019	5.69±0.34		Otisco	0.024±0.002	1.75±0.08
	C	0.366±0.019	5.71±0.39		Otisco	0.025±0.002	1.73±0.07
	C	0.370±0.019	5.73±0.41		Otisco	0.026±0.002	1.72±0.06
Lake 138 09/07/2021	D	0.107±0.011	3.28±0.26				
	D	0.202±0.024	4.30±0.33				
	D	0.280±0.029	4.90±0.36				
	D	0.326±0.028	5.19±0.38				
	D	0.365±0.023	5.34±0.43				
	D	0.389±0.025	5.42±0.42				
	D	0.402±0.024	5.45±0.32				
	D	0.412±0.026	5.47±0.31				
	D	0.417±0.026	5.48±0.33				

**Table S17.** $\Phi_{app,\ ^1O_2}$ for bloom supernatants (continued)						
Sample ID	Bloom ID	OD_{680} (A.U.)	$\Phi_{app,\ ^1O_2,\ high\text{-}energy}$ ($\times 10^{-2}$ mol mol-photons^{-1})	$\Phi_{app,\ ^1O_2,\ low\text{-}energy}$ ($\times 10^{-2}$ mol mol-photons^{-1})	$\%\Phi_{app,\ ^1O_2,\ high\text{-}energy}$	$\%\Phi_{app,\ ^1O_2,\ low\text{-}energy}$
Lake 261 09/04/2021	A	0.379±0.009	2.51±0.12	2.13±0.22	54.2±1.4	43.9±0.1
Lake 238 09/07/2021	B	0.349±0.018	2.62±0.12	2.24±0.26	54.1±1.8	43.2±1.0
Lake 147 08/30/2021	C	0.370±0.019	3.23±0.14	2.51±0.27	56.4±1.6	38.9±0.2
Lake 138 09/07/2021	D	0.417±0.026	3.06±0.10	2.42±0.23	55.8±1.5	40.4±1.0
Lake 33 09/04/2021	E	0.368±0.018	1.99±0.09	1.86±0.17	51.7±1.2	48.3±1.2
Lake 37 08/20/2021	F	0.267±0.013	1.66±0.11	1.69±0.18	49.6±0.9	50.4±0.9
Lake 38 08/29/2021	G	0.430±0.022	2.87±0.09	2.24±0.22	56.3±1.7	43.7±1.7
Lake 40 09/16/2021	H	0.392±0.020	3.03±0.08	2.31±0.24	56.9±1.8	43.1±1.8
Lake 78 08/22/2021	I	0.410±0.020	2.84±0.12	2.17±0.20	56.8±1.3	43.2±1.3
Lake 82 08/17/2021	J	0.475±0.024	3.21±0.14	2.44±0.25	56.8±1.5	43.2±1.5
Lake 93 08/29/2021	K	0.245±0.012	1.74±0.01	1.74±0.18	50.0±2.5	50.0±2.5
Lake 221 09/06/2021	L	0.542±0.027	4.03±0.13	2.80±0.29	59.1±1.7	40.9±1.7
Lake 256 10/12/2021	Otisco	0.026±0.001	0.56±0.02	1.17±0.09	32.3±2.7	67.7±2.7

**Table S18.** $\Phi_{app, {}^1O_2}$ for bloom supernatants minus the contribution from Otisco Lake water							
Sample ID	Bloom ID	ΔOD_{680} (A.U.)	$\Delta\Phi_{app, {}^1O_2}$ ($\times 10^{-2}$ mol mol-photons^{-1})	Sample ID	Bloom ID	ΔOD_{680} (A.U.)	$\Delta\Phi_{app, {}^1O_2}$ ($\times 10^{-2}$ mol mol-photons^{-1})
Lake 261 09/04/2021	A	0.143±0.007	1.13±0.03	Lake 33 09/04/2021	E	0.343±0.017	2.12±0.18
	A	0.168±0.008	1.67±0.14	Lake 37 08/20/2021	F	0.242±0.012	1.63±0.22
	A	0.199±0.010	2.12±0.21	Lake 38 08/29/2021	G	0.405±0.020	3.38±0.24
	A	0.241±0.012	2.44±0.24	Lake 40 09/16/2021	H	0.367±0.018	3.62±0.25
	A	0.286±0.014	2.67±0.23	Lake 78 08/22/2021	I	0.384±0.019	3.29±0.25
	A	0.325±0.016	2.80±0.25	Lake 82 08/17/2021	J	0.450±0.022	3.94±0.32
	A	0.346±0.017	2.87±0.26	Lake 93 08/29/2021	K	0.219±0.011	1.76±0.12
	A	0.352±0.018	2.89±0.22	Lake 221 09/06/2021	L	0.517±0.026	5.11±0.36
	A	0.354±0.018	2.91±0.26				
Lake 238 09/07/2021	B	0.130±0.006	1.15±0.18				
	B	0.146±0.007	1.70±0.24				
	B	0.179±0.009	2.22±0.24				
	B	0.224±0.011	2.63±0.24				
	B	0.263±0.013	2.87±0.24				
	B	0.299±0.015	3.04±0.32				
	B	0.315±0.016	3.09±0.30				
	B	0.320±0.016	3.12±0.30				
	B	0.323±0.016	3.14±0.31				
Lake 147 08/30/2021	C	0.070±0.003	1.53±0.15				
	C	0.137±0.007	2.57±0.25				
	C	0.192±0.010	3.17±0.27				
	C	0.246±0.012	3.60±0.25				
	C	0.290±0.015	3.78±0.27				
	C	0.318±0.016	3.88±0.24				
	C	0.331±0.017	3.94±0.25				
	C	0.341±0.017	3.98±0.31				
	C	0.345±0.017	4.01±0.34				
Lake 138 09/07/2021	D	0.099±0.005	1.45±0.18				
	D	0.189±0.009	2.49±0.32				
	D	0.264±0.013	3.10±0.30				
	D	0.308±0.015	3.40±0.31				
	D	0.345±0.017	3.56±0.36				
	D	0.367±0.018	3.65±0.34				
	D	0.379±0.019	3.70±0.23				
	D	0.388±0.019	3.74±0.24				
	D	0.391±0.020	3.76±0.26				

**Table S18.** $\Phi_{app,\, ^1O_2}$ for bloom supernatants minus the contribution from Otisco Lake water (continued)						
Sample ID	Bloom ID	ΔOD_{680} (A.U.)	$\Delta\Phi_{app,\, ^1O_2,\, high\text{-}energy}$ ($\times 10^{-2}$ mol mol-photons^{-1})	$\Delta\Phi_{app,\, ^1O_2,\, low\text{-}energy}$ ($\times 10^{-2}$ mol mol-photons^{-1})	$\%\Delta\Phi_{app,\, ^1O_2,\, high\text{-}energy}$	$\%\Delta\Phi_{app,\, ^1O_2,\, low\text{-}energy}$
Lake 261 09/04/2021	A	0.354±0.018	1.95±0.14	0.96±0.12	67.1±1.3	32.9±1.3
Lake 238 09/07/2021	B	0.323±0.016	2.07±0.14	1.07±0.17	66.0±2.0	34.0±2.0
Lake 147 08/30/2021	C	0.345±0.017	2.67±0.16	1.34±0.18	66.7±1.6	33.3±1.6
Lake 138 09/07/2021	D	0.391±0.020	2.50±0.12	1.26±0.13	66.6±1.3	33.4±1.3
Lake 33 09/04/2021	E	0.343±0.017	1.43±0.11	0.69±0.07	67.4±0.6	32.6±0.6
Lake 37 08/20/2021	F	0.242±0.012	1.11±0.14	0.53±0.08	67.7±0.7	32.3±0.7
Lake 38 08/29/2021	G	0.405±0.020	2.31±0.11	1.07±0.13	68.4±1.6	31.6±1.6
Lake 40 09/16/2021	H	0.367±0.018	2.48±0.11	1.14±0.14	68.6±1.8	31.4±1.8
Lake 78 08/22/2021	I	0.384±0.019	2.29±0.14	1.00±0.11	69.6±1.0	30.4±1.0
Lake 82 08/17/2021	J	0.450±0.022	2.66±0.16	1.28±0.16	67.6±1.4	32.4±1.4
Lake 93 08/29/2021	K	0.219±0.011	1.18±0.03	0.58±0.09	67.2±2.8	32.8±2.8
Lake 221 09/06/2021	L	0.517±0.026	3.48±0.16	1.63±0.20	68.1±1.7	31.9±1.7

9. 2,4,6-Trimethylphenol (TMP) as an electron transfer probe for $^3DOM^*$

TMP was spiked into samples to measure the photoproduction of $^3DOM^*_{TMP}$. For each sample, the loss of TMP was monitored to determine the pseudo-first order rate constant for the photodegradation of TMP, $k_{obs,\,TMP}$ (s^{-1}), with the negligible contribution from direct photolysis of TMP (1.7±0.9% for whole water samples, 3.8±2.1% for bloom lysates, and 1.3±1.5% for bloom supernatants, respectively)[76] and its reactions with $^\bullet OH$ (3.6±0.8% for whole water samples, 3.6±0.5% for bloom lysates, and 2.7±0.9% for bloom supernatants, respectively) and $^1O_2$ (6.2±0.7% for whole water samples, 9.3±0.9% for bloom lysates, and 7.9±0.9% for bloom supernatants, respectively):[28, 77-85]

$$R_{loss,\,TMP} = -\frac{d[TMP]}{dt} = k_{obs,\,TMP}[TMP]$$

$$= k_{TMP,\,^3DOM^*_{TMP}}[TMP][^3DOM^*_{TMP}]_{ss} + k_{direct\ photolysis,\,TMP}[TMP]SF_{\Sigma\lambda} + k_{TMP,\,^1O_2}[TMP][^1O_2]_{ss} + k_{TMP,\,^\bullet OH}[TMP][^\bullet OH]_{ss} \quad (S15)$$

where $R_{loss,\,TMP}$ (M s^{-1}) is the loss rate of TMP, [TMP] is the initial concentration of TMP (10 μM), $k_{TMP,\,^3DOM^*_{TMP}}$ (M^{-1} s^{-1}) is the second-order reaction rate constant of TMP with $^3DOM^*_{TMP}$, $[^3DOM^*_{TMP}]_{ss}$ is the steady-state concentration of $^3DOM^*_{TMP}$, $k_{direct\ photolysis,\,TMP}$ (s^{-1}) is the experimentally determined direct photolysis rate constant of TMP, $SF_{\Sigma\lambda}$ is the sample-specific light screening factor, $k_{TMP,\,^1O_2}$ (5.1(±0.2)×10^7 M^{-1} s^{-1}) is the second-order reaction rate constant of TMP with $^1O_2$,[86] $[^1O_2]_{ss}$ is the steady-state concentration of $^1O_2$ measured by FFA, $k_{TMP,\,^\bullet OH}$ (1.6(±0.1)×10^{10} M^{-1} s^{-1}) is the estimated second-order reaction rate constant of TMP with $^\bullet OH$,[56, 87] and $[^\bullet OH]_{ss}$ is the steady-state concentration of $^\bullet OH$ measured by TPA.

To account for the inhibition of TMP loss by reduced DOM moieties,[88-92] the pseudo-first order rate constant for the loss of TMP attributable to $^3DOM^*$ was further corrected for DOM-induced inhibition:[29, 83]

$$k^{corr}_{obs,\,TMP} = \frac{(k_{obs,\,TMP} - k_{direct\ photolysis,\,TMP}SF_{\Sigma\lambda} - k_{TMP,\,^1O_2}[^1O_2]_{ss} - k_{TMP,\,^\bullet OH}[^\bullet OH]_{ss})}{IF_{TMP}} \quad (S16)$$

where $k^{corr}_{obs,\,TMP}$ (s^{-1}) is the pseudo-first order rate constant for the loss of TMP attributable to $^3DOM^*$ corrected for inhibition and IF_{TMP} is the inhibition factor predicted from $1/IF_{TMP} = 0.021[DOC] + 0.965$ [29] using [DOC].

For each sample, the steady-state concentration of $^3\mathrm{DOM}^*_{\mathrm{TMP}}$, $[^3\mathrm{DOM}^*_{\mathrm{TMP}}]_{\mathrm{ss}}$, was calculated as:[83, 93]

$$[^3\mathrm{DOM}^*_{\mathrm{TMP}}]_{\mathrm{ss}} = \frac{R_{\mathrm{f},\,^3\mathrm{DOM}^*_{\mathrm{TMP}}}}{k'_{\mathrm{q},\,^3\mathrm{DOM}^*_{\mathrm{TMP}}}} = \frac{k^{\mathrm{corr}}_{\mathrm{obs,\,TMP}}(k'_{\mathrm{q},\,^3\mathrm{DOM}^*_{\mathrm{TMP}}} + k_{\mathrm{TMP},\,^3\mathrm{DOM}^*_{\mathrm{TMP}}}[\mathrm{TMP}])}{k'_{\mathrm{q},\,^3\mathrm{DOM}^*_{\mathrm{TMP}}}k_{\mathrm{TMP},\,^3\mathrm{DOM}^*_{\mathrm{TMP}}}} \approx \frac{k^{\mathrm{corr}}_{\mathrm{obs,\,TMP}}}{k_{\mathrm{TMP},\,^3\mathrm{DOM}^*_{\mathrm{TMP}}}} \qquad \text{(S17)}$$

where $R_{\mathrm{f},\,^3\mathrm{DOM}^*_{\mathrm{TMP}}}$ (M s^{-1}) is the formation rate of $^3\mathrm{DOM}^*_{\mathrm{TMP}}$, $k'_{\mathrm{q},\,^3\mathrm{DOM}^*_{\mathrm{TMP}}}$ ($3.2(\pm0.6)\times10^5$ s^{-1}; note that $k'_{\mathrm{q},\,^3\mathrm{DOM}^*_{\mathrm{TMP}}} \gg k_{\mathrm{TMP},\,^3\mathrm{DOM}^*_{\mathrm{TMP}}}[\mathrm{TMP}]$ with a maximum value of $9.7(\pm1.2)\times10^3$ s^{-1} at [TMP] = 10 μM) is the sum of pseudo-first order rate constants for $^3\mathrm{DOM}^*_{\mathrm{TMP}}$ quenching via energy transfer to dissolved O_2 ($2.3(\pm0.2)\times10^5$ s^{-1} calculated from $k_{O_2}[O_{2(aq)}]$ where k_{O_2} = $8.9(\pm0.6)\times10^8$ M^{-1} s^{-1} and $[O_{2(aq)}]$ = ~258 μM at T = 25 °C[94]) and via other non-O_2 dependent nonradiative relaxation pathways ($k^{\mathrm{T}}_{\mathrm{d}}$ = $9.0(\pm2.8)\times10^4$ s^{-1}).[75, 86]

To solve for $k_{\mathrm{TMP},\,^3\mathrm{DOM}^*_{\mathrm{TMP}}}$ (**Table S19**), a linear regression of [TMP] (at varying concentrations of 10, 20, 50, 100, and 250 μM) and $k^{\mathrm{corr}}_{\mathrm{obs,\,TMP}}$ data was performed using the linearized form of Equation S18 (i.e., $y = ax + b$ where $y = 1/k^{\mathrm{corr,IF}}_{\mathrm{obs,\,TMP}}$ and x = [TMP]):[29, 35, 83, 95, 96]

$$\frac{1}{k^{\mathrm{corr}}_{\mathrm{obs,\,TMP}}} = \frac{[\mathrm{TMP}]}{R_{\mathrm{f},\,^3\mathrm{DOM}^*_{\mathrm{TMP}}}} + \frac{k'_{\mathrm{q},\,^3\mathrm{DOM}^*_{\mathrm{TMP}}}}{R_{\mathrm{f},\,^3\mathrm{DOM}^*_{\mathrm{TMP}}}k_{\mathrm{TMP},\,^3\mathrm{DOM}^*_{\mathrm{TMP}}}} \qquad \text{(S18)}$$

The formation rate of $^3\mathrm{DOM}^*_{\mathrm{TMP}}$ (at [TMP] = 10 μM), $R_{\mathrm{f},\,^3\mathrm{DOM}^*_{\mathrm{TMP}}}$ (M s^{-1}), was calculated as:

$$R_{\mathrm{f},\,^3\mathrm{DOM}^*_{\mathrm{TMP}}} = [^3\mathrm{DOM}^*_{\mathrm{TMP}}]_{\mathrm{ss}}k'_{\mathrm{q},\,^3\mathrm{DOM}^*_{\mathrm{TMP}}} \qquad \text{(S19)}$$

The apparent quantum yield of $^3\mathrm{DOM}^*_{\mathrm{TMP}}$, $\Phi_{\mathrm{app},\,^3\mathrm{DOM}^*_{\mathrm{TMP}}}$ (mol mol-photons^{-1}), was calculated as:[29, 35, 83, 97]

$$\Phi_{\mathrm{app},\,^3\mathrm{DOM}^*_{\mathrm{TMP}}} = \frac{R_{\mathrm{f},\,^3\mathrm{DOM}^*_{\mathrm{TMP}}}}{R_{\mathrm{a}}} \approx \frac{[^3\mathrm{DOM}^*_{\mathrm{TMP}}]_{\mathrm{ss}}k'_{\mathrm{q},\,^3\mathrm{DOM}^*_{\mathrm{TMP}}}}{R_{\mathrm{a}}} \qquad \text{(S20)}$$

The quantum yield coefficient of $^3\mathrm{DOM}^*_{\mathrm{TMP}}$ with TMP, f_{TMP} (L mol-photons^{-1}), was calculated as:[36, 37, 75, 78, 82, 93, 97-102]

$$f_{\mathrm{TMP}} = \Phi_{\mathrm{app},\,^3\mathrm{DOM}^*_{\mathrm{TMP}}} \times \frac{k_{\mathrm{TMP},\,^3\mathrm{DOM}^*_{\mathrm{TMP}}}}{k'_{\mathrm{q},\,^3\mathrm{DOM}^*_{\mathrm{TMP}}}} = \frac{k^{\mathrm{corr}}_{\mathrm{obs,\,TMP}}}{R_{\mathrm{a}}} \qquad \text{(S21)}$$

To determine $\Phi_{\text{app, }^3\text{DOM}^*_{\text{TMP}}}$ attributable to high-energy $^3DOM^*$ (i.e., $\Phi_{\text{app, }^3\text{DOM}^*_{\text{TMP}}\text{, high-energy}}$) and low-energy $^3DOM^*$ (i.e., $\Phi_{\text{app, }^3\text{DOM}^*_{\text{TMP}}\text{, low-energy}}$) for the supernatants of recultivated bloom samples, *t,t*-HDO (2 mM) was spiked into TMP-containing (10 μM) supernatants to preferentially quench high-energy $^3DOM^*$ capable of sensitizing *t,t*-HDO isomerization (i.e., $^3DOM^*$ with $E_T \geq 250$ kJ mol^{-1} [74]). The percentage contribution of $\Phi_{\text{app, }^3\text{DOM}^*_{\text{TMP}}\text{, high-energy}}$ to $\Phi_{\text{app, }^3\text{DOM}^*_{\text{TMP}}}$, and the percentage contribution of $\Phi_{\text{app, }^3\text{DOM}^*_{\text{TMP}}\text{, low-energy}}$ to $\Phi_{\text{app, }^3\text{DOM}^*_{\text{TMP}}}$, and the yield of $^1O_2$ from the O_2-dependent quenching of $^3DOM^*_{TMP}$ were calculated as:[75, 103]

$$\%\Phi_{\text{app, }^3\text{DOM}^*_{\text{TMP}}\text{, high-energy}} = \%\Phi_{\text{app, }^3\text{DOM}^*_{\text{TMP}\_}{}^3\text{DOM}^*_{\text{HDO}}} = \frac{(\Phi_{\text{app, }^3\text{DOM}^*_{\text{TMP}}} - \Phi_{\text{app, }^3\text{DOM}^*_{\text{TMP}}\text{, HDO}})}{\Phi_{\text{app, }^3\text{DOM}^*_{\text{TMP}}}} \times 100\% \quad \text{(S22)}$$

$$\%\Phi_{\text{app, }^3\text{DOM}^*_{\text{TMP}}\text{, low-energy}} = \frac{\Phi_{\text{app, }^3\text{DOM}^*_{\text{TMP}}\text{, HDO}}}{\Phi_{\text{app, }^3\text{DOM}^*_{\text{TMP}}}} \times 100\% \quad \text{(S23)}$$

$$f_{^1\text{O}_2\_{}^3\text{DOM}^*_{\text{TMP}}} = \frac{\Phi_{\text{app, }^1\text{O}_2}(k_{\text{O}_2}[\text{O}_{2(\text{aq})}] + k_{\text{d}}^{\text{T}})}{\Phi_{\text{app, }^3\text{DOM}^*_{\text{TMP}}} k_{\text{O}_2}[\text{O}_{2(\text{aq})}]} \quad \text{(S24)}$$

$\Phi_{\text{app, }^3\text{DOM}^*_{\text{TMP}}}$ for whole water and bloom samples are summarized in **Tables S20-S23**. f_{TMP} for whole water and bloom samples are summarized in **Tables S24-S27**.

**Table S19.** $k_{TMP,\ ^3DOM^*_{TMP}}$ for whole water and bloom samples			
Sample ID	Bloom ID	OD_{680} (A.U.)	$k_{TMP,\ ^3DOM^*_{TMP}}$ ($\times 10^8\ M^{-1}\ s^{-1}$)
**Pooled whole water samples**			
Lake 2 06/08/2019 to Lake 17 07/15/2018 (*n*=16)	-	-	8.51±0.43
Lake 17 07/28/2018 to Lake 22 09/16/2019 (*n*=16)	-	-	7.36±0.44
Lake 22 09/16/2019 to Lake 37 08/19/2019 (*n*=16)	-	-	8.38±0.19
Lake 38 06/17/2019 to Lake 52 06/30/2019 (*n*=16)	-	-	8.19±0.33
Lake 53 06/09/2019 to Lake 72 07/12/2019 (*n*=16)	-	-	8.58±0.15
Lake 73 07/20/2019 to Lake 90 09/11/2018 (*n*=16)	-	-	7.61±0.38
Lake 90 06/08/2019 to Lake 103 06/09/2019 (*n*=16)	-	-	7.98±0.30
Lake 107 08/15/2019 to Lake 130 08/25/2019 (*n*=16)	-	-	7.73±0.17
Lake 130 09/22/2019 to Lake 149 09/21/2019 (*n*=16)	-	-	8.36±0.31
Lake 153 08/25/2019 to Lake 182 08/14/2018 (*n*=16)	-	-	8.79±0.28
Lake 182 09/11/2018 to Lake 194 07/23/2018 (*n*=16)	-	-	8.64±0.35
Lake 194 08/07/2018 to Lake 199 08/18/2019 (*n*=16)	-	-	8.08±0.53
Lake 199 09/15/2019 to Lake 215 09/15/2019 (*n*=16)	-	-	8.14±0.25
Lake 223 08/19/2019 to Lake 245 09/22/2019 (*n*=16)	-	-	8.03±0.22
Lake 246 08/05/2019 to Lake 256 07/31/2018 (*n*=16)	-	-	8.70±0.35
Lake 256 08/15/2018 to Lake 262 09/21/2019 (*n*=17)	-	-	7.87±0.10
**Bloom lysates**			
Lake 261 09/04/2021	A	0.379±0.009	4.66±0.30
Lake 238 09/07/2021	B	0.349±0.018	4.80±0.68
Lake 147 08/30/2021	C	0.370±0.019	4.55±0.22
Lake 138 09/07/2021	D	0.417±0.026	4.86±0.31
Lake 33 09/04/2021	E	0.368±0.018	4.57±0.30
Lake 37 08/20/2021	F	0.267±0.013	4.34±0.54
Lake 38 08/29/2021	G	0.430±0.022	4.86±0.34
Lake 40 09/16/2021	H	0.392±0.020	5.04±0.30
Lake 78 08/22/2021	I	0.410±0.020	4.72±0.39
Lake 82 08/17/2021	J	0.475±0.024	4.94±0.36
Lake 93 08/29/2021	K	0.245±0.012	4.45±0.32
Lake 221 09/06/2021	L	0.542±0.027	5.13±0.20
**Bloom supernatants**			
Lake 261 09/04/2021	A	0.379±0.009	7.75±0.29
Lake 238 09/07/2021	B	0.349±0.018	8.04±0.58
Lake 147 08/30/2021	C	0.370±0.019	8.53±0.34
Lake 138 09/07/2021	D	0.417±0.026	8.31±0.27
Lake 33 09/04/2021	E	0.368±0.018	7.59±0.22
Lake 37 08/20/2021	F	0.267±0.013	7.22±0.39
Lake 38 08/29/2021	G	0.430±0.022	8.79±0.46
Lake 40 09/16/2021	H	0.392±0.020	8.93±0.25
Lake 78 08/22/2021	I	0.410±0.020	8.52±0.38
Lake 82 08/17/2021	J	0.475±0.024	9.30±0.58
Lake 93 08/29/2021	K	0.245±0.012	7.10±0.47
Lake 221 09/06/2021	L	0.542±0.027	9.83±0.31

**Table S19.** $k_{TMP, ^3DOM^*_{TMP}}$ for whole water and bloom samples (continued)			
Sample ID	Bloom ID	OD_{680} (A.U.)	$k_{TMP, ^3DOM^*_{TMP}}$ ($\times 10^8$ M^{-1} s^{-1})
**Mixtures of bloom lysates and Otisco Lake water / SRNOM**			
Lake 256 10/12/2021	Otisco	0.026±0.001	5.55±0.42
Lysate 25% + Otisco 75%	A	-	5.87±0.32
Lysate 50% + Otisco 50%	A	-	5.44±0.36
Lysate 75% + Otisco 25%	A	-	5.00±0.39
Lysate 25% + Otisco 75%	B	-	5.81±0.40
Lysate 50% + Otisco 50%	B	-	5.32±0.52
Lysate 75% + Otisco 25%	B	-	4.83±0.64
Lysate 25% + Otisco 75%	C	-	5.89±0.32
Lysate 50% + Otisco 50%	C	-	5.48±0.35
Lysate 75% + Otisco 25%	C	-	5.07±0.39
Lysate 25% + Otisco 75%	D	-	5.93±0.45
Lysate 50% + Otisco 50%	D	-	5.55±0.62
Lysate 75% + Otisco 25%	D	-	5.18±0.79
Lysate 25% + Otisco 75%	E	-	5.86±0.29
Lysate 50% + Otisco 50%	E	-	5.42±0.30
Lysate 75% + Otisco 25%	E	-	4.99±0.30
Lysate 25% + Otisco 75%	F	-	5.94±0.32
Lysate 50% + Otisco 50%	F	-	5.58±0.36
Lysate 75% + Otisco 25%	F	-	5.22±0.40
SRNOM	SRNOM	-	6.30±0.20[86]
Lysate 25% + SRNOM 75%	A	-	5.30±0.56
Lysate 50% + SRNOM 50%	A	-	5.06±0.51
Lysate 75% + SRNOM 25%	A	-	4.82±0.47
Lysate 25% + SRNOM 75%	B	-	5.24±0.64
Lysate 50% + SRNOM 50%	B	-	4.94±0.68
Lysate 75% + SRNOM 25%	B	-	4.64±0.72
Lysate 25% + SRNOM 75%	C	-	5.33±0.55
Lysate 50% + SRNOM 50%	C	-	5.10±0.51
Lysate 75% + SRNOM 25%	C	-	4.88±0.46
Lysate 25% + SRNOM 75%	D	-	5.36±0.69
Lysate 50% + SRNOM 50%	D	-	5.17±0.78
Lysate 75% + SRNOM 25%	D	-	4.99±0.87
Lysate 25% + SRNOM 75%	E	-	5.30±0.53
Lysate 50% + SRNOM 50%	E	-	5.05±0.46
Lysate 75% + SRNOM 25%	E	-	4.80±0.38
Lysate 25% + SRNOM 75%	F	-	5.37±0.56
Lysate 50% + SRNOM 50%	F	-	5.20±0.52
Lysate 75% + SRNOM 25%	F	-	5.03±0.48

**Table S20.** $\Phi_{app, ^3DOM^*_{TMP}}$ for whole water samples			
Sample ID	$\Phi_{app, ^3DOM^*_{TMP}}$ ($\times10^{-2}$ mol mol-photons^{-1})	Sample ID	$\Phi_{app, ^3DOM^*_{TMP}}$ ($\times10^{-2}$ mol mol-photons^{-1})
Lake 2 06/08/2019	1.99±0.14	Lake 40 08/29/2018	3.32±0.23
Lake 2 08/31/2019	2.19±0.15	Lake 40 06/19/2019	2.65±0.18
Lake 6 06/18/2019	2.06±0.14	Lake 41 07/07/2018	3.45±0.24
Lake 6 08/28/2019	3.36±0.24	Lake 41 07/17/2018	2.40±0.16
Lake 8 08/05/2019	2.08±0.14	Lake 41 07/29/2018	3.44±0.24
Lake 12 06/08/2019	2.10±0.15	Lake 41 08/19/2018	3.60±0.25
Lake 12 09/01/2019	2.60±0.18	Lake 45 08/18/2019	2.31±0.15
Lake 13 07/08/2018	2.92±0.21	Lake 49 07/08/2019	2.49±0.17
Lake 13 07/22/2018	2.45±0.17	Lake 50 06/10/2019	2.26±0.15
Lake 13 08/06/2018	2.83±0.20	Lake 52 06/30/2019	3.37±0.23
Lake 13 08/21/2018	2.95±0.21	Lake 53 06/09/2019	1.99±0.14
Lake 13 09/02/2018	2.92±0.20	Lake 57 06/29/2019	2.40±0.16
Lake 13 06/10/2019	1.66±0.12	Lake 57 09/08/2019	2.46±0.17
Lake 13 08/31/2019	2.29±0.16	Lake 58 09/10/2019	3.55±0.25
Lake 17 07/01/2018	2.17±0.15	Lake 61 07/09/2018	2.07±0.14
Lake 17 07/15/2018	2.03±0.14	Lake 61 07/19/2018	1.96±0.13
Lake 17 07/28/2018	2.16±0.15	Lake 61 08/19/2018	2.22±0.15
Lake 17 08/12/2018	2.68±0.18	Lake 61 09/02/2018	3.83±0.26
Lake 17 08/26/2018	2.61±0.18	Lake 61 09/22/2018	2.19±0.15
Lake 17 07/15/2019	2.02±0.14	Lake 61 07/21/2019	2.63±0.18
Lake 17 08/12/2019	2.12±0.14	Lake 61 07/23/2019	3.18±0.22
Lake 18 08/05/2018	2.42±0.16	Lake 66 08/16/2019	3.17±0.22
Lake 18 08/18/2018	2.33±0.16	Lake 68 09/17/2019	2.58±0.18
Lake 18 09/09/2018	2.27±0.15	Lake 69 06/09/2019	2.05±0.14
Lake 18 09/30/2018	2.46±0.17	Lake 69 08/19/2019	1.95±0.14
Lake 18 06/15/2019	2.09±0.14	Lake 72 07/12/2019	2.01±0.14
Lake 18 08/12/2019	2.07±0.14	Lake 73 07/20/2019	2.20±0.15
Lake 21 07/02/2019	2.22±0.15	Lake 73 08/24/2019	2.68±0.18
Lake 21 08/13/2019	2.21±0.15	Lake 74 06/08/2019	2.21±0.15
Lake 22 06/23/2019	2.28±0.16	Lake 74 08/18/2019	2.21±0.15
Lake 22 06/23/2019	2.24±0.16	Lake 75 06/02/2019	2.25±0.15
Lake 22 09/16/2019	2.27±0.16	Lake 77 06/16/2019	2.13±0.15
Lake 22 09/16/2019	2.36±0.16	Lake 77 06/16/2019	2.23±0.16
Lake 23 06/12/2019	2.19±0.15	Lake 77 09/22/2019	2.73±0.19
Lake 23 09/17/2019	2.78±0.19	Lake 77 09/22/2019	2.33±0.16
Lake 25 07/08/2018	2.50±0.17	Lake 78 06/02/2019	2.43±0.17
Lake 25 07/29/2018	2.38±0.16	Lake 88 06/24/2019	2.15±0.15
Lake 25 06/09/2019	2.41±0.16	Lake 88 08/21/2019	2.24±0.15
Lake 29 06/02/2019	2.15±0.15	Lake 89 07/10/2019	2.23±0.15
Lake 29 08/12/2019	2.08±0.14	Lake 90 07/01/2018	1.99±0.14
Lake 31 06/15/2019	2.43±0.17	Lake 90 08/12/2018	2.60±0.18
Lake 31 08/27/2019	2.15±0.15	Lake 90 09/11/2018	2.25±0.16
Lake 33 08/18/2019	2.45±0.17	Lake 90 06/08/2019	1.82±0.13
Lake 33 09/01/2019	2.91±0.20	Lake 90 08/17/2019	1.93±0.13
Lake 34 06/04/2019	1.82±0.13	Lake 92 08/06/2018	3.92±0.27
Lake 34 06/04/2019	1.80±0.13	Lake 92 08/19/2018	3.79±0.26
Lake 37 06/23/2019	2.62±0.17	Lake 92 09/03/2018	2.38±0.16
Lake 37 08/19/2019	2.32±0.16	Lake 92 06/04/2019	3.99±0.27
Lake 38 06/17/2019	2.19±0.15	Lake 92 06/17/2019	3.43±0.24
Lake 39 07/20/2019	2.66±0.18	Lake 92 08/11/2019	2.62±0.18
Lake 40 06/27/2018	1.83±0.13	Lake 92 09/01/2019	2.11±0.14
Lake 40 07/14/2018	3.88±0.26	Lake 96 08/05/2019	2.27±0.15
Lake 40 08/01/2018	2.07±0.14	Lake 99 08/10/2019	2.01±0.14
Lake 40 08/12/2018	3.25±0.22	Lake 100 06/23/2019	1.83±0.13

Table S20. $\Phi_{app, ^3DOM^*_{TMP}}$ for whole water samples (continued)			
Sample ID	$\Phi_{app, ^3DOM^*_{TMP}}$ ($\times10^{-2}$ mol mol-photons^{-1})	Sample ID	$\Phi_{app, ^3DOM^*_{TMP}}$ ($\times10^{-2}$ mol mol-photons^{-1})
Lake 100 08/18/2019	2.06±0.14	Lake 182 06/18/2019	2.44±0.17
Lake 102 06/16/2019	1.96±0.14	Lake 182 07/01/2019	2.35±0.16
Lake 102 09/22/2019	2.47±0.17	Lake 182 07/08/2019	2.03±0.14
Lake 103 06/09/2019	1.56±0.11	Lake 182 08/12/2019	2.10±0.14
Lake 107 08/15/2019	2.32±0.16	Lake 182 08/12/2019	2.27±0.15
Lake 108 08/17/2019	2.29±0.16	Lake 182 08/27/2019	2.29±0.16
Lake 109 07/08/2019	2.82±0.20	Lake 183 08/25/2019	1.89±0.13
Lake 109 08/18/2019	3.55±0.25	Lake 183 09/10/2019	2.15±0.15
Lake 115 07/08/2019	2.32±0.16	Lake 190 06/17/2019	3.20±0.22
Lake 115 07/08/2019	2.14±0.15	Lake 190 08/19/2019	3.29±0.23
Lake 115 09/09/2019	2.33±0.16	Lake 192 09/25/2019	2.05±0.14
Lake 115 09/09/2019	2.22±0.15	Lake 194 06/26/2018	2.13±0.15
Lake 117 08/22/2019	2.14±0.15	Lake 194 07/09/2018	2.17±0.15
Lake 117 09/05/2019	2.24±0.15	Lake 194 07/23/2018	2.43±0.17
Lake 120 06/10/2019	2.02±0.14	Lake 194 08/07/2018	2.38±0.17
Lake 120 08/19/2019	2.43±0.17	Lake 194 08/20/2018	2.28±0.16
Lake 126 07/08/2018	2.39±0.16	Lake 194 09/09/2018	2.32±0.16
Lake 126 07/21/2018	2.39±0.17	Lake 194 09/22/2019	2.45±0.17
Lake 126 08/17/2019	2.17±0.15	Lake 195 06/03/2019	1.81±0.13
Lake 130 08/25/2019	2.22±0.15	Lake 199 06/19/2018	2.69±0.18
Lake 130 09/22/2019	2.10±0.14	Lake 199 07/03/2018	1.71±0.12
Lake 132 06/10/2019	1.97±0.14	Lake 199 07/18/2018	4.22±0.28
Lake 132 09/03/2019	2.62±0.18	Lake 199 07/31/2018	3.02±0.20
Lake 133 06/04/2019	1.80±0.12	Lake 199 08/14/2018	1.54±0.11
Lake 133 08/19/2019	2.41±0.16	Lake 199 08/28/2018	2.17±0.15
Lake 135 05/27/2019	2.02±0.14	Lake 199 09/11/2018	3.14±0.21
Lake 135 08/31/2019	2.23±0.15	Lake 199 06/23/2019	3.67±0.25
Lake 136 06/08/2019	2.13±0.15	Lake 199 07/08/2019	1.86±0.13
Lake 136 09/20/2019	2.63±0.18	Lake 199 07/30/2019	3.14±0.21
Lake 137 08/18/2019	2.99±0.20	Lake 199 08/18/2019	2.50±0.17
Lake 139 08/06/2018	1.55±0.11	Lake 199 09/15/2019	4.24±0.29
Lake 139 09/19/2018	2.44±0.17	Lake 203 06/16/2019	1.81±0.13
Lake 145 06/06/2019	2.00±0.14	Lake 203 09/22/2019	2.15±0.15
Lake 145 08/15/2019	1.92±0.13	Lake 205 06/08/2019	2.16±0.15
Lake 149 06/10/2019	2.16±0.15	Lake 205 08/06/2019	2.08±0.14
Lake 149 09/21/2019	2.45±0.17	Lake 209 08/18/2019	2.01±0.14
Lake 153 08/25/2019	1.96±0.13	Lake 210 06/02/2019	1.72±0.12
Lake 153 09/08/2019	2.19±0.15	Lake 210 08/18/2019	1.56±0.11
Lake 164 06/23/2019	2.24±0.15	Lake 212 06/16/2018	2.43±0.17
Lake 164 08/27/2019	2.46±0.17	Lake 212 07/14/2018	2.29±0.16
Lake 166 07/14/2019	2.37±0.16	Lake 212 07/28/2018	2.42±0.17
Lake 166 09/29/2019	2.49±0.17	Lake 212 08/12/2018	2.34±0.16
Lake 169 07/22/2019	2.59±0.18	Lake 212 06/17/2019	2.18±0.15
Lake 169 08/18/2019	2.50±0.17	Lake 213 08/18/2019	2.67±0.18
Lake 176 07/15/2018	2.10±0.15	Lake 215 06/09/2019	2.45±0.17
Lake 176 06/15/2019	1.93±0.14	Lake 215 09/15/2019	2.86±0.20
Lake 176 08/19/2019	1.99±0.14	Lake 223 08/19/2019	2.64±0.19
Lake 177 08/19/2019	2.05±0.14	Lake 225 07/28/2019	1.98±0.14
Lake 178 08/06/2019	2.24±0.15	Lake 229 06/29/2019	1.91±0.13
Lake 182 07/17/2018	2.25±0.15	Lake 230 06/08/2019	2.44±0.17
Lake 182 07/31/2018	2.49±0.17	Lake 231 06/04/2019	2.10±0.15
Lake 182 08/14/2018	3.52±0.24	Lake 234 06/20/2018	3.35±0.23
Lake 182 09/11/2018	3.82±0.27	Lake 234 07/15/2018	3.24±0.22
Lake 182 06/18/2019	3.53±0.24	Lake 234 06/09/2019	1.97±0.14

**Table S20.** $\Phi_{app,\ ^3DOM^*_{TMP}}$ for whole water samples (continued)			
Sample ID	$\Phi_{app,\ ^3DOM^*_{TMP}}$ ($\times10^{-2}$ mol mol-photons^{-1})	Sample ID	$\Phi_{app,\ ^3DOM^*_{TMP}}$ ($\times10^{-2}$ mol mol-photons^{-1})
Lake 234 08/12/2019	1.97±0.14	Lake 256 07/05/2018	3.11±0.21
Lake 235 07/14/2019	1.99±0.14	Lake 256 07/16/2018	2.74±0.19
Lake 236 07/14/2019	2.70±0.19	Lake 256 07/31/2018	2.50±0.17
Lake 236 09/15/2019	2.23±0.15	Lake 256 08/15/2018	2.78±0.19
Lake 238 08/12/2019	2.49±0.17	Lake 256 08/29/2018	2.62±0.18
Lake 239 06/09/2019	2.39±0.16	Lake 256 09/14/2018	2.42±0.16
Lake 239 08/18/2019	2.53±0.17	Lake 256 09/28/2018	2.53±0.17
Lake 245 09/22/2019	2.39±0.16	Lake 256 06/18/2019	1.73±0.12
Lake 246 08/05/2019	2.36±0.16	Lake 256 06/18/2019	2.33±0.16
Lake 247 06/08/2019	2.04±0.14	Lake 256 08/13/2019	2.39±0.16
Lake 247 09/20/2019	2.25±0.15	Lake 257 08/24/2019	2.42±0.17
Lake 248 08/18/2019	2.07±0.15	Lake 258 06/16/2019	2.27±0.16
Lake 249 07/08/2019	2.02±0.14	Lake 258 08/11/2019	2.35±0.16
Lake 249 09/01/2019	2.03±0.14	Lake 259 09/01/2019	4.08±0.29
Lake 250 07/08/2019	2.62±0.18	Lake 260 06/01/2019	1.96±0.14
Lake 250 08/18/2019	1.74±0.12	Lake 260 08/12/2019	2.47±0.17
Lake 251 08/27/2019	2.48±0.17	Lake 261 06/17/2019	2.66±0.19
Lake 251 09/17/2019	2.74±0.19	Lake 261 08/12/2019	2.35±0.16
Lake 253 07/02/2019	2.40±0.16	Lake 262 06/17/2019	1.83±0.13
Lake 253 08/25/2019	3.66±0.25	Lake 262 09/21/2019	1.78±0.12
Lake 253 09/15/2019	2.27±0.15		

Table S21. $\Phi_{app,\ ^3DOM^*_{TMP}}$ for bloom lysates

Sample ID	Bloom ID	OD_{680} (A.U.)	$\Phi_{app,\ ^3DOM^*_{TMP}}$ ($\times10^{-2}$ mol mol-photons^{-1})	Sample ID	Bloom ID	OD_{680} (A.U.)	$\Phi_{app,\ ^3DOM^*_{TMP}}$ ($\times10^{-2}$ mol mol-photons^{-1})
Lake 261 09/04/2021	A	0.379±0.009	1.00±0.07				
Lake 238 09/07/2021	B	0.349±0.018	1.02±0.06				
Lake 147 08/30/2021	C	0.370±0.019	1.07±0.08				
Lake 138 09/07/2021	D	0.417±0.026	1.05±0.07				
Lake 33 09/04/2021	E	0.368±0.018	0.96±0.08				
Lake 37 08/20/2021	F	0.267±0.013	0.87±0.01				
Lake 38 08/29/2021	G	0.430±0.022	1.21±0.06				
Lake 40 09/16/2021	H	0.392±0.020	1.22±0.05				
Lake 78 08/22/2021	I	0.410±0.020	1.21±0.06				
Lake 82 08/17/2021	J	0.475±0.024	1.29±0.04				
Lake 93 08/29/2021	K	0.245±0.012	0.89±0.10				
Lake 221 09/06/2021	L	0.542±0.027	1.32±0.11				
Lake 256 10/12/2021	Otisco	0.026±0.001	1.75±0.01	SRNOM	-	-	2.68±0.14
Lysate 25% + Otisco 75%	A	-	1.34±0.03	Lysate 25% + SRNOM 75%	A	-	1.99±0.15
Lysate 50% + Otisco 50%	A	-	1.14±0.11	Lysate 50% + SRNOM 50%	A	-	1.57±0.16
Lysate 75% + Otisco 25%	A	-	1.00±0.05	Lysate 75% + SRNOM 25%	A	-	1.18±0.13
Lysate 25% + Otisco 75%	B	-	1.34±0.04	Lysate 25% + SRNOM 75%	B	-	1.91±0.09
Lysate 50% + Otisco 50%	B	-	1.12±0.04	Lysate 50% + SRNOM 50%	B	-	1.46±0.03
Lysate 75% + Otisco 25%	B	-	0.94±0.05	Lysate 75% + SRNOM 25%	B	-	1.08±0.04
Lysate 25% + Otisco 75%	C	-	1.37±0.04	Lysate 25% + SRNOM 75%	C	-	1.95±0.11
Lysate 50% + Otisco 50%	C	-	1.18±0.04	Lysate 50% + SRNOM 50%	C	-	1.53±0.08
Lysate 75% + Otisco 25%	C	-	1.05±0.03	Lysate 75% + SRNOM 25%	C	-	1.16±0.08
Lysate 25% + Otisco 75%	D	-	1.40±0.05	Lysate 25% + SRNOM 75%	D	-	1.99±0.14
Lysate 50% + Otisco 50%	D	-	1.22±0.03	Lysate 50% + SRNOM 50%	D	-	1.60±0.05
Lysate 75% + Otisco 25%	D	-	1.10±0.06	Lysate 75% + SRNOM 25%	D	-	1.24±0.08
Lysate 25% + Otisco 75%	E	-	1.38±0.06	Lysate 25% + SRNOM 75%	E	-	1.94±0.09
Lysate 50% + Otisco 50%	E	-	1.19±0.05	Lysate 50% + SRNOM 50%	E	-	1.54±0.07
Lysate 75% + Otisco 25%	E	-	1.07±0.02	Lysate 75% + SRNOM 25%	E	-	1.17±0.04
Lysate 25% + Otisco 75%	F	-	1.41±0.05	Lysate 25% + SRNOM 75%	F	-	1.98±0.11
Lysate 50% + Otisco 50%	F	-	1.23±0.05	Lysate 50% + SRNOM 50%	F	-	1.58±0.09
Lysate 75% + Otisco 25%	F	-	1.09±0.06	Lysate 75% + SRNOM 25%	F	-	1.22±0.08

Table S22. $\Phi_{app,\ ^3DOM^*_{TMP}}$ for bloom supernatants

Sample ID	Bloom ID	OD_{680} (A.U.)	$\Phi_{app,\ ^3DOM^*_{TMP}}$ ($\times 10^{-2}$ mol mol-photons^{-1})	Sample ID	Bloom ID	OD_{680} (A.U.)	$\Phi_{app,\ ^3DOM^*_{TMP}}$ ($\times 10^{-2}$ mol mol-photons^{-1})
Lake 261 09/04/2021	A	0.151±0.003	2.58±0.13	Lake 33 09/04/2021	E	0.368±0.018	3.83±0.48
	A	0.182±0.006	3.06±0.10	Lake 37 08/20/2021	F	0.267±0.013	3.49±0.01
	A	0.215±0.008	3.47±0.12	Lake 38 08/29/2021	G	0.430±0.022	4.86±0.31
	A	0.259±0.009	3.83±0.19	Lake 40 09/16/2021	H	0.392±0.020	4.89±0.15
	A	0.306±0.009	4.02±0.17	Lake 78 08/22/2021	I	0.410±0.020	4.71±0.21
	A	0.347±0.009	4.12±0.22	Lake 82 08/17/2021	J	0.475±0.024	5.17±0.07
	A	0.370±0.009	4.18±0.18	Lake 93 08/29/2021	K	0.245±0.012	3.51±0.03
	A	0.376±0.009	4.20±0.21	Lake 221 09/06/2021	L	0.542±0.027	6.58±0.44
	A	0.379±0.009	4.21±0.27				
Lake 238 09/07/2021	B	0.138±0.003	2.54±0.03	Lake 256 10/12/2021	Otisco	0.008±0.001	1.87±0.04
	B	0.159±0.006	3.04±0.03		Otisco	0.013±0.001	1.85±0.02
	B	0.195±0.009	3.50±0.04		Otisco	0.016±0.001	1.84±0.01
	B	0.242±0.009	3.88±0.16		Otisco	0.018±0.002	1.83±0.03
	B	0.283±0.014	4.10±0.03		Otisco	0.020±0.002	1.81±0.01
	B	0.321±0.015	4.22±0.09		Otisco	0.022±0.002	1.79±0.01
	B	0.338±0.017	4.30±0.06		Otisco	0.023±0.002	1.77±0.03
	B	0.345±0.017	4.34±0.09		Otisco	0.024±0.002	1.76±0.01
	B	0.349±0.018	4.35±0.01		Otisco	0.025±0.002	1.75±0.02
Lake 147 08/30/2021	C	0.078±0.009	2.95±0.18	Lake 256 10/12/2021	Otisco	0.009±0.001	1.87±0.05
	C	0.150±0.011	3.81±0.20		Otisco	0.014±0.001	1.86±0.02
	C	0.208±0.016	4.44±0.20		Otisco	0.017±0.001	1.84±0.02
	C	0.264±0.018	4.78±0.16		Otisco	0.019±0.001	1.83±0.02
	C	0.310±0.020	5.00±0.22		Otisco	0.021±0.002	1.81±0.03
	C	0.340±0.019	5.10±0.23		Otisco	0.023±0.002	1.79±0.04
	C	0.354±0.019	5.18±0.29		Otisco	0.024±0.002	1.77±0.04
	C	0.366±0.019	5.21±0.29		Otisco	0.025±0.002	1.76±0.01
	C	0.370±0.019	5.22±0.27		Otisco	0.026±0.002	1.75±0.01
Lake 138 09/07/2021	D	0.107±0.011	2.86±0.14				
	D	0.202±0.024	3.72±0.09				
	D	0.280±0.029	4.36±0.13				
	D	0.326±0.028	4.72±0.11				
	D	0.365±0.023	4.90±0.17				
	D	0.389±0.025	5.00±0.15				
	D	0.402±0.024	5.06±0.17				
	D	0.412±0.026	5.08±0.14				
	D	0.417±0.026	5.09±0.18				

**Table S22.** $\Phi_{app,\ ^3DOM^*_{TMP}}$ for bloom supernatants (continued)						
Sample ID	Bloom ID	OD_{680} (A.U.)	$\Phi_{app,\ ^3DOM^*_{TMP},\ high\text{-}energy}$ ($\times10^{-2}$ mol mol-photons^{-1})	$\Phi_{app,\ ^3DOM^*_{TMP},\ low\text{-}energy}$ ($\times10^{-2}$ mol mol-photons^{-1})	$\%\Phi_{app,\ ^3DOM^*_{TMP},\ high\text{-}energy}$	$\%\Phi_{app,\ ^3DOM^*_{TMP},\ low\text{-}energy}$
Lake 261 09/04/2021	A	0.379±0.009	2.36±0.15	1.85±0.13	43.9±0.1	56.1±0.1
Lake 238 09/07/2021	B	0.349±0.018	2.47±0.05	1.88±0.04	43.2±1.0	56.8±1.0
Lake 147 08/30/2021	C	0.370±0.019	3.19±0.15	2.03±0.12	38.9±0.2	61.1±0.2
Lake 138 09/07/2021	D	0.417±0.026	3.03±0.06	2.06±0.13	40.4±1.0	59.6±1.0
Lake 33 09/04/2021	E	0.368±0.018	2.10±0.37	1.73±0.11	54.6±2.9	45.4±2.9
Lake 37 08/20/2021	F	0.267±0.013	1.85±0.01	1.65±0.01	52.9±0.3	47.1±0.3
Lake 38 08/29/2021	G	0.430±0.022	2.93±0.28	1.93±0.03	60.2±1.8	39.8±1.8
Lake 40 09/16/2021	H	0.392±0.020	2.84±0.03	2.05±0.11	58.1±1.1	41.9±1.1
Lake 78 08/22/2021	I	0.410±0.020	2.77±0.09	1.94±0.12	58.8±0.7	41.2±0.7
Lake 82 08/17/2021	J	0.475±0.024	3.06±0.01	2.11±0.07	59.2±0.8	40.8±0.8
Lake 93 08/29/2021	K	0.245±0.012	1.84±0.02	1.67±0.05	52.4±1.0	47.6±1.0
Lake 221 09/06/2021	L	0.542±0.027	4.19±0.26	2.39±0.17	63.7±0.2	36.3±0.2
Lake 256 10/12/2021	Otisco	0.026±0.001	0.53±0.01	1.22±0.01	30.3±0.3	69.7±0.3

Table S23. $\Phi_{app,\ ^{3}DOM^{*}_{TMP}}$ for bloom supernatants minus the contribution from Otisco Lake water

Sample ID	Bloom ID	ΔOD_{680} (A.U.)	$\Delta\Phi_{app,\ ^{3}DOM^{*}_{TMP}}$ ($\times10^{-2}$ mol mol-photons^{-1})	Sample ID	Bloom ID	ΔOD_{680} (A.U.)	$\Delta\Phi_{app,\ ^{3}DOM^{*}_{TMP}}$ ($\times10^{-2}$ mol mol-photons^{-1})
Lake 261 09/04/2021	A	0.143±0.007	0.71±0.09	Lake 33 09/04/2021	E	0.343±0.017	2.08±0.47
	A	0.168±0.008	1.21±0.07	Lake 37 08/20/2021	F	0.242±0.012	1.74±0.01
	A	0.199±0.010	1.63±0.12	Lake 38 08/29/2021	G	0.405±0.020	3.11±0.30
	A	0.241±0.012	2.00±0.21	Lake 40 09/16/2021	H	0.367±0.018	3.14±0.13
	A	0.286±0.014	2.21±0.15	Lake 78 08/22/2021	I	0.384±0.019	2.96±0.19
	A	0.325±0.016	2.33±0.19	Lake 82 08/17/2021	J	0.450±0.022	3.42±0.05
	A	0.346±0.017	2.40±0.15	Lake 93 08/29/2021	K	0.219±0.011	1.76±0.05
	A	0.352±0.018	2.44±0.20	Lake 221 09/06/2021	L	0.517±0.026	4.83±0.42
	A	0.354±0.018	2.46±0.26				
Lake 238 09/07/2021	B	0.130±0.006	0.67±0.01				
	B	0.146±0.007	1.18±0.01				
	B	0.179±0.009	1.65±0.04				
	B	0.224±0.011	2.05±0.18				
	B	0.263±0.013	2.29±0.04				
	B	0.299±0.015	2.43±0.12				
	B	0.315±0.016	2.53±0.09				
	B	0.320±0.016	2.58±0.09				
	B	0.323±0.016	2.60±0.01				
Lake 147 08/30/2021	C	0.070±0.003	1.08±0.14				
	C	0.137±0.007	1.96±0.18				
	C	0.192±0.010	2.59±0.20				
	C	0.246±0.012	2.95±0.14				
	C	0.290±0.015	3.19±0.21				
	C	0.318±0.016	3.31±0.21				
	C	0.331±0.017	3.40±0.25				
	C	0.341±0.017	3.45±0.29				
	C	0.345±0.017	3.47±0.26				
Lake 138 09/07/2021	D	0.099±0.005	1.00±0.10				
	D	0.189±0.009	1.87±0.07				
	D	0.264±0.013	2.52±0.13				
	D	0.308±0.015	2.89±0.08				
	D	0.345±0.017	3.09±0.16				
	D	0.367±0.018	3.21±0.13				
	D	0.379±0.019	3.28±0.14				
	D	0.388±0.019	3.32±0.13				
	D	0.391±0.020	3.34±0.17				

Table S23. $\Phi_{app,\ ^3DOM^*_{TMP}}$ for bloom supernatants minus the contribution from Otisco Lake water (continued)

Sample ID	Bloom ID	ΔOD_{680} (A.U.)	$\Delta\Phi_{app,\ ^3DOM^*_{TMP},\ high\text{-}energy}$ ($\times10^{-2}$ mol mol-photons^{-1})	$\Delta\Phi_{app,\ ^3DOM^*_{TMP},\ low\text{-}energy}$ ($\times10^{-2}$ mol mol-photons^{-1})	$\%\Delta\Phi_{app,\ ^3DOM^*_{TMP},\ high\text{-}energy}$	$\%\Delta\Phi_{app,\ ^3DOM^*_{TMP},\ low\text{-}energy}$
Lake 261 09/04/2021	A	0.354±0.018	1.83±0.14	0.63±0.12	67.1±1.3	32.9±1.3
Lake 238 09/07/2021	B	0.323±0.016	1.94±0.04	0.66±0.04	66.0±2.0	34.0±2.0
Lake 147 08/30/2021	C	0.345±0.017	2.66±0.15	0.81±0.11	66.7±1.6	33.3±1.6
Lake 138 09/07/2021	D	0.391±0.020	2.50±0.05	0.84±0.12	66.6±1.3	33.4±1.3
Lake 33 09/04/2021	E	0.343±0.017	1.57±0.37	0.51±0.10	67.4±0.6	32.6±0.6
Lake 37 08/20/2021	F	0.242±0.012	1.31±0.01	0.43±0.01	67.7±0.7	32.3±0.7
Lake 38 08/29/2021	G	0.405±0.020	2.40±0.27	0.71±0.03	68.4±1.6	31.6±1.6
Lake 40 09/16/2021	H	0.367±0.018	2.31±0.02	0.83±0.11	68.6±1.8	31.4±1.8
Lake 78 08/22/2021	I	0.384±0.019	2.24±0.08	0.73±0.11	69.6±1.0	30.4±1.0
Lake 82 08/17/2021	J	0.450±0.022	2.53±0.01	0.89±0.06	67.6±1.4	32.4±1.4
Lake 93 08/29/2021	K	0.219±0.011	1.31±0.01	0.45±0.06	67.2±2.8	32.8±2.8
Lake 221 09/06/2021	L	0.517±0.026	3.66±0.25	1.17±0.17	68.1±1.7	31.9±1.7

Table S24. f_{TMP} for whole water samples			
Sample ID	f_{TMP} (L mol-photons^{-1})	Sample ID	f_{TMP} (L mol-photons^{-1})
Lake 2 06/08/2019	50.8±0.1	Lake 40 08/29/2018	84.8±0.1
Lake 2 08/31/2019	55.9±0.1	Lake 40 06/19/2019	67.7±0.3
Lake 6 06/18/2019	52.5±0.1	Lake 41 07/07/2018	88.2±0.3
Lake 6 08/28/2019	85.8±0.1	Lake 41 07/17/2018	61.4±0.2
Lake 8 08/05/2019	53.2±0.1	Lake 41 07/29/2018	87.9±0.2
Lake 12 06/08/2019	53.6±0.1	Lake 41 08/19/2018	92.1±0.3
Lake 12 09/01/2019	66.4±0.2	Lake 45 08/18/2019	58.9±0.3
Lake 13 07/08/2018	74.6±0.1	Lake 49 07/08/2019	63.6±0.2
Lake 13 07/22/2018	62.6±0.1	Lake 50 06/10/2019	57.7±0.2
Lake 13 08/06/2018	72.4±0.2	Lake 52 06/30/2019	86.0±0.4
Lake 13 08/21/2018	75.3±0.1	Lake 53 06/09/2019	50.8±0.1
Lake 13 09/02/2018	74.6±0.2	Lake 57 06/29/2019	61.2±0.2
Lake 13 06/10/2019	42.4±0.1	Lake 57 09/08/2019	62.8±0.2
Lake 13 08/31/2019	58.4±0.1	Lake 58 09/10/2019	90.8±0.1
Lake 17 07/01/2018	55.5±0.2	Lake 61 07/09/2018	53.0±0.2
Lake 17 07/15/2018	52.0±0.1	Lake 61 07/19/2018	50.0±0.1
Lake 17 07/28/2018	55.1±0.2	Lake 61 08/19/2018	56.7±0.2
Lake 17 08/12/2018	68.5±0.2	Lake 61 09/02/2018	97.7±0.3
Lake 17 08/26/2018	66.6±0.2	Lake 61 09/22/2018	55.9±0.1
Lake 17 07/15/2019	51.5±0.2	Lake 61 07/21/2019	67.2±0.2
Lake 17 08/12/2019	54.1±0.2	Lake 61 07/23/2019	81.1±0.2
Lake 18 08/05/2018	61.8±0.2	Lake 66 08/16/2019	81.1±0.1
Lake 18 08/18/2018	59.5±0.3	Lake 68 09/17/2019	65.9±0.2
Lake 18 09/09/2018	58.0±0.2	Lake 69 06/09/2019	52.5±0.1
Lake 18 09/30/2018	62.8±0.2	Lake 69 08/19/2019	49.9±0.1
Lake 18 06/15/2019	53.4±0.2	Lake 72 07/12/2019	51.2±0.1
Lake 18 08/12/2019	52.9±0.2	Lake 73 07/20/2019	56.3±0.2
Lake 21 07/02/2019	56.8±0.1	Lake 73 08/24/2019	68.5±0.2
Lake 21 08/13/2019	56.6±0.2	Lake 74 06/08/2019	56.6±0.1
Lake 22 06/23/2019	58.3±0.2	Lake 74 08/18/2019	56.5±0.1
Lake 22 06/23/2019	57.2±0.1	Lake 75 06/02/2019	57.6±0.2
Lake 22 09/16/2019	57.9±0.2	Lake 77 06/16/2019	54.3±0.1
Lake 22 09/16/2019	60.4±0.2	Lake 77 06/16/2019	57.1±0.1
Lake 23 06/12/2019	55.9±0.1	Lake 77 09/22/2019	69.6±0.1
Lake 23 09/17/2019	70.9±0.1	Lake 77 09/22/2019	59.4±0.1
Lake 25 07/08/2018	63.9±0.2	Lake 78 06/02/2019	62.0±0.1
Lake 25 07/29/2018	60.8±0.2	Lake 88 06/24/2019	54.9±0.2
Lake 25 06/09/2019	61.5±0.2	Lake 88 08/21/2019	57.3±0.2
Lake 29 06/02/2019	55.0±0.2	Lake 89 07/10/2019	57.1±0.2
Lake 29 08/12/2019	53.2±0.2	Lake 90 07/01/2018	50.9±0.2
Lake 31 06/15/2019	62.1±0.1	Lake 90 08/12/2018	66.4±0.1
Lake 31 08/27/2019	54.9±0.1	Lake 90 09/11/2018	57.5±0.1
Lake 33 08/18/2019	62.5±0.2	Lake 90 06/08/2019	46.5±0.1
Lake 33 09/01/2019	74.5±0.2	Lake 90 08/17/2019	49.2±0.1
Lake 34 06/04/2019	46.6±0.1	Lake 92 08/06/2018	100.1±0.3
Lake 34 06/04/2019	45.9±0.1	Lake 92 08/19/2018	96.7±0.3
Lake 37 06/23/2019	66.9±0.3	Lake 92 09/03/2018	60.7±0.2
Lake 37 08/19/2019	59.2±0.3	Lake 92 06/04/2019	101.8±0.4
Lake 38 06/17/2019	55.9±0.2	Lake 92 06/17/2019	87.7±0.3
Lake 39 07/20/2019	67.9±0.2	Lake 92 08/11/2019	66.9±0.2
Lake 40 06/27/2018	46.8±0.1	Lake 92 09/01/2019	53.8±0.2
Lake 40 07/14/2018	99.1±0.4	Lake 96 08/05/2019	58.1±0.2
Lake 40 08/01/2018	52.8±0.2	Lake 99 08/10/2019	51.4±0.1
Lake 40 08/12/2018	83.1±0.2	Lake 100 06/23/2019	46.6±0.1

**Table S24.** f_{TMP} for whole water samples (continued)			
Sample ID	f_{TMP} (L mol-photons^{-1})	Sample ID	f_{TMP} (L mol-photons^{-1})
Lake 100 08/18/2019	52.6±0.1	Lake 182 06/18/2019	62.2±0.2
Lake 102 06/16/2019	50.2±0.1	Lake 182 07/01/2019	60.0±0.1
Lake 102 09/22/2019	63.2±0.2	Lake 182 07/08/2019	52.0±0.2
Lake 103 06/09/2019	39.8±0.1	Lake 182 08/12/2019	53.6±0.2
Lake 107 08/15/2019	59.4±0.2	Lake 182 08/12/2019	58.0±0.2
Lake 108 08/17/2019	58.5±0.2	Lake 182 08/27/2019	58.5±0.2
Lake 109 07/08/2019	72.0±0.1	Lake 183 08/25/2019	48.4±0.2
Lake 109 08/18/2019	90.7±0.1	Lake 183 09/10/2019	55.0±0.2
Lake 115 07/08/2019	59.2±0.2	Lake 190 06/17/2019	81.7±0.1
Lake 115 07/08/2019	54.6±0.2	Lake 190 08/19/2019	84.0±0.1
Lake 115 09/09/2019	59.4±0.2	Lake 192 09/25/2019	52.3±0.1
Lake 115 09/09/2019	56.8±0.2	Lake 194 06/26/2018	54.4±0.2
Lake 117 08/22/2019	54.7±0.1	Lake 194 07/09/2018	55.3±0.1
Lake 117 09/05/2019	57.2±0.2	Lake 194 07/23/2018	62.0±0.1
Lake 120 06/10/2019	51.7±0.2	Lake 194 08/07/2018	60.7±0.1
Lake 120 08/19/2019	62.0±0.2	Lake 194 08/20/2018	58.3±0.2
Lake 126 07/08/2018	61.2±0.2	Lake 194 09/09/2018	59.3±0.2
Lake 126 07/21/2018	61.1±0.2	Lake 194 09/22/2019	62.5±0.2
Lake 126 08/17/2019	55.5±0.2	Lake 195 06/03/2019	46.1±0.1
Lake 130 08/25/2019	56.7±0.2	Lake 199 06/19/2018	68.8±0.3
Lake 130 09/22/2019	53.7±0.3	Lake 199 07/03/2018	43.7±0.1
Lake 132 06/10/2019	50.4±0.1	Lake 199 07/18/2018	107.8±0.5
Lake 132 09/03/2019	67.0±0.2	Lake 199 07/31/2018	77.1±0.3
Lake 133 06/04/2019	46.0±0.2	Lake 199 08/14/2018	39.5±0.1
Lake 133 08/19/2019	61.6±0.2	Lake 199 08/28/2018	55.5±0.2
Lake 135 05/27/2019	51.6±0.1	Lake 199 09/11/2018	80.1±0.3
Lake 135 08/31/2019	56.9±0.1	Lake 199 06/23/2019	93.7±0.3
Lake 136 06/08/2019	54.5±0.2	Lake 199 07/08/2019	47.5±0.2
Lake 136 09/20/2019	67.2±0.2	Lake 199 07/30/2019	80.1±0.3
Lake 137 08/18/2019	76.3±0.3	Lake 199 08/18/2019	63.9±0.4
Lake 139 08/06/2018	39.6±0.1	Lake 199 09/15/2019	108.2±0.5
Lake 139 09/19/2018	62.3±0.2	Lake 203 06/16/2019	46.3±0.1
Lake 145 06/06/2019	51.2±0.1	Lake 203 09/22/2019	55.0±0.1
Lake 145 08/15/2019	49.1±0.1	Lake 205 06/08/2019	55.1±0.1
Lake 149 06/10/2019	55.3±0.1	Lake 205 08/06/2019	53.1±0.1
Lake 149 09/21/2019	62.7±0.3	Lake 209 08/18/2019	51.2±0.1
Lake 153 08/25/2019	50.0±0.1	Lake 210 06/02/2019	44.0±0.1
Lake 153 09/08/2019	55.9±0.2	Lake 210 08/18/2019	39.9±0.1
Lake 164 06/23/2019	57.3±0.2	Lake 212 06/16/2018	62.2±0.2
Lake 164 08/27/2019	62.8±0.2	Lake 212 07/14/2018	58.6±0.1
Lake 166 07/14/2019	60.6±0.2	Lake 212 07/28/2018	61.8±0.1
Lake 166 09/29/2019	63.6±0.2	Lake 212 08/12/2018	59.8±0.1
Lake 169 07/22/2019	66.2±0.2	Lake 212 06/17/2019	55.7±0.1
Lake 169 08/18/2019	63.9±0.2	Lake 213 08/18/2019	68.3±0.2
Lake 176 07/15/2018	53.8±0.1	Lake 215 06/09/2019	62.7±0.1
Lake 176 06/15/2019	49.4±0.1	Lake 215 09/15/2019	73.1±0.1
Lake 176 08/19/2019	50.9±0.1	Lake 223 08/19/2019	67.5±0.1
Lake 177 08/19/2019	52.3±0.1	Lake 225 07/28/2019	50.5±0.1
Lake 178 08/06/2019	57.2±0.2	Lake 229 06/29/2019	48.8±0.1
Lake 182 07/17/2018	57.6±0.3	Lake 230 06/08/2019	62.4±0.2
Lake 182 07/31/2018	63.6±0.3	Lake 231 06/04/2019	53.7±0.1
Lake 182 08/14/2018	89.8±0.2	Lake 234 06/20/2018	85.5±0.2
Lake 182 09/11/2018	97.7±0.1	Lake 234 07/15/2018	82.9±0.2
Lake 182 06/18/2019	90.1±0.2	Lake 234 06/09/2019	50.4±0.1

Table S24. f_{TMP} for whole water samples (continued)			
Sample ID	f_{TMP} (L mol-photons^{-1})	Sample ID	f_{TMP} (L mol-photons^{-1})
Lake 234 08/12/2019	50.4±0.2	Lake 256 07/05/2018	79.3±0.2
Lake 235 07/14/2019	50.9±0.1	Lake 256 07/16/2018	70.0±0.3
Lake 236 07/14/2019	69.0±0.2	Lake 256 07/31/2018	63.9±0.2
Lake 236 09/15/2019	57.1±0.2	Lake 256 08/15/2018	71.1±0.2
Lake 238 08/12/2019	63.5±0.1	Lake 256 08/29/2018	66.8±0.2
Lake 239 06/09/2019	61.1±0.2	Lake 256 09/14/2018	61.9±0.2
Lake 239 08/18/2019	64.7±0.2	Lake 256 09/28/2018	64.5±0.3
Lake 245 09/22/2019	61.1±0.2	Lake 256 06/18/2019	44.3±0.1
Lake 246 08/05/2019	60.2±0.1	Lake 256 06/18/2019	59.5±0.1
Lake 247 06/08/2019	52.0±0.1	Lake 256 08/13/2019	60.9±0.2
Lake 247 09/20/2019	57.5±0.2	Lake 257 08/24/2019	61.7±0.1
Lake 248 08/18/2019	52.7±0.1	Lake 258 06/16/2019	57.9±0.1
Lake 249 07/08/2019	51.7±0.1	Lake 258 08/11/2019	60.0±0.1
Lake 249 09/01/2019	51.9±0.1	Lake 259 09/01/2019	104.2±0.1
Lake 250 07/08/2019	67.0±0.2	Lake 260 06/01/2019	50.2±0.1
Lake 250 08/18/2019	44.3±0.1	Lake 260 08/12/2019	63.0±0.1
Lake 251 08/27/2019	63.4±0.1	Lake 261 06/17/2019	68.0±0.1
Lake 251 09/17/2019	70.0±0.1	Lake 261 08/12/2019	60.1±0.2
Lake 253 07/02/2019	61.3±0.2	Lake 262 06/17/2019	46.6±0.1
Lake 253 08/25/2019	93.6±0.3	Lake 262 09/21/2019	45.6±0.1
Lake 253 09/15/2019	58.0±0.2		

Table S25. f_{TMP} for bloom lysates							
Sample ID	Bloom ID	OD_{680} (A.U.)	f_{TMP} (L mol-photons^{-1})	Sample ID	Bloom ID	OD_{680} (A.U.)	f_{TMP} (L mol-photons^{-1})
Lake 261 09/04/2021	A	0.379±0.009	14.6±1.6				
Lake 238 09/07/2021	B	0.349±0.018	15.2±0.1				
Lake 147 08/30/2021	C	0.370±0.019	15.3±2.9				
Lake 138 09/07/2021	D	0.417±0.026	16.0±2.2				
Lake 33 09/04/2021	E	0.368±0.018	13.6±1.1				
Lake 37 08/20/2021	F	0.267±0.013	11.7±0.2				
Lake 38 08/29/2021	G	0.430±0.022	18.4±2.2				
Lake 40 09/16/2021	H	0.392±0.020	19.2±1.2				
Lake 78 08/22/2021	I	0.410±0.020	17.9±2.6				
Lake 82 08/17/2021	J	0.475±0.024	20.0±0.6				
Lake 93 08/29/2021	K	0.245±0.012	12.4±2.0				
Lake 221 09/06/2021	L	0.542±0.027	21.1±2.0				
Lake 256 10/12/2021	Otisco	0.026±0.001	30.3±0.1	SRNOM	-	-	52.8±4.3
Lysate 25% + Otisco 75%	A	-	22.2±0.9	Lysate 25% + SRNOM 75%	A	-	36.6±3.8
Lysate 50% + Otisco 50%	A	-	18.1±2.2	Lysate 50% + SRNOM 50%	A	-	26.6±3.2
Lysate 75% + Otisco 25%	A	-	15.1±0.9	Lysate 75% + SRNOM 25%	A	-	18.4±2.3
Lysate 25% + Otisco 75%	B	-	21.9±1.0	Lysate 25% + SRNOM 75%	B	-	34.7±3.1
Lysate 50% + Otisco 50%	B	-	17.2±0.2	Lysate 50% + SRNOM 50%	B	-	24.3±1.8
Lysate 75% + Otisco 25%	B	-	13.6±0.2	Lysate 75% + SRNOM 25%	B	-	16.3±1.5
Lysate 25% + Otisco 75%	C	-	22.8±2.7	Lysate 25% + SRNOM 75%	C	-	36.1±3.8
Lysate 50% + Otisco 50%	C	-	18.8±2.0	Lysate 50% + SRNOM 50%	C	-	26.3±2.9
Lysate 75% + Otisco 25%	C	-	16.0±1.4	Lysate 75% + SRNOM 25%	C	-	18.5±2.3
Lysate 25% + Otisco 75%	D	-	23.1±0.2	Lysate 25% + SRNOM 75%	D	-	36.5±2.9
Lysate 50% + Otisco 50%	D	-	19.3±2.2	Lysate 50% + SRNOM 50%	D	-	27.1±0.6
Lysate 75% + Otisco 25%	D	-	16.6±2.6	Lysate 75% + SRNOM 25%	D	-	19.4±1.4
Lysate 25% + Otisco 75%	E	-	23.1±2.6	Lysate 25% + SRNOM 75%	E	-	36.0±3.2
Lysate 50% + Otisco 50%	E	-	19.2±1.8	Lysate 50% + SRNOM 50%	E	-	26.8±2.7
Lysate 75% + Otisco 25%	E	-	16.5±1.1	Lysate 75% + SRNOM 25%	E	-	18.9±0.6
Lysate 25% + Otisco 75%	F	-	23.7±3.1	Lysate 25% + SRNOM 75%	F	-	36.8±3.6
Lysate 50% + Otisco 50%	F	-	20.0±2.6	Lysate 50% + SRNOM 50%	F	-	27.6±2.9
Lysate 75% + Otisco 25%	F	-	17.2±2.3	Lysate 75% + SRNOM 25%	F	-	20.0±2.5

Table S26. f_{TMP} for bloom supernatants

Sample ID	Bloom ID	OD_{680} (A.U.)	f_{TMP} (L mol-photons^{-1})	Sample ID	Bloom ID	OD_{680} (A.U.)	f_{TMP} (L mol-photons^{-1})
Lake 261 09/04/2021	A	0.151±0.003	62.4±0.4	Lake 33 09/04/2021	E	0.368±0.018	90.8±5.1
	A	0.182±0.006	74.2±1.8	Lake 37 08/20/2021	F	0.267±0.013	78.8±3.0
	A	0.215±0.008	84.0±1.4	Lake 38 08/29/2021	G	0.430±0.022	133.3±2.9
	A	0.259±0.009	93.0±8.7	Lake 40 09/16/2021	H	0.392±0.020	136.6±6.3
	A	0.306±0.009	97.4±0.2	Lake 78 08/22/2021	I	0.410±0.020	125.4±0.9
	A	0.347±0.009	99.8±1.9	Lake 82 08/17/2021	J	0.475±0.024	150.1±1.6
	A	0.370±0.009	101.2±1.2	Lake 93 08/29/2021	K	0.245±0.012	77.8±2.1
	A	0.376±0.009	101.9±1.7	Lake 221 09/06/2021	L	0.542±0.027	202.1±1.2
	A	0.379±0.009	102.0±3.4				
Lake 238 09/07/2021	B	0.138±0.003	63.7±3.8	Lake 256 10/12/2021	Otisco	0.008±0.001	32.3±0.6
	B	0.159±0.006	76.3±4.4		Otisco	0.013±0.001	32.1±0.2
	B	0.195±0.009	87.8±3.4		Otisco	0.016±0.001	31.8±0.3
	B	0.242±0.009	97.3±1.2		Otisco	0.018±0.002	31.6±0.3
	B	0.283±0.014	102.9±4.9		Otisco	0.020±0.002	31.3±0.4
	B	0.321±0.015	106.0±3.9		Otisco	0.022±0.002	31.0±0.1
	B	0.338±0.017	108.0±4.9		Otisco	0.023±0.002	30.7±0.1
	B	0.345±0.017	109.1±4.3		Otisco	0.024±0.002	30.5±0.3
	B	0.349±0.018	109.2±6.8		Otisco	0.025±0.002	30.3±0.1
Lake 147 08/30/2021	C	0.078±0.009	78.6±0.6	Lake 256 10/12/2021	Otisco	0.009±0.001	32.3±0.7
	C	0.150±0.011	101.6±1.0		Otisco	0.014±0.001	32.1±0.2
	C	0.208±0.016	118.3±0.4		Otisco	0.017±0.001	31.9±0.1
	C	0.264±0.018	127.5±1.3		Otisco	0.019±0.001	31.6±0.2
	C	0.310±0.020	133.3±0.4		Otisco	0.021±0.002	31.3±0.1
	C	0.340±0.019	136.1±1.0		Otisco	0.023±0.002	31.0±0.4
	C	0.354±0.019	138.0±2.5		Otisco	0.024±0.002	30.7±0.2
	C	0.366±0.019	138.8±2.7		Otisco	0.025±0.002	30.5±0.3
	C	0.370±0.019	139.2±2.2		Otisco	0.026±0.002	30.3±0.2
Lake 138 09/07/2021	D	0.107±0.011	74.3±1.1				
	D	0.202±0.024	96.7±3.7				
	D	0.280±0.029	113.2±3.3				
	D	0.326±0.028	122.7±4.0				
	D	0.365±0.023	127.3±2.1				
	D	0.389±0.025	129.9±2.5				
	D	0.402±0.024	131.3±2.1				
	D	0.412±0.026	132.0±2.9				
	D	0.417±0.026	132.2±1.8				

Table S27. f_{TMP} for bloom supernatants minus the contribution from Otisco Lake water

Sample ID	Bloom ID	ΔOD_{680} (A.U.)	Δf_{TMP} (L mol-photons^{-1})	Sample ID	Bloom ID	ΔOD_{680} (A.U.)	Δf_{TMP} (L mol-photons^{-1})
Lake 261 09/04/2021	A	0.143±0.007	30.1±1.0	Lake 33 09/04/2021	E	0.343±0.017	60.5±5.2
	A	0.168±0.008	42.1±2.0	Lake 37 08/20/2021	F	0.242±0.012	48.5±2.9
	A	0.199±0.010	52.2±1.3	Lake 38 08/29/2021	G	0.405±0.020	103.1±3.1
	A	0.241±0.012	61.4±8.9	Lake 40 09/16/2021	H	0.367±0.018	106.4±6.2
	A	0.286±0.014	66.1±0.4	Lake 78 08/22/2021	I	0.384±0.019	95.1±0.8
	A	0.325±0.016	68.8±1.8	Lake 82 08/17/2021	J	0.450±0.022	119.8±1.4
	A	0.346±0.017	70.5±1.0	Lake 93 08/29/2021	K	0.219±0.011	47.6±1.9
	A	0.352±0.018	71.4±2.0	Lake 221 09/06/2021	L	0.517±0.026	171.8±1.0
	A	0.354±0.018	71.7±3.6				
Lake 238 09/07/2021	B	0.130±0.006	31.5±3.2				
	B	0.146±0.007	44.3±4.2				
	B	0.179±0.009	55.9±3.5				
	B	0.224±0.011	65.7±1.0				
	B	0.263±0.013	71.6±5.0				
	B	0.299±0.015	75.1±3.7				
	B	0.315±0.016	77.3±4.7				
	B	0.320±0.016	78.5±4.6				
	B	0.323±0.016	78.9±7.0				
Lake 147 08/30/2021	C	0.070±0.003	46.3±0.1				
	C	0.137±0.007	69.5±0.2				
	C	0.192±0.010	86.4±0.3				
	C	0.246±0.012	95.9±1.6				
	C	0.290±0.015	102.0±0.5				
	C	0.318±0.016	105.1±0.9				
	C	0.331±0.017	107.3±2.3				
	C	0.341±0.017	108.3±2.9				
	C	0.345±0.017	108.9±2.3				
Lake 138 09/07/2021	D	0.099±0.005	42.0±1.8				
	D	0.189±0.009	64.6±3.9				
	D	0.264±0.013	81.4±3.2				
	D	0.308±0.015	91.1±4.2				
	D	0.345±0.017	96.1±2.0				
	D	0.367±0.018	98.9±2.7				
	D	0.379±0.019	100.6±2.2				
	D	0.388±0.019	101.4±2.6				
	D	0.391±0.020	101.9±1.6				

10. ***trans,trans*-2,4-Hexadien-1-ol (*t,t*-HDO) as an energy transfer probe for ^{3}DOM***

t,t-HDO (i.e., sorbic alcohol) was spiked into samples to measure the photoproduction of $^{3}\mathrm{DOM}^{*}_{\mathrm{HDO}}$. For each sample, the formation of three *t,t*-HDO isomers (i.e., *c,c*-HDO, *c,t*-HDO, and *t,c*-HDO) and the loss of *t,t*-HDO were monitored to derive the overall production rate of four isomers, $R_{\mathrm{prod,\,HDO}}$ (M s^{-1}), for each initial *t,t*-HDO concentration:[30, 104]

$$\begin{aligned} R_{\mathrm{prod,\,HDO}} &= \frac{\mathrm{d[HDO]}}{\mathrm{dt}} = R_{\mathrm{f},\,c,t\text{-HDO}} + R_{\mathrm{f},\,c,c\text{-HDO}} + R_{\mathrm{f},\,t,c\text{-HDO}} + R_{\mathrm{f},\,t,t\text{-HDO}} \\ &= \frac{\mathrm{d}[c,t\text{-HDO}]}{\mathrm{dt}} + \frac{\mathrm{d}[c,c\text{-HDO}]}{\mathrm{dt}} + \frac{\mathrm{d}[t,c\text{-HDO}]}{\mathrm{dt}} + \frac{\mathrm{d}[t,t\text{-HDO}]}{\mathrm{dt}} \end{aligned} \qquad \text{(S25)}$$

where $R_{\mathrm{f},\,c,t\text{-HDO}}$ (M s^{-1}) is the formation rate of *c,t*-HDO, $R_{\mathrm{f},\,c,c\text{-HDO}}$ (M s^{-1}) is the formation rate of *c,c*-HDO, $R_{\mathrm{f},\,t,c\text{-HDO}}$ (M s^{-1}) is the formation rate of *t,c*-HDO, and $R_{\mathrm{f},\,t,t\text{-HDO}}$ (M s^{-1}) is the *reformation* rate of *t,t*-HDO upon the relaxation of the excited triplet state of *t,t*-HDO (i.e., *t,t*-HDO*).

To determine the rate constant for *t,t*-HDO reformation relative to that of *c,t*-HDO, a multiple linear regression of the *t,t*-HDO and *c,t*-HDO data in the initial rate kinetics regime was performed using Equation S26 (i.e., $y = ax_1 + bx_2$ where y = d[*t,t*-HDO]/dt, x_1 = d[*c,t*-HDO]/dt, and x_2 = [*t,t*-HDO]):[30, 104]

$$\frac{\mathrm{d}[t,t\text{-HDO}]}{\mathrm{dt}} = \frac{k_{t,t\text{-HDO}}}{k_{c,t\text{-HDO}}}\frac{\mathrm{d}[c,t\text{-HDO}]}{\mathrm{dt}} - k'_{t,t\text{-HDO}}[t,t\text{-HDO}] \qquad \text{(S26)}$$

where $k_{t,t\text{-HDO}}$ (s^{-1}) is the pseudo-first order rate constant for *t,t*-HDO reformation from *t,t*-HDO*, $k_{c,t\text{-HDO}}$ (s^{-1}) is the pseudo-first order rate constant for *c,t*-HDO formation from *t,t*-HDO*, $k'_{t,t\text{-HDO}}$ (s^{-1}) is the sum of pseudo-first-order rate constants for the reaction of *t,t*-HDO with ^{3}DOM* to form *t,t*-HDO* and reactions of *t,t*-HDO with other scavengers under steady-state conditions (e.g., $^{\bullet}$OH and $^{1}O_2$[30]), and [*t,t*-HDO] is the initial concentration of *t,t*-HDO.

To approximate the sample-specific *t,t*-HDO reformation, relative rate constants (i.e., $k_{t,t\text{-HDO}}/k_{c,t\text{-HDO}}$) of 2.58±0.15 and 2.63±0.28 were derived by regressing Equation S26 with data from bloom lysates and bloom supernatants, respectively.

For each sample, the steady-state concentration of $^3DOM^*$ in the *presence* of *t,t*-HDO, $[^3DOM^*_{HDO}]_{ss}$, was calculated by considering the formation of $^3DOM^*_{HDO}$ and the simultaneous quenching by *t,t*-HDO and other deactivation processes:[30]

$$[^3\mathrm{DOM}^*_{\mathrm{HDO}}]_{\mathrm{ss}} = \frac{R_{\mathrm{f},\,^3\mathrm{DOM}^*_{\mathrm{HDO}}}}{k_{t,t\text{-HDO},\,^3\mathrm{DOM}^*_{\mathrm{HDO}}}[t,t\text{-HDO}] + k'_{\mathrm{q},\,^3\mathrm{DOM}^*_{\mathrm{HDO}}}} \tag{S27}$$

where $R_{\mathrm{f},\,^3\mathrm{DOM}^*_{\mathrm{HDO}}}$ (M s^{-1}) is the formation rate of $^3DOM^*_{HDO}$, $k_{t,t\text{-HDO},\,^3\mathrm{DOM}^*_{\mathrm{HDO}}}$ (M^{-1} s^{-1}) is the second-order reaction rate constant of *t,t*-HDO with $^3DOM^*_{HDO}$, and $k'_{\mathrm{q},\,^3\mathrm{DOM}^*_{\mathrm{HDO}}}$ ($3.2(\pm0.6)\times10^5$ s^{-1}) is the sum of pseudo-first order rate constants for $^3DOM^*_{HDO}$ quenching via energy transfer to dissolved O_2 ($2.1(\pm0.2)\times10^5$ s^{-1} calculated from $k_{O_2}[O_{2(aq)}]$ where $k_{O_2} = 8.9(\pm0.6)\times10^8$ M^{-1} s^{-1} and $[O_{2(aq)}]$ = ~258 μM at T = 25 °C[94]) and via other non-O_2 dependent nonradiative relaxation pathways ($k_d^T = 9.0(\pm2.8)\times10^4$ s^{-1}).[75, 86]

To solve for $k_{t,t\text{-HDO},\,^3\mathrm{DOM}^*_{\mathrm{HDO}}}$ (**Table S28**), a linear regression of [*t,t*-HDO] (at varying concentrations of 50, 100, 250, 500, 1000, and 2000 μM) and $R_{\mathrm{p,\,HDO}}$ data was performed with the linearized form of Equation S28 (i.e., $y = ax + b$ where $y = [t,t\text{-HDO}]/R_{\mathrm{p,\,HDO}}$ and $x = [t,t\text{-HDO}]$):[30]

$$\frac{[t,t\text{-HDO}]}{R_{\mathrm{p,\,HDO}}} = \frac{[t,t\text{-HDO}]}{R_{\mathrm{f},\,^3\mathrm{DOM}^*_{\mathrm{HDO}}}} + \frac{k'_{\mathrm{q},\,^3\mathrm{DOM}^*_{\mathrm{HDO}}}}{R_{\mathrm{f},\,^3\mathrm{DOM}^*_{\mathrm{HDO}}} k_{t,t\text{-HDO},\,^3\mathrm{DOM}^*_{\mathrm{HDO}}}} \tag{S28}$$

The formation rate of $^3DOM^*_{HDO}$, $R_{\mathrm{f},\,^3\mathrm{DOM}^*_{\mathrm{HDO}}}$ (M s^{-1}), was calculated as:

$$R_{\mathrm{f},\,^3\mathrm{DOM}^*_{\mathrm{HDO}}} = \frac{1}{\mathrm{slope}} \tag{S29}$$

For each sample, the steady-state concentration of $^3DOM^*$ in the *absence* of *t,t*-HDO, $[^3DOM^*_{HDO}]_{ss}$, was calculated as:

$$[^3\mathrm{DOM}^*_{\mathrm{HDO}}]_{\mathrm{ss}} = \frac{R_{\mathrm{f},\,^3\mathrm{DOM}^*_{\mathrm{HDO}}}}{k'_{\mathrm{q},\,^3\mathrm{DOM}^*_{\mathrm{HDO}}}} = \frac{1}{k'_{\mathrm{q},\,^3\mathrm{DOM}^*_{\mathrm{HDO}}}}\frac{1}{\mathrm{slope}} \tag{S30}$$

The apparent quantum yield of $^3DOM^*_{HDO}$, $\Phi_{\mathrm{app},\,^3\mathrm{DOM}^*_{\mathrm{HDO}}}$ (mol mol-photons^{-1}), was calculated as:[30]

$$\Phi_{\mathrm{app},\,^3\mathrm{DOM}^*_{\mathrm{HDO}}} = \frac{R_{\mathrm{f},\,^3\mathrm{DOM}^*_{\mathrm{HDO}}}}{R_{\mathrm{a}}} = \frac{1}{R_{\mathrm{a}}}\frac{1}{\mathrm{slope}} \tag{S31}$$

The quantum yield coefficient of $^3\mathrm{DOM}^*_{\mathrm{HDO}}$ with *t,t*-HDO, f_{HDO} (L mol-photons^{-1}), was calculated as:

$$f_{\mathrm{HDO}} = \Phi_{\mathrm{app},\,^3\mathrm{DOM}^*_{\mathrm{HDO}}} \times \frac{k_{t,t\text{-HDO},\,^3\mathrm{DOM}^*_{\mathrm{HDO}}}}{k'_{\mathrm{q},\,^3\mathrm{DOM}^*_{\mathrm{HDO}}}} = \frac{1}{R_{\mathrm{a}}\mathrm{intercept}} \quad \text{(S32)}$$

The yield of $^1O_2$ the O_2-dependent quenching of $^3\mathrm{DOM}^*_{\mathrm{HDO}}$ was calculated as:[75, 103]

$$f_{^1\mathrm{O}_2\_^3\mathrm{DOM}^*_{\mathrm{HDO}}} = \frac{\Phi_{\mathrm{app},\,^1\mathrm{O}_2}(k_{\mathrm{O}_2}[\mathrm{O}_{2(\mathrm{aq})}] + k_{\mathrm{d}}^{\mathrm{T}})}{\Phi_{\mathrm{app},\,^3\mathrm{DOM}^*_{\mathrm{HDO}}} k_{\mathrm{O}_2}[\mathrm{O}_{2(\mathrm{aq})}]} \quad \text{(S33)}$$

$\Phi_{\mathrm{app},\,^3\mathrm{DOM}^*_{\mathrm{HDO}}}$ and f_{HDO} for bloom samples are summarized in **Tables S29-S34**.

**Table S28.** $k_{t,t\text{-HDO},\ ^3DOM^*_{HDO}}$ for bloom samples			
Sample ID	Bloom ID	OD_{680} (A.U.)	$k_{t,t\text{-HDO},\ ^3DOM^*_{HDO}}$ ($\times10^8$ M^{-1} s^{-1})
**Bloom lysates**			
Lake 261 09/04/2021	A	0.379±0.009	5.63±0.11
Lake 238 09/07/2021	B	0.349±0.018	5.81±0.05
Lake 147 08/30/2021	C	0.370±0.019	5.49±0.10
Lake 138 09/07/2021	D	0.417±0.026	5.97±0.22
Lake 33 09/04/2021	E	0.368±0.018	5.32±0.01
Lake 37 08/20/2021	F	0.267±0.013	5.56±0.16
Lake 38 08/29/2021	G	0.430±0.022	5.98±0.02
Lake 40 09/16/2021	H	0.392±0.020	6.07±0.05
Lake 78 08/22/2021	I	0.410±0.020	5.74±0.14
Lake 82 08/17/2021	J	0.475±0.024	6.25±0.09
Lake 93 08/29/2021	K	0.245±0.012	5.18±0.31
Lake 221 09/06/2021	L	0.542±0.027	6.14±0.14
**Bloom supernatants**			
Lake 261 09/04/2021	A	0.379±0.009	9.06±0.34
Lake 238 09/07/2021	B	0.349±0.018	9.07±0.24
Lake 147 08/30/2021	C	0.370±0.019	9.15±0.26
Lake 138 09/07/2021	D	0.417±0.026	9.13±0.56
Lake 33 09/04/2021	E	0.368±0.018	9.27±0.49
Lake 37 08/20/2021	F	0.267±0.013	9.00±0.52
Lake 38 08/29/2021	G	0.430±0.022	9.74±0.27
Lake 40 09/16/2021	H	0.392±0.020	9.67±0.32
Lake 78 08/22/2021	I	0.410±0.020	9.60±0.39
Lake 82 08/17/2021	J	0.475±0.024	9.73±0.57
Lake 93 08/29/2021	K	0.245±0.012	8.83±0.46
Lake 221 09/06/2021	L	0.542±0.027	9.95±0.37

**Table S28.** $k_{t,t\text{-HDO},\ ^3DOM^*_{HDO}}$ for bloom samples (continued)			
Sample ID	Bloom ID	OD_{680} (A.U.)	$k_{t,t\text{-HDO},\ ^3DOM^*_{HDO}}$ ($\times 10^8$ M^{-1} s^{-1})
**Mixtures of bloom lysates and Otisco Lake water / SRNOM**			
Lake 256 10/12/2021	Otisco	0.026±0.001	6.62±0.07
Lysate 25% + Otisco 75%	A	-	6.23±0.04
Lysate 50% + Otisco 50%	A	-	5.90±0.03
Lysate 75% + Otisco 25%	A	-	5.60±0.02
Lysate 25% + Otisco 75%	B	-	6.20±0.01
Lysate 50% + Otisco 50%	B	-	5.81±0.05
Lysate 75% + Otisco 25%	B	-	5.43±0.08
Lysate 25% + Otisco 75%	C	-	6.29±0.18
Lysate 50% + Otisco 50%	C	-	6.02±0.25
Lysate 75% + Otisco 25%	C	-	5.80±0.16
Lysate 25% + Otisco 75%	D	-	6.36±0.08
Lysate 50% + Otisco 50%	D	-	6.14±0.07
Lysate 75% + Otisco 25%	D	-	5.96±0.03
Lysate 25% + Otisco 75%	E	-	6.23±0.15
Lysate 50% + Otisco 50%	E	-	5.93±0.19
Lysate 75% + Otisco 25%	E	-	5.75±0.18
Lysate 25% + Otisco 75%	F	-	6.39±0.11
Lysate 50% + Otisco 50%	F	-	6.20±0.14
Lysate 75% + Otisco 25%	F	-	6.06±0.18
SRNOM	SRNOM	-	7.20±0.12
Lysate 25% + SRNOM 75%	A	-	6.92±0.08
Lysate 50% + SRNOM 50%	A	-	6.58±0.06
Lysate 75% + SRNOM 25%	A	-	6.11±0.05
Lysate 25% + SRNOM 75%	B	-	6.88±0.02
Lysate 50% + SRNOM 50%	B	-	6.47±0.05
Lysate 75% + SRNOM 25%	B	-	5.90±0.09
Lysate 25% + SRNOM 75%	C	-	6.93±0.17
Lysate 50% + SRNOM 50%	C	-	6.63±0.30
Lysate 75% + SRNOM 25%	C	-	6.24±0.23
Lysate 25% + SRNOM 75%	D	-	7.02±0.11
Lysate 50% + SRNOM 50%	D	-	6.78±0.09
Lysate 75% + SRNOM 25%	D	-	6.42±0.05
Lysate 25% + SRNOM 75%	E	-	6.94±0.19
Lysate 50% + SRNOM 50%	E	-	6.65±0.23
Lysate 75% + SRNOM 25%	E	-	6.29±0.23
Lysate 25% + SRNOM 75%	F	-	7.10±0.15
Lysate 50% + SRNOM 50%	F	-	6.93±0.18
Lysate 75% + SRNOM 25%	F	-	6.63±0.20

Table S29. $\Phi_{app,\ ^3DOM^*_{HDO}}$ for bloom lysates

Sample ID	Bloom ID	OD_{680} (A.U.)	$\Phi_{app,\ ^3DOM^*_{HDO}}$ ($\times 10^{-2}$ mol mol-photons^{-1})	Sample ID	Bloom ID	OD_{680} (A.U.)	$\Phi_{app,\ ^3DOM^*_{HDO}}$ ($\times 10^{-2}$ mol mol-photons^{-1})
Lake 261 09/04/2021	A	0.379±0.009	0.62±0.05				
Lake 238 09/07/2021	B	0.349±0.018	0.62±0.04				
Lake 147 08/30/2021	C	0.370±0.019	0.65±0.05				
Lake 138 09/07/2021	D	0.417±0.026	0.57±0.05				
Lake 33 09/04/2021	E	0.368±0.018	0.55±0.04				
Lake 37 08/20/2021	F	0.267±0.013	0.51±0.03				
Lake 38 08/29/2021	G	0.430±0.022	0.78±0.06				
Lake 40 09/16/2021	H	0.392±0.020	0.78±0.06				
Lake 78 08/22/2021	I	0.410±0.020	0.79±0.06				
Lake 82 08/17/2021	J	0.475±0.024	0.74±0.05				
Lake 93 08/29/2021	K	0.245±0.012	0.50±0.05				
Lake 221 09/06/2021	L	0.542±0.027	0.75±0.06				
Lake 256 10/12/2021	Otisco	0.026±0.001	1.28±0.11	SRNOM	-	-	1.26±0.10
Lysate 25% + Otisco 75%	A	-	0.91±0.07	Lysate 25% + SRNOM 75%	A	-	0.95±0.07
Lysate 50% + Otisco 50%	A	-	0.72±0.05	Lysate 50% + SRNOM 50%	A	-	0.79±0.06
Lysate 75% + Otisco 25%	A	-	0.59±0.04	Lysate 75% + SRNOM 25%	A	-	0.62±0.05
Lysate 25% + Otisco 75%	B	-	0.90±0.07	Lysate 25% + SRNOM 75%	B	-	0.93±0.07
Lysate 50% + Otisco 50%	B	-	0.71±0.05	Lysate 50% + SRNOM 50%	B	-	0.77±0.05
Lysate 75% + Otisco 25%	B	-	0.56±0.04	Lysate 75% + SRNOM 25%	B	-	0.60±0.04
Lysate 25% + Otisco 75%	C	-	0.95±0.06	Lysate 25% + SRNOM 75%	C	-	1.00±0.06
Lysate 50% + Otisco 50%	C	-	0.78±0.04	Lysate 50% + SRNOM 50%	C	-	0.85±0.05
Lysate 75% + Otisco 25%	C	-	0.66±0.04	Lysate 75% + SRNOM 25%	C	-	0.69±0.04
Lysate 25% + Otisco 75%	D	-	0.95±0.08	Lysate 25% + SRNOM 75%	D	-	1.03±0.08
Lysate 50% + Otisco 50%	D	-	0.78±0.06	Lysate 50% + SRNOM 50%	D	-	0.87±0.07
Lysate 75% + Otisco 25%	D	-	0.66±0.05	Lysate 75% + SRNOM 25%	D	-	0.70±0.05
Lysate 25% + Otisco 75%	E	-	0.97±0.09	Lysate 25% + SRNOM 75%	E	-	1.01±0.08
Lysate 50% + Otisco 50%	E	-	0.80±0.07	Lysate 50% + SRNOM 50%	E	-	0.84±0.07
Lysate 75% + Otisco 25%	E	-	0.69±0.06	Lysate 75% + SRNOM 25%	E	-	0.68±0.06
Lysate 25% + Otisco 75%	F	-	0.92±0.08	Lysate 25% + SRNOM 75%	F	-	0.92±0.07
Lysate 50% + Otisco 50%	F	-	0.75±0.06	Lysate 50% + SRNOM 50%	F	-	0.78±0.06
Lysate 75% + Otisco 25%	F	-	0.61±0.05	Lysate 75% + SRNOM 25%	F	-	0.64±0.05

Table S30. $\Phi_{app,\ ^{3}DOM^{*}_{HDO}}$ for bloom supernatants

Sample ID	Bloom ID	OD_{680} (A.U.)	$\Phi_{app,\ ^{3}DOM^{*}_{HDO}}$ ($\times10^{-2}$ mol mol-photons^{-1})	Sample ID	Bloom ID	OD_{680} (A.U.)	$\Phi_{app,\ ^{3}DOM^{*}_{HDO}}$ ($\times10^{-2}$ mol mol-photons^{-1})
Lake 261 09/04/2021	A	0.151±0.003	-	Lake 33 09/04/2021	E	0.368±0.018	3.48±0.35
	A	0.182±0.006	-	Lake 37 08/20/2021	F	0.267±0.013	3.29±0.33
	A	0.215±0.008	-	Lake 38 08/29/2021	G	0.430±0.022	4.34±0.44
	A	0.259±0.009	-	Lake 40 09/16/2021	H	0.392±0.020	3.91±0.36
	A	0.306±0.009	-	Lake 78 08/22/2021	I	0.410±0.020	4.13±0.39
	A	0.347±0.009	-	Lake 82 08/17/2021	J	0.475±0.024	4.00±0.34
	A	0.370±0.009	-	Lake 93 08/29/2021	K	0.245±0.012	3.18±0.31
	A	0.376±0.009	-	Lake 221 09/06/2021	L	0.542±0.027	4.95±0.45
	A	0.379±0.009	3.71±0.37				
Lake 238 09/07/2021	B	0.138±0.003	-	Lake 256 10/12/2021	Otisco	0.008±0.001	-
	B	0.159±0.006	-		Otisco	0.013±0.001	-
	B	0.195±0.009	-		Otisco	0.016±0.001	-
	B	0.242±0.009	-		Otisco	0.018±0.002	-
	B	0.283±0.014	-		Otisco	0.020±0.002	-
	B	0.321±0.015	-		Otisco	0.022±0.002	-
	B	0.338±0.017	-		Otisco	0.023±0.002	-
	B	0.345±0.017	-		Otisco	0.024±0.002	-
	B	0.349±0.018	3.61±0.30		Otisco	0.025±0.002	1.28±0.11
Lake 147 08/30/2021	C	0.078±0.009	-	Lake 256 10/12/2021	Otisco	0.009±0.001	-
	C	0.150±0.011	-		Otisco	0.014±0.001	-
	C	0.208±0.016	-		Otisco	0.017±0.001	-
	C	0.264±0.018	-		Otisco	0.019±0.001	-
	C	0.310±0.020	-		Otisco	0.021±0.002	-
	C	0.340±0.019	-		Otisco	0.023±0.002	-
	C	0.354±0.019	-		Otisco	0.024±0.002	-
	C	0.366±0.019	-		Otisco	0.025±0.002	-
	C	0.370±0.019	4.00±0.37		Otisco	0.026±0.002	1.28±0.11
Lake 138 09/07/2021	D	0.107±0.011	-				
	D	0.202±0.024	-				
	D	0.280±0.029	-				
	D	0.326±0.028	-				
	D	0.365±0.023	-				
	D	0.389±0.025	-				
	D	0.402±0.024	-				
	D	0.412±0.026	-				
	D	0.417±0.026	3.75±0.33				

Table S31. $\Phi_{app,\ ^{3}DOM^{*}_{HDO}}$ for bloom supernatants minus the contribution from Otisco Lake water

Sample ID	Bloom ID	ΔOD_{680} (A.U.)	$\Delta\Phi_{app,\ ^{3}DOM^{*}_{HDO}}$ ($\times10^{-2}$ mol mol-photons^{-1})	Sample ID	Bloom ID	ΔOD_{680} (A.U.)	$\Delta\Phi_{app,\ ^{3}DOM^{*}_{HDO}}$ ($\times10^{-2}$ mol mol-photons^{-1})
Lake 261 09/04/2021	A	0.143±0.007	-	Lake 33 09/04/2021	E	0.343±0.017	2.20±0.24
	A	0.168±0.008	-	Lake 37 08/20/2021	F	0.242±0.012	2.01±0.22
	A	0.199±0.010	-	Lake 38 08/29/2021	G	0.405±0.020	3.06±0.33
	A	0.241±0.012	-	Lake 40 09/16/2021	H	0.367±0.018	2.62±0.25
	A	0.286±0.014	-	Lake 78 08/22/2021	I	0.384±0.019	2.85±0.28
	A	0.325±0.016	-	Lake 82 08/17/2021	J	0.450±0.022	2.72±0.22
	A	0.346±0.017	-	Lake 93 08/29/2021	K	0.219±0.011	1.90±0.20
	A	0.352±0.018	-	Lake 221 09/06/2021	L	0.517±0.026	3.67±0.34
	A	0.354±0.018	2.43±0.26				
Lake 238 09/07/2021	B	0.130±0.006	-				
	B	0.146±0.007	-				
	B	0.179±0.009	-				
	B	0.224±0.011	-				
	B	0.263±0.013	-				
	B	0.299±0.015	-				
	B	0.315±0.016	-				
	B	0.320±0.016	-				
	B	0.323±0.016	2.33±0.19				
Lake 147 08/30/2021	C	0.070±0.003	-				
	C	0.137±0.007	-				
	C	0.192±0.010	-				
	C	0.246±0.012	-				
	C	0.290±0.015	-				
	C	0.318±0.016	-				
	C	0.331±0.017	-				
	C	0.341±0.017	-				
	C	0.345±0.017	2.72±0.26				
Lake 138 09/07/2021	D	0.099±0.005	-				
	D	0.189±0.009	-				
	D	0.264±0.013	-				
	D	0.308±0.015	-				
	D	0.345±0.017	-				
	D	0.367±0.018	-				
	D	0.379±0.019	-				
	D	0.388±0.019	-				
	D	0.391±0.020	2.47±0.22				

Table S32. f_{HDO} for bloom lysates

Sample ID	Bloom ID	OD_{680} (A.U.)	f_{HDO} (L mol-photons^{-1})	Sample ID	Bloom ID	OD_{680} (A.U.)	f_{HDO} (L mol-photons^{-1})
Lake 261 09/04/2021	A	0.379±0.009	10.9±0.7				
Lake 238 09/07/2021	B	0.349±0.018	11.3±0.8				
Lake 147 08/30/2021	C	0.370±0.019	11.2±0.6				
Lake 138 09/07/2021	D	0.417±0.026	10.7±0.5				
Lake 33 09/04/2021	E	0.368±0.018	9.1±0.7				
Lake 37 08/20/2021	F	0.267±0.013	8.9±0.7				
Lake 38 08/29/2021	G	0.430±0.022	14.5±1.0				
Lake 40 09/16/2021	H	0.392±0.020	14.7±1.0				
Lake 78 08/22/2021	I	0.410±0.020	14.1±0.7				
Lake 82 08/17/2021	J	0.475±0.024	14.5±0.9				
Lake 93 08/29/2021	K	0.245±0.012	8.0±0.4				
Lake 221 09/06/2021	L	0.542±0.027	14.4±0.9				
Lake 256 10/12/2021	Otisco	0.026±0.001	26.5±2.0	SRNOM	-	-	28.4±1.8
Lysate 25% + Otisco 75%	A	-	17.7±1.3	Lysate 25% + SRNOM 75%	A	-	20.5±1.3
Lysate 50% + Otisco 50%	A	-	13.3±0.9	Lysate 50% + SRNOM 50%	A	-	16.4±1.0
Lysate 75% + Otisco 25%	A	-	10.4±0.7	Lysate 75% + SRNOM 25%	A	-	11.8±0.8
Lysate 25% + Otisco 75%	B	-	17.5±1.4	Lysate 25% + SRNOM 75%	B	-	20.0±1.4
Lysate 50% + Otisco 50%	B	-	13.0±1.1	Lysate 50% + SRNOM 50%	B	-	15.5±1.2
Lysate 75% + Otisco 25%	B	-	9.6±0.8	Lysate 75% + SRNOM 25%	B	-	11.1±0.9
Lysate 25% + Otisco 75%	C	-	18.7±1.8	Lysate 25% + SRNOM 75%	C	-	21.7±1.9
Lysate 50% + Otisco 50%	C	-	14.7±1.5	Lysate 50% + SRNOM 50%	C	-	17.6±1.7
Lysate 75% + Otisco 25%	C	-	12.0±1.1	Lysate 75% + SRNOM 25%	C	-	13.4±1.3
Lysate 25% + Otisco 75%	D	-	19.0±1.4	Lysate 25% + SRNOM 75%	D	-	22.7±1.5
Lysate 50% + Otisco 50%	D	-	15.0±1.1	Lysate 50% + SRNOM 50%	D	-	18.4±1.2
Lysate 75% + Otisco 25%	D	-	12.3±0.9	Lysate 75% + SRNOM 25%	D	-	14.0±0.9
Lysate 25% + Otisco 75%	E	-	18.8±1.2	Lysate 25% + SRNOM 75%	E	-	21.8±1.2
Lysate 50% + Otisco 50%	E	-	14.8±0.9	Lysate 50% + SRNOM 50%	E	-	17.4±0.9
Lysate 75% + Otisco 25%	E	-	12.3±0.7	Lysate 75% + SRNOM 25%	E	-	13.4±0.6
Lysate 25% + Otisco 75%	F	-	18.5±1.3	Lysate 25% + SRNOM 75%	F	-	20.4±1.2
Lysate 50% + Otisco 50%	F	-	14.5±0.9	Lysate 50% + SRNOM 50%	F	-	17.0±0.9
Lysate 75% + Otisco 25%	F	-	11.6±0.6	Lysate 75% + SRNOM 25%	F	-	13.3±0.7

Table S33. f_{HDO} for bloom supernatants

Sample ID	Bloom ID	OD_{680} (A.U.)	f_{HDO} (L mol-photons^{-1})	Sample ID	Bloom ID	OD_{680} (A.U.)	f_{HDO} (L mol-photons^{-1})
Lake 261 09/04/2021	A	0.151±0.003	-	Lake 33 09/04/2021	E	0.368±0.018	100.6±4.7
	A	0.182±0.006	-	Lake 37 08/20/2021	F	0.267±0.013	92.3±3.9
	A	0.215±0.008	-	Lake 38 08/29/2021	G	0.430±0.022	132.0±9.8
	A	0.259±0.009	-	Lake 40 09/16/2021	H	0.392±0.020	118.0±7.0
	A	0.306±0.009	-	Lake 78 08/22/2021	I	0.410±0.020	123.6±6.7
	A	0.347±0.009	-	Lake 82 08/17/2021	J	0.475±0.024	121.3±3.0
	A	0.370±0.009	-	Lake 93 08/29/2021	K	0.245±0.012	87.7±4.1
	A	0.376±0.009	-	Lake 221 09/06/2021	L	0.542±0.027	153.7±8.2
	A	0.379±0.009	105.0±6.5				
Lake 238 09/07/2021	B	0.138±0.003	-	Lake 256 10/12/2021	Otisco	0.008±0.001	-
	B	0.159±0.006	-		Otisco	0.013±0.001	-
	B	0.195±0.009	-		Otisco	0.016±0.001	-
	B	0.242±0.009	-		Otisco	0.018±0.002	-
	B	0.283±0.014	-		Otisco	0.020±0.002	-
	B	0.321±0.015	-		Otisco	0.022±0.002	-
	B	0.338±0.017	-		Otisco	0.023±0.002	-
	B	0.345±0.017	-		Otisco	0.024±0.002	-
	B	0.349±0.018	102.6±11.3		Otisco	0.025±0.002	26.5±2.0
Lake 147 08/30/2021	C	0.078±0.009	-	Lake 256 10/12/2021	Otisco	0.009±0.001	-
	C	0.150±0.011	-		Otisco	0.014±0.001	-
	C	0.208±0.016	-		Otisco	0.017±0.001	-
	C	0.264±0.018	-		Otisco	0.019±0.001	-
	C	0.310±0.020	-		Otisco	0.021±0.002	-
	C	0.340±0.019	-		Otisco	0.023±0.002	-
	C	0.354±0.019	-		Otisco	0.024±0.002	-
	C	0.366±0.019	-		Otisco	0.025±0.002	-
	C	0.370±0.019	114.4±7.3		Otisco	0.026±0.002	26.5±2.0
Lake 138 09/07/2021	D	0.107±0.011	-				
	D	0.202±0.024	-				
	D	0.280±0.029	-				
	D	0.326±0.028	-				
	D	0.365±0.023	-				
	D	0.389±0.025	-				
	D	0.402±0.024	-				
	D	0.412±0.026	-				
	D	0.417±0.026	107.4±16.1				

Table S34. f_{HDO} for bloom supernatants minus the contribution from Otisco Lake water

Sample ID	Bloom ID	ΔOD_{680} (A.U.)	Δf_{HDO} (L mol-photons^{-1})	Sample ID	Bloom ID	ΔOD_{680} (A.U.)	Δf_{HDO} (L mol-photons^{-1})
Lake 261 09/04/2021	A	0.143±0.007	-	Lake 33 09/04/2021	E	0.343±0.017	74.1±2.7
	A	0.168±0.008	-	Lake 37 08/20/2021	F	0.242±0.012	65.8±1.8
	A	0.199±0.010	-	Lake 38 08/29/2021	G	0.405±0.020	105.5±7.8
	A	0.241±0.012	-	Lake 40 09/16/2021	H	0.367±0.018	91.5±5.0
	A	0.286±0.014	-	Lake 78 08/22/2021	I	0.384±0.019	97.1±4.6
	A	0.325±0.016	-	Lake 82 08/17/2021	J	0.450±0.022	94.8±1.0
	A	0.346±0.017	-	Lake 93 08/29/2021	K	0.219±0.011	61.2±2.0
	A	0.352±0.018	-	Lake 221 09/06/2021	L	0.517±0.026	127.2±6.1
	A	0.354±0.018	78.5±4.4				
Lake 238 09/07/2021	B	0.130±0.006	-				
	B	0.146±0.007	-				
	B	0.179±0.009	-				
	B	0.224±0.011	-				
	B	0.263±0.013	-				
	B	0.299±0.015	-				
	B	0.315±0.016	-				
	B	0.320±0.016	-				
	B	0.323±0.016	76.1±9.3				
Lake 147 08/30/2021	C	0.070±0.003	-				
	C	0.137±0.007	-				
	C	0.192±0.010	-				
	C	0.246±0.012	-				
	C	0.290±0.015	-				
	C	0.318±0.016	-				
	C	0.331±0.017	-				
	C	0.341±0.017	-				
	C	0.345±0.017	87.9±5.3				
Lake 138 09/07/2021	D	0.099±0.005	-				
	D	0.189±0.009	-				
	D	0.264±0.013	-				
	D	0.308±0.015	-				
	D	0.345±0.017	-				
	D	0.367±0.018	-				
	D	0.379±0.019	-				
	D	0.388±0.019	-				
	D	0.391±0.020	80.8±14.1				

11. Correlation matrices of $\Phi_{app,RI}$ for whole water samples

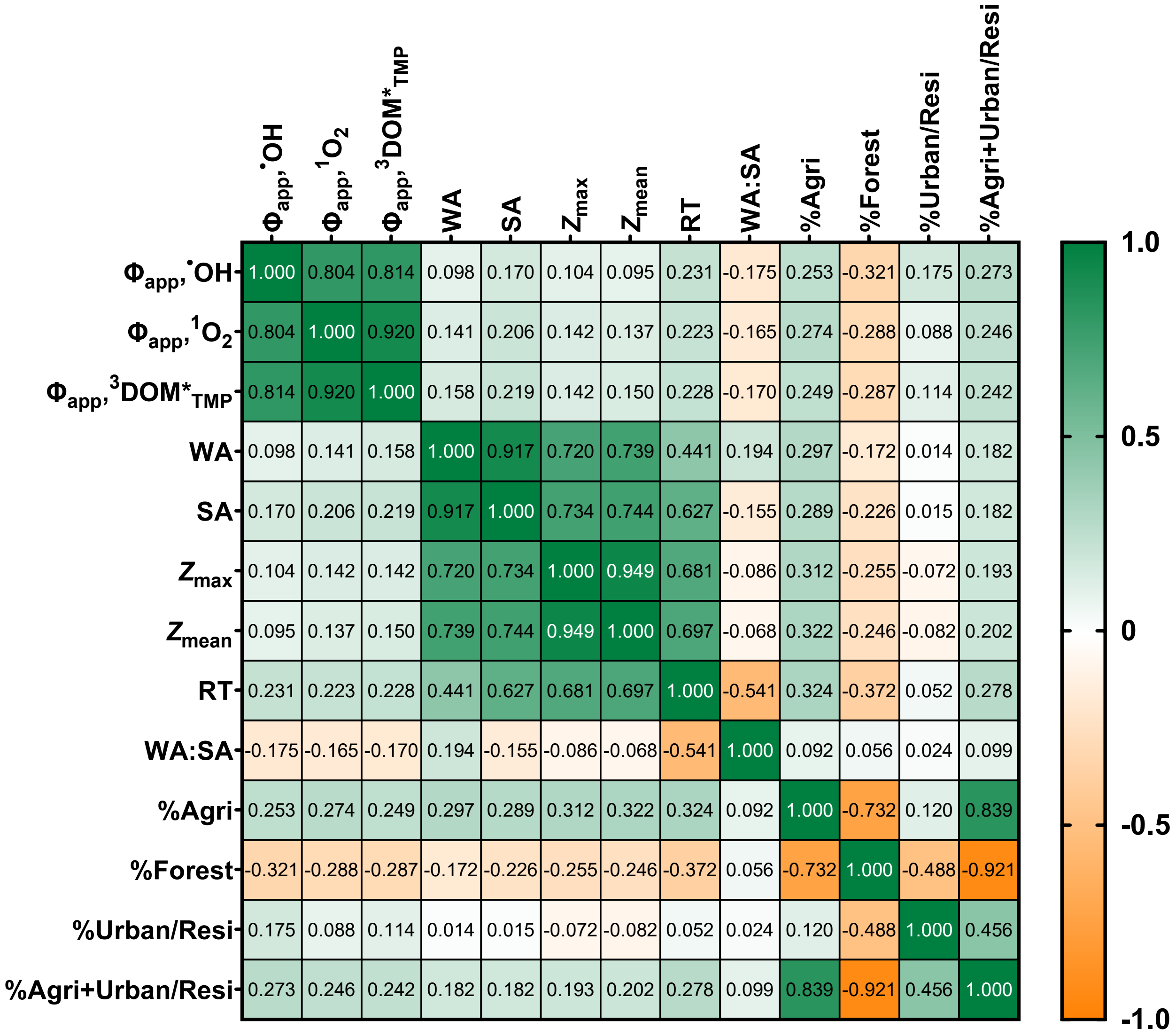

Figure S4. Spearman's correlation matrix between $\Phi_{app,RI}$ and lake-watershed characteristics for whole water samples (n=257) from CSLAP lakes. "WA" represents the lake watershed area (ha), "SA" represents the lake surface area (ha), "Z_{max}" represents the maximum depth of the lake (m), "Z_{mean}" represents the mean depth of the lake (m), "RT" represents the water residence time of the lake (year), "WA:SA" represents the watershed-to-surface-area ratio, "%Agri" represents the percent agricultural land usage in the lake watershed, "%Forest" represents the percent forested land usage in the lake watershed, "%Urban/Resi" represents the percent urban/residential land usage in the lake watershed, and "%Agri+Urban/Resi" represents the percent agricultural and urban/residential land usage in the lake watershed.

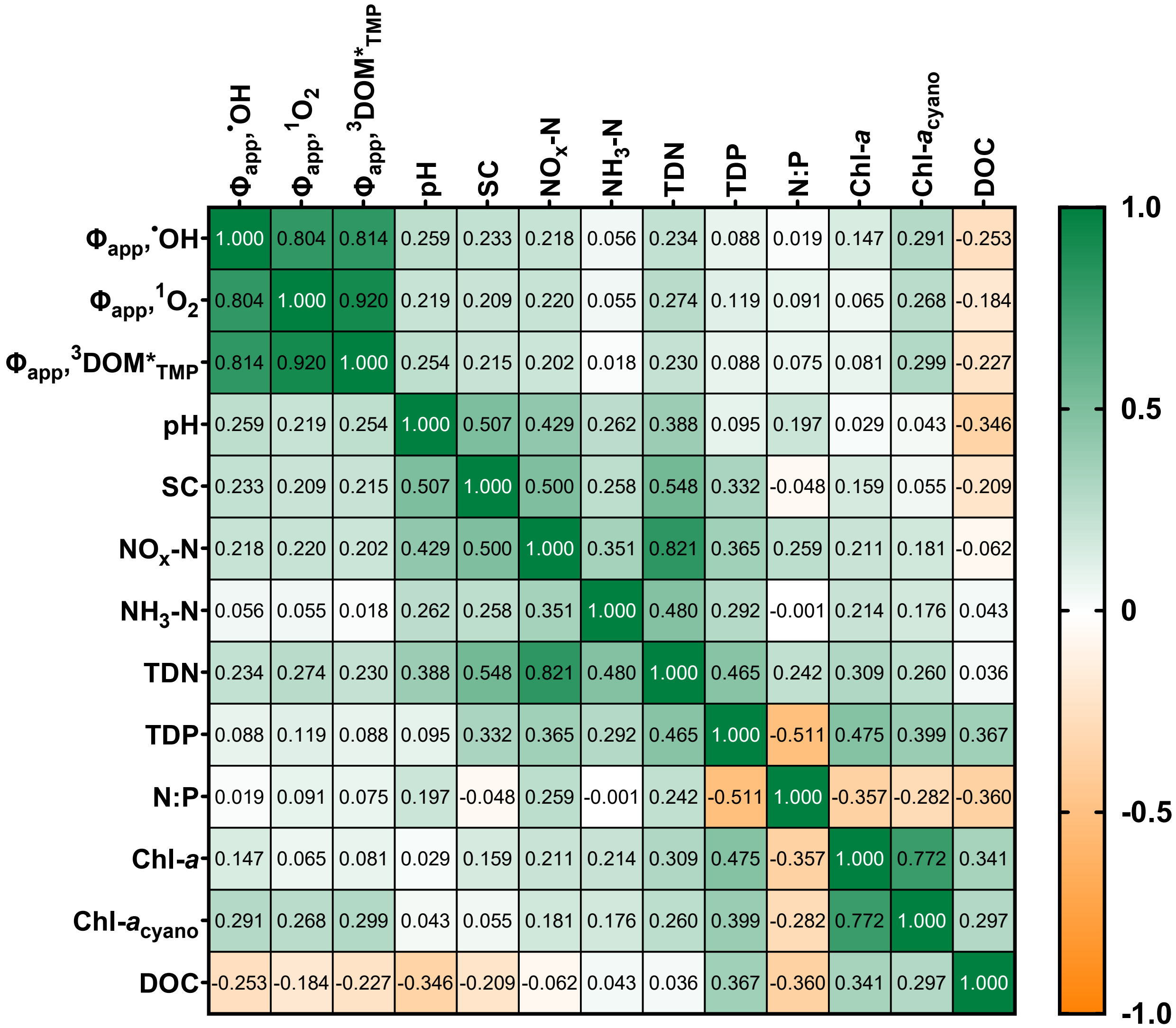

Figure S5. Spearman's correlation matrix between $\Phi_{app,RI}$ and physicochemical parameters for whole water samples (n=257) from CSLAP lakes. "SC" represents specific conductance (μS/cm), "NO_x-N" represents the concentration of nitrate-nitrite nitrogen (μgN/L), "NH_3-N" represents the concentration of ammonia nitrogen (μg/L), "TDN" represents the concentration of total dissolved nitrogen (μgN/L), "TDP" represents the concentration of total dissolved phosphorus (μg/L), "N:P" represents the concentration ratio of total nitrogen to total phosphorus, "Chl-*a*" represents the concentration of chlorophyll *a* (μg/L), and "Chl-a_{cyano}" represents the concentration of cyanobacterial chlorophyll *a* (μg/L).

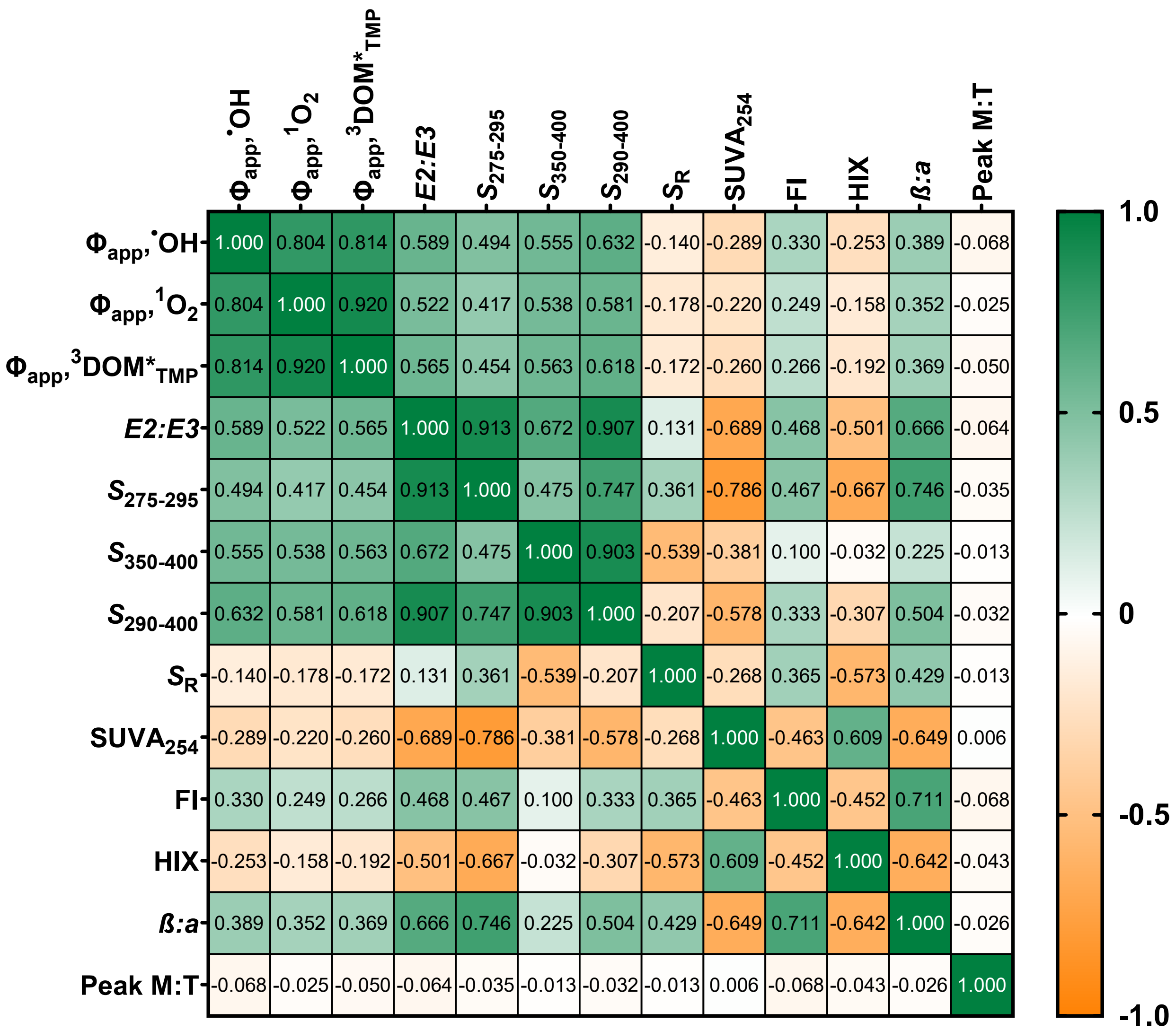

	$\Phi_{app,{}^{\bullet}OH}$	$\Phi_{app,{}^1O_2}$	$\Phi_{app,{}^3DOM^*_{TMP}}$	*E2:E3*	$S_{275-295}$	$S_{350-400}$	$S_{290-400}$	S_R	$SUVA_{254}$	FI	HIX	*ß:a*	Peak M:T
$\Phi_{app,{}^{\bullet}OH}$	1.000	0.804	0.814	0.589	0.494	0.555	0.632	-0.140	-0.289	0.330	-0.253	0.389	-0.068
$\Phi_{app,{}^1O_2}$	0.804	1.000	0.920	0.522	0.417	0.538	0.581	-0.178	-0.220	0.249	-0.158	0.352	-0.025
$\Phi_{app,{}^3DOM^*_{TMP}}$	0.814	0.920	1.000	0.565	0.454	0.563	0.618	-0.172	-0.260	0.266	-0.192	0.369	-0.050
E2:E3	0.589	0.522	0.565	1.000	0.913	0.672	0.907	0.131	-0.689	0.468	-0.501	0.666	-0.064
$S_{275-295}$	0.494	0.417	0.454	0.913	1.000	0.475	0.747	0.361	-0.786	0.467	-0.667	0.746	-0.035
$S_{350-400}$	0.555	0.538	0.563	0.672	0.475	1.000	0.903	-0.539	-0.381	0.100	-0.032	0.225	-0.013
$S_{290-400}$	0.632	0.581	0.618	0.907	0.747	0.903	1.000	-0.207	-0.578	0.333	-0.307	0.504	-0.032
S_R	-0.140	-0.178	-0.172	0.131	0.361	-0.539	-0.207	1.000	-0.268	0.365	-0.573	0.429	-0.013
$SUVA_{254}$	-0.289	-0.220	-0.260	-0.689	-0.786	-0.381	-0.578	-0.268	1.000	-0.463	0.609	-0.649	0.006
FI	0.330	0.249	0.266	0.468	0.467	0.100	0.333	0.365	-0.463	1.000	-0.452	0.711	-0.068
HIX	-0.253	-0.158	-0.192	-0.501	-0.667	-0.032	-0.307	-0.573	0.609	-0.452	1.000	-0.642	-0.043
ß:a	0.389	0.352	0.369	0.666	0.746	0.225	0.504	0.429	-0.649	0.711	-0.642	1.000	-0.026
Peak M:T	-0.068	-0.025	-0.050	-0.064	-0.035	-0.013	-0.032	-0.013	0.006	-0.068	-0.043	-0.026	1.000

Figure S6. Spearman's correlation matrix between $\Phi_{app,RI}$ and optical indices for whole water samples (n=257) from CSLAP lakes. "*E2:E3*" represents the ratio of absorption coefficients at 250 and 365 nm, "$S_{275-295}$" represents the spectral slope coefficient from 275 to 295 nm, "$S_{350-400}$" represents the spectral slope coefficient from 350 to 400 nm, "$S_{290-400}$" represents the spectral slope coefficient from 290 to 400 nm, "S_R" represents the ratio of spectral slope coefficient $S_{275-295}$ to $S_{350-400}$, "$SUVA_{254}$" represents the specific UV absorbance at 254 nm (L mg C^{-1}•m^{-1}), "FI" represents fluorescence index, "HIX" represents humification index, "*β:α*" represents freshness index, and "Peak M:T" represents the ratio of microbial humic-like to protein-like DOM fluorescence.

12. Comparisons of $\Phi_{app,RI}$ for whole water samples

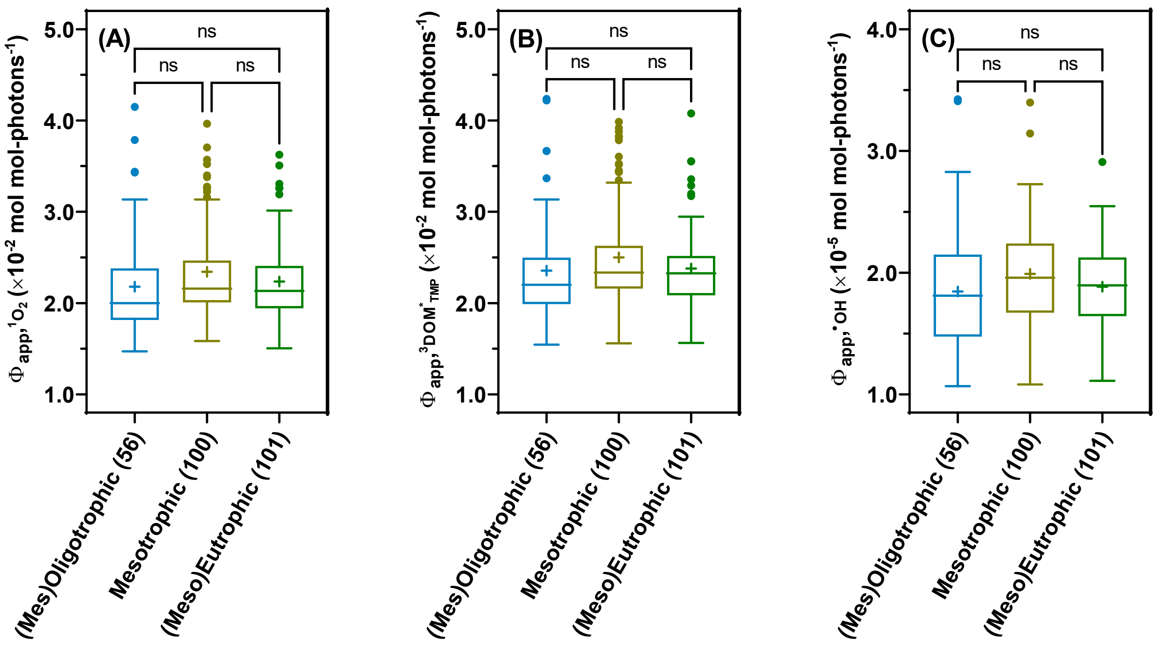

Figure S7. Multiple comparisons of $\Phi_{app,RI}$ for whole water samples from CSLAP lakes: **(A)** Box-and-whiskers plots of $\Phi_{app, ^1O_2}$ for whole water samples (n=257) grouped by the trophic state of lakes (i.e., (mes)oligotrophic, mesotrophic, and (meso)eutrophic). **(B)** Box-and-whiskers plots of $\Phi_{app, ^3DOM^*_{TMP}}$ for whole water samples grouped by the trophic state of lakes. **(C)** Box-and-whiskers plots of $\Phi_{app, \cdot OH}$ for whole water samples grouped by the trophic state of lakes. Each box extends from the 25th to 75th percentiles. The whiskers extend down to the 25th percentile minus 1.5 times the interquartile range and up to the 75th percentile plus 1.5 times the interquartile range. The centerline and "+" sign mark the median and mean, respectively. Filled circles represent the outliers. The numbers in parentheses next to the x-axis tick labels represent the counts of samples in different categories. "ns" represents no statistically significant difference.

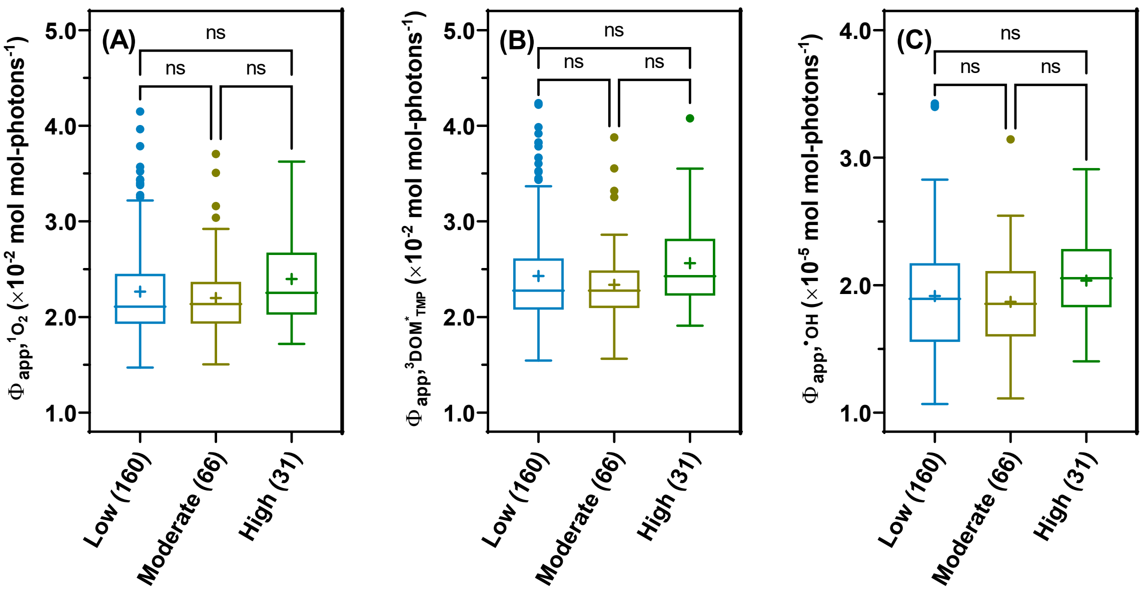

Figure S8. Multiple comparisons of $\Phi_{app,RI}$ for whole water samples from CSLAP lakes: **(A)** Box-and-whiskers plot of $\Phi_{app,{}^1O_2}$ for whole water samples (n=257) grouped by the bloom susceptibility of lakes (i.e., low, moderate, and high susceptibility). **(B)** Box-and-whiskers plot of $\Phi_{app,{}^3DOM^*_{TMP}}$ for whole water samples grouped by the bloom susceptibility of lakes. **(C)** Box-and-whiskers plot of $\Phi_{app,{}^{\bullet}OH}$ for whole water samples grouped by the bloom susceptibility of lakes. Each box extends from the 25th to 75th percentiles. The whiskers extend down to the 25th percentile minus 1.5 times the interquartile range and up to the 75th percentile plus 1.5 times the interquartile range. The centerline and "+" sign mark the median and mean, respectively. Filled circles represent the outliers. The numbers in parentheses next to the x-axis tick labels represent the counts of samples in different categories. "ns" represents no statistically significant difference.

13. Performance statistics of the OPLS and MLR models of $\Phi_{app,RI}$ for whole water samples

Table S35. Performance statistics of the OPLS model of $\Phi_{app,RI}$ for Chl-a_{cyano}-containing whole water samples

Component	R^2X	Eigenvalue	R^2	Q^2	R^2Y	Eigenvalue Y
Overall Model	0.608		0.553	0.425	0.978	
Predictive	0.130		0.553	0.425	0.978	
P1	0.0630	1.64	0.514	0.413	0.820	2.460
P2	0.0671	1.74	0.0383	0.0117	0.158	0.474
Orthogonal in X	0.478		5.12×10^{-4}			
O1	0.251	6.53	0			
O2	0.123	3.19	2.79×10^{-4}			
O3	0.104	2.69	5.12×10^{-4}			
Y Variable			R^2VY (cum)	Q^2VY (cum)		
$\Phi_{app,\,^1O_2}$			0.562	0.415		
$\Phi_{app,\,^3DOM^*_{TMP}}$			0.632	0.507		
$\Phi_{app,\,^{\bullet}OH}$			0.465	0.353		

X Variable	VIP Score (>1.0)	*X* Variable	VIP Score (≤1.0)
Chl-a_{cyano} (μg/L)	2.376	*E2:E3*	0.992
Ch-*a* (μg/L)	1.441	%Forested	0.952
TDN (μg/L)	1.188	Maximum Depth (m)	0.928
NO_x-N (μg/L)	1.181	Mean Depth (m)	0.927
Fluorescence Index (FI)	1.089	Residence Time (year)	0.925
$S_{290–400}$ (μm^{-1})	1.015	Freshness Index ($\beta:\alpha$)	0.906
		$SUVA_{254}$ (L mg $C^{-1}\bullet m^{-1}$)	0.896
		Peak M:T	0.856
		Surface Area (ha)	0.854
		Watershed Area (ha)	0.832
		%Urban/Residential	0.830
		N:P	0.798
		S_R	0.794
		Specific Conductance (μS/cm)	0.723
		Humification Index (HIX)	0.706
		%Agricultural	0.671
		pH	0.637
		TDP (μg/L)	0.595
		Watershed Area: Surface Area	0.572
		NH_3-N (μg/L)	0.512

R^2X = the fraction of X variation modeled in the component, using the X model; Eigenvalue = the number of X variables times R^2X; R^2 = the fraction of Y variation modeled by X in the component, using the X model; Q^2 = the overall cross-validated R^2 for the component; R^2Y = the fraction of Y variation modeled by Y in the component, using the Y model; Eigenvalue Y = the number of Y variables times R^2Y; R^2VY (cum) = the cumulative predicted fraction (cross validation) of the variation of Y; Q^2VY (cum) = the cumulative fraction of the variation of the Y variable explained after the selected component; VIP = variable importance in the projection.

**Table S36.** Performance statistics of the MLR models of $\Phi_{app,RI}$ for Chl-a_{cyano}-containing whole water samples						
$\Phi_{app, ^1O_2}$ Model Criterion		Coefficient	Estimate	Standard Error	p Value	VIF
Adjusted R^2	0.497	β0 (intercept)	1.537	0.1893	<0.0001	-
RMSE	0.2537	β1 (Chl-a_{cyano} (μg/L))	0.01267	1.112×10^{-3}	<0.0001	1.028
AIC	-357.5	β2 ($S_{290–400}$ (μm^{-1}))	0.03247	9.564×10^{-3}	0.0009	1.028
$\Phi_{app, ^3DOM^*_{TMP}}$ Model Criterion		Coefficient	Estimate	Standard Error	p Value	VIF
Adjusted R^2	0.594	β0 (intercept)	1.620	0.1650	<0.0001	-
RMSE	0.2211	β1 (Chl-a_{cyano} (μg/L))	0.01338	9.692×10^{-4}	<0.0001	1.028
AIC	-394.1	β2 ($S_{290–400}$ (μm^{-1}))	0.03624	8.336×10^{-3}	<0.0001	1.028
$\Phi_{app, \cdot OH}$ Model Criterion		Coefficient	Estimate	Standard Error	p Value	VIF
Adjusted R^2	0.363	β0 (intercept)	1.174	0.1712	<0.0001	-
RMSE	0.2295	β1 (Chl-a_{cyano} (μg/L))	0.008347	1.006×10^{-3}	<0.0001	1.028
AIC	-384.2	β2 ($S_{290–400}$ (μm^{-1}))	0.03670	8.651×10^{-3}	<0.0001	1.028

Adjusted R^2 = the adjusted determination coefficient for the model; RMSE = root mean square of the errors; AIC = Akaike information criterion; VIF = variance inflation factor.

14. Correlations between $\Phi_{app,RI}$ and Chl-a_{cyano}, Chl-a, or %Chl-a_{cyano} for whole water samples

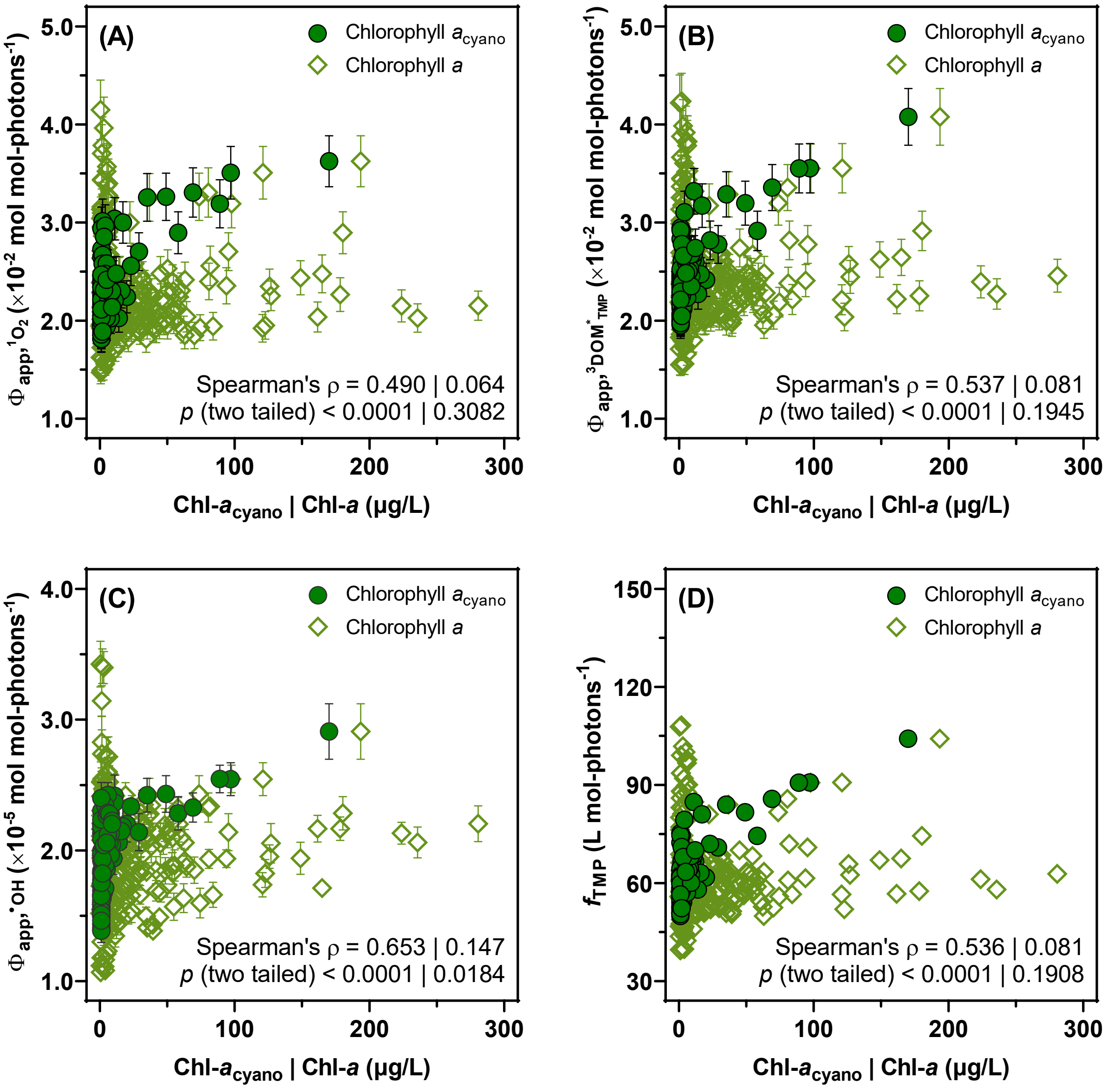

Figure S9. Correlations between $\Phi_{app,RI}$ and cyanobacterial chlorophyll a (Chl-a_{cyano}) or chlorophyll a (Chl-a) for whole water samples from CSLAP lakes: **(A)** Spearman's correlations between $\Phi_{app, {}^1O_2}$ and Chl-a for whole water samples (n=257; [DOC]=3.2±1.0 mg C/L; pH 7.4±0.5) or between $\Phi_{app, {}^1O_2}$ and Chl-a_{cyano} for Chl-a_{cyano}-containing whole water samples (n=133; [DOC]=3.5±1.2 mg C/L; pH 7.4±0.5). **(B)** Spearman's correlations between $\Phi_{app, {}^3DOM^*_{TMP}}$ and Chl-a for whole water samples or between $\Phi_{app, {}^3DOM^*_{TMP}}$ and Chl-a_{cyano} for Chl-a_{cyano}-containing whole water samples. **(C)** Spearman's correlations between $\Phi_{app, \cdot OH}$ and Chl-a for whole water samples or between $\Phi_{app, \cdot OH}$ and Chl-a_{cyano} for Chl-a_{cyano}-containing whole water samples. **(D)** Spearman's correlations between f_{TMP} and Chl-a for whole water samples or between f_{TMP} and Chl-a_{cyano} for Chl-a_{cyano}-containing whole water samples. Error bars represent the standard deviations from duplicate measurements of $\Phi_{app,RI}$ or f_{TMP}; where absent, bars fall within symbols.

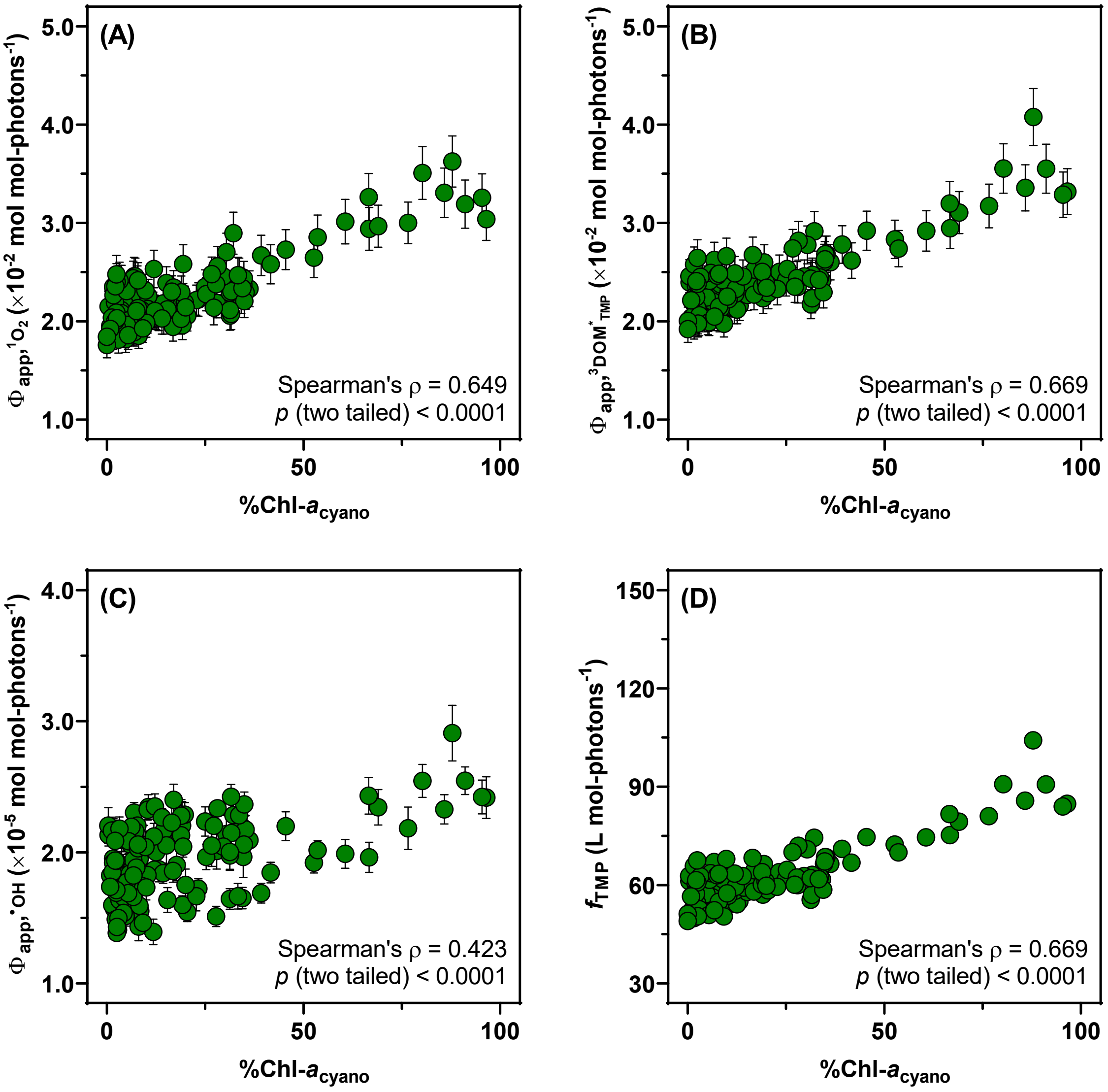

Figure S10. Correlations between $\Phi_{app,RI}$ and the proportion of cyanobacterial chlorophyll *a* (Chl-a_{cyano}) in Chl-*a* (%Chl-a_{cyano}) for Chl-a_{cyano}-containing whole water samples from CSLAP lakes: **(A)** Spearman's correlation between $\Phi_{app,\,^1O_2}$ and %Chl-a_{cyano} for Chl-a_{cyano}-containing whole water samples (*n*=133; [DOC]=3.5±1.2 mg C/L; pH 7.4±0.5). **(B)** Spearman's correlation between $\Phi_{app,\,^3DOM^*_{TMP}}$ and %Chl-a_{cyano} for Chl-a_{cyano}-containing whole water samples. **(C)** Spearman's correlation between $\Phi_{app,\,^{\bullet}OH}$ and %Chl-a_{cyano} for Chl-a_{cyano}-containing whole water samples. **(D)** Spearman's correlation between f_{TMP} and %Chl-a_{cyano} for Chl-a_{cyano}-containing whole water samples. Error bars represent the standard deviations from duplicate measurements of $\Phi_{app,RI}$ or f_{TMP}; where absent, bars fall within symbols.

15. Comparisons of $\Phi_{app,RI}$ for bloom lysates

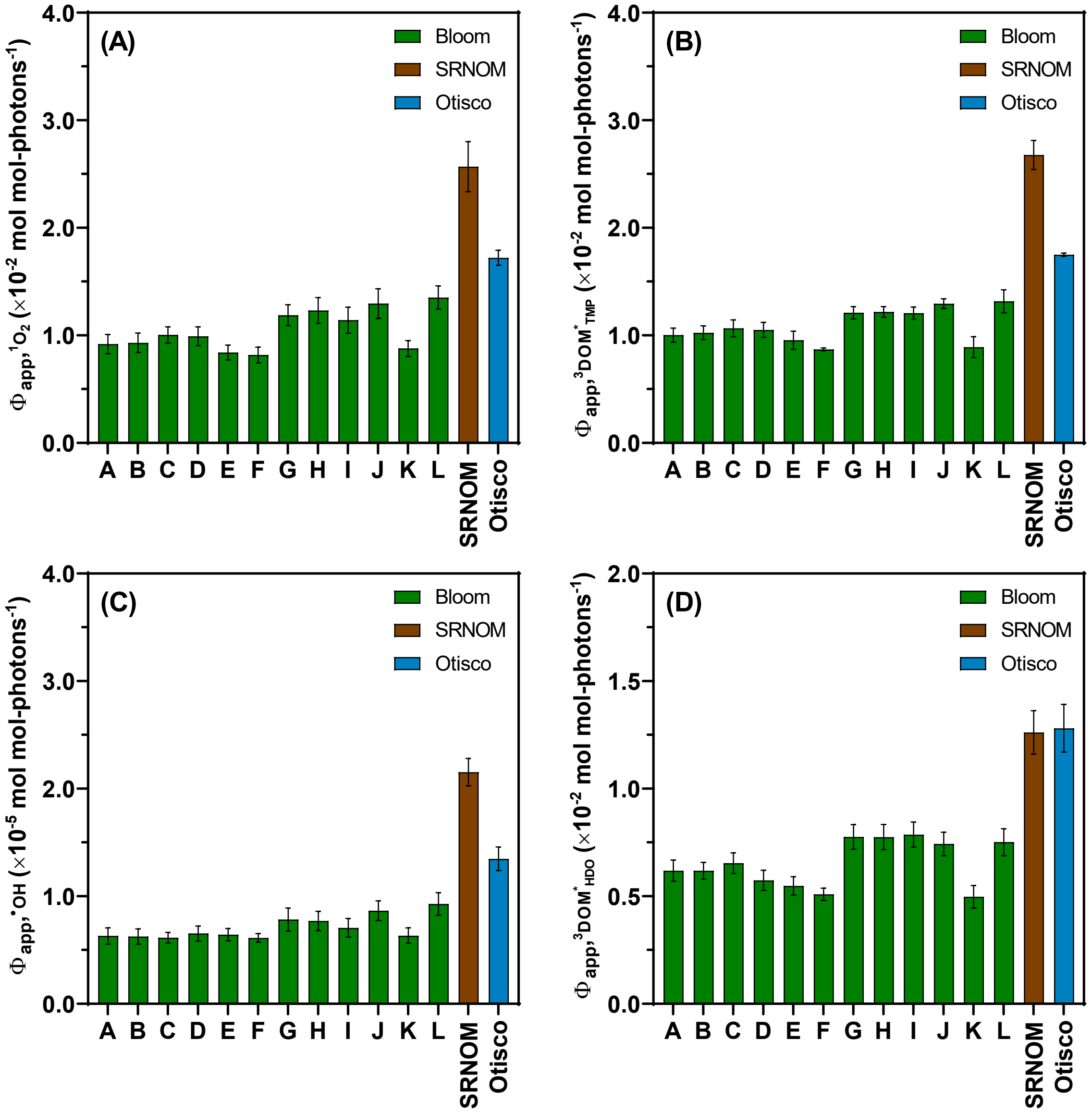

Figure S11. Comparisons of $\Phi_{app,RI}$ for the lysates extracted from bloom samples recultivated in unfiltered Otisco Lake water until the stationary phase: **(A)** $\Phi_{app,{}^1O_2}$ for bloom lysates (n=12; [DOC]=3.0±0.1 mg C/L; pH 7.5±0.3), SRNOM, and Otisco Lake water (incubated under the same conditions as bloom samples). **(B)** $\Phi_{app,{}^3DOM^*_{TMP}}$ for bloom lysates, SRNOM, and Otisco Lake water. **(C)** $\Phi_{app,{}^\bullet OH}$ for bloom lysates, SRNOM, and Otisco Lake water. **(D)** $\Phi_{app,{}^3DOM^*_{HDO}}$ for bloom lysates, SRNOM, and Otisco Lake water. Error bars represent the standard deviations from duplicate measurements of $\Phi_{app,RI}$; where absent, bars fall within bars.

16. Correlations between $\Phi_{app,RI}$ and AOC for bloom lysates

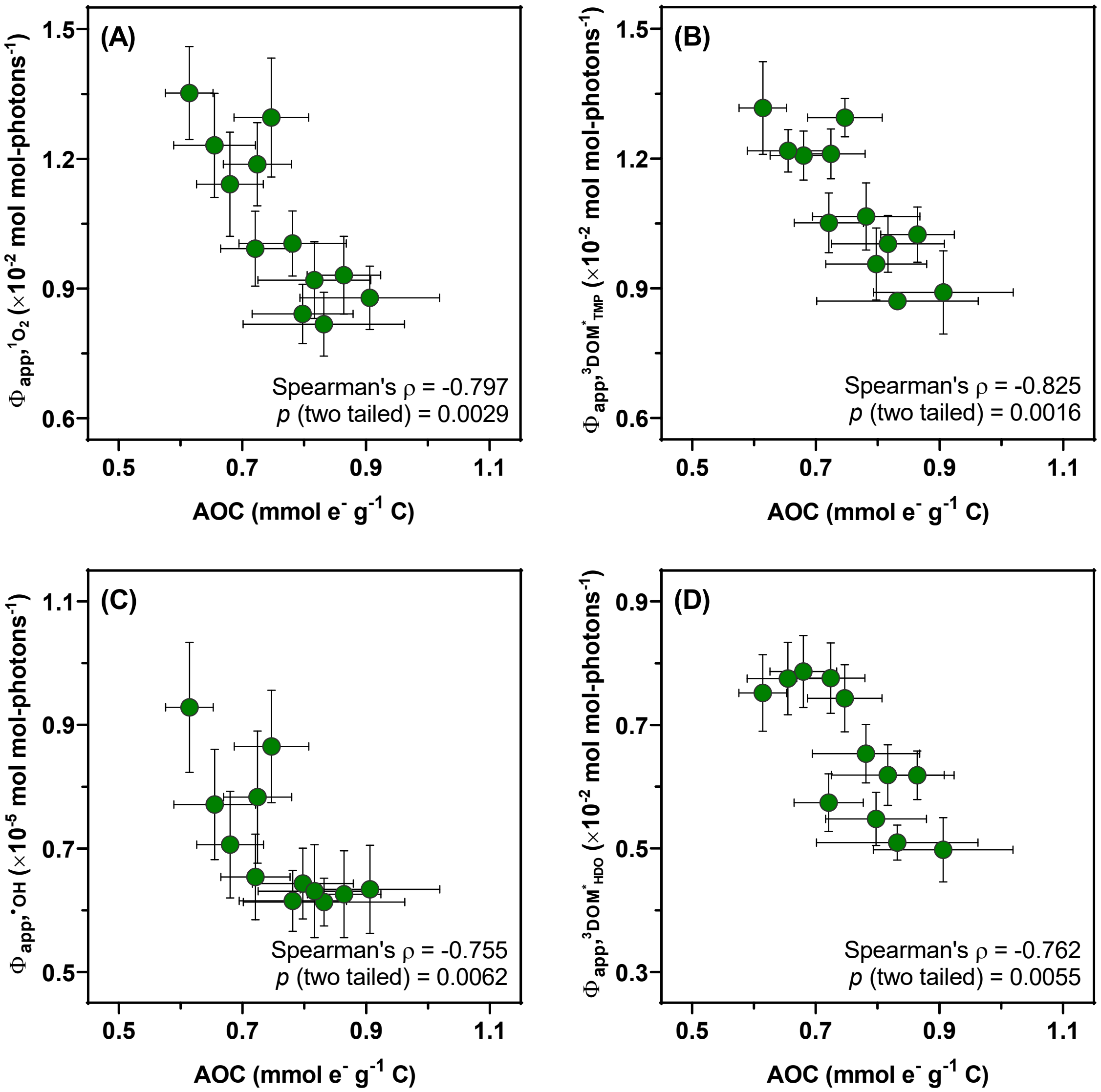

Figure S12. Correlations between $\Phi_{app,RI}$ and antioxidant capacity (AOC) for the lysates extracted from bloom samples recultivated in unfiltered Otisco Lake water until the stationary phase: **(A)** Spearman's correlation between $\Phi_{app,\ ^1O_2}$ and AOC for bloom lysates (*n*=12; [DOC]=3.0±0.1 mg C/L; pH 7.5±0.3). **(B)** Spearman's correlation between $\Phi_{app,\ ^3DOM^*_{TMP}}$ and AOC for bloom lysates. **(C)** Spearman's correlation between $\Phi_{app,\ ^\bullet OH}$ and AOC for bloom lysates. **(D)** Spearman's correlation between $\Phi_{app,\ ^3DOM^*_{HDO}}$ and AOC for bloom lysates. Error bars represent the standard deviations from duplicate measurements of $\Phi_{app,RI}$ and AOC; where absent, bars fall within symbols.

17. Changes in $\Phi_{app,RI}$ upon mixing bloom lysates with SRNOM or Otisco Lake water

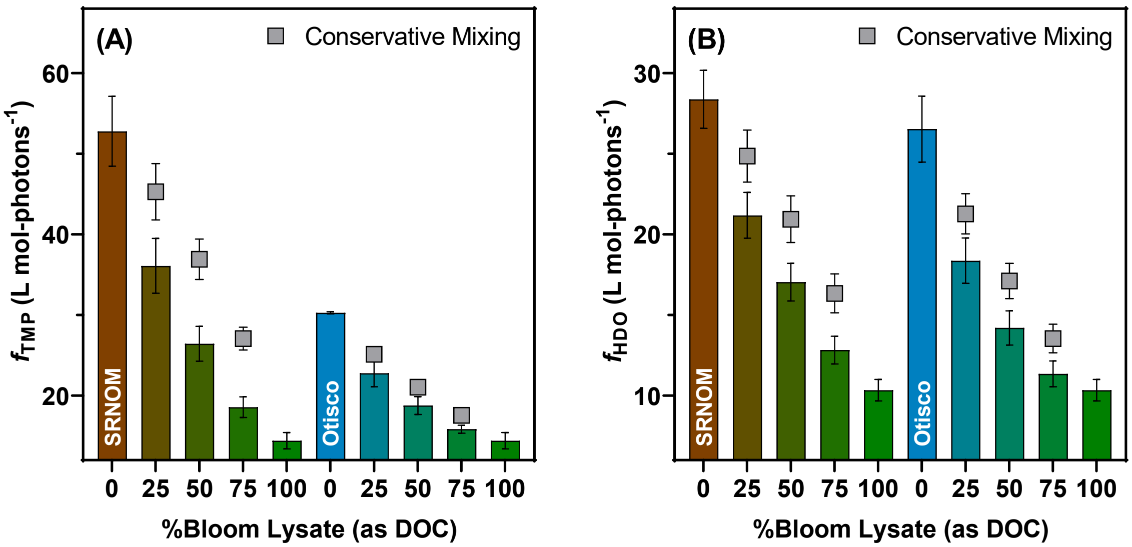

Figure S13. Changes in f_{TMP} and f_{HDO} upon mixing the lysates extracted from bloom samples A-E (recultivated until the stationary phase) with SRNOM or Otisco Lake water at different DOC ratios: **(A)** Comparison between f_{TMP} measured for the mixtures of bloom lysates with SRNOM or Otisco Lake water and f_{TMP} calculated assuming conservative mixing. **(B)** Comparison between f_{HDO} measured for the mixtures of bloom lysates with SRNOM or Otisco Lake water and f_{HDO} calculated assuming conservative mixing. Error bars represent the standard deviation of duplicate measurements for f_{TMP} and f_{HDO} or the 95% confidence interval for f_{TMP} and f_{HDO} calculated assuming conservative mixing; where absent, bars fall within symbols or bars. f_{TMP} and f_{HDO} profiles for the lysates extracted from bloom samples A-E were pooled for the clarity of presentation.

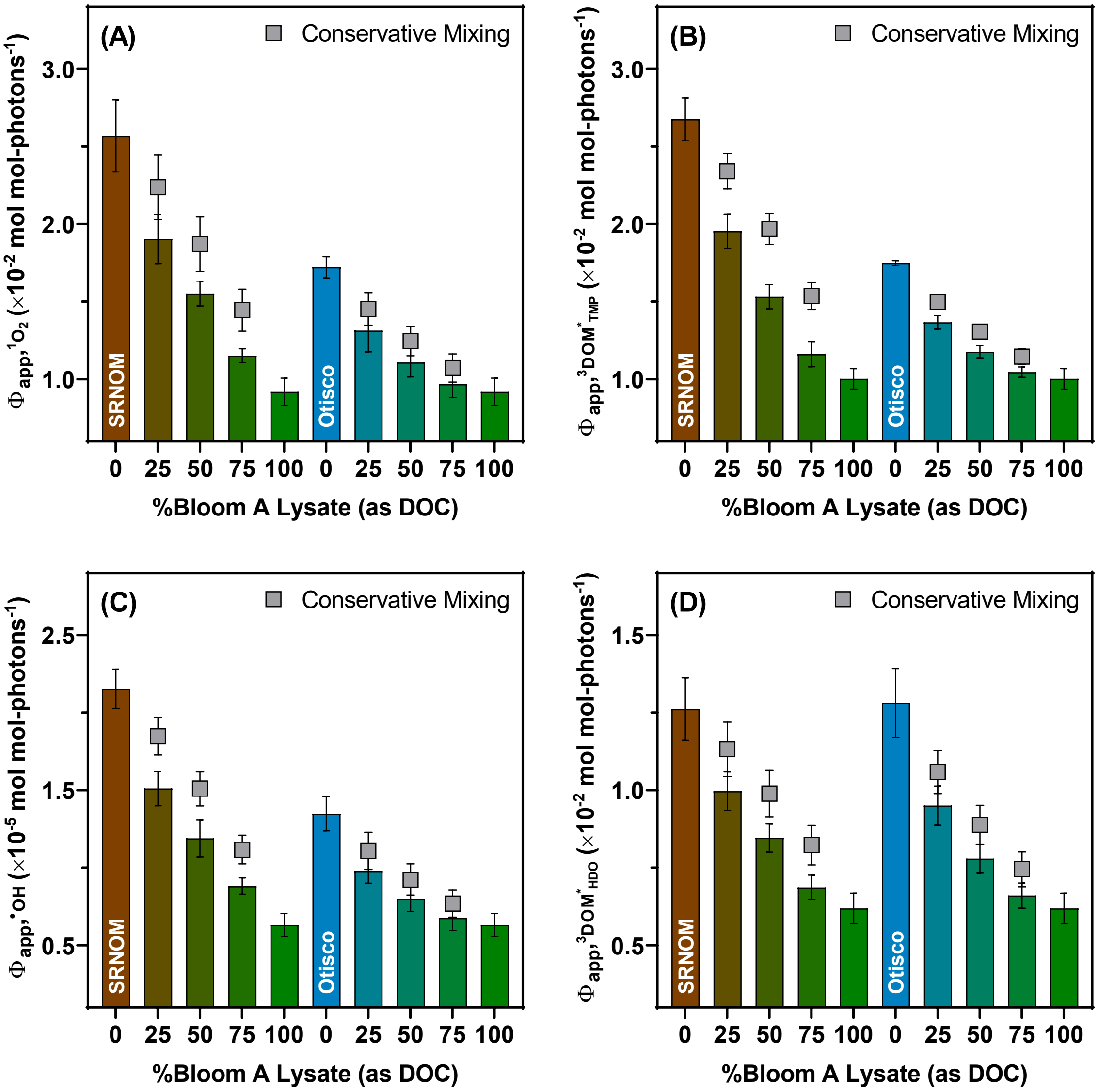

Figure S14. Changes in $\Phi_{app,RI}$ upon mixing the lysate extracted from bloom sample A (recultivated until the stationary phase) with SRNOM or Otisco Lake water at different DOC ratios: **(A)** Comparison between $\Phi_{app,\ {}^1O_2}$ measured for the mixtures of bloom A lysate with SRNOM or Otisco Lake water and $\Phi_{app,\ {}^1O_2}$ calculated assuming conservative mixing. **(B)** Comparison between $\Phi_{app,\ {}^3DOM^*_{TMP}}$ measured for the mixtures of bloom A lysate with SRNOM or Otisco Lake water and $\Phi_{app,\ {}^3DOM^*_{TMP}}$ calculated assuming conservative mixing. **(C)** Comparison between $\Phi_{app,\ {}^\cdot OH}$ measured for the mixtures of bloom A lysate with SRNOM or Otisco Lake water and $\Phi_{app,\ {}^\cdot OH}$ calculated assuming conservative mixing. **(D)** Comparison between $\Phi_{app,\ {}^3DOM^*_{HDO}}$ measured for the mixtures of bloom A lysate with SRNOM or Otisco Lake water and $\Phi_{app,\ {}^3DOM^*_{HDO}}$ calculated assuming conservative mixing. Error bars represent the standard deviation of duplicate measurements for $\Phi_{app,RI}$ or the 95% confidence interval for $\Phi_{app,RI}$ calculated assuming conservative mixing; where absent, bars fall within symbols.

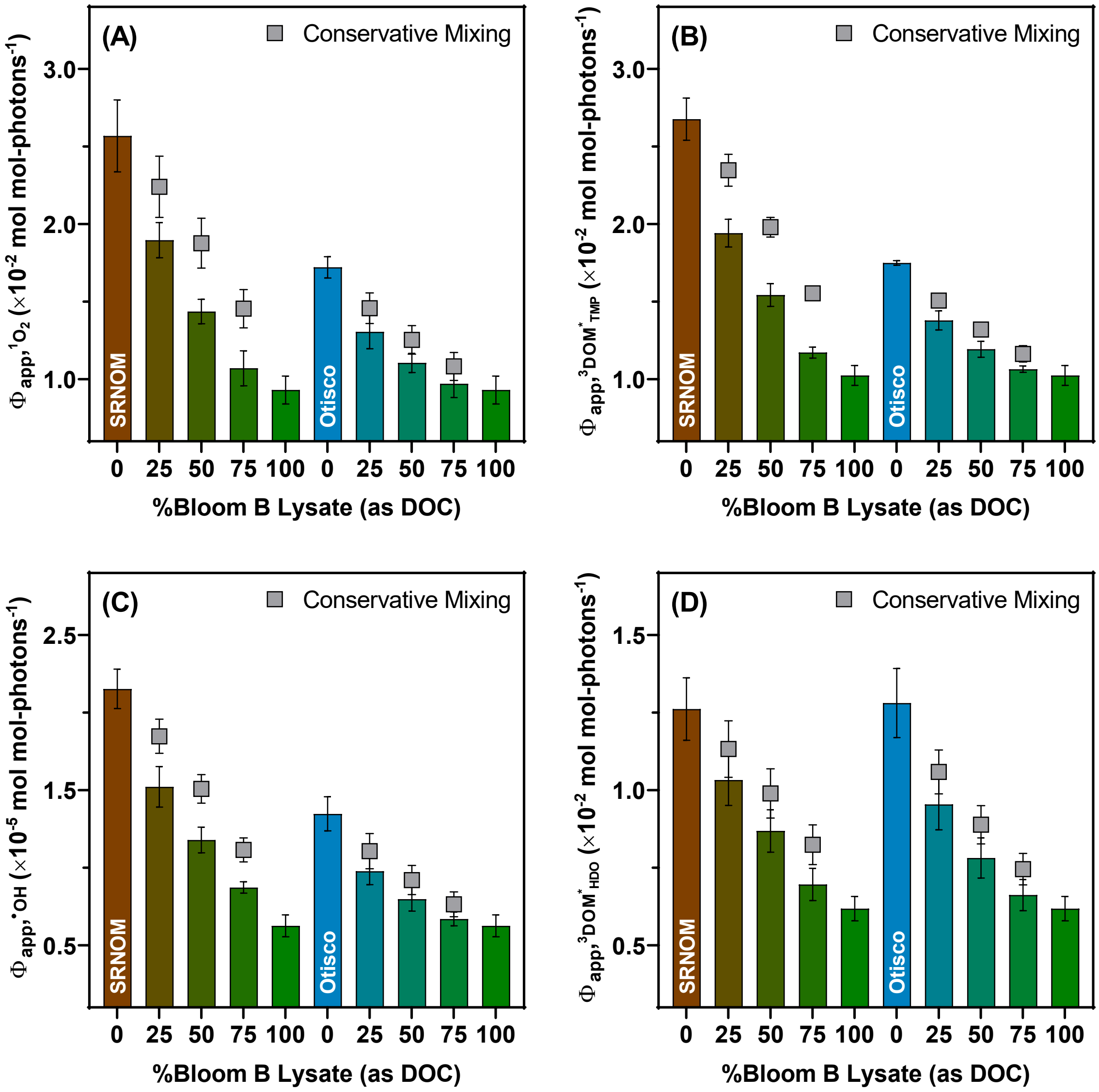

Figure S15. Changes in $\Phi_{app,RI}$ upon mixing the lysate extracted from bloom sample B (recultivated until the stationary phase) with SRNOM or Otisco Lake water at different DOC ratios: **(A)** Comparison between $\Phi_{app,\,^1O_2}$ measured for the mixtures of bloom B lysate with SRNOM or Otisco Lake water and $\Phi_{app,\,^1O_2}$ calculated assuming conservative mixing. **(B)** Comparison between $\Phi_{app,\,^3DOM^*_{TMP}}$ measured for the mixtures of bloom B lysate with SRNOM or Otisco Lake water and $\Phi_{app,\,^3DOM^*_{TMP}}$ calculated assuming conservative mixing. **(C)** Comparison between $\Phi_{app,\,^{\cdot}OH}$ measured for the mixtures of bloom B lysate with SRNOM or Otisco Lake water and $\Phi_{app,\,^{\cdot}OH}$ calculated assuming conservative mixing. **(D)** Comparison between $\Phi_{app,\,^3DOM^*_{HDO}}$ measured for the mixtures of bloom B lysate with SRNOM or Otisco Lake water and $\Phi_{app,\,^3DOM^*_{HDO}}$ calculated assuming conservative mixing. Error bars represent the standard deviation of duplicate measurements for $\Phi_{app,RI}$ or the 95% confidence interval for $\Phi_{app,RI}$ calculated assuming conservative mixing; where absent, bars fall within symbols.

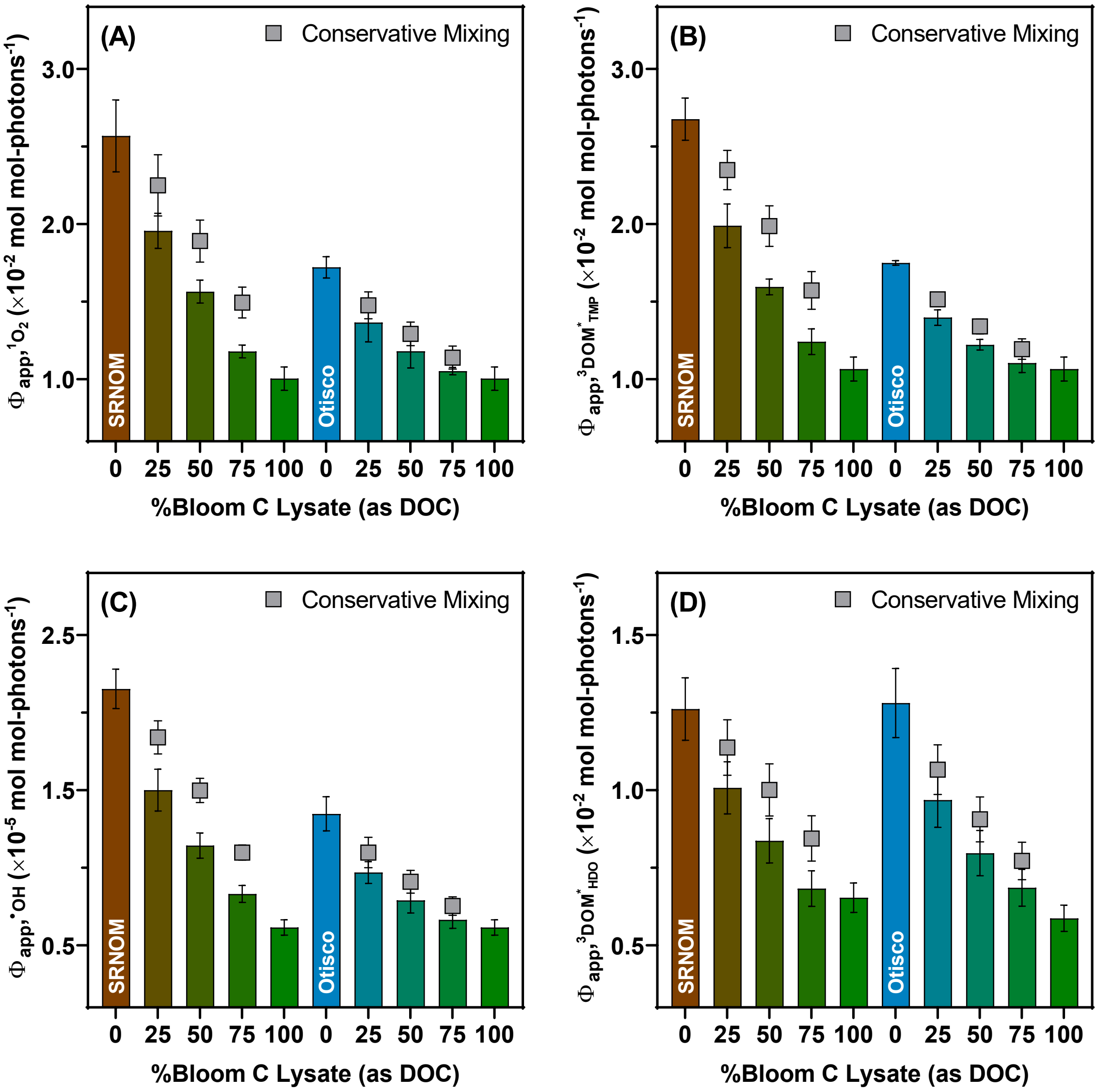

Figure S16. Changes in $\Phi_{app,RI}$ upon mixing the lysate extracted from bloom sample C (recultivated until the stationary phase) with SRNOM or Otisco Lake water at different DOC ratios: **(A)** Comparison between $\Phi_{app,\,^1O_2}$ measured for the mixtures of bloom C lysate with SRNOM or Otisco Lake water and $\Phi_{app,\,^1O_2}$ calculated assuming conservative mixing. **(B)** Comparison between $\Phi_{app,\,^3DOM^*_{TMP}}$ measured for the mixtures of bloom C lysate with SRNOM or Otisco Lake water and $\Phi_{app,\,^3DOM^*_{TMP}}$ calculated assuming conservative mixing. **(C)** Comparison between $\Phi_{app,\,\cdot OH}$ measured for the mixtures of bloom C lysate with SRNOM or Otisco Lake water and $\Phi_{app,\,\cdot OH}$ calculated assuming conservative mixing. **(D)** Comparison between $\Phi_{app,\,^3DOM^*_{HDO}}$ measured for the mixtures of bloom C lysate with SRNOM or Otisco Lake water and $\Phi_{app,\,^3DOM^*_{HDO}}$ calculated assuming conservative mixing. Error bars represent the standard deviation of duplicate measurements for $\Phi_{app,RI}$ or the 95% confidence interval for $\Phi_{app,RI}$ calculated assuming conservative mixing; where absent, bars fall within symbols.

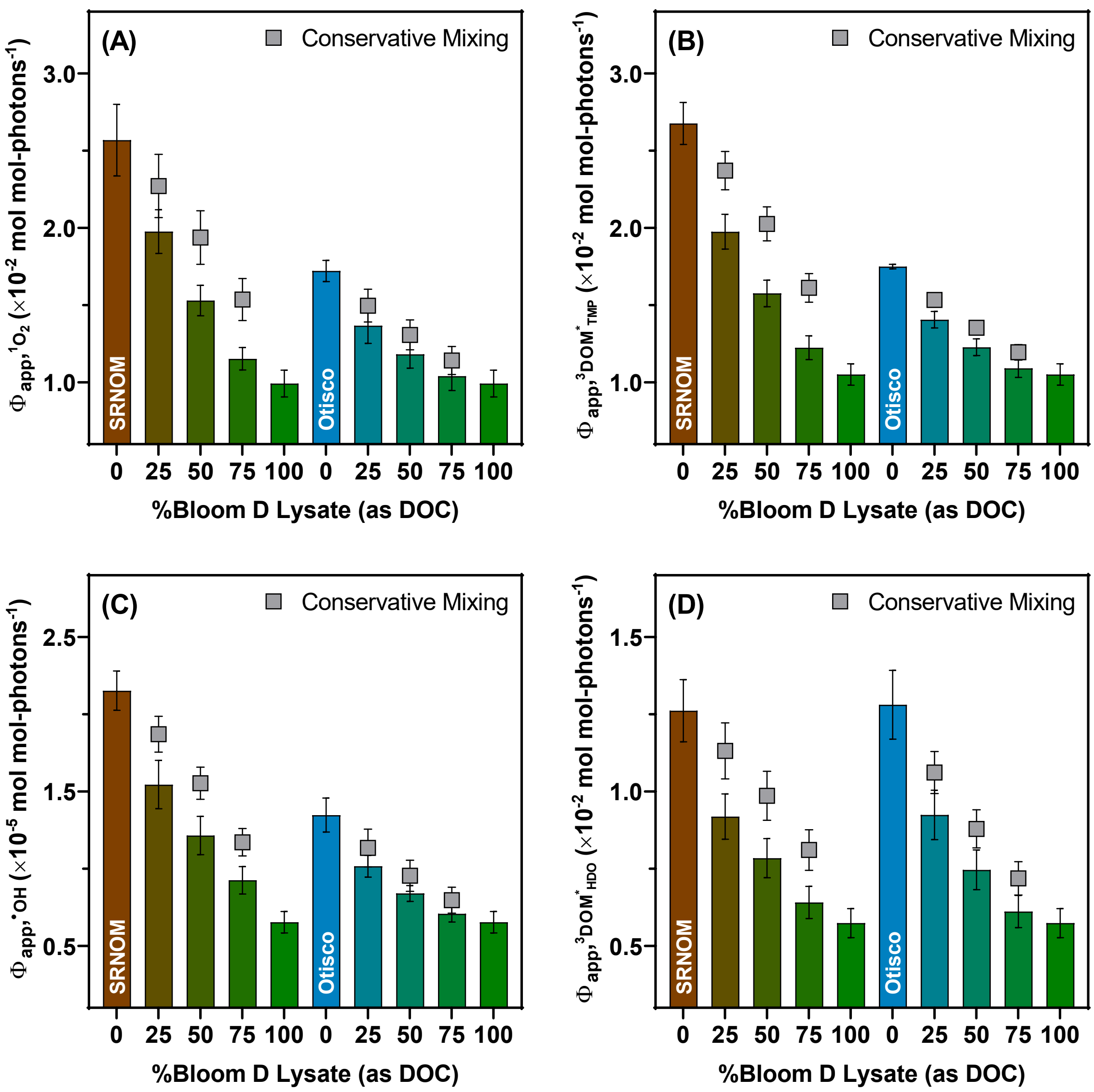

Figure S17. Changes in $\Phi_{app,RI}$ upon mixing the lysate extracted from bloom sample D (recultivated until the stationary phase) with SRNOM or Otisco Lake water at different DOC ratios: **(A)** Comparison between $\Phi_{app,\ {}^1O_2}$ measured for the mixtures of bloom D lysate with SRNOM or Otisco Lake water and $\Phi_{app,\ {}^1O_2}$ calculated assuming conservative mixing. **(B)** Comparison between $\Phi_{app,\ {}^3DOM^*_{TMP}}$ measured for the mixtures of bloom D lysate with SRNOM or Otisco Lake water and $\Phi_{app,\ {}^3DOM^*_{TMP}}$ calculated assuming conservative mixing. **(C)** Comparison between $\Phi_{app,\ {}^\cdot OH}$ measured for the mixtures of bloom D lysate with SRNOM or Otisco Lake water and $\Phi_{app,\ {}^\cdot OH}$ calculated assuming conservative mixing. **(D)** Comparison between $\Phi_{app,\ {}^3DOM^*_{HDO}}$ measured for the mixtures of bloom D lysate with SRNOM or Otisco Lake water and $\Phi_{app,\ {}^3DOM^*_{HDO}}$ calculated assuming conservative mixing. Error bars represent the standard deviation of duplicate measurements for $\Phi_{app,RI}$ or the 95% confidence interval for $\Phi_{app,RI}$ calculated assuming conservative mixing; where absent, bars fall within symbols.

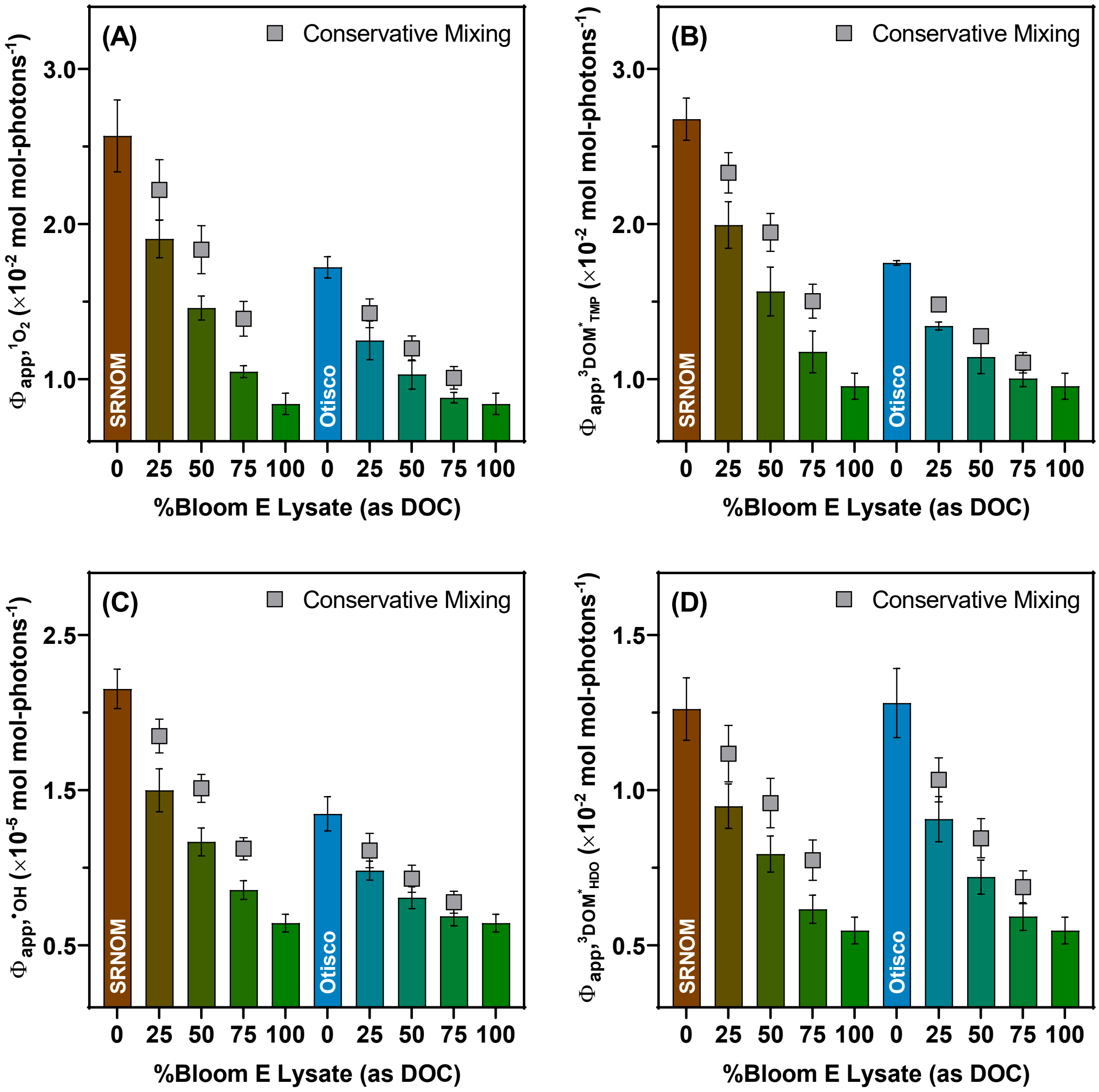

Figure S18. Changes in $\Phi_{app,RI}$ upon mixing the lysate extracted from bloom sample E (recultivated until the stationary phase) with SRNOM or Otisco Lake water at different DOC ratios: **(A)** Comparison between $\Phi_{app,\,^1O_2}$ measured for the mixtures of bloom E lysate with SRNOM or Otisco Lake water and $\Phi_{app,\,^1O_2}$ calculated assuming conservative mixing. **(B)** Comparison between $\Phi_{app,\,^3DOM^*_{TMP}}$ measured for the mixtures of bloom E lysate with SRNOM or Otisco Lake water and $\Phi_{app,\,^3DOM^*_{TMP}}$ calculated assuming conservative mixing. **(C)** Comparison between $\Phi_{app,\,\cdot OH}$ measured for the mixtures of bloom E lysate with SRNOM or Otisco Lake water and $\Phi_{app,\,\cdot OH}$ calculated assuming conservative mixing. **(D)** Comparison between $\Phi_{app,\,^3DOM^*_{HDO}}$ measured for the mixtures of bloom E lysate with SRNOM or Otisco Lake water and $\Phi_{app,\,^3DOM^*_{HDO}}$ calculated assuming conservative mixing. Error bars represent the standard deviation of duplicate measurements for $\Phi_{app,RI}$ or the 95% confidence interval for $\Phi_{app,RI}$ calculated assuming conservative mixing; where absent, bars fall within symbols.

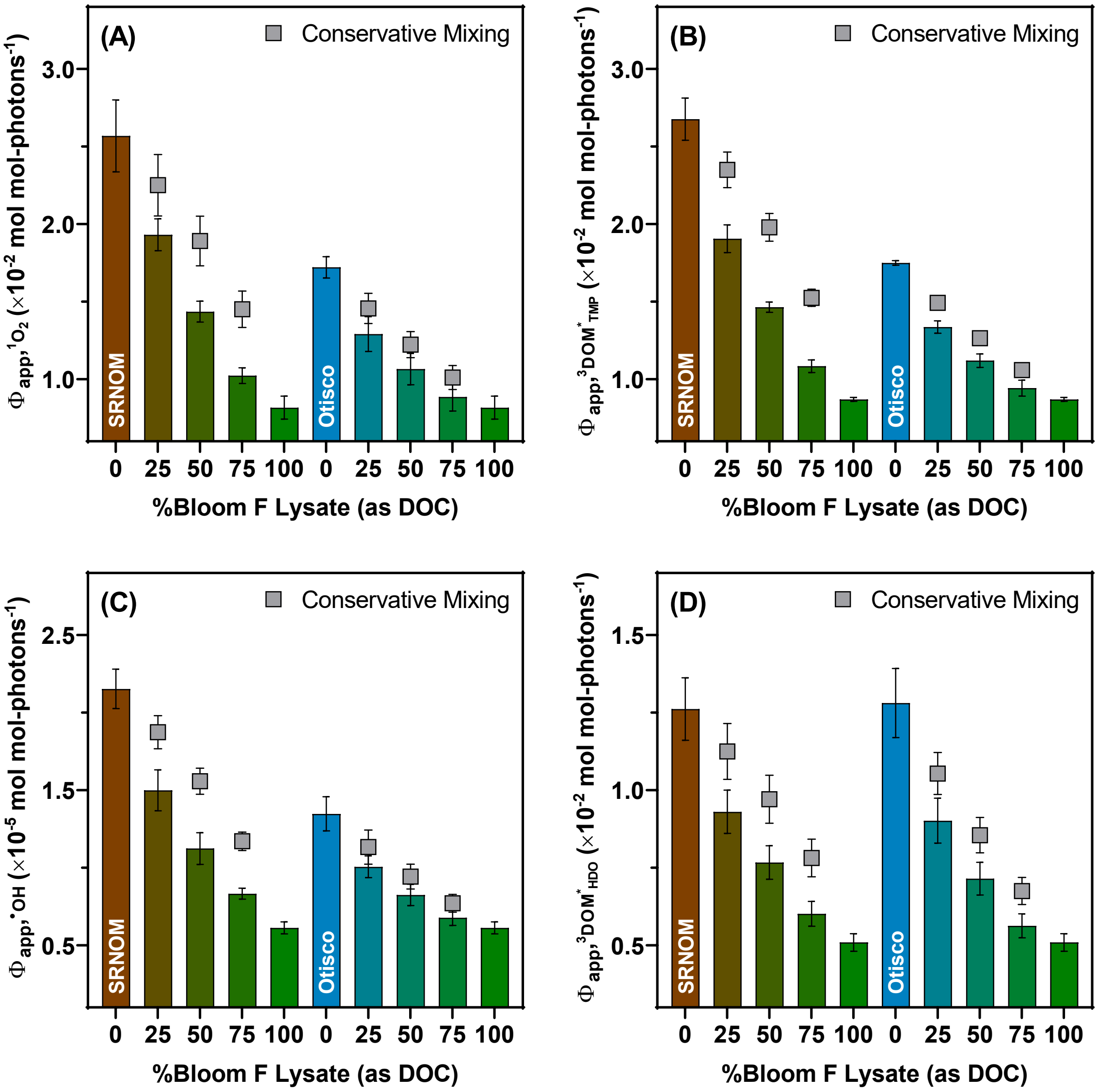

Figure S19. Changes in $\Phi_{app,RI}$ upon mixing the lysate extracted from bloom sample F (recultivated until the stationary phase) with SRNOM or Otisco Lake water at different DOC ratios: **(A)** Comparison between $\Phi_{app,\,^1O_2}$ measured for the mixtures of bloom F lysate with SRNOM or Otisco Lake water and $\Phi_{app,\,^1O_2}$ calculated assuming conservative mixing. **(B)** Comparison between $\Phi_{app,\,^3DOM^*_{TMP}}$ measured for the mixtures of bloom F lysate with SRNOM or Otisco Lake water and $\Phi_{app,\,^3DOM^*_{TMP}}$ calculated assuming conservative mixing. **(C)** Comparison between $\Phi_{app,\,\cdot OH}$ measured for the mixtures of bloom F lysate with SRNOM or Otisco Lake water and $\Phi_{app,\,\cdot OH}$ calculated assuming conservative mixing. **(D)** Comparison between $\Phi_{app,\,^3DOM^*_{HDO}}$ measured for the mixtures of bloom F lysate with SRNOM or Otisco Lake water and $\Phi_{app,\,^3DOM^*_{HDO}}$ calculated assuming conservative mixing. Error bars represent the standard deviation of duplicate measurements for $\Phi_{app,RI}$ or the 95% confidence interval for $\Phi_{app,RI}$ calculated assuming conservative mixing; where absent, bars fall within symbols.

18. Comparisons of $\Phi_{app,RI}$ for bloom supernatants

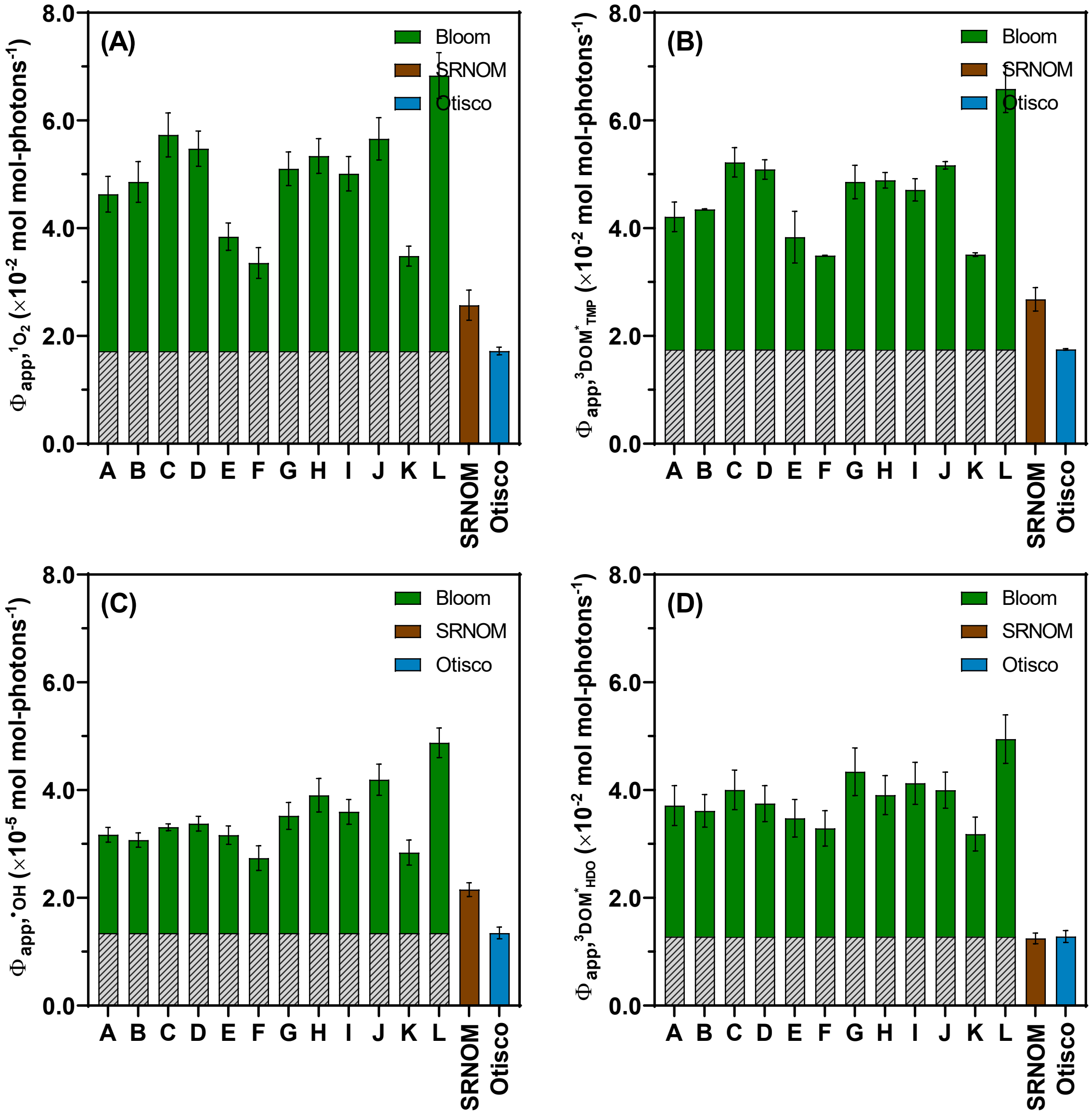

Figure S20. Comparisons of $\Phi_{app,RI}$ for the supernatants harvested from bloom samples recultivated in unfiltered Otisco Lake water until the stationary phase: **(A)** $\Phi_{app,\,{}^1O_2}$ for bloom supernatants (*n*=12; [DOC]=3.8±1.1 mg C/L; pH 7.2±0.2), SRNOM, and Otisco Lake water. **(B)** $\Phi_{app,\,{}^3DOM^*_{TMP}}$ for bloom supernatants, SRNOM, and Otisco Lake water. **(C)** $\Phi_{app,\,{}^\bullet OH}$ for bloom supernatants, SRNOM, and Otisco Lake water. **(D)** $\Phi_{app,\,{}^3DOM^*_{HDO}}$ for bloom supernatants, SRNOM, and Otisco Lake water. Error bars represent the standard deviations from duplicate measurements of $\Phi_{app,RI}$; where absent, bars fall within bars. Grey shaded bars represent the contribution of Otisco Lake water to $\Phi_{app,RI}$ for bloom supernatants.

19. Correlations between $\Phi_{app,RI}$ and %Chl-a_{cyano} for bloom supernatants

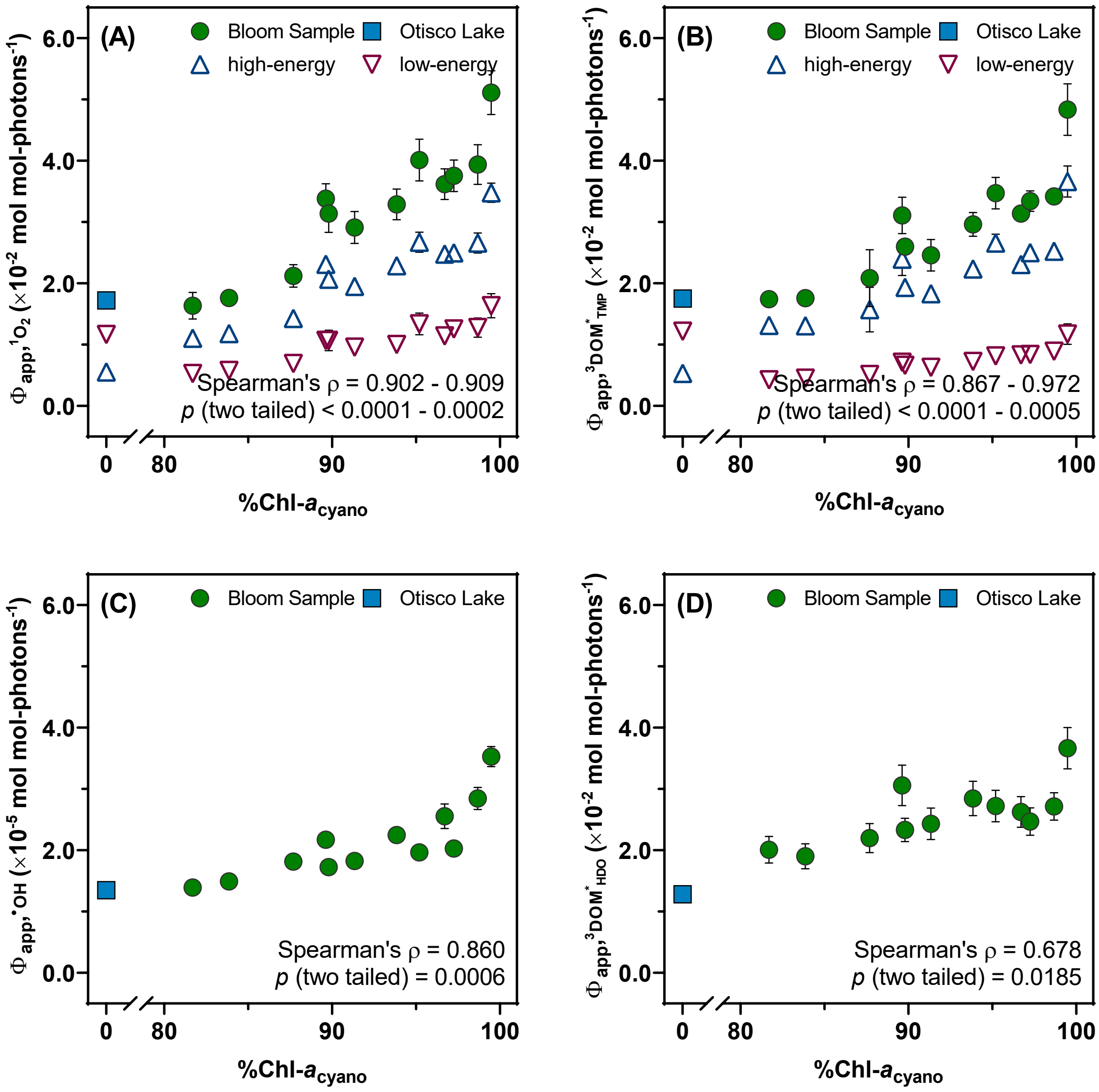

Figure S21. Correlations between $\Phi_{app,RI}$ (minus the contribution from Otisco Lake water) and the proportion of cyanobacterial chlorophyll *a* (Chl-a_{cyano}) in Chl-*a* (%Chl-a_{cyano}) for the supernatants harvested from bloom samples recultivated in unfiltered Otisco Lake water until the stationary phase: **(A)** Spearman's correlation between $\Phi_{app, ^1O_2}$ (attributable to $\Phi_{app, ^1O_2, high\text{-}energy}$ and $\Phi_{app, ^1O_2, low\text{-}energy}$) and %Chl-$a_{cyano}$ for bloom supernatants (n=12; [DOC]=3.8±1.1 mg C/L; pH 7.2±0.2). **(B)** Spearman's correlation between $\Phi_{app, ^3DOM^*_{TMP}}$ (attributable to $\Phi_{app, ^3DOM^*_{TMP}, high\text{-}energy}$ and $\Phi_{app, ^3DOM^*_{TMP}, low\text{-}energy}$) and %Chl-$a_{cyano}$ for bloom supernatants. **(C)** Spearman's correlation between $\Phi_{app, \cdot OH}$ and %Chl-a_{cyano} for bloom supernatants. **(D)** Spearman's correlation between $\Phi_{app, ^3DOM^*_{HDO}}$ and %Chl-a_{cyano} for bloom supernatants. Error bars represent the standard deviations from duplicate measurements of ratios; where absent, bars fall within symbols.

20. Correlations between $k_{TMP,\, ^3DOM^*_{TMP}}$ or $k_{t,t\text{-HDO},\, ^3DOM^*_{HDO}}$ and %Chl-a_{cyano} for bloom supernatants

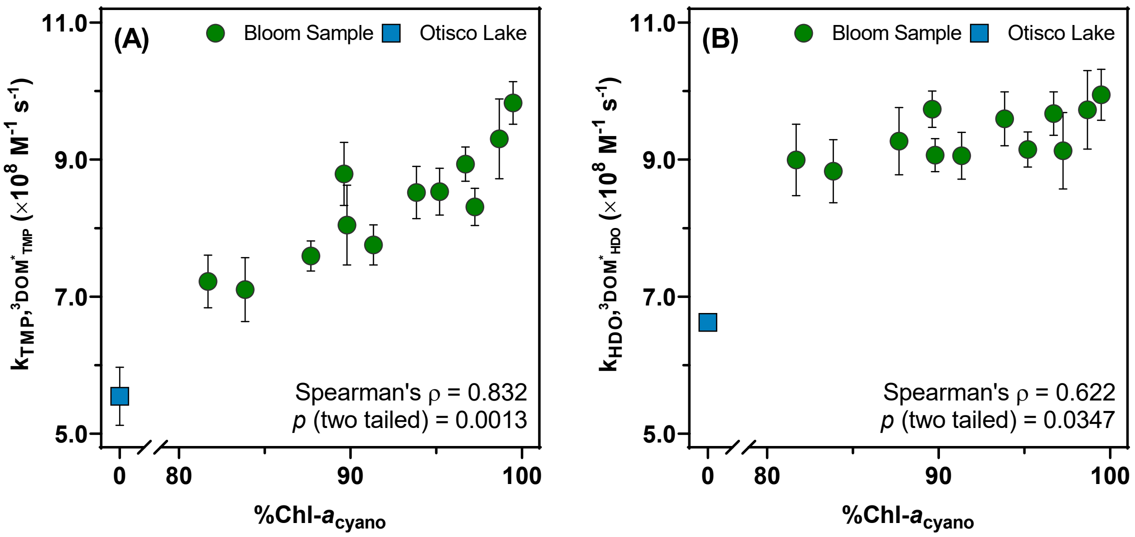

Figure S22. Correlations between $k_{TMP,\, ^3DOM^*_{TMP}}$ or $k_{t,t\text{-HDO},\, ^3DOM^*_{HDO}}$ and the proportion of cyanobacterial chlorophyll *a* (Chl-a_{cyano}) in Chl-*a* (%Chl-a_{cyano}) for the supernatants harvested from bloom samples recultivated in unfiltered Otisco Lake water until the stationary phase: **(A)** Spearman's correlation between the second-order reaction rate constants of TMP with $^3DOM^*$ ($k_{TMP,\, ^3DOM^*_{TMP}}$) and %Chl-a_{cyano} for bloom supernatants (n=12; [DOC]=3.8±1.1 mg C/L; pH 7.2±0.2). **(B)** Spearman's correlation between the second-order reaction rate constants of *t,t*-HDO with $^3DOM^*$ ($k_{t,t\text{-HDO},\, ^3DOM^*_{HDO}}$) and %Chl-$a_{cyano}$ for bloom supernatants. Error bars represent the standard deviations from duplicate measurements of $k_{TMP,\, ^3DOM^*_{TMP}}$ and $k_{t,t\text{-HDO},\, ^3DOM^*_{HDO}}$; where absent, bars fall within symbols.

21. Changes in $\Phi_{app,RI}$ with optical indices for bloom supernatants during recultivation

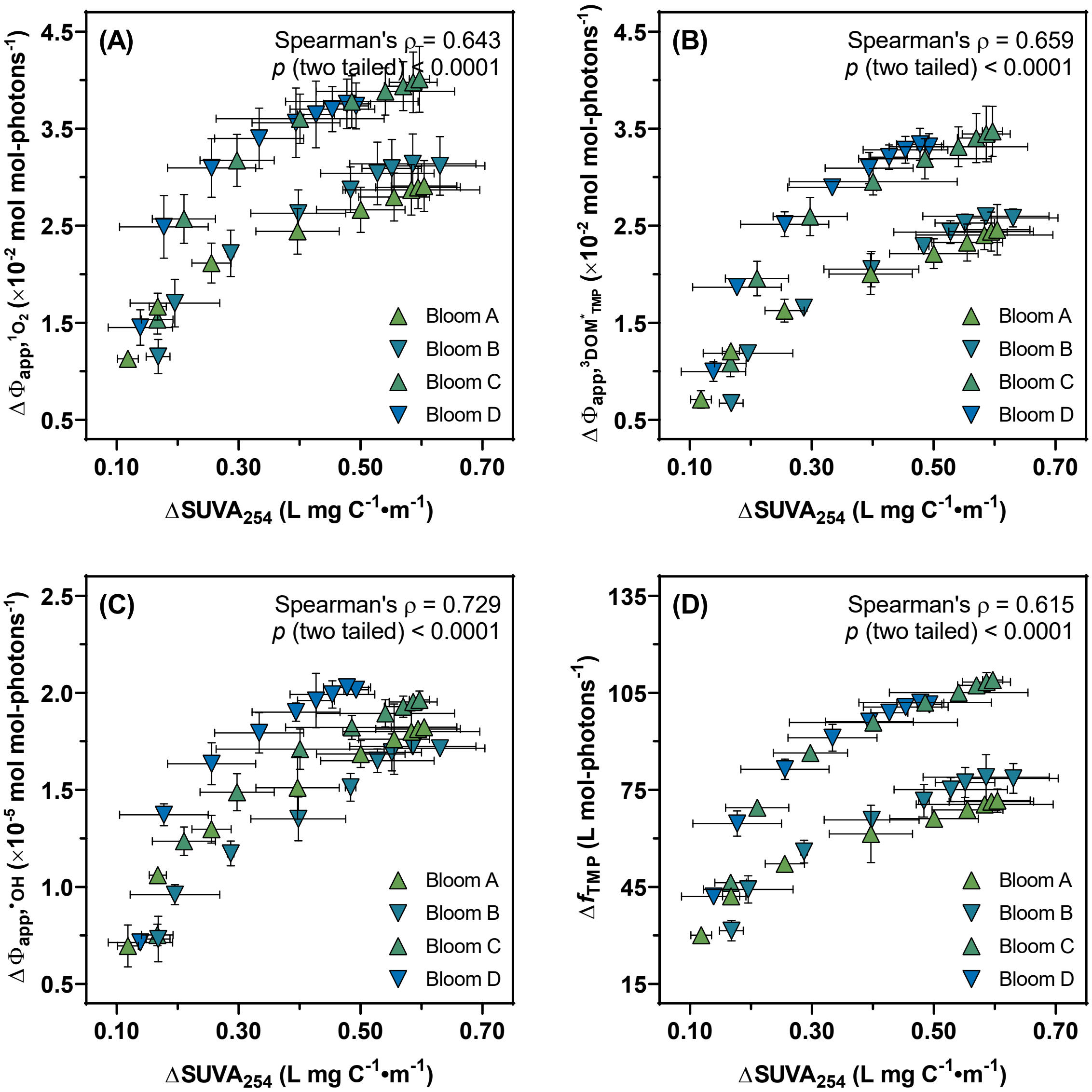

Figure S23. Changes in $\Phi_{app,RI}$ with $SUVA_{254}$ for the supernatants harvested from bloom samples A-D relative to changes in $\Phi_{app,RI}$ for Otisco Lake water with $SUVA_{254}$ over the course of recultivation: **(A)** Spearman's correlation between $\Delta\Phi_{app,\,^1O_2}$ and $\Delta SUVA_{254}$ for four sets of bloom supernatants (n=36; [DOC]=4.0±0.4 mg C/L; pH 7.1±0.1). **(B)** Spearman's correlation between $\Delta\Phi_{app,\,^3DOM^*_{TMP}}$ and $\Delta SUVA_{254}$ for four sets of bloom supernatants. **(C)** Spearman's correlation between $\Delta\Phi_{app,\,^\bullet OH}$ and $\Delta SUVA_{254}$ for four sets of bloom supernatants. **(D)** Spearman's correlation between Δf_{TMP} and $\Delta SUVA_{254}$ for four sets of bloom supernatants. Error bars represent the standard deviations from duplicate measurements of $\Phi_{app,RI}$, f_{TMP}, or $SUVA_{254}$; where absent, bars fall within symbols.

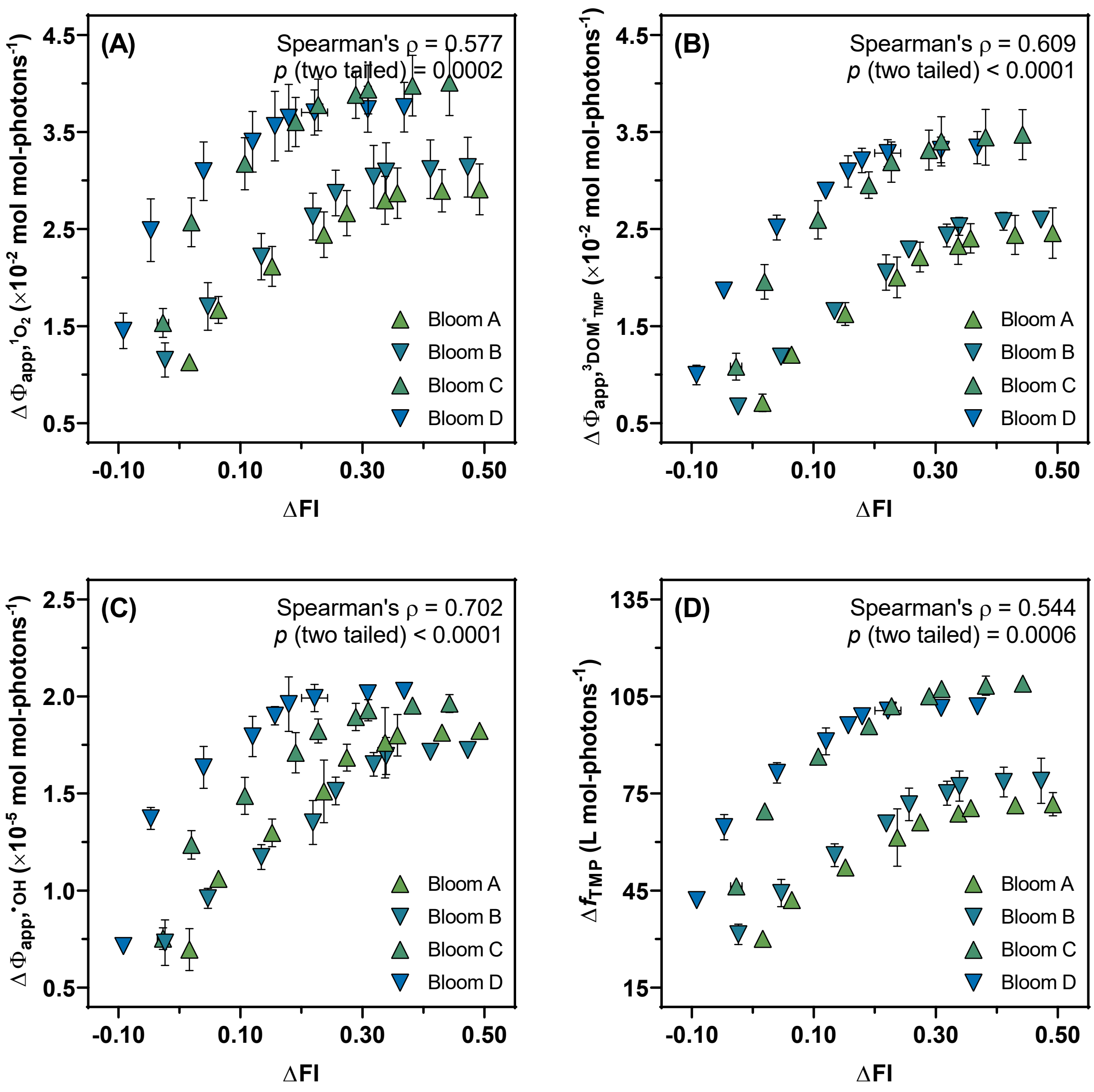

Figure S24. Changes in $\Phi_{app,RI}$ with fluorescence index (FI) for the supernatants harvested from bloom samples A-D relative to changes in $\Phi_{app,RI}$ for Otisco Lake water with FI over the course of recultivation: **(A)** Spearman's correlation between $\Delta\Phi_{app,\ ^1O_2}$ and ΔFI for four sets of bloom supernatants (*n*=36; [DOC]=4.0±0.4 mg C/L; pH 7.1±0.1). **(B)** Spearman's correlation between $\Delta\Phi_{app,\ ^3DOM^*_{TMP}}$ and ΔFI for four sets of bloom supernatants. **(C)** Spearman's correlation between $\Delta\Phi_{app,\ ^\bullet OH}$ and ΔFI for four sets of bloom supernatants. **(D)** Spearman's correlation between Δf_{TMP} and ΔFI for four sets of bloom supernatants. Error bars represent the standard deviations from duplicate measurements of $\Phi_{app,RI}$, f_{TMP}, or FI; where absent, bars fall within symbols.

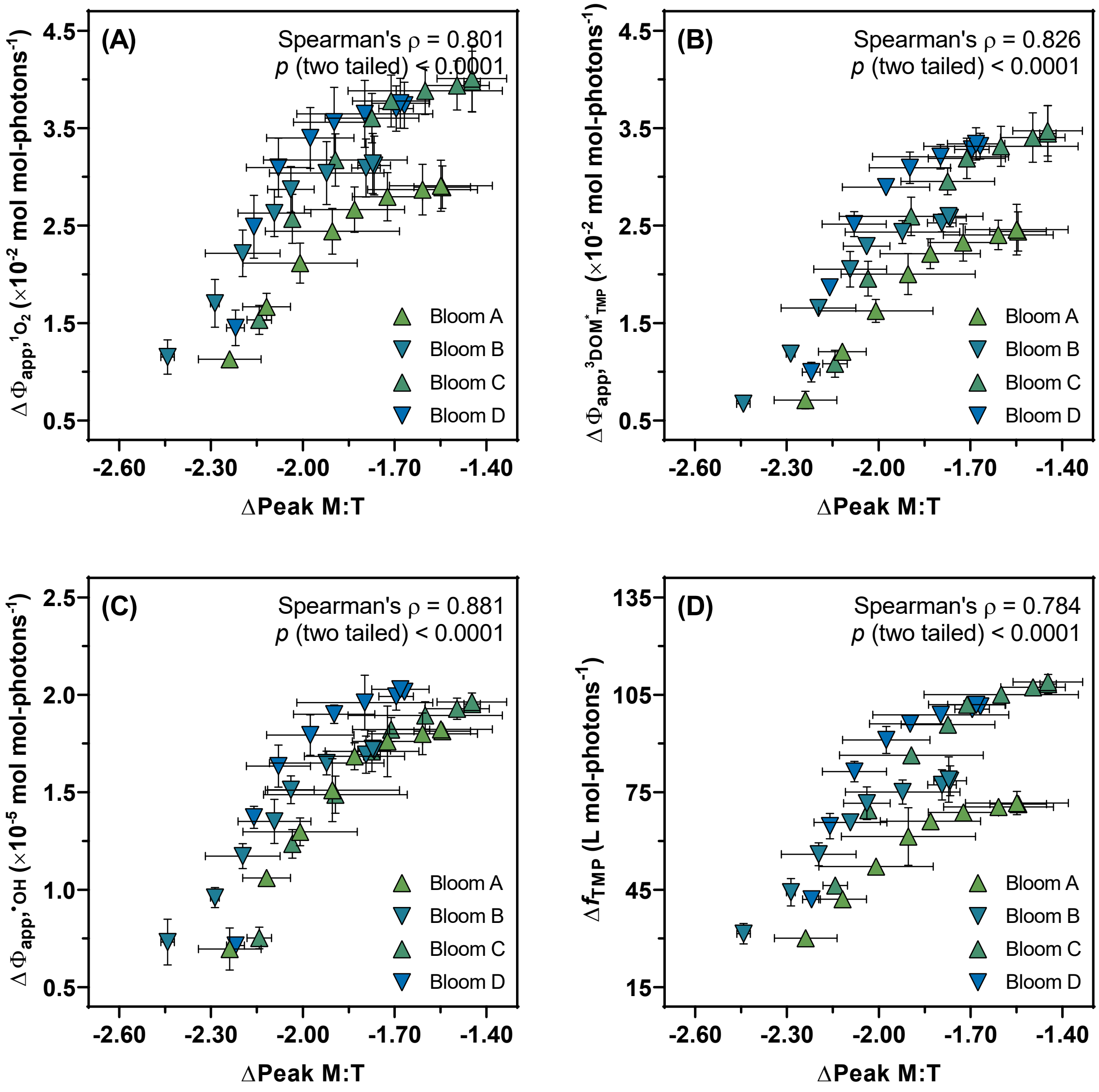

Figure S25. Changes in $\Phi_{app,RI}$ with the ratio of microbial humic-like to protein-like DOM fluorescence (peak M:T) for the supernatants harvested from bloom samples A-D relative to changes in $\Phi_{app,RI}$ for Otisco Lake water with peak M:T over the course of recultivation: **(A)** Spearman's correlation between $\Delta\Phi_{app,\,^1O_2}$ and Δpeak M:T for four sets of bloom supernatants (n=36; [DOC]=4.0±0.4 mg C/L; pH 7.1±0.1). **(B)** Spearman's correlation between $\Delta\Phi_{app,\,^3DOM^*_{TMP}}$ and Δpeak M:T for four sets of bloom supernatants. **(C)** Spearman's correlation between $\Delta\Phi_{app,\,^{\bullet}OH}$ and Δpeak M:T for four sets of bloom supernatants. **(D)** Spearman's correlation between Δf_{TMP} and Δpeak M:T for four sets of bloom supernatants. Error bars represent the standard deviations from duplicate measurements of $\Phi_{app,RI}$, f_{TMP}, or peak M:T; where absent, bars fall within symbols.

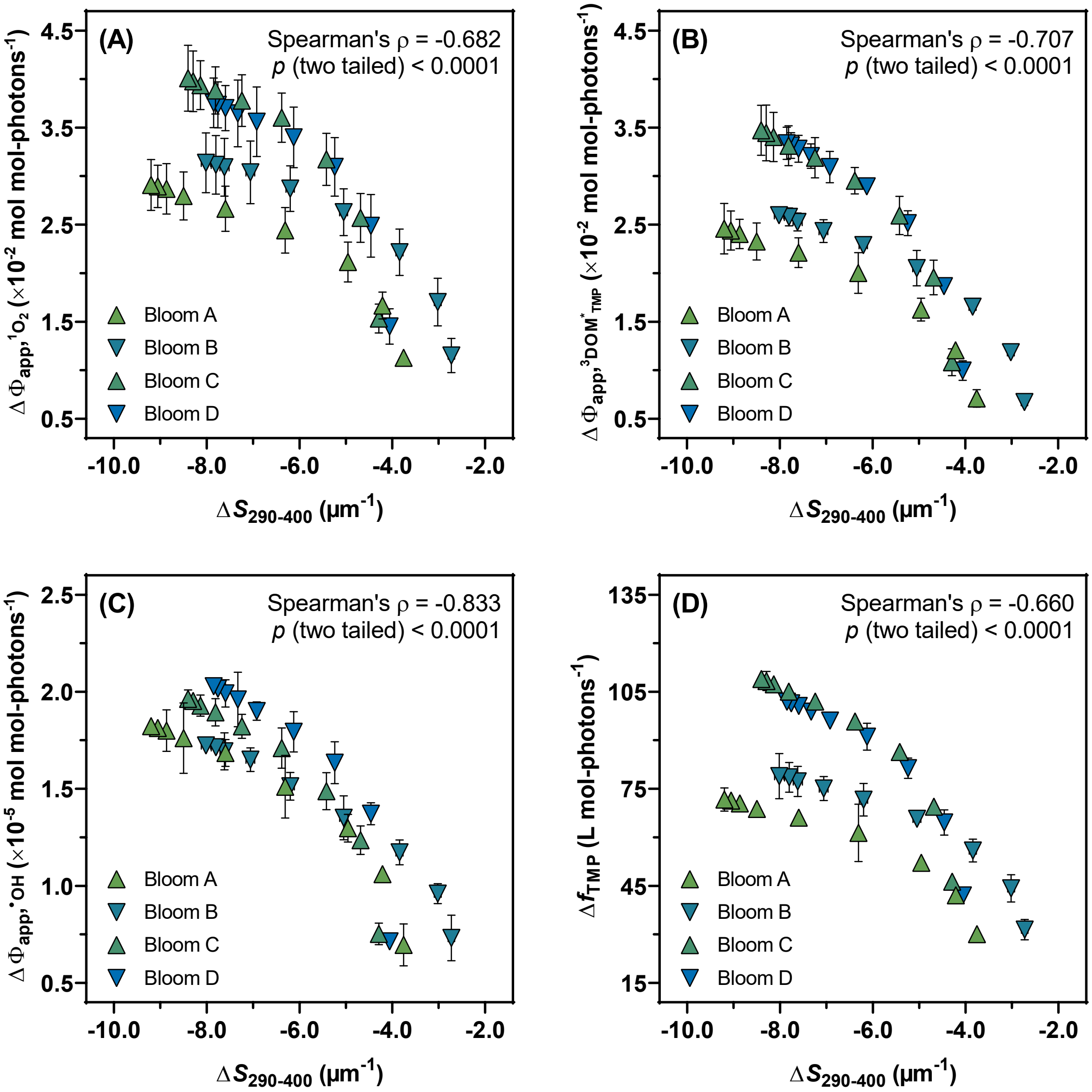

Figure S26. Changes in $\Phi_{app,RI}$ and the spectral slope coefficient from 290 to 400 nm ($S_{290-400}$) for the supernatants harvested from bloom samples A-D relative to changes in $\Phi_{app,RI}$ for Otisco Lake water with $S_{290-400}$ over the course of recultivation: **(A)** Spearman's correlation between $\Delta\Phi_{app,\,^1O_2}$ and $\Delta S_{290-400}$ for four sets of bloom supernatants (n=36; [DOC]=4.0±0.4 mg C/L; pH 7.1±0.1). **(B)** Spearman's correlation between $\Delta\Phi_{app,\,^3DOM^*_{TMP}}$ and $\Delta S_{290-400}$ for four sets of bloom supernatants. **(C)** Spearman's correlation between $\Delta\Phi_{app,\,^{\bullet}OH}$ and $\Delta S_{290-400}$ for four sets of bloom supernatants. **(D)** Spearman's correlation between Δf_{TMP} and $\Delta S_{290-400}$ for four sets of bloom supernatants. Error bars represent the standard deviations from duplicate measurements of $\Phi_{app,RI}$, f_{TMP}, or $S_{290-400}$; where absent, bars fall within symbols.

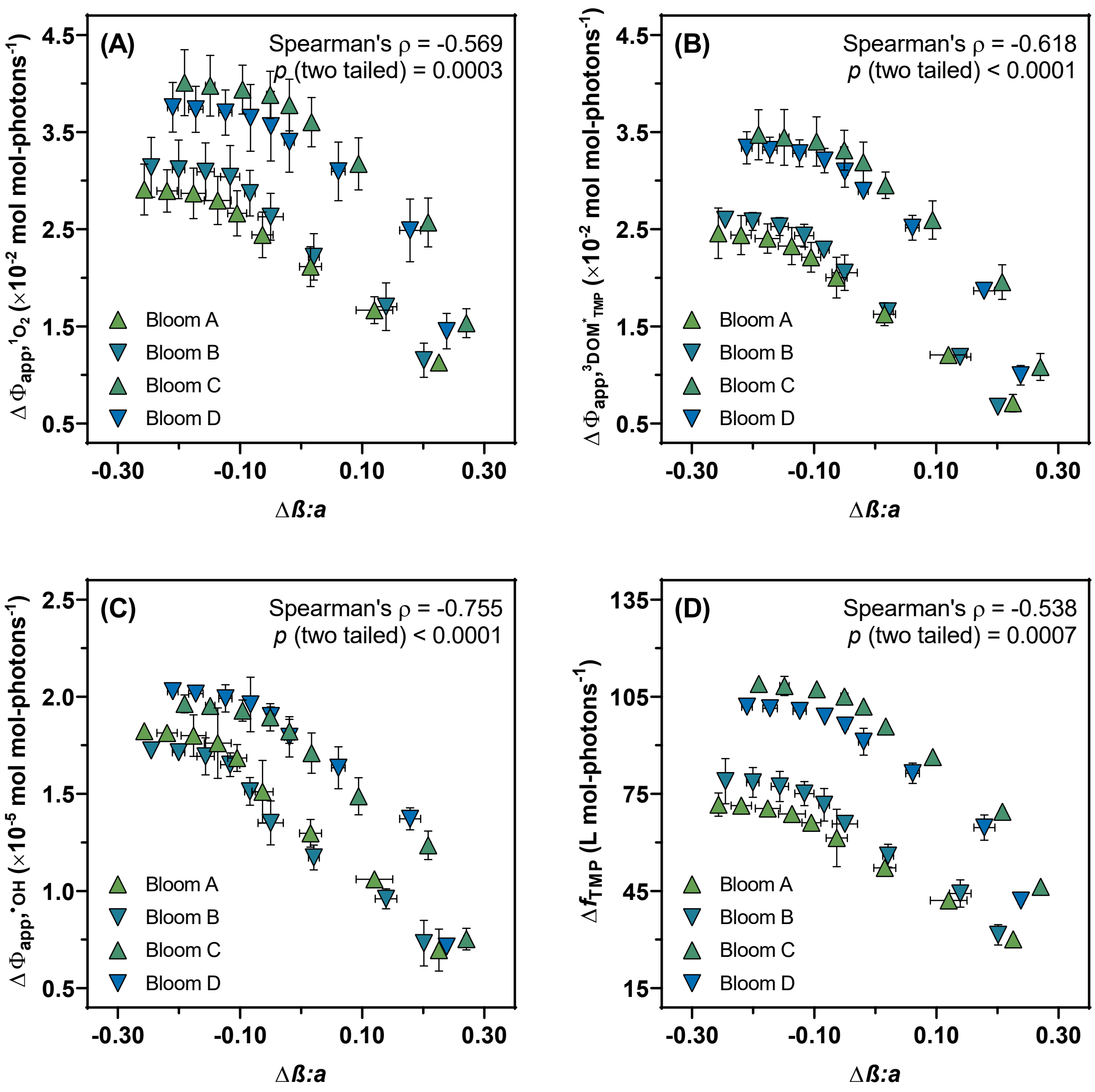

Figure S27. Changes in $\Phi_{app,RI}$ with freshness index (β:α) for the supernatants harvested from bloom samples A-D relative to changes in $\Phi_{app,RI}$ for Otisco Lake water with β:α over the course of recultivation: **(A)** Spearman's correlation between $\Delta\Phi_{app,\,^1O_2}$ and $\Delta\beta$:α for four sets of bloom supernatants (n=36; [DOC]=4.0±0.4 mg C/L; pH 7.1±0.1). **(B)** Spearman's correlation between $\Delta\Phi_{app,\,^3DOM^*_{TMP}}$ and $\Delta\beta$:α for four sets of bloom supernatants. **(C)** Spearman's correlation between $\Delta\Phi_{app,\,^{\bullet}OH}$ and $\Delta\beta$:α for four sets of bloom supernatants. **(D)** Spearman's correlation between Δf_{TMP} and $\Delta\beta$:α for four sets of bloom supernatants. Error bars represent the standard deviations from duplicate measurements of $\Phi_{app,RI}$, f_{TMP}, or β:α; where absent, bars fall within symbols.

22. Photolysis of protriptyline and fluridone in bloom supernatants under simulated sunlight conditions

Protriptyline underwent direct and indirect photolysis with varying pseudo-first-order rate constants in bloom supernatants, Otisco Lake water, and SRNOM solution (**Figure S28**), with indirect photolysis likely facilitated by electron transfer from its amine nitrogen atom to $^3DOM^*$ according to previous studies on structurally similar secondary amine-containing drugs.[105-107] Fluridone, on the other hand, primarily underwent direct photolysis as demonstrated by its comparable pseudo-first-order rate constants in different aqueous matrices (**Figure S29**), which agreed with prior work measuring similar photolysis rates in eutrophic lake water and distilled water.[108] However, a detailed investigation of the photolysis mechanisms and transformation products of protriptyline or fluridone was not within the scope of this study.

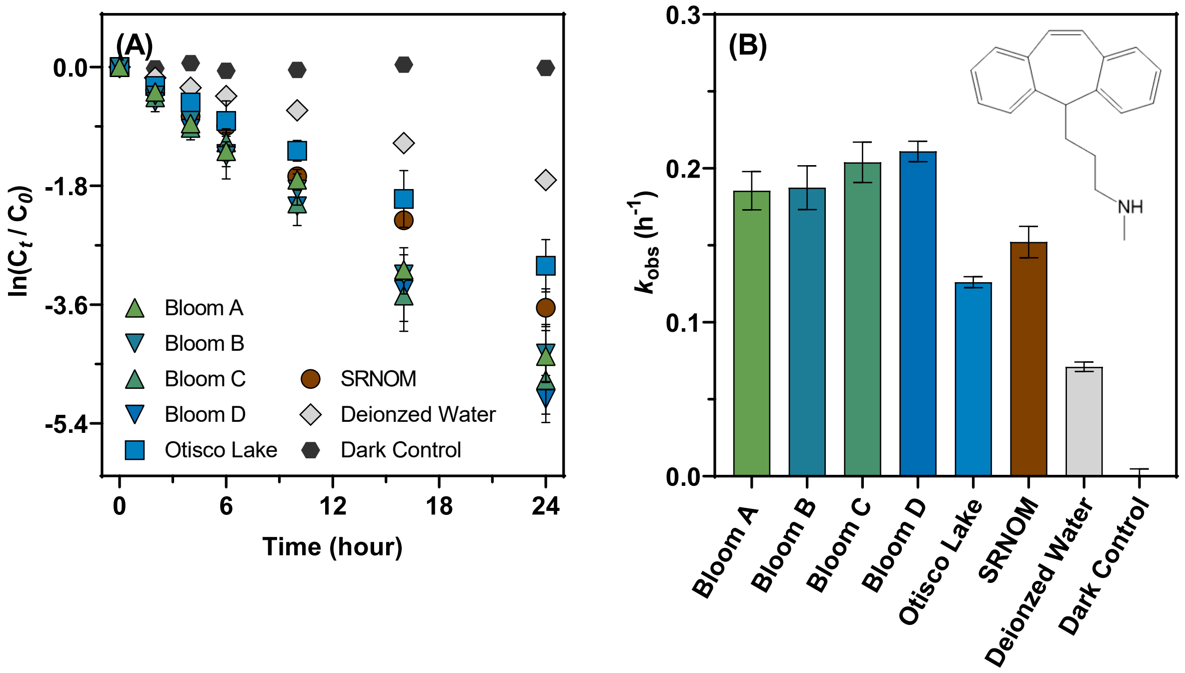

Figure S28. Photolysis kinetics of protriptyline in bloom supernatants, Otisco Lake water, and SRNOM solution under simulated sunlight conditions: **(A)** Profiles of protriptyline photolysis in the supernatants harvested from bloom samples A-D (recultivated in unfiltered Otisco Lake water until the stationary phase), Otisco Lake water (incubated under the same conditions as bloom samples), SRNOM solution, deionized water, and dark control. Error bars represent the standard deviations from duplicate measurements of protriptyline concentrations by online solid-phase extraction coupled with liquid chromatography-high-resolution mass spectrometry. **(B)** Comparison of the pseudo-first order rate constants for protriptyline photolysis (k_{obs}) measured in the supernatants harvested from bloom samples A-D, Otisco Lake water, SRNOM solution, deionized water, and dark control. Error bars represent the standard deviations from duplicate measurements of k_{obs}.

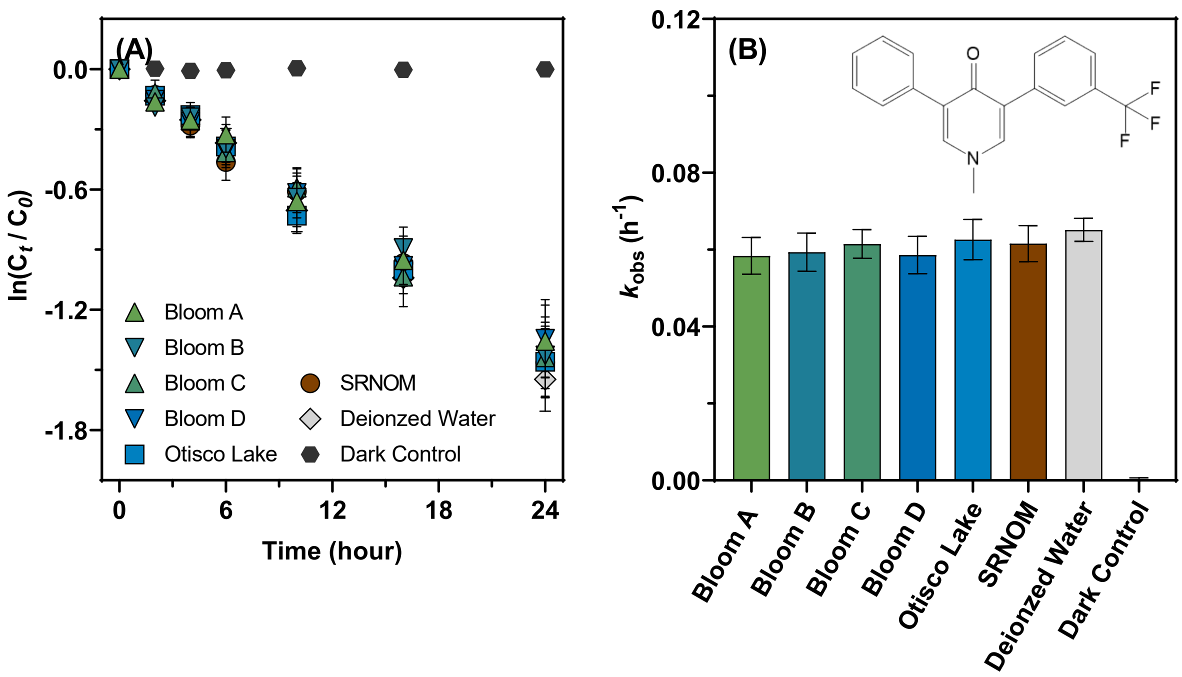

Figure S29. Photolysis kinetics of fluridone in bloom supernatants, Otisco Lake water, and SRNOM solution under simulated sunlight conditions: **(A)** Profiles of fluridone photolysis in the supernatants harvested from bloom samples A-D (recultivated in unfiltered Otisco Lake water until the stationary phase), Otisco Lake water (incubated under the same conditions as bloom samples), SRNOM solution, deionized water, and dark control. Error bars represent the standard deviations from duplicate measurements of fluridone concentrations by online solid-phase extraction coupled with liquid chromatography-high-resolution mass spectrometry. **(B)** Comparison of the pseudo-first order rate constants for fluridone photolysis (k_{obs}) measured in the supernatants harvested from bloom samples A-D, Otisco Lake water, SRNOM solution, deionized water, and dark control. Error bars represent the standard deviations from duplicate measurements of k_{obs}.

23. Summary of literature data on the apparent quantum yields of RIs

Table S37. Summary of literature $\Phi_{app,RI}$ data

Source	Sample ID	Sample Classification	Wavelength Range (nm)	$\Phi_{app,\ ^3DOM^*_{TMP}}$ ($\times10^{-2}$ mol mol-photons^{-1})	$\Phi_{app,\ ^1O_2}$ ($\times10^{-2}$ mol mol-photons^{-1})	$\Phi_{app,\ ^3DOM^*_{Sorbate}}$ ($\times10^{-2}$ mol mol-photons^{-1})	$\Phi_{app,\ ^{\bullet}OH}$ ($\times10^{-6}$ mol mol-photons^{-1})
Ref 109	SR-HPO	IHSS Isolate (SRFA)	300-400	NA	NA	NA	8.4
	MA-AOM	XAD Fraction (M. aeruginosa)	300-400	NA	NA	NA	21.8
	BG-AOM	XAD Fraction (Algae Lake)	300-400	NA	NA	NA	30.3
Ref 110	CK_1d	Whole Water (Paddy)	290-400	3.61	1.43	NA	18.2
	Biochar_1d	Whole Water (Paddy)	290-400	4.02	2.31	NA	12.7
	Straw_1d	Whole Water (Paddy)	290-400	0.53	1.48	NA	3.9
	Lime_1d	Whole Water (Paddy)	290-400	0.46	1.80	NA	6.2
	CK_10d	Whole Water (Paddy)	290-400	1.91	1.57	NA	1.8
	Biochar_10d	Whole Water (Paddy)	290-400	1.01	0.93	NA	1.1
	Straw_10d	Whole Water (Paddy)	290-400	1.79	1.83	NA	4.4
	Lime_10d	Whole Water (Paddy)	290-400	3.21	2.36	NA	3.9
	CK_20d	Whole Water (Paddy)	290-400	0.71	1.58	NA	2.9
	Biochar_20d	Whole Water (Paddy)	290-400	0.73	2.47	NA	3.1
	Straw_20d	Whole Water (Paddy)	290-400	0.98	2.86	NA	22.6
	Lime_20d	Whole Water (Paddy)	290-400	2.03	5.72	NA	15.2
	CK_30d	Whole Water (Paddy)	290-400	0.64	1.43	NA	1.0
	Biochar_30d	Whole Water (Paddy)	290-400	0.55	1.03	NA	0.8
	Straw_30d	Whole Water (Paddy)	290-400	0.81	1.46	NA	4.0
	Lime_30d	Whole Water (Paddy)	290-400	1.49	3.30	NA	6.3
	CK_45d	Whole Water (Paddy)	290-400	0.49	1.53	NA	3.8
	Biochar_45d	Whole Water (Paddy)	290-400	1.45	2.20	NA	2.1
	Straw_45d	Whole Water (Paddy)	290-400	2.10	3.47	NA	19.8
	Lime_45d	Whole Water (Paddy)	290-400	3.34	7.28	NA	20.2
	CK_60d	Whole Water (Paddy)	290-400	0.13	1.31	NA	4.1
	Biochar_60d	Whole Water (Paddy)	290-400	0.24	2.03	NA	4.8
	Straw_60d	Whole Water (Paddy)	290-400	0.79	1.73	NA	12.2
	Lime_60d	Whole Water (Paddy)	290-400	0.45	2.50	NA	12.5
Ref 111	ADOM_Bulk	WEOM (Algae Lake)	290-600	4.43	2.21	NA	466.4
	ADOM_HMW	WEOM+UF Fraction (Algae Lake)	290-600	1.56	1.52	NA	307.6
	ADOM_LMW	WEOM+UF Fraction (Algae Lake)	290-600	9.02	3.18	NA	639.5
	MDOM_Bulk	WEOM (Microphyte Lake)	290-600	0.70	0.74	NA	713.5
	MDOM_HMW	WEOM+UF Fraction (Microphyte Lake)	290-600	0.15	0.19	NA	485.0
	MDOM_LMW	WEOM+UF Fraction (Microphyte Lake)	290-600	1.43	1.37	NA	1142.0
	SRHA_Bulk	IHSS Isolate (SRHA)	290-600	2.04	1.28	NA	480.2
	SRHA_HMW	UF Fraction (SRHA)	290-600	1.53	1.13	NA	371.4
	SRHA_LMW	UF Fraction (SRHA)	290-600	4.34	3.02	NA	1196.2
Ref 112	I1_<1 kDa	UF Fraction (M. aeruginosa)	290-400	16.54	25.21	NA	608.4
	I2_1–5 kDa	UF Fraction (M. aeruginosa)	290-400	7.31	14.28	NA	228.2
	I3_5–10 kDa	UF Fraction (M. aeruginosa)	290-400	5.47	9.92	NA	128.4

Table S37. Summary of literature $\Phi_{app,RI}$ data (continued)							
Source	Sample ID	Sample Classification	Wavelength Range (nm)	$\Phi_{app,\ ^3DOM^*_{TMP}}$ (×10^{-2} mol mol-photons^{-1})	$\Phi_{app,\ ^1O_2}$ (×10^{-2} mol mol-photons^{-1})	$\Phi_{app,\ ^3DOM^*_{Sorbate}}$ (×10^{-2} mol mol-photons^{-1})	$\Phi_{app,\ ^{\cdot}OH}$ (×10^{-6} mol mol-photons^{-1})
Ref 112	I4_10–30 kDa	UF Fraction (M. aeruginosa)	290-400	6.58	15.93	NA	155.4
	I5_30–100 kDa	UF Fraction (M. aeruginosa)	290-400	3.47	18.64	NA	176.0
	I6_>100 kDa	UF Fraction (M. aeruginosa)	290-400	0.66	9.10	NA	75.8
	I0_bulk	UF Fraction (M. aeruginosa)	290-400	1.43	17.50	NA	166.6
	SRFA	IHSS Isolate (SRFA)	290-400	0.30	2.15	NA	17.2
Ref 113	Henan Changsheng Industrial Fulvic acid (FA)	XAD Fraction (Soil)	290-400	3.03	2.82	NA	12.8
	FA + NaClO	XAD Fraction (Soil, Cl_2)	290-400	4.42	4.04	NA	30.0
	FA + UV_{254}	XAD Fraction (Soil, UV)	290-400	1.88	2.15	NA	29.3
	FA + O_3	XAD Fraction (Soil, O_3)	290-400	1.99	2.42	NA	16.4
	Aldrich humic acid (HA)	XAD Fraction (Soil)	290-400	2.10	1.69	NA	23.2
	HA + NaClO	XAD Fraction (Soil, Cl_2)	290-400	3.38	3.20	NA	88.0
	HA + UV_{254}	XAD Fraction (Soil, UV)	290-400	1.70	1.53	NA	57.1
	HA + O_3	XAD Fraction (Soil, O_3)	290-400	1.94	1.62	NA	81.8
	EfOM	PPL Extract (WWTP Effluent)	290-400	4.88	4.11	NA	102.5
	EfOM + NaClO	PPL Extract (WWTP Effluent, Cl_2)	290-400	6.41	5.08	NA	162.2
	EfOM + UV_{254}	PPL Extract (WWTP Effluent, UV)	290-400	5.87	4.84	NA	158.3
	EfOM + O_3	PPL Extract (WWTP Effluent, O_3)	290-400	5.02	4.70	NA	207.8
	WW	Whole Water (WWTP Effluent)	290-400	5.57	4.94	NA	807.7
	WW + NaClO	Whole Water (WWTP Effluent, Cl_2)	290-400	12.80	10.16	NA	1849.4
	WW + UV_{254}	Whole Water (WWTP Effluent, UV)	290-400	7.56	7.01	NA	2166.0
	WW + O_3	Whole Water (WWTP Effluent, O_3)	290-400	9.10	7.04	NA	1943.6
Ref 114	Scindapsus aureus 265 nm	C18+PPL Extract (Root Exudate)	280-410	6.06	6.63	NA	548.5
	Scindapsus aureus 275 nm	C18+PPL Extract (Root Exudate)	280-410	10.94	10.52	NA	528.4
	Scindapsus aureus 285 nm	C18+PPL Extract (Root Exudate)	280-410	6.91	8.25	NA	455.2
	Scindapsus aureus 295 nm	C18+PPL Extract (Root Exudate)	280-410	4.83	7.28	NA	202.7
	Scindapsus aureus 305 nm	C18+PPL Extract (Root Exudate)	280-410	1.23	3.58	NA	95.5
	Scindapsus aureus 335 nm	C18+PPL Extract (Root Exudate)	280-410	0.60	2.90	NA	62.6
	Scindapsus aureus 365 nm	C18+PPL Extract (Root Exudate)	280-410	0.60	0.59	NA	15.9
	Scindapsus aureus 390 nm	C18+PPL Extract (Root Exudate)	280-410	0.51	0.57	NA	5.3
	Scindapsus aureus 400 nm	C18+PPL Extract (Root Exudate)	280-410	0.33	0.33	NA	2.1
	Pistia stratiotes 265 nm	C18+PPL Extract (Root Exudate)	280-410	8.66	10.10	NA	478.5
	Pistia stratiotes 275 nm	C18+PPL Extract (Root Exudate)	280-410	12.52	15.18	NA	507.2
	Pistia stratiotes 285 nm	C18+PPL Extract (Root Exudate)	280-410	9.35	11.01	NA	441.4
	Pistia stratiotes 295 nm	C18+PPL Extract (Root Exudate)	280-410	6.06	8.29	NA	245.1
	Pistia stratiotes 305 nm	C18+PPL Extract (Root Exudate)	280-410	2.19	4.08	NA	123.1
	Pistia stratiotes 335 nm	C18+PPL Extract (Root Exudate)	280-410	1.56	3.58	NA	65.8
	Pistia stratiotes 365 nm	C18+PPL Extract (Root Exudate)	280-410	1.63	1.31	NA	19.1
	Pistia stratiotes 390 nm	C18+PPL Extract (Root Exudate)	280-410	1.16	1.10	NA	8.5
	Pistia stratiotes 400 nm	C18+PPL Extract (Root Exudate)	280-410	0.81	0.79	NA	5.3
	Eichhornia crassipes 265 nm	C18+PPL Extract (Root Exudate)	280-410	3.17	6.47	NA	539.7
	Eichhornia crassipes 275 nm	C18+PPL Extract (Root Exudate)	280-410	6.28	7.74	NA	507.8
	Eichhornia crassipes 285 nm	C18+PPL Extract (Root Exudate)	280-410	2.97	5.90	NA	505.7

Table S37. Summary of literature $\Phi_{app,RI}$ data (continued)							
Source	Sample ID	Sample Classification	Wavelength Range (nm)	$\Phi_{app,\ ^3DOM^*_{TMP}}$ (×10^{-2} mol mol-photons^{-1})	$\Phi_{app,\ ^1O_2}$ (×10^{-2} mol mol-photons^{-1})	$\Phi_{app,\ ^3DOM^*_{Sorbate}}$ (×10^{-2} mol mol-photons^{-1})	$\Phi_{app,\ ^{\cdot}OH}$ (×10^{-6} mol mol-photons^{-1})
Ref 114	Eichhornia crassipes 295 nm	C18+PPL Extract (Root Exudate)	280-410	1.85	3.97	NA	324.0
	Eichhornia crassipes 305 nm	C18+PPL Extract (Root Exudate)	280-410	0.95	2.99	NA	153.0
	Eichhornia crassipes 335 nm	C18+PPL Extract (Root Exudate)	280-410	0.86	2.33	NA	96.7
	Eichhornia crassipes 365 nm	C18+PPL Extract (Root Exudate)	280-410	1.20	1.45	NA	59.5
	Eichhornia crassipes 390 nm	C18+PPL Extract (Root Exudate)	280-410	0.79	1.26	NA	40.4
	Eichhornia crassipes 400 nm	C18+PPL Extract (Root Exudate)	280-410	0.63	0.93	NA	17.0
	Nymphaea tetragona 265 nm	C18+PPL Extract (Root Exudate)	280-410	1.14	4.83	NA	323.6
	Nymphaea tetragona 275 nm	C18+PPL Extract (Root Exudate)	280-410	1.87	5.57	NA	292.8
	Nymphaea tetragona 285 nm	C18+PPL Extract (Root Exudate)	280-410	0.91	4.33	NA	145.4
	Nymphaea tetragona 295 nm	C18+PPL Extract (Root Exudate)	280-410	0.97	3.62	NA	100.8
	Nymphaea tetragona 305 nm	C18+PPL Extract (Root Exudate)	280-410	0.30	1.42	NA	23.3
	Nymphaea tetragona 335 nm	C18+PPL Extract (Root Exudate)	280-410	0.27	1.26	NA	22.3
	Nymphaea tetragona 365 nm	C18+PPL Extract (Root Exudate)	280-410	0.41	0.90	NA	4.2
	Nymphaea tetragona 390 nm	C18+PPL Extract (Root Exudate)	280-410	0.35	0.72	NA	3.2
	Nymphaea tetragona 400 nm	C18+PPL Extract (Root Exudate)	280-410	0.18	0.45	NA	2.1
Ref 115	Flood	Whole Water (Lake)	290-600	1.64	1.01	NA	404.0
	Drought	Whole Water (Lake)	290-600	1.14	6.66	NA	310.0
Ref 116	DOM_{ACS}	WEOM (Bio-Stabilization Sludge)	300-700	0.39	0.52	NA	21.3
	DOM_{ADS}	WEOM (Bio-Stabilization Sludge)	300-700	0.12	0.52	NA	4.8
Ref 117	DBC	DBC (pyDOM)	300-400	6.70	5.90	NA	40.0
	LDOC	WEOM (Biochar)	300-400	2.00	1.70	NA	160.0
Ref 118	EfOM	C18 Extract (WWTP Effluent, O_3)	300-600	1.18	2.01	NA	40.4
	EfOM + 0.29 g O_3/g TOC	C18 Extract (WWTP Effluent, O_3)	300-600	1.04	1.87	NA	48.4
	EfOM + 0.53 g O_3/g TOC	C18 Extract (WWTP Effluent, O_3)	300-600	0.94	1.63	NA	60.1
	EfOM + 1.05 g O_3/g TOC	C18 Extract (WWTP Effluent, O_3)	300-600	0.90	1.85	NA	22.2
	EfOM+ 1.92 g O_3/g TOC	C18 Extract (WWTP Effluent, O_3)	300-600	0.87	1.31	NA	46.1
	Aldrich humic acid (HA)	XAD Fraction (Soil)	300-600	0.48	1.13	NA	0.5
	HA + 0.29 g O_3/g TOC	XAD Fraction (Soil, O_3)	300-600	0.36	0.73	NA	1.3
	HA + 0.53 g O_3/g TOC	XAD Fraction (Soil, O_3)	300-600	0.34	0.59	NA	1.8
	HA + 1.05 g O_3/g TOC	XAD Fraction (Soil, O_3)	300-600	0.37	0.52	NA	3.2
	HA + 1.92 g O_3/g TOC	XAD Fraction (Soil, O_3)	300-600	0.29	0.45	NA	2.3
	Henan Changsheng Industrial Fulvic acid (FA)	XAD Fraction (Soil)	300-600	0.66	5.05	NA	0.5
	FA + 0.29 g O_3/g TOC	XAD Fraction (Soil, O_3)	300-600	0.49	3.52	NA	1.8
	FA + 0.53 g O_3/g TOC	XAD Fraction (Soil, O_3)	300-600	0.41	2.83	NA	2.1
	FA + 1.05 g O_3/g TOC	XAD Fraction (Soil, O_3)	300-600	0.33	1.92	NA	3.3
	FA + 1.92 g O_3/g TOC	XAD Fraction (Soil, O_3)	300-600	0.31	1.37	NA	4.8
Ref 119	1_Iwanai	Whole Water (Reservoir)	280-400	NA	2.84	NA	NA
	2_Kanayama	Whole Water (Reservoir)	280-400	NA	2.98	NA	NA
	3_Katsurazawa	Whole Water (Reservoir)	280-400	NA	2.00	NA	NA
	4_Tokachi	Whole Water (Reservoir)	280-400	NA	1.89	NA	NA
	5_Tokisato	Whole Water (Reservoir)	280-400	NA	3.23	NA	NA
	6_Chūbetsu	Whole Water (Reservoir)	280-400	NA	2.11	NA	NA

Table S37. Summary of literature $\Phi_{app,RI}$ data (continued)

Source	Sample ID	Sample Classification	Wavelength Range (nm)	$\Phi_{app,\ ^3DOM^*_{TMP}}$ ($\times10^{-2}$ mol mol-photons^{-1})	$\Phi_{app,\ ^1O_2}$ ($\times10^{-2}$ mol mol-photons^{-1})	$\Phi_{app,\ ^3DOM^*_{Sorbate}}$ ($\times10^{-2}$ mol mol-photons^{-1})	$\Phi_{app,\ ^{\bullet}OH}$ ($\times10^{-6}$ mol mol-photons^{-1})
Ref 119	7_Shijūshida	Whole Water (Reservoir)	280-400	NA	2.45	NA	NA
	8_Sagae	Whole Water (Reservoir)	280-400	NA	2.30	NA	NA
	9_Naruko	Whole Water (Reservoir)	280-400	NA	2.10	NA	NA
	10_Kamafusa	Whole Water (Reservoir)	280-400	NA	1.99	NA	NA
	11_Shichikashuku	Whole Water (Reservoir)	280-400	NA	1.77	NA	NA
	12_Gassan	Whole Water (Reservoir)	280-400	NA	2.25	NA	NA
	13_Surikamigawa	Whole Water (Reservoir)	280-400	NA	1.67	NA	NA
	14_Futase	Whole Water (Reservoir)	280-400	NA	2.15	NA	NA
	15_Ikari	Whole Water (Reservoir)	280-400	NA	2.65	NA	NA
	16_Kawamata	Whole Water (Reservoir)	280-400	NA	3.14	NA	NA
	17_Miyagase	Whole Water (Reservoir)	280-400	NA	3.91	NA	NA
	18_Sonohara	Whole Water (Reservoir)	280-400	NA	1.63	NA	NA
	19_Fujiwara	Whole Water (Reservoir)	280-400	NA	1.46	NA	NA
	20_Naramata	Whole Water (Reservoir)	280-400	NA	1.63	NA	NA
	21_Yagisawa	Whole Water (Reservoir)	280-400	NA	2.07	NA	NA
	22_Shimokubo	Whole Water (Reservoir)	280-400	NA	6.21	NA	NA
	23_Ōishi	Whole Water (Reservoir)	280-400	NA	1.78	NA	NA
	24_Ōkawa	Whole Water (Reservoir)	280-400	NA	3.07	NA	NA
	25_Ōmachi	Whole Water (Reservoir)	280-400	NA	3.81	NA	NA
	26_Yawagi	Whole Water (Reservoir)	280-400	NA	2.23	NA	NA
	27_Yokoyama	Whole Water (Reservoir)	280-400	NA	2.25	NA	NA
	28_Nagashima	Whole Water (Reservoir)	280-400	NA	4.11	NA	NA
	29_Misogawa	Whole Water (Reservoir)	280-400	NA	3.07	NA	NA
	30_Tokuyama	Whole Water (Reservoir)	280-400	NA	3.26	NA	NA
	31_Takayama	Whole Water (Reservoir)	280-400	NA	2.38	NA	NA
	32_Shorenji	Whole Water (Reservoir)	280-400	NA	1.93	NA	NA
	33_Hinachi	Whole Water (Reservoir)	280-400	NA	2.11	NA	NA
	34_Hitokura	Whole Water (Reservoir)	280-400	NA	3.11	NA	NA
	35_Tomata	Whole Water (Reservoir)	280-400	NA	1.79	NA	NA
	36_Sugesawa	Whole Water (Reservoir)	280-400	NA	2.26	NA	NA
	37_Haji	Whole Water (Reservoir)	280-400	NA	2.53	NA	NA
	38_Hattabara	Whole Water (Reservoir)	280-400	NA	3.68	NA	NA
	39_Ikeda	Whole Water (Reservoir)	280-400	NA	2.52	NA	NA
	40_Sameura	Whole Water (Reservoir)	280-400	NA	1.70	NA	NA
	41_Shingu	Whole Water (Reservoir)	280-400	NA	2.45	NA	NA
	42_Tomisato	Whole Water (Reservoir)	280-400	NA	2.04	NA	NA
	43_Ōdo	Whole Water (Reservoir)	280-400	NA	5.91	NA	NA
	44_Nagayasuguchi	Whole Water (Reservoir)	280-400	NA	1.79	NA	NA
	45_Tsuruda	Whole Water (Reservoir)	280-400	NA	2.17	NA	NA
	46_Shimouke	Whole Water (Reservoir)	280-400	NA	2.38	NA	NA
	47_Yabakei	Whole Water (Reservoir)	280-400	NA	2.05	NA	NA
	48_Ryūmon	Whole Water (Reservoir)	280-400	NA	1.84	NA	NA

**Table S37.** Summary of literature $\Phi_{app,RI}$ data (continued)							
Source	Sample ID	Sample Classification	Wavelength Range (nm)	$\Phi_{app, ^3DOM^*_{TMP}}$ ($\times 10^{-2}$ mol mol-photons^{-1})	$\Phi_{app, ^1O_2}$ ($\times 10^{-2}$ mol mol-photons^{-1})	$\Phi_{app, ^3DOM^*_{Sorbate}}$ ($\times 10^{-2}$ mol mol-photons^{-1})	$\Phi_{app, ^{\bullet}OH}$ ($\times 10^{-6}$ mol mol-photons^{-1})
Ref 119	49_Haneji	Whole Water (Reservoir)	280-400	NA	2.82	NA	NA
	50_Arakawa	Whole Water (Reservoir)	280-400	NA	1.94	NA	NA
	L1_Shinobazuike	Whole Water (Lake)	280-400	NA	2.63	NA	NA
	L2_Senzokuike	Whole Water (Lake)	280-400	NA	3.52	NA	NA
	L3_Suwako	Whole Water (Lake)	280-400	NA	3.52	NA	NA
	L4_Inbanuma	Whole Water (Lake)	280-400	NA	3.93	NA	NA
	L5_Teganuma	Whole Water (Lake)	280-400	NA	3.24	NA	NA
	L6_Ushikunuma	Whole Water (Lake)	280-400	NA	4.99	NA	NA
	L7_Kasumigaura	Whole Water (Lake)	280-400	NA	2.95	NA	NA
	SRNOM	IHSS Isolate (SRNOM)	280-400	NA	2.00	NA	NA
	SRFA	IHSS Isolate (SRFA)	280-400	NA	1.44	NA	NA
	UMRNOM	IHSS Isolate (UMRNOM)	280-400	NA	1.92	NA	NA
Ref 120	Crystal Bog	Whole Water (Lake)	315-415	0.43	1.40	NA	80.0
	Trout Bog	Whole Water (Lake)	315-415	0.54	1.10	NA	120.0
	Allequash Lake	Whole Water (Lake)	315-415	0.77	2.10	NA	46.0
	Big Muskellunge Lake	Whole Water (Lake)	315-415	0.73	3.30	NA	50.0
	Crystal Lake	Whole Water (Lake)	315-415	0.45	2.50	NA	40.0
	Sparkling Lake	Whole Water (Lake)	315-415	0.98	2.50	NA	34.0
	Trout Lake	Whole Water (Lake)	315-415	0.59	2.80	NA	32.0
	Memories	Whole Water (River)	315-415	1.63	7.30	NA	45.0
	Olsen Ditch	Whole Water (Agricultural Ditch)	315-415	2.04	4.57	NA	99.0
	Wammer Ditch	Whole Water (Agricultural Ditch)	315-415	1.54	4.04	NA	55.0
	Seven-Mile Creek	Whole Water (River)	315-415	1.47	4.32	NA	34.0
	WRRF Pre-Cl	Whole Water (WWTP Effluent)	315-415	1.37	3.00	NA	48.0
	WRRF Reuse	Whole Water (WWTP Effluent, Cl_2)	315-415	2.11	7.70	NA	100.0
	WRRF Post-Cl	Whole Water (WWTP Effluent, Cl_2)	315-415	1.39	5.80	NA	42.0
	Kiwannis	Whole Water (WW-Impacted River)	315-415	1.75	4.40	NA	35.0
	North Yahara	Whole Water (River)	315-415	1.12	3.08	NA	266.0
	Lake Mendota	Whole Water (Lake)	315-415	1.45	2.80	NA	9.7
	Lake Wingra	Whole Water (Lake)	315-415	1.22	3.20	NA	56.0
	Lake Kegonsa	Whole Water (Lake)	315-415	2.10	5.25	NA	140.0
	South Yahara	Whole Water (River)	315-415	1.50	2.80	NA	27.0
	Badfish Upstream	Whole Water (River)	315-415	1.67	3.80	NA	43.0
	Nine Springs PreUV	Whole Water (WWTP Effluent)	315-415	1.07	3.42	NA	53.0
	Nine Springs PostUV	Whole Water (WWTP Effluent, UV)	315-415	1.62	3.90	NA	30.8
	Badfish Downstream	Whole Water (WW-Impacted River)	315-415	1.52	2.76	NA	110.0
	Confluence	Whole Water (WW-Impacted River)	315-415	1.75	2.40	NA	30.0
	Sand Creek	Whole Water (River)	315-415	0.85	6.00	NA	583.0
	Meadowlands	Whole Water (River)	315-415	1.11	1.10	NA	139.0
	River Inn	Whole Water (River)	315-415	1.15	1.37	NA	125.0
	Munger Landing	Whole Water (River)	315-415	1.15	1.70	NA	71.0
	East Detroit	Whole Water (River)	315-415	1.12	1.55	NA	84.0

**Table S37.** Summary of literature $\Phi_{app,RI}$ data (continued)							
Source	Sample ID	Sample Classification	Wavelength Range (nm)	$\Phi_{app,\ ^3DOM^*_{TMP}}$ ($\times10^{-2}$ mol mol-photons^{-1})	$\Phi_{app,\ ^1O_2}$ ($\times10^{-2}$ mol mol-photons^{-1})	$\Phi_{app,\ ^3DOM^*_{Sorbate}}$ ($\times10^{-2}$ mol mol-photons^{-1})	$\Phi_{app,\ ^{\bullet}OH}$ ($\times10^{-6}$ mol mol-photons^{-1})
Ref 120	WLSSD Pre-Cl; Mill Shutdown	Whole Water (WWTP Effluent)	315-415	0.63	1.07	NA	NA
	WLSSD Post-Cl; Mill Shutdown	Whole Water (WWTP Effluent, Cl_2)	315-415	0.83	1.56	NA	NA
	WLSSD Pre-Cl	Whole Water (WWTP Effluent)	315-415	0.78	2.04	NA	149.0
	WLSSD Post-Cl	Whole Water (WWTP Effluent, Cl_2)	315-415	0.71	1.96	NA	610.0
	Blatnik Bridge	Whole Water (River)	315-415	1.00	1.80	NA	68.0
	Wisconsin Point	Whole Water (Lake)	315-415	0.52	2.25	NA	144.0
	River Front Park	Whole Water (River)	315-415	2.26	4.70	NA	189.0
	East River Parkway	Whole Water (River)	315-415	1.85	3.20	NA	11.0
	Minnesota River	Whole Water (River)	315-415	3.23	5.90	NA	240.0
	Metro WWTP Pre-Cl	Whole Water (WWTP Effluent)	315-415	1.00	8.27	NA	1050.0
	Metro WWTP Post-Cl	Whole Water (WWTP Effluent, Cl_2)	315-415	1.27	7.00	NA	1030.0
	Metro Downstream	Whole Water (WW-Impacted River)	315-415	1.40	3.70	NA	107.0
	Eagles Point Pre-UV	Whole Water (WWTP Effluent)	315-415	0.78	2.60	NA	249.0
	Eagles Point Post-UV	Whole Water (WWTP Effluent, UV)	315-415	0.89	2.79	NA	400.0
	Eagles Point Downstream	Whole Water (WW-Impacted River)	315-415	0.99	3.74	NA	126.0
	Lake of the Isles	Whole Water (Lake)	315-415	2.89	5.80	NA	53.0
	Vadnais Lake	Whole Water (Lake)	315-415	2.22	4.65	NA	34.0
	Lake Phalen	Whole Water (Lake)	315-415	2.96	7.00	NA	3.4
Ref 121	IOM 0H	PPL Extract (Algae Lake)	290-400	1.59	3.14	NA	7.9
	IOM 1H	PPL Extract (Algae Lake, Photo)	290-400	1.26	1.93	NA	6.7
	IOM 10H	PPL Extract (Algae Lake, Photo)	290-400	1.26	1.31	NA	4.8
	IOM 50H	PPL Extract (Algae Lake, Photo)	290-400	1.07	0.94	NA	14.2
	IOM 100H	PPL Extract (Algae Lake, Photo)	290-400	1.15	0.46	NA	31.5
	EOM 0H	PPL Extract (Algae Lake)	290-400	4.44	5.48	NA	8.6
	EOM 1H	PPL Extract (Algae Lake, Photo)	290-400	3.59	5.47	NA	7.7
	EOM 10H	PPL Extract (Algae Lake, Photo)	290-400	3.04	5.30	NA	8.5
	EOM 50H	PPL Extract (Algae Lake, Photo)	290-400	2.78	5.51	NA	6.0
	EOM 100H	PPL Extract (Algae Lake, Photo)	290-400	3.00	5.75	NA	4.3
	SRDOM 0H	IHSS Isolate (SRNOM)	290-400	3.52	4.83	NA	17.6
	SRDOM 1H	IHSS Isolate (SRNOM, Photo)	290-400	3.29	4.48	NA	10.9
	SRDOM 10H	IHSS Isolate (SRNOM, Photo)	290-400	2.52	3.81	NA	11.1
	SRDOM 50H	IHSS Isolate (SRNOM, Photo)	290-400	2.38	3.71	NA	28.2
	SRDOM 100H	IHSS Isolate (SRNOM, Photo)	290-400	2.19	3.41	NA	42.2
Ref 122	SRFA pH 4	IHSS Isolate (SRFA)	350-400	NA	0.96	NA	NA
	SRFA pH 5	IHSS Isolate (SRFA)	350-400	NA	0.88	NA	NA
	SRFA pH 6	IHSS Isolate (SRFA)	350-400	NA	0.83	NA	NA
	SRFA pH 7	IHSS Isolate (SRFA)	350-400	NA	0.81	NA	NA
	SRFA pH 8	IHSS Isolate (SRFA)	350-400	NA	0.79	NA	NA
	SRFA pH 9	IHSS Isolate (SRFA)	350-400	NA	0.61	NA	NA
	SRNOM pH 4	IHSS Isolate (SRNOM)	350-400	NA	1.06	NA	NA
	SRNOM pH 5	IHSS Isolate (SRNOM)	350-400	NA	0.99	NA	NA
	SRNOM pH 6	IHSS Isolate (SRNOM)	350-400	NA	0.99	NA	NA

Table S37. Summary of literature $\Phi_{app,RI}$ data (continued)							
Source	Sample ID	Sample Classification	Wavelength Range (nm)	$\Phi_{app,\ ^3DOM^*_{TMP}}$ (×10^{-2} mol mol-photons^{-1})	$\Phi_{app,\ ^1O_2}$ (×10^{-2} mol mol-photons^{-1})	$\Phi_{app,\ ^3DOM^*_{Sorbate}}$ (×10^{-2} mol mol-photons^{-1})	$\Phi_{app,\ ^{\bullet}OH}$ (×10^{-6} mol mol-photons^{-1})
Ref 122	SRNOM pH 7	IHSS Isolate (SRNOM)	350-400	NA	0.95	NA	NA
	SRNOM pH 8	IHSS Isolate (SRNOM)	350-400	NA	0.78	NA	NA
	SRNOM pH 9	IHSS Isolate (SRNOM)	350-400	NA	0.74	NA	NA
	MRNOM pH 4	IHSS Isolate (UMRNOM)	350-400	NA	1.42	NA	NA
	MRNOM pH 5	IHSS Isolate (UMRNOM)	350-400	NA	1.29	NA	NA
	MRNOM pH 6	IHSS Isolate (UMRNOM)	350-400	NA	1.15	NA	NA
	MRNOM pH 7	IHSS Isolate (UMRNOM)	350-400	NA	1.24	NA	NA
	MRNOM pH 8	IHSS Isolate (UMRNOM)	350-400	NA	1.26	NA	NA
	MRNOM pH 9	IHSS Isolate (UMRNOM)	350-400	NA	1.11	NA	NA
	PPFA pH 4	IHSS Isolate (PPFA)	350-400	NA	3.30	NA	NA
	PPFA pH 5	IHSS Isolate (PPFA)	350-400	NA	2.92	NA	NA
	PPFA pH 6	IHSS Isolate (PPFA)	350-400	NA	3.17	NA	NA
	PPFA pH 7	IHSS Isolate (PPFA)	350-400	NA	3.01	NA	NA
	PPFA pH 8	IHSS Isolate (PPFA)	350-400	NA	2.74	NA	NA
	PPFA pH 9	IHSS Isolate (PPFA)	350-400	NA	2.95	NA	NA
	PPHA pH 4	IHSS Isolate (PPHA)	350-400	NA	0.70	NA	NA
	PPHA pH 5	IHSS Isolate (PPHA)	350-400	NA	0.90	NA	NA
	PPHA pH 6	IHSS Isolate (PPHA)	350-400	NA	0.81	NA	NA
	PPHA pH 7	IHSS Isolate (PPHA)	350-400	NA	0.91	NA	NA
	PPHA pH 8	IHSS Isolate (PPHA)	350-400	NA	0.89	NA	NA
	PPHA pH 9	IHSS Isolate (PPHA)	350-400	NA	0.86	NA	NA
	ESHA pH 4	IHSS Isolate (ESHA)	350-400	NA	0.94	NA	NA
	ESHA pH 5	IHSS Isolate (ESHA)	350-400	NA	1.41	NA	NA
	ESHA pH 6	IHSS Isolate (ESHA)	350-400	NA	1.62	NA	NA
	ESHA pH 7	IHSS Isolate (ESHA)	350-400	NA	1.58	NA	NA
	ESHA pH 8	IHSS Isolate (ESHA)	350-400	NA	1.56	NA	NA
	ESHA pH 9	IHSS Isolate (ESHA)	350-400	NA	1.70	NA	NA
Ref 123	PLFA 0 mmol O_3 / mmol C Fraction 1	IHSS Isolate (PLFA, SEC)	290-400	NA	0.88	NA	NA
	PLFA 0 mmol O_3 / mmol C Fraction 2	IHSS Isolate (PLFA, SEC)	290-400	NA	1.61	NA	NA
	PLFA 0 mmol O_3 / mmol C Fraction 3	IHSS Isolate (PLFA, SEC)	290-400	NA	2.01	NA	NA
	PLFA 0 mmol O_3 / mmol C Reconstituted	IHSS Isolate (PLFA)	290-400	NA	1.50	NA	NA
	PLFA 0.05 mmol O_3 / mmol C Fraction 1	IHSS Isolate (PLFA, SEC, O_3)	290-400	NA	2.73	NA	NA
	PLFA 0.05 mmol O_3 / mmol C Fraction 2	IHSS Isolate (PLFA, SEC, O_3)	290-400	NA	2.18	NA	NA
	PLFA 0.05 mmol O_3 / mmol C Fraction 3	IHSS Isolate (PLFA, SEC, O_3)	290-400	NA	5.06	NA	NA
	PLFA 0.05 mmol O_3 / mmol C Reconstituted	IHSS Isolate (PLFA, O_3)	290-400	NA	2.87	NA	NA
	PLFA 0.05 t-BuOH mmol O_3 / mmol C Fraction 1	IHSS Isolate (PLFA, SEC, O_3, tBuOH)	290-400	NA	1.42	NA	NA
	PLFA 0.05 t-BuOH mmol O_3 / mmol C Fraction 2	IHSS Isolate (PLFA, SEC, O_3, tBuOH)	290-400	NA	2.28	NA	NA
	PLFA 0.05 t-BuOH mmol O_3 / mmol C Fraction 3	IHSS Isolate (PLFA, SEC, O_3, tBuOH)	290-400	NA	4.42	NA	NA
	PLFA 0.05 t-BuOH mmol O_3 / mmol C Reconstituted	IHSS Isolate (PLFA, O_3, tBuOH)	290-400	NA	2.50	NA	NA
	PLFA 0.1 mmol O_3 / mmol C Fraction 1	IHSS Isolate (PLFA, SEC, O_3)	290-400	NA	2.36	NA	NA
	PLFA 0.1 mmol O_3 / mmol C Fraction 2	IHSS Isolate (PLFA, SEC, O_3)	290-400	NA	3.24	NA	NA
	PLFA 0.1 mmol O_3 / mmol C Fraction 3	IHSS Isolate (PLFA, SEC, O_3)	290-400	NA	4.95	NA	NA

Table S37. Summary of literature $\Phi_{app,RI}$ data (continued)							
Source	Sample ID	Sample Classification	Wavelength Range (nm)	$\Phi_{app, ^3DOM^*_{TMP}}$ ($\times 10^{-2}$ mol mol-photons^{-1})	$\Phi_{app, ^1O_2}$ ($\times 10^{-2}$ mol mol-photons^{-1})	$\Phi_{app, ^3DOM^*_{Sorbate}}$ ($\times 10^{-2}$ mol mol-photons^{-1})	$\Phi_{app, \cdot OH}$ ($\times 10^{-6}$ mol mol-photons^{-1})
Ref 123	PLFA 0.1 mmol O_3 / mmol C Reconstituted	IHSS Isolate (PLFA, O_3)	290-400	NA	3.35	NA	NA
	PLFA 0.1 t-BuOH mmol O_3 / mmol C Fraction	IHSS Isolate (PLFA, SEC, O_3, tBuOH)	290-400	NA	2.89	NA	NA
	PLFA 0.1 t-BuOH mmol O_3 / mmol C Fraction	IHSS Isolate (PLFA, SEC, O_3, tBuOH)	290-400	NA	4.36	NA	NA
	PLFA 0.1 t-BuOH mmol O_3 / mmol C Fraction	IHSS Isolate (PLFA, SEC, O_3, tBuOH)	290-400	NA	5.70	NA	NA
	PLFA 0.1 t-BuOH mmol O_3 / mmol C Reconstituted	IHSS Isolate (PLFA, O_3, tBuOH)	290-400	NA	4.31	NA	NA
	PLFA 0.2 mmol O_3 / mmol C Fraction 1	IHSS Isolate (PLFA, SEC, O_3)	290-400	NA	2.60	NA	NA
	PLFA 0.2 mmol O_3 / mmol C Fraction 2	IHSS Isolate (PLFA, SEC, O_3)	290-400	NA	2.96	NA	NA
	PLFA 0.2 mmol O_3 / mmol C Fraction 3	IHSS Isolate (PLFA, SEC, O_3)	290-400	NA	7.37	NA	NA
	PLFA 0.2 mmol O_3 / mmol C Reconstituted	IHSS Isolate (PLFA, O_3)	290-400	NA	3.84	NA	NA
	PLFA 0.2 t-BuOH mmol O_3 / mmol C Fraction	IHSS Isolate (PLFA, SEC, O_3, tBuOH)	290-400	NA	3.22	NA	NA
	PLFA 0.2 t-BuOH mmol O_3 / mmol C Fraction	IHSS Isolate (PLFA, SEC, O_3, tBuOH)	290-400	NA	4.07	NA	NA
	PLFA 0.2 t-BuOH mmol O_3 / mmol C Fraction	IHSS Isolate (PLFA, SEC, O_3, tBuOH)	290-400	NA	6.72	NA	NA
	PLFA 0.2 t-BuOH mmol O_3 / mmol C Reconstituted	IHSS Isolate (PLFA, O_3, tBuOH)	290-400	NA	4.46	NA	NA
	SRFA 0 mmol O_3 / mmol C Fraction 1	IHSS Isolate (SRFA, SEC)	290-400	NA	0.69	NA	NA
	SRFA 0 mmol O_3 / mmol C Fraction 2	IHSS Isolate (SRFA, SEC)	290-400	NA	2.30	NA	NA
	SRFA 0 mmol O_3 / mmol C Fraction 3	IHSS Isolate (SRFA, SEC)	290-400	NA	3.23	NA	NA
	SRFA 0 mmol O_3 / mmol C Reconstituted	IHSS Isolate (SRFA)	290-400	NA	1.36	NA	NA
	SRFA 0.05 mmol O_3 / mmol C Fraction 1	IHSS Isolate (SRFA, SEC, O_3)	290-400	NA	0.68	NA	NA
	SRFA 0.05 mmol O_3 / mmol C Fraction 2	IHSS Isolate (SRFA, SEC, O_3)	290-400	NA	1.45	NA	NA
	SRFA 0.05 mmol O_3 / mmol C Fraction 3	IHSS Isolate (SRFA, SEC, O_3)	290-400	NA	4.04	NA	NA
	SRFA 0.05 mmol O_3 / mmol C Reconstituted	IHSS Isolate (SRFA, O_3)	290-400	NA	1.29	NA	NA
	SRFA 0.05 t-BuOH mmol O_3 / mmol C Fraction 1	IHSS Isolate (SRFA, SEC, O_3, tBuOH)	290-400	NA	1.12	NA	NA
	SRFA 0.05 t-BuOH mmol O_3 / mmol C Fraction 2	IHSS Isolate (SRFA, SEC, O_3, tBuOH)	290-400	NA	2.52	NA	NA
	SRFA 0.05 t-BuOH mmol O_3 / mmol C Fraction 3	IHSS Isolate (SRFA, SEC, O_3, tBuOH)	290-400	NA	4.51	NA	NA
	SRFA 0.05 t-BuOH mmol O_3 / mmol C Reconstituted	IHSS Isolate (SRFA, O_3, tBuOH)	290-400	NA	2.01	NA	NA
	SRFA 0.1 mmol O_3 / mmol C Fraction 1	IHSS Isolate (SRFA, SEC, O_3)	290-400	NA	0.81	NA	NA
	SRFA 0.1 mmol O_3 / mmol C Fraction 2	IHSS Isolate (SRFA, SEC, O_3)	290-400	NA	2.08	NA	NA
	SRFA 0.1 mmol O_3 / mmol C Fraction 3	IHSS Isolate (SRFA, SEC, O_3)	290-400	NA	6.04	NA	NA
	SRFA 0.1 mmol O_3 / mmol C Reconstituted	IHSS Isolate (SRFA, O_3)	290-400	NA	1.74	NA	NA
	SRFA 0.2 mmol O_3 / mmol C Fraction 1	IHSS Isolate (SRFA, SEC, O_3)	290-400	NA	2.04	NA	NA
	SRFA 0.2 mmol O_3 / mmol C Fraction 2	IHSS Isolate (SRFA, SEC, O_3)	290-400	NA	2.06	NA	NA
	SRFA 0.2 mmol O_3 / mmol C Fraction 3	IHSS Isolate (SRFA, SEC, O_3)	290-400	NA	10.86	NA	NA
	SRFA 0.2 mmol O_3 / mmol C Reconstituted	IHSS Isolate (SRFA, O_3)	290-400	NA	3.35	NA	NA
	SRFA 0.2 t-BuOH mmol O_3 / mmol C Fraction	IHSS Isolate (SRFA, SEC, O_3, tBuOH)	290-400	NA	2.89	NA	NA
	SRFA 0.2 t-BuOH mmol O_3 / mmol C Fraction	IHSS Isolate (SRFA, SEC, O_3, tBuOH)	290-400	NA	3.06	NA	NA
	SRFA 0.2 t-BuOH mmol O_3 / mmol C Fraction	IHSS Isolate (SRFA, SEC, O_3, tBuOH)	290-400	NA	10.65	NA	NA
	SRFA 0.2 t-BuOH mmol O_3 / mmol C Reconstituted	IHSS Isolate (SRFA, O_3, tBuOH)	290-400	NA	3.95	NA	NA
Ref 124	Pine Needle BC 500 C 365 nm	DBC (pyDOM)	365	3.86	2.17	NA	80.9
	Shell BC 365 nm	DBC (pyDOM)	365	2.58	2.54	NA	88.4
	Straw BC 365 nm	DBC (pyDOM)	365	3.02	3.73	NA	139.1
	Wood BC 365 nm	DBC (pyDOM)	365	1.50	1.65	NA	157.8
	Wood BC 375 nm	DBC (pyDOM)	375	3.91	3.63	NA	303.9

Table S37. Summary of literature $\Phi_{app,RI}$ data (continued)							
Source	Sample ID	Sample Classification	Wavelength Range (nm)	$\Phi_{app,\ ^3DOM^*_{TMP}}$ (×10^{-2} mol mol-photons^{-1})	$\Phi_{app,\ ^1O_2}$ (×10^{-2} mol mol-photons^{-1})	$\Phi_{app,\ ^3DOM^*_{Sorbate}}$ (×10^{-2} mol mol-photons^{-1})	$\Phi_{app,\ ^\bullet OH}$ (×10^{-6} mol mol-photons^{-1})
Ref 124	Wood BC 387 nm	DBC (pyDOM)	387	2.12	2.14	NA	99.4
	Wood BC 425 nm	DBC (pyDOM)	425	1.64	1.31	NA	89.3
	Wood BC 461 nm	DBC (pyDOM)	461	1.43	0.76	NA	70.0
	Wood BC 490 nm	DBC (pyDOM)	490	0.82	0.33	NA	71.3
	Wood BC 531 nm	DBC (pyDOM)	531	NA	NA	NA	43.2
	Wood BC 300 C	DBC (pyDOM)	365	1.21	0.95	NA	158.5
	Wood BC 500 C	DBC (pyDOM)	365	1.51	1.48	NA	163.4
	Wood BC 700 C	DBC (pyDOM)	365	0.38	0.40	NA	290.2
	Wood BC 900 C	DBC (pyDOM)	365	0.18	0.05	NA	222.0
	Wood BC 0.22 µm	DBC (pyDOM)	365	6.69	7.38	NA	704.1
	Wood BC 10 µm	DBC (pyDOM)	365	1.98	1.06	NA	379.0
	Wood BC 80 µm	DBC (pyDOM)	365	1.38	1.05	NA	219.3
Ref 125	BDBC	DBC (pyDOM)	290-400	9.50	NA	NA	NA
	RDBC	DBC (pyDOM)	290-400	3.90	NA	NA	NA
	PDBC	DBC (pyDOM)	290-400	3.70	NA	NA	NA
	CDBC	DBC (pyDOM)	290-400	6.70	NA	NA	NA
	SDBC	DBC (pyDOM)	290-400	6.30	NA	NA	NA
	SRHA	IHSS Isolate (SRHA)	290-400	1.20	NA	NA	NA
	SRFA	IHSS Isolate (SRFA)	290-400	1.40	NA	NA	NA
	SRNOM	IHSS Isolate (SRNOM)	290-400	1.70	NA	NA	NA
	AHA	XAD Fraction (Soil)	290-400	1.40	NA	NA	NA
Ref 126	DBC200	DBC (pyDOM)	290-400	0.49	0.21	0.12	36.3
	DBC300	DBC (pyDOM)	290-400	0.67	0.21	0.15	22.0
	DBC400	DBC (pyDOM)	290-400	0.57	0.61	0.37	15.9
	DBC500	DBC (pyDOM)	290-400	1.15	1.77	0.76	65.2
Ref 127	Sep._1	Whole Water (Seawater)	290-700	1.35	5.09	8.90	2688.5
	Sep._2	Whole Water (Seawater)	290-700	0.51	2.27	0.99	213.1
	Sep._3	Whole Water (Seawater)	290-700	0.56	1.65	1.24	166.2
	Sep._4	Whole Water (Seawater)	290-700	0.24	0.92	0.84	NA
	Sep._5	Whole Water (Seawater)	290-700	0.28	0.78	0.76	NA
	Sep._6	Whole Water (Seawater)	290-700	0.29	0.88	0.52	NA
	Sep._7	Whole Water (Seawater)	290-700	0.23	1.03	0.66	NA
	Sep._8	Whole Water (Seawater)	290-700	0.66	2.07	1.85	395.7
	Nov._1	Whole Water (Seawater)	290-700	2.34	6.14	5.68	1517.6
	Nov._2	Whole Water (Seawater)	290-700	1.17	4.24	4.37	414.7
	Nov._3	Whole Water (Seawater)	290-700	1.24	4.13	5.69	371.7
	Nov._4	Whole Water (Seawater)	290-700	0.45	1.83	2.88	NA
	Nov._5	Whole Water (Seawater)	290-700	0.97	3.38	3.20	NA
	Nov._6	Whole Water (Seawater)	290-700	0.82	3.19	1.76	NA
	Nov._7	Whole Water (Seawater)	290-700	0.93	3.69	3.94	NA
	Nov._8	Whole Water (Seawater)	290-700	0.59	2.69	2.41	NA
	SRNOM	IHSS Isolate (SRNOM)	290-700	0.15	1.66	0.79	19.8

Table S37. Summary of literature $\Phi_{app,RI}$ data (continued)							
Source	Sample ID	Sample Classification	Wavelength Range (nm)	$\Phi_{app,\ ^3DOM^*_{TMP}}$ (×10^{-2} mol mol-photons^{-1})	$\Phi_{app,\ ^1O_2}$ (×10^{-2} mol mol-photons^{-1})	$\Phi_{app,\ ^3DOM^*_{Sorbate}}$ (×10^{-2} mol mol-photons^{-1})	$\Phi_{app,\ ^{\cdot}OH}$ (×10^{-6} mol mol-photons^{-1})
Ref 128	SRNOM_1-10 kDa fraction 305 nm	UF Fraction (SRNOM)	280-400	NA	NA	NA	70.3
	SRNOMCl0.2_1-10 kDa fraction 305 nm	UF Fraction (SRNOM, Cl_2)	280-400	NA	NA	NA	99.3
	SRNOMCl0.5_1-10 kDa fraction 305 nm	UF Fraction (SRNOM, Cl_2)	280-400	NA	NA	NA	160.0
	SRNOMCl1.0_1-10 kDa fraction 305 nm	UF Fraction (SRNOM, Cl_2)	280-400	NA	NA	NA	204.5
	SRNOMCl2.0_1-10 kDa fraction 305 nm	UF Fraction (SRNOM, Cl_2)	280-400	NA	NA	NA	225.8
	YRNOM_1-10 kDa fraction 305 nm	C18+PPL Extract+UF Fraction (WW-Impacted River)	280-400	NA	NA	NA	68.1
	YRNOMCl0.2_1-10 kDa fraction 305 nm	C18+PPL Extract+UF Fraction (WW-Impacted River, Cl_2)	280-400	NA	NA	NA	69.4
	YRNOMCl0.5_1-10 kDa fraction 305 nm	C18+PPL Extract+UF Fraction (WW-Impacted River, Cl_2)	280-400	NA	NA	NA	105.0
	YRNOMCl1.0_1-10 kDa fraction 305 nm	C18+PPL Extract+UF Fraction (WW-Impacted River, Cl_2)	280-400	NA	NA	NA	132.4
	YRNOMCl2.0_1-10 kDa fraction 305 nm	C18+PPL Extract+UF Fraction (WW-Impacted River, Cl_2)	280-400	NA	NA	NA	149.4
	TLNOM_1-10 kDa fraction 305 nm	C18+PPL Extract+UF Fraction (WW-Impacted Lake)	280-400	NA	NA	NA	89.4
	TLNOMCl0.2_1-10 kDa fraction 305 nm	C18+PPL Extract+UF Fraction (WW-Impacted Lake, Cl_2)	280-400	NA	NA	NA	107.1
	TLNOMCl0.5_1-10 kDa fraction 305 nm	C18+PPL Extract+UF Fraction (WW-Impacted Lake, Cl_2)	280-400	NA	NA	NA	126.0
	TLNOMCl1.0_1-10 kDa fraction 305 nm	C18+PPL Extract+UF Fraction (WW-Impacted Lake, Cl_2)	280-400	NA	NA	NA	132.6
	TLNOMCl2.0_1-10 kDa fraction 305 nm	C18+PPL Extract+UF Fraction (WW-Impacted Lake, Cl_2)	280-400	NA	NA	NA	134.2
	SRNOM_1-10 kDa fraction 365 nm	UF Fraction (SRNOM)	280-400	NA	NA	NA	7.4
	SRNOMCl0.2_1-10 kDa fraction 365 nm	UF Fraction (SRNOM, Cl_2)	280-400	NA	NA	NA	8.6
	SRNOMCl0.5_1-10 kDa fraction 365 nm	UF Fraction (SRNOM, Cl_2)	280-400	NA	NA	NA	16.7
	SRNOMCl1.0_1-10 kDa fraction 365 nm	UF Fraction (SRNOM, Cl_2)	280-400	NA	NA	NA	22.5
	SRNOMCl2.0_1-10 kDa fraction 365 nm	UF Fraction (SRNOM, Cl_2)	280-400	NA	NA	NA	27.8
	YRNOM_1-10 kDa fraction 365 nm	C18+PPL Extract+UF Fraction (WW-Impacted River)	280-400	NA	NA	NA	5.4
	YRNOMCl0.2_1-10 kDa fraction 365 nm	C18+PPL Extract+UF Fraction (WW-Impacted River, Cl_2)	280-400	NA	NA	NA	6.1
	YRNOMCl0.5_1-10 kDa fraction 365 nm	C18+PPL Extract+UF Fraction (WW-Impacted River, Cl_2)	280-400	NA	NA	NA	10.6
	YRNOMCl1.0_1-10 kDa fraction 365 nm	C18+PPL Extract+UF Fraction (WW-Impacted River, Cl_2)	280-400	NA	NA	NA	14.2
	YRNOMCl2.0_1-10 kDa fraction 365 nm	C18+PPL Extract+UF Fraction (WW-Impacted River, Cl_2)	280-400	NA	NA	NA	16.7
	TLNOM_1-10 kDa fraction 365 nm	C18+PPL Extract+UF Fraction (WW-Impacted Lake)	280-400	NA	NA	NA	8.5
	TLNOMCl0.2_1-10 kDa fraction 365 nm	C18+PPL Extract+UF Fraction (WW-Impacted Lake, Cl_2)	280-400	NA	NA	NA	9.5
	TLNOMCl0.5_1-10 kDa fraction 365 nm	C18+PPL Extract+UF Fraction (WW-Impacted Lake, Cl_2)	280-400	NA	NA	NA	10.3
	TLNOMCl1.0_1-10 kDa fraction 365 nm	C18+PPL Extract+UF Fraction (WW-Impacted Lake, Cl_2)	280-400	NA	NA	NA	13.1
	TLNOMCl2.0_1-10 kDa fraction 365 nm	C18+PPL Extract+UF Fraction (WW-Impacted Lake, Cl_2)	280-400	NA	NA	NA	13.3
	2-HBA	Model Compound	280-400	NA	NA	NA	120.0
	3-Cl-2-HBA	Model Compound	280-400	NA	NA	NA	360.0
	5-Cl-2-HBA	Model Compound	280-400	NA	NA	NA	310.0
	3,5-diCl-2-HBA	Model Compound	280-400	NA	NA	NA	1120.0
	2-Cl-5-HBA	Model Compound	280-400	NA	NA	NA	1280.0
	3-Cl-5-HBA	Model Compound	280-400	NA	NA	NA	280.0
	4-Cl-3-HBA	Model Compound	280-400	NA	NA	NA	810.0
	2-Cl-4-HBA	Model Compound	280-400	NA	NA	NA	2960.0
	3-Cl-4-HBA	Model Compound	280-400	NA	NA	NA	1020.0
	3,5-diCl-4-HBA	Model Compound	280-400	NA	NA	NA	470.0
	2,3-DHBA	Model Compound	280-400	NA	NA	NA	80.0
	2,4-DHBA	Model Compound	280-400	NA	NA	NA	5920.0

Table S37. Summary of literature $\Phi_{app,RI}$ data (continued)							
Source	Sample ID	Sample Classification	Wavelength Range (nm)	$\Phi_{app,\ ^3DOM^*_{TMP}}$ ($\times10^{-2}$ mol mol-photons^{-1})	$\Phi_{app,\ ^1O_2}$ ($\times10^{-2}$ mol mol-photons^{-1})	$\Phi_{app,\ ^3DOM^*_{Sorbate}}$ ($\times10^{-2}$ mol mol-photons^{-1})	$\Phi_{app,\ ^{\bullet}OH}$ ($\times10^{-6}$ mol mol-photons^{-1})
Ref 128	2,5-DHBA	Model Compound	280-400	NA	NA	NA	160.0
	3,4-DHBA	Model Compound	280-400	NA	NA	NA	480.0
	3,5-DHBA	Model Compound	280-400	NA	NA	NA	690.0
	2-Cl-3,4-DHBA	Model Compound	280-400	NA	NA	NA	1650.0
	3-Cl-4,5-DHBA	Model Compound	280-400	NA	NA	NA	180.0
	5-Cl-2,4-DHBA	Model Compound	280-400	NA	NA	NA	2350.0
	2-HBA	Model Compound	280-400	NA	NA	NA	230.0
	3-Cl-2-HBA	Model Compound	280-400	NA	NA	NA	1350.0
	5-Cl-2-HBA	Model Compound	280-400	NA	NA	NA	650.0
	3,5-diCl-2-HBA	Model Compound	280-400	NA	NA	NA	7050.0
	3-HBA	Model Compound	280-400	NA	NA	NA	290.0
	2-Cl-5-HBA	Model Compound	280-400	NA	NA	NA	380.0
	3-Cl-5-HBA	Model Compound	280-400	NA	NA	NA	530.0
	4-Cl-3-HBA	Model Compound	280-400	NA	NA	NA	700.0
	4-HBA	Model Compound	280-400	NA	NA	NA	350.0
	2-Cl-4-HBA	Model Compound	280-400	NA	NA	NA	940.0
	3-Cl-4-HBA	Model Compound	280-400	NA	NA	NA	720.0
	3,5-diCl-4-HBA	Model Compound	280-400	NA	NA	NA	870.0
	2,3-DHBA	Model Compound	280-400	NA	NA	NA	240.0
	2,4-DHBA	Model Compound	280-400	NA	NA	NA	9460.0
	2,5-DHBA	Model Compound	280-400	NA	NA	NA	520.0
	3,4-DHBA	Model Compound	280-400	NA	NA	NA	590.0
	3,5-DHBA	Model Compound	280-400	NA	NA	NA	2570.0
	2-Cl-3,4-DHBA	Model Compound	280-400	NA	NA	NA	2170.0
	3-Cl-4,5-DHBA	Model Compound	280-400	NA	NA	NA	740.0
	5-Cl-2,4-DHBA	Model Compound	280-400	NA	NA	NA	8410.0
Ref 129	SRNOM 300 nm Phosphorescence	IHSS Isolate (SRNOM)	300	NA	2.70	NA	NA
	Grizzly Creek 300 nm	DBC (pyDOM)	330	NA	5.70	NA	NA
	Oak250 pyDOM 300 nm	DBC (pyDOM)	365	NA	6.30	NA	NA
	Pine150 pyDOM 300 nm	DBC (pyDOM)	390	NA	4.50	NA	NA
	Pine250 pyDOM 300 nm	DBC (pyDOM)	420	NA	8.20	NA	NA
	Pine350 pyDOM 300 nm	DBC (pyDOM)	430	NA	1.70	NA	NA
	Grizzly Creek 330 nm	DBC (pyDOM)	500	NA	3.50	NA	NA
	Oak250 pyDOM 330 nm	DBC (pyDOM)	300	NA	4.30	NA	NA
	Pine250 pyDOM 330 nm	DBC (pyDOM)	365	NA	3.70	NA	NA
	SRNOM 365 nm	IHSS Isolate (SRNOM)	420	NA	2.40	NA	NA
	Grizzly Creek 365 nm	DBC (pyDOM)	430	NA	2.10	NA	NA
	Oak250 pyDOM 365 nm	DBC (pyDOM)	450	NA	2.40	NA	NA
	Pine150 pyDOM 365 nm	DBC (pyDOM)	500	NA	2.10	NA	NA
	Pine250 pyDOM 365 nm	DBC (pyDOM)	300	NA	2.70	NA	NA
	Pine350 pyDOM 365 nm	DBC (pyDOM)	330	NA	0.60	NA	NA
	Grizzly Creek 390 nm	DBC (pyDOM)	390	NA	2.00	NA	NA

Table S37. Summary of literature $\Phi_{app,RI}$ data (continued)							
Source	Sample ID	Sample Classification	Wavelength Range (nm)	$\Phi_{app, ^3DOM^*_{TMP}}$ (×10^{-2} mol mol-photons^{-1})	$\Phi_{app, ^1O_2}$ (×10^{-2} mol mol-photons^{-1})	$\Phi_{app, ^3DOM^*_{Sorbate}}$ (×10^{-2} mol mol-photons^{-1})	$\Phi_{app, ^{\cdot}OH}$ (×10^{-6} mol mol-photons^{-1})
Ref 129	Oak250 pyDOM 390 nm	DBC (pyDOM)	420	NA	1.50	NA	NA
	Pine250 pyDOM 390 nm	DBC (pyDOM)	450	NA	2.10	NA	NA
	Grizzly Creek 420 nm	DBC (pyDOM)	330	NA	1.80	NA	NA
	Pine250 pyDOM 420 nm	DBC (pyDOM)	420	NA	0.80	NA	NA
	SRNOM 430 nm	IHSS Isolate (SRNOM)	450	NA	1.30	NA	NA
	Grizzly Creek 450 nm	DBC (pyDOM)	430	NA	0.90	NA	NA
	Oak250 pyDOM 450 nm	DBC (pyDOM)	450	NA	0.70	NA	NA
	Pine150 pyDOM 450 nm	DBC (pyDOM)	500	NA	0.20	NA	NA
	Pine250 pyDOM 450 nm	DBC (pyDOM)	300	NA	0.50	NA	NA
	Pine350 pyDOM 450 nm	DBC (pyDOM)	330	NA	0.20	NA	NA
	SRNOM 500 nm	IHSS Isolate (SRNOM)	365	NA	0.60	NA	NA
	Grizzly Creek 500 nm	DBC (pyDOM)	390	NA	0.50	NA	NA
	Pine250 pyDOM 500 nm	DBC (pyDOM)	450	NA	0.50	NA	NA
	SRNOM Polychromatic	IHSS Isolate (SRNOM)	300-430	NA	1.81	NA	NA
	Grizzly Creek Polychromatic	DBC (pyDOM)	300-450	NA	2.10	NA	NA
	Oak250 pyDOM Polychromatic	DBC (pyDOM)	300-450	NA	2.31	NA	NA
	Pine150 pyDOM Polychromatic	DBC (pyDOM)	300-450	NA	1.80	NA	NA
	Pine250 pyDOM Polychromatic	DBC (pyDOM)	300-450	NA	2.31	NA	NA
	Pine350 pyDOM Polychromatic	DBC (pyDOM)	300-450	NA	0.51	NA	NA
	1,2-Benzenedicarboxylic Acid 300 nm	Model Compound	300	NA	21.80	NA	NA
	1,2,4-Benzenetricarboxylic Acid 300 nm	Model Compound	300	NA	8.20	NA	NA
	1,2,3-Benzetricarboxylic Acid 300 nm	Model Compound	300	NA	13.90	NA	NA
	SRNOM 365 nm FFA	IHSS Isolate (SRNOM)	300-430	NA	1.10	NA	NA
	Grizzly Creek 365 nm FFA	DBC (pyDOM)	300-450	NA	1.40	NA	NA
Ref 130	DOM_{Re}	Whole Water (Reservoir)	300-600	0.07	0.29	NA	4.2
	DOM_{Ri}	Whole Water (River)	300-600	0.03	0.14	NA	3.3
Ref 131	F1_Iwanai	Whole Water (Reservoir)	280-400	2.47	NA	NA	NA
	F2_Kanayama	Whole Water (Reservoir)	280-400	2.53	NA	NA	NA
	F3_Katsurazawa	Whole Water (Reservoir)	280-400	1.61	NA	NA	NA
	F4_Tokachi	Whole Water (Reservoir)	280-400	1.53	NA	NA	NA
	F5_Tokisato	Whole Water (Reservoir)	280-400	2.86	NA	NA	NA
	F6_Chūbetsu	Whole Water (Reservoir)	280-400	1.91	NA	NA	NA
	F7_Shijūshida	Whole Water (Reservoir)	280-400	2.31	NA	NA	NA
	F8_Sagae	Whole Water (Reservoir)	280-400	1.52	NA	NA	NA
	F9_Naruko	Whole Water (Reservoir)	280-400	1.77	NA	NA	NA
	F10_Kamafusa	Whole Water (Reservoir)	280-400	1.65	NA	NA	NA
	F11_Shichikashuku	Whole Water (Reservoir)	280-400	1.47	NA	NA	NA
	F12_Gassan	Whole Water (Reservoir)	280-400	1.92	NA	NA	NA
	F13_Surikamigawa	Whole Water (Reservoir)	280-400	1.32	NA	NA	NA
	F14_Futase	Whole Water (Reservoir)	280-400	2.54	NA	NA	NA
	F15_Ikari	Whole Water (Reservoir)	280-400	2.00	NA	NA	NA
	F16_Kawamata	Whole Water (Reservoir)	280-400	2.83	NA	NA	NA

Table S37. Summary of literature $\Phi_{app,RI}$ data (continued)							
Source	Sample ID	Sample Classification	Wavelength Range (nm)	$\Phi_{app, ^3DOM^*_{TMP}}$ ($\times10^{-2}$ mol mol-photons^{-1})	$\Phi_{app, ^1O_2}$ ($\times10^{-2}$ mol mol-photons^{-1})	$\Phi_{app, ^3DOM^*_{Sorbate}}$ ($\times10^{-2}$ mol mol-photons^{-1})	$\Phi_{app, ^{\cdot}OH}$ ($\times10^{-6}$ mol mol-photons^{-1})
Ref 131	F21_Yagisawa	Whole Water (Reservoir)	280-400	1.87	NA	NA	NA
	F22_Shimokubo	Whole Water (Reservoir)	280-400	6.22	NA	NA	NA
	F23_Ōishi	Whole Water (Reservoir)	280-400	1.60	NA	NA	NA
	F24_Ōkawa	Whole Water (Reservoir)	280-400	2.79	NA	NA	NA
	F25_Ōmachi	Whole Water (Reservoir)	280-400	5.89	NA	NA	NA
	F26_Yawagi	Whole Water (Reservoir)	280-400	2.40	NA	NA	NA
	F27_Yokoyama	Whole Water (Reservoir)	280-400	2.34	NA	NA	NA
	F28_Nagashima	Whole Water (Reservoir)	280-400	4.52	NA	NA	NA
	F29_Misogawa	Whole Water (Reservoir)	280-400	3.33	NA	NA	NA
	F30_Tokuyama	Whole Water (Reservoir)	280-400	3.15	NA	NA	NA
	F31_Takayama	Whole Water (Reservoir)	280-400	2.14	NA	NA	NA
	F32_Shorenji	Whole Water (Reservoir)	280-400	1.55	NA	NA	NA
	F33_Hinachi	Whole Water (Reservoir)	280-400	1.88	NA	NA	NA
	F34_Hitokura	Whole Water (Reservoir)	280-400	2.86	NA	NA	NA
	F35_Tomata	Whole Water (Reservoir)	280-400	1.33	NA	NA	NA
	F36_Sugesawa	Whole Water (Reservoir)	280-400	1.96	NA	NA	NA
	F37_Haji	Whole Water (Reservoir)	280-400	2.20	NA	NA	NA
	F38_Hattabara	Whole Water (Reservoir)	280-400	3.17	NA	NA	NA
	F39_Ikeda	Whole Water (Reservoir)	280-400	1.98	NA	NA	NA
	F40_Sameura	Whole Water (Reservoir)	280-400	1.16	NA	NA	NA
	F41_Shingu	Whole Water (Reservoir)	280-400	1.66	NA	NA	NA
	F42_Tomisato	Whole Water (Reservoir)	280-400	1.41	NA	NA	NA
	F43_Ōdo	Whole Water (Reservoir)	280-400	5.56	NA	NA	NA
	F44_Nagayasuguchi	Whole Water (Reservoir)	280-400	1.66	NA	NA	NA
	F45_Tsuruda	Whole Water (Reservoir)	280-400	1.58	NA	NA	NA
	F46_Shimouke	Whole Water (Reservoir)	280-400	1.77	NA	NA	NA
	F47_Yabakei	Whole Water (Reservoir)	280-400	1.41	NA	NA	NA
	F48_Ryūmon	Whole Water (Reservoir)	280-400	1.20	NA	NA	NA
	F49_Haneji	Whole Water (Reservoir)	280-400	2.33	NA	NA	NA
	F50_Arakawa	Whole Water (Reservoir)	280-400	1.17	NA	NA	NA
	S1_Tokyo bay (Daiba)	Whole Water (Seawater)	280-400	2.64	NA	NA	NA
	S2_Tokyo bay (Chiba port)	Whole Water (Seawater)	280-400	3.48	NA	NA	NA
	S3_Suruga bay (No. 1)	Whole Water (Seawater)	280-400	3.03	NA	NA	NA
	S4_Ise bay (Nagoya port)	Whole Water (Seawater)	280-400	3.24	NA	NA	NA
	S5_Sagami bay (Enoshima)	Whole Water (Seawater)	280-400	3.34	NA	NA	NA
	S6_Wakasa bay (Tsuruga)	Whole Water (Seawater)	280-400	6.52	NA	NA	NA
	S7_Tainai	Whole Water (Seawater)	280-400	3.16	NA	NA	NA
	S8_Hachinohe	Whole Water (Seawater)	280-400	4.33	NA	NA	NA
	S9_Hirono	Whole Water (Seawater)	280-400	2.34	NA	NA	NA
	S10_Kamaishi	Whole Water (Seawater)	280-400	3.70	NA	NA	NA
	S11_Kagoshima bay (Sakurajima)	Whole Water (Seawater)	280-400	3.68	NA	NA	NA
	S12_Mutsu bay (Aomori)	Whole Water (Seawater)	280-400	1.00	NA	NA	NA

**Table S37.** Summary of literature $\Phi_{app,RI}$ data (continued)							
Source	Sample ID	Sample Classification	Wavelength Range (nm)	$\Phi_{app,\ ^3DOM^*_{TMP}}$ ($\times10^{-2}$ mol mol-photons^{-1})	$\Phi_{app,\ ^1O_2}$ ($\times10^{-2}$ mol mol-photons^{-1})	$\Phi_{app,\ ^3DOM^*_{Sorbate}}$ ($\times10^{-2}$ mol mol-photons^{-1})	$\Phi_{app,\ ^{\bullet}OH}$ ($\times10^{-6}$ mol mol-photons^{-1})
Ref 131	S13_Tomakomai	Whole Water (Seawater)	280-400	2.31	NA	NA	NA
	S14_Matsushima	Whole Water (Seawater)	280-400	3.45	NA	NA	NA
	S15_Tsuruokashi	Whole Water (Seawater)	280-400	1.41	NA	NA	NA
	S16_Suruga bay (No. 2)	Whole Water (Seawater)	280-400	1.87	NA	NA	NA
	S17_Himeji	Whole Water (Seawater)	280-400	2.81	NA	NA	NA
	S18_Okayama	Whole Water (Seawater)	280-400	3.54	NA	NA	NA
	S19_Fukuyama	Whole Water (Seawater)	280-400	2.81	NA	NA	NA
	S20_Naruto	Whole Water (Seawater)	280-400	2.89	NA	NA	NA
	S21_Imabari	Whole Water (Seawater)	280-400	2.71	NA	NA	NA
	S22_Iyo	Whole Water (Seawater)	280-400	2.24	NA	NA	NA
	S23_Kochi	Whole Water (Seawater)	280-400	2.26	NA	NA	NA
	S24_Mitoyoshi	Whole Water (Seawater)	280-400	3.17	NA	NA	NA
	S25_Kobe	Whole Water (Seawater)	280-400	2.57	NA	NA	NA
	S26_Ise bay (Yokkaichi)	Whole Water (Seawater)	280-400	3.14	NA	NA	NA
	S27_Naha	Whole Water (Seawater)	280-400	2.20	NA	NA	NA
	S28_Sakai	Whole Water (Seawater)	280-400	2.23	NA	NA	NA
	S29_Takaoka	Whole Water (Seawater)	280-400	3.18	NA	NA	NA
	S30_Uozu	Whole Water (Seawater)	280-400	3.33	NA	NA	NA
	L1_Shinobazu pond	Whole Water (Lake)	280-400	6.78	NA	NA	NA
	L2_Senzokuike pond	Whole Water (Lake)	280-400	4.07	NA	NA	NA
	L3_Suwako lake	Whole Water (Lake)	280-400	3.60	NA	NA	NA
	L4_Inbanuma pond	Whole Water (Lake)	280-400	4.26	NA	NA	NA
	L5_Teganuma pond	Whole Water (Lake)	280-400	2.87	NA	NA	NA
	L6_Ukushinuma pond	Whole Water (Lake)	280-400	4.85	NA	NA	NA
	L7_Kasumigaura lake	Whole Water (Lake)	280-400	3.44	NA	NA	NA
	L8_Biwako lake	Whole Water (Lake)	280-400	6.63	NA	NA	NA
	SRFA	IHSS Isolate (SRNOM)	280-400	2.59	NA	NA	NA
	SRNOM	IHSS Isolate (SRFA)	280-400	2.82	NA	NA	NA
	UMRNOM	IHSS Isolate (UMRNOM)	280-400	2.30	NA	NA	NA
Ref 132	Henan Changsheng Industrial Fulvic acid (FA) 365 nm	XAD Fraction (Soil)	365	*50.40 (excl.)*	*375.50 (excl.)*	NA	120.0
	FA 310 nm	XAD Fraction (Soil)	310	0.30	1.60	NA	2.0
	FA 280 nm	XAD Fraction (Soil)	280	5.10	10.60	NA	140.0
	FA 260 nm	XAD Fraction (Soil)	260	2.40	7.30	NA	70.0
	Aldrich humic acid (HA) 365 nm	XAD Fraction (Soil)	365	*65.20 (excl.)*	*134.50 (excl.)*	NA	770.0
	HA 310 nm	XAD Fraction (Soil)	310	0.30	0.50	NA	30.0
	HA 280 nm	XAD Fraction (Soil)	280	11.00	3.90	NA	870.0
	HA 260 nm	XAD Fraction (Soil)	260	2.90	2.30	NA	280.0
	EfOM 365 nm	PPL Extract (WWTP Effluent)	365	*180.00 (excl.)*	*785.20 (excl.)*	NA	*150140.0 (excl.)*
	EfOM 310 nm	PPL Extract (WWTP Effluent)	310	0.90	0.90	NA	250.0
	EfOM 280 nm	PPL Extract (WWTP Effluent)	280	27.80	13.80	NA	2410.0
	EfOM 260 nm	PPL Extract (WWTP Effluent)	260	8.30	10.20	NA	430.0

**Table S37.** Summary of literature $\Phi_{app,RI}$ data (continued)							
Source	Sample ID	Sample Classification	Wavelength Range (nm)	$\Phi_{app,\ ^3DOM^*_{TMP}}$ ($\times10^{-2}$ mol mol-photons^{-1})	$\Phi_{app,\ ^1O_2}$ ($\times10^{-2}$ mol mol-photons^{-1})	$\Phi_{app,\ ^3DOM^*_{Sorbate}}$ ($\times10^{-2}$ mol mol-photons^{-1})	$\Phi_{app,\ ^{\bullet}OH}$ ($\times10^{-6}$ mol mol-photons^{-1})
Ref 133	CM200	DBC (pyDOM)	NA	0.17	0.32	NA	NA
	CM230	DBC (pyDOM)	NA	0.25	0.59	NA	NA
	CM260	DBC (pyDOM)	NA	0.35	0.86	NA	NA
	SM200	DBC (pyDOM)	NA	0.02	0.02	NA	NA
	SM230	DBC (pyDOM)	NA	0.02	0.05	NA	NA
	SM260	DBC (pyDOM)	NA	0.04	0.06	NA	NA
Ref 134	DOM50_Model1	WEOM (Soil)	290-400	2.23	NA	NA	NA
	DOM50_Model2	WEOM (Soil)	290-400	1.55	NA	NA	NA
	DOM50_Model3	WEOM (Soil)	290-400	1.14	NA	NA	NA
	DOM250_Model1	WEOM (Soil)	290-400	2.05	NA	NA	NA
	DOM250_Model2	WEOM (Soil)	290-400	1.69	NA	NA	NA
	DOM250_Model3	WEOM (Soil)	290-400	1.45	NA	NA	NA
	DOM400_Model1	WEOM (Soil)	290-400	3.00	NA	NA	NA
	DOM400_Model2	WEOM (Soil)	290-400	2.54	NA	NA	NA
	DOM400_Model3	WEOM (Soil)	290-400	1.97	NA	NA	NA
	SRFA_Model1	IHSS Isolate (SRFA)	290-400	1.39	NA	NA	NA
	SRFA_Model2	IHSS Isolate (SRFA)	290-400	0.94	NA	NA	NA
	SRFA_Model3	IHSS Isolate (SRFA)	290-400	0.83	NA	NA	NA
Ref 135	YQH_Whole water	Whole Water (River)	290-400	5.26	10.80	NA	68.0
	YQH_100 mL loading	PPL Extract (River)	290-400	1.26	2.40	NA	18.0
	YQH_2 L loading	PPL Extract (River)	290-400	1.11	1.80	NA	21.0
	CH_Whole water	Whole Water (River)	290-400	56.44	69.80	NA	188.0
	CH_100 mL loading	PPL Extract (River)	290-400	6.37	7.20	NA	52.0
	CH_2 L loading	PPL Extract (River)	290-400	4.67	4.90	NA	42.0
	YDH_Whole water	Whole Water (River)	290-400	2.52	3.60	NA	16.0
	YDH_100 mL loading	PPL Extract (River)	290-400	0.56	0.60	NA	11.0
	YDH_2 L loading	PPL Extract (River)	290-400	0.44	0.40	NA	10.0
	QH_Whole water	Whole Water (WW-Impacted River)	290-400	5.41	6.50	NA	28.0
	QH_100 mL loading	PPL Extract (WW-Impacted River)	290-400	2.04	2.00	NA	17.0
	QH_2 L loading	PPL Extract (WW-Impacted River)	290-400	1.26	1.30	NA	14.0
	YMY_Whole water	Whole Water (WW-Impacted River)	290-400	17.26	29.30	NA	116.0
	YMY_100 mL loading	PPL Extract (WW-Impacted River)	290-400	1.93	2.40	NA	19.0
	YMY_2 L loading	PPL Extract (WW-Impacted River)	290-400	1.26	1.50	NA	12.0
	HGZ_Whole water	Whole Water (WW-Impacted River)	290-400	12.15	14.50	NA	81.0
	HGZ_100 mL loading	PPL Extract (WW-Impacted River)	290-400	1.81	2.00	NA	18.0
	HGZ_2 L loading	PPL Extract (WW-Impacted River)	290-400	1.33	1.60	NA	14.0
	HT_Whole water	Whole Water (WW-Impacted River)	290-400	33.33	73.30	NA	618.0
	HT_100 mL loading	PPL Extract (WW-Impacted River)	290-400	4.52	11.50	NA	78.0
	HT_2 L loading	PPL Extract (WW-Impacted River)	290-400	1.78	2.80	NA	59.0
	SRHA	IHSS Isolate (SRHA)	290-400	NA	1.40	NA	43.0
	SRFA	IHSS Isolate (SRFA)	290-400	NA	2.20	NA	60.0

Table S37. Summary of literature $\Phi_{app,RI}$ data (continued)							
Source	Sample ID	Sample Classification	Wavelength Range (nm)	$\Phi_{app,\ ^3DOM^*_{TMP}}$ (×10^{-2} mol mol-photons^{-1})	$\Phi_{app,\ ^1O_2}$ (×10^{-2} mol mol-photons^{-1})	$\Phi_{app,\ ^3DOM^*_{Sorbate}}$ (×10^{-2} mol mol-photons^{-1})	$\Phi_{app,\ ^{\cdot}OH}$ (×10^{-6} mol mol-photons^{-1})
This work	Lake 2 06/08/2019	Whole Water (Lake)	290-550	1.99±0.14	1.97±0.16	NA	14.1±0.3
	Lake 2 08/31/2019	Whole Water (Lake)	290-550	2.19±0.15	2.09±0.15	NA	18.5±1.0
	Lake 6 06/18/2019	Whole Water (Lake)	290-550	2.06±0.14	1.85±0.13	NA	18.5±1.0
	Lake 6 08/28/2019	Whole Water (Lake)	290-550	3.36±0.24	3.31±0.25	NA	23.3±1.1
	Lake 8 08/05/2019	Whole Water (Lake)	290-550	2.08±0.14	1.87±0.14	NA	15.0±0.8
	Lake 12 06/08/2019	Whole Water (Lake)	290-550	2.10±0.15	1.93±0.15	NA	13.6±0.4
	Lake 12 09/01/2019	Whole Water (Lake)	290-550	2.60±0.18	2.33±0.18	NA	20.9±0.6
	Lake 13 07/08/2018	Whole Water (Lake)	290-550	2.92±0.21	3.01±0.23	NA	19.9±1.1
	Lake 13 07/22/2018	Whole Water (Lake)	290-550	2.45±0.17	2.29±0.17	NA	16.5±0.8
	Lake 13 08/06/2018	Whole Water (Lake)	290-550	2.83±0.20	2.65±0.20	NA	19.2±0.8
	Lake 13 08/21/2018	Whole Water (Lake)	290-550	2.95±0.21	2.94±0.22	NA	19.6±1.2
	Lake 13 09/02/2018	Whole Water (Lake)	290-550	2.92±0.20	2.73±0.20	NA	22.0±1.1
	Lake 13 06/10/2019	Whole Water (Lake)	290-550	1.66±0.12	1.68±0.13	NA	12.3±0.4
	Lake 13 08/31/2019	Whole Water (Lake)	290-550	2.29±0.16	2.06±0.16	NA	15.4±0.7
	Lake 17 07/01/2018	Whole Water (Lake)	290-550	2.17±0.15	2.03±0.17	NA	18.3±0.4
	Lake 17 07/15/2018	Whole Water (Lake)	290-550	2.03±0.14	1.85±0.13	NA	14.3±1.1
	Lake 17 07/28/2018	Whole Water (Lake)	290-550	2.16±0.15	2.06±0.15	NA	18.7±1.0
	Lake 17 08/12/2018	Whole Water (Lake)	290-550	2.68±0.18	2.29±0.17	NA	21.7±1.1
	Lake 17 08/26/2018	Whole Water (Lake)	290-550	2.61±0.18	2.37±0.15	NA	19.6±1.6
	Lake 17 07/15/2019	Whole Water (Lake)	290-550	2.02±0.14	2.06±0.15	NA	19.8±1.0
	Lake 17 08/12/2019	Whole Water (Lake)	290-550	2.12±0.14	2.07±0.15	NA	20.0±1.0
	Lake 18 08/05/2018	Whole Water (Lake)	290-550	2.42±0.16	2.11±0.16	NA	17.4±0.7
	Lake 18 08/18/2018	Whole Water (Lake)	290-550	2.33±0.16	2.10±0.16	NA	23.5±1.0
	Lake 18 09/09/2018	Whole Water (Lake)	290-550	2.27±0.15	1.94±0.15	NA	18.6±0.9
	Lake 18 09/30/2018	Whole Water (Lake)	290-550	2.46±0.17	2.46±0.19	NA	20.4±0.9
	Lake 18 06/15/2019	Whole Water (Lake)	290-550	2.09±0.14	2.11±0.15	NA	19.0±1.1
	Lake 18 08/12/2019	Whole Water (Lake)	290-550	2.07±0.14	1.94±0.15	NA	16.6±0.7
	Lake 21 07/02/2019	Whole Water (Lake)	290-550	2.22±0.15	2.01±0.12	NA	19.2±1.6
	Lake 21 08/13/2019	Whole Water (Lake)	290-550	2.21±0.15	2.06±0.16	NA	19.1±0.8
	Lake 22 06/23/2019	Whole Water (Lake)	290-550	2.28±0.16	2.04±0.16	NA	18.8±0.8
	Lake 22 06/23/2019	Whole Water (Lake)	290-550	2.24±0.16	2.01±0.14	NA	15.7±1.0
	Lake 22 09/16/2019	Whole Water (Lake)	290-550	2.27±0.16	2.11±0.15	NA	18.8±1.2
	Lake 22 09/16/2019	Whole Water (Lake)	290-550	2.36±0.16	2.19±0.16	NA	18.4±1.0
	Lake 23 06/12/2019	Whole Water (Lake)	290-550	2.19±0.15	1.93±0.13	NA	16.0±1.0
	Lake 23 09/17/2019	Whole Water (Lake)	290-550	2.78±0.19	2.70±0.19	NA	21.4±1.4
	Lake 25 07/08/2018	Whole Water (Lake)	290-550	2.50±0.17	2.30±0.17	NA	22.8±1.2
	Lake 25 07/29/2018	Whole Water (Lake)	290-550	2.38±0.16	2.40±0.19	NA	19.4±0.7
	Lake 25 06/09/2019	Whole Water (Lake)	290-550	2.41±0.16	2.35±0.18	NA	19.4±0.7
	Lake 29 06/02/2019	Whole Water (Lake)	290-550	2.15±0.15	2.01±0.15	NA	18.0±0.9
	Lake 29 08/12/2019	Whole Water (Lake)	290-550	2.08±0.14	1.85±0.14	NA	14.6±0.6
	Lake 31 06/15/2019	Whole Water (Lake)	290-550	2.43±0.17	2.74±0.21	NA	17.3±0.7
	Lake 31 08/27/2019	Whole Water (Lake)	290-550	2.15±0.15	2.13±0.15	NA	20.3±1.3

Table S37. Summary of literature $\Phi_{app,RI}$ data (continued)							
Source	Sample ID	Sample Classification	Wavelength Range (nm)	$\Phi_{app,\ ^3DOM^*_{TMP}}$ ($\times10^{-2}$ mol mol-photons^{-1})	$\Phi_{app,\ ^1O_2}$ ($\times10^{-2}$ mol mol-photons^{-1})	$\Phi_{app,\ ^3DOM^*_{Sorbate}}$ ($\times10^{-2}$ mol mol-photons^{-1})	$\Phi_{app,\ ^\cdot OH}$ ($\times10^{-6}$ mol mol-photons^{-1})
This work	Lake 33 08/18/2019	Whole Water (Lake)	290-550	2.45±0.17	2.25±0.15	NA	20.5±1.5
	Lake 33 09/01/2019	Whole Water (Lake)	290-550	2.91±0.20	2.90±0.21	NA	22.8±1.3
	Lake 34 06/04/2019	Whole Water (Lake)	290-550	1.82±0.13	1.78±0.14	NA	14.3±0.4
	Lake 34 06/04/2019	Whole Water (Lake)	290-550	1.80±0.13	1.61±0.12	NA	11.9±0.5
	Lake 37 06/23/2019	Whole Water (Lake)	290-550	2.62±0.17	2.52±0.20	NA	24.5±0.9
	Lake 37 08/19/2019	Whole Water (Lake)	290-550	2.32±0.16	2.03±0.17	NA	21.6±0.7
	Lake 38 06/17/2019	Whole Water (Lake)	290-550	2.19±0.15	1.93±0.14	NA	17.7±0.8
	Lake 39 07/20/2019	Whole Water (Lake)	290-550	2.66±0.18	2.50±0.15	NA	22.6±2.1
	Lake 40 06/27/2018	Whole Water (Lake)	290-550	1.83±0.13	1.96±0.15	NA	17.3±0.8
	Lake 40 07/14/2018	Whole Water (Lake)	290-550	3.88±0.26	3.70±0.29	NA	31.4±1.2
	Lake 40 08/01/2018	Whole Water (Lake)	290-550	2.07±0.14	2.18±0.15	NA	21.1±1.5
	Lake 40 08/12/2018	Whole Water (Lake)	290-550	3.25±0.22	3.16±0.22	NA	25.3±1.6
	Lake 40 08/29/2018	Whole Water (Lake)	290-550	3.32±0.23	3.04±0.22	NA	24.2±1.6
	Lake 40 06/19/2019	Whole Water (Lake)	290-550	2.65±0.18	2.39±0.19	NA	24.7±0.8
	Lake 41 07/07/2018	Whole Water (Lake)	290-550	3.45±0.24	3.10±0.25	NA	22.1±0.7
	Lake 41 07/17/2018	Whole Water (Lake)	290-550	2.40±0.16	2.46±0.19	NA	21.6±0.9
	Lake 41 07/29/2018	Whole Water (Lake)	290-550	3.44±0.24	3.07±0.22	NA	25.5±1.4
	Lake 41 08/19/2018	Whole Water (Lake)	290-550	3.60±0.25	3.14±0.23	NA	25.7±1.4
	Lake 45 08/18/2019	Whole Water (Lake)	290-550	2.31±0.15	2.12±0.17	NA	23.2±0.8
	Lake 49 07/08/2019	Whole Water (Lake)	290-550	2.49±0.17	2.25±0.15	NA	20.7±1.5
	Lake 50 06/10/2019	Whole Water (Lake)	290-550	2.26±0.15	2.05±0.16	NA	18.9±0.7
	Lake 52 06/30/2019	Whole Water (Lake)	290-550	3.37±0.23	2.96±0.24	NA	25.2±0.8
	Lake 53 06/09/2019	Whole Water (Lake)	290-550	1.99±0.14	1.77±0.13	NA	14.4±0.9
	Lake 57 06/29/2019	Whole Water (Lake)	290-550	2.40±0.16	2.15±0.16	NA	21.3±0.8
	Lake 57 09/08/2019	Whole Water (Lake)	290-550	2.46±0.17	2.15±0.15	NA	22.0±1.4
	Lake 58 09/10/2019	Whole Water (Lake)	290-550	3.55±0.25	3.51±0.27	NA	25.4±1.3
	Lake 61 07/09/2018	Whole Water (Lake)	290-550	2.07±0.14	2.01±0.14	NA	18.9±1.2
	Lake 61 07/19/2018	Whole Water (Lake)	290-550	1.96±0.13	2.26±0.18	NA	14.1±0.4
	Lake 61 08/19/2018	Whole Water (Lake)	290-550	2.22±0.15	2.00±0.15	NA	18.6±1.0
	Lake 61 09/02/2018	Whole Water (Lake)	290-550	3.83±0.26	3.40±0.25	NA	27.2±1.5
	Lake 61 09/22/2018	Whole Water (Lake)	290-550	2.19±0.15	1.94±0.15	NA	15.2±0.7
	Lake 61 07/21/2019	Whole Water (Lake)	290-550	2.63±0.18	2.48±0.16	NA	23.2±1.7
	Lake 61 07/23/2019	Whole Water (Lake)	290-550	3.18±0.22	2.89±0.22	NA	24.2±1.2
	Lake 66 08/16/2019	Whole Water (Lake)	290-550	3.17±0.22	3.00±0.21	NA	21.8±1.6
	Lake 68 09/17/2019	Whole Water (Lake)	290-550	2.58±0.18	2.35±0.18	NA	19.5±0.9
	Lake 69 06/09/2019	Whole Water (Lake)	290-550	2.05±0.14	1.80±0.13	NA	14.6±0.8
	Lake 69 08/19/2019	Whole Water (Lake)	290-550	1.95±0.14	1.85±0.14	NA	19.2±0.9
	Lake 72 07/12/2019	Whole Water (Lake)	290-550	2.01±0.14	1.96±0.14	NA	17.7±1.1
	Lake 73 07/20/2019	Whole Water (Lake)	290-550	2.20±0.15	2.00±0.14	NA	21.9±1.3
	Lake 73 08/24/2019	Whole Water (Lake)	290-550	2.68±0.18	2.21±0.17	NA	23.7±0.9
	Lake 74 06/08/2019	Whole Water (Lake)	290-550	2.21±0.15	1.94±0.14	NA	16.6±1.0
	Lake 74 08/18/2019	Whole Water (Lake)	290-550	2.21±0.15	1.92±0.14	NA	17.4±0.9

Table S37. Summary of literature $\Phi_{app,RI}$ data (continued)							
Source	Sample ID	Sample Classification	Wavelength Range (nm)	$\Phi_{app,\ ^3DOM^*_{TMP}}$ ($\times10^{-2}$ mol mol-photons^{-1})	$\Phi_{app,\ ^1O_2}$ ($\times10^{-2}$ mol mol-photons^{-1})	$\Phi_{app,\ ^3DOM^*_{Sorbate}}$ ($\times10^{-2}$ mol mol-photons^{-1})	$\Phi_{app,\ ^{\bullet}OH}$ ($\times10^{-6}$ mol mol-photons^{-1})
This work	Lake 75 06/02/2019	Whole Water (Lake)	290-550	2.25±0.15	2.14±0.16	NA	20.0±0.8
	Lake 77 06/16/2019	Whole Water (Lake)	290-550	2.13±0.15	2.14±0.16	NA	13.9±1.0
	Lake 77 06/16/2019	Whole Water (Lake)	290-550	2.23±0.16	2.18±0.14	NA	22.0±1.8
	Lake 77 09/22/2019	Whole Water (Lake)	290-550	2.73±0.19	2.92±0.21	NA	18.7±1.6
	Lake 77 09/22/2019	Whole Water (Lake)	290-550	2.33±0.16	2.21±0.17	NA	16.8±0.8
	Lake 78 06/02/2019	Whole Water (Lake)	290-550	2.43±0.17	2.67±0.20	NA	16.0±1.0
	Lake 88 06/24/2019	Whole Water (Lake)	290-550	2.15±0.15	2.23±0.18	NA	20.2±0.6
	Lake 88 08/21/2019	Whole Water (Lake)	290-550	2.24±0.15	1.82±0.13	NA	15.1±0.8
	Lake 89 07/10/2019	Whole Water (Lake)	290-550	2.23±0.15	1.98±0.14	NA	18.2±1.1
	Lake 90 07/01/2018	Whole Water (Lake)	290-550	1.99±0.14	1.86±0.15	NA	16.9±0.5
	Lake 90 08/12/2018	Whole Water (Lake)	290-550	2.60±0.18	2.58±0.20	NA	20.4±0.8
	Lake 90 09/11/2018	Whole Water (Lake)	290-550	2.25±0.16	1.98±0.15	NA	17.3±1.0
	Lake 90 06/08/2019	Whole Water (Lake)	290-550	1.82±0.13	1.71±0.13	NA	13.9±0.6
	Lake 90 08/17/2019	Whole Water (Lake)	290-550	1.93±0.13	1.88±0.14	NA	15.9±0.7
	Lake 92 08/06/2018	Whole Water (Lake)	290-550	3.92±0.27	3.52±0.27	NA	27.3±1.4
	Lake 92 08/19/2018	Whole Water (Lake)	290-550	3.79±0.26	3.22±0.25	NA	26.0±1.1
	Lake 92 09/03/2018	Whole Water (Lake)	290-550	2.38±0.16	2.09±0.15	NA	19.9±1.0
	Lake 92 06/04/2019	Whole Water (Lake)	290-550	3.99±0.27	3.96±0.32	NA	34.0±1.2
	Lake 92 06/17/2019	Whole Water (Lake)	290-550	3.43±0.24	3.28±0.25	NA	25.9±1.2
	Lake 92 08/11/2019	Whole Water (Lake)	290-550	2.62±0.18	2.30±0.17	NA	21.4±1.0
	Lake 92 09/01/2019	Whole Water (Lake)	290-550	2.11±0.14	1.90±0.15	NA	14.7±0.6
	Lake 96 08/05/2019	Whole Water (Lake)	290-550	2.27±0.15	2.03±0.14	NA	20.6±1.2
	Lake 99 08/10/2019	Whole Water (Lake)	290-550	2.01±0.14	1.94±0.15	NA	13.8±0.6
	Lake 100 06/23/2019	Whole Water (Lake)	290-550	1.83±0.13	1.81±0.15	NA	14.7±0.4
	Lake 100 08/18/2019	Whole Water (Lake)	290-550	2.06±0.14	1.81±0.14	NA	15.0±0.7
	Lake 102 06/16/2019	Whole Water (Lake)	290-550	1.96±0.14	1.90±0.13	NA	16.3±1.1
	Lake 102 09/22/2019	Whole Water (Lake)	290-550	2.47±0.17	2.30±0.18	NA	21.5±0.9
	Lake 103 06/09/2019	Whole Water (Lake)	290-550	1.56±0.11	1.58±0.12	NA	10.8±0.6
	Lake 107 08/15/2019	Whole Water (Lake)	290-550	2.32±0.16	2.02±0.15	NA	18.3±0.9
	Lake 108 08/17/2019	Whole Water (Lake)	290-550	2.29±0.16	2.15±0.15	NA	19.9±1.4
	Lake 109 07/08/2019	Whole Water (Lake)	290-550	2.82±0.20	2.56±0.20	NA	23.4±0.7
	Lake 109 08/18/2019	Whole Water (Lake)	290-550	3.55±0.25	3.19±0.25	NA	25.5±1.1
	Lake 115 07/08/2019	Whole Water (Lake)	290-550	2.32±0.16	2.08±0.16	NA	18.3±0.9
	Lake 115 07/08/2019	Whole Water (Lake)	290-550	2.14±0.15	2.31±0.18	NA	21.0±0.8
	Lake 115 09/09/2019	Whole Water (Lake)	290-550	2.33±0.16	1.96±0.14	NA	17.3±0.9
	Lake 115 09/09/2019	Whole Water (Lake)	290-550	2.22±0.15	2.03±0.15	NA	18.3±0.9
	Lake 117 08/22/2019	Whole Water (Lake)	290-550	2.14±0.15	1.94±0.14	NA	15.9±0.8
	Lake 117 09/05/2019	Whole Water (Lake)	290-550	2.24±0.15	1.95±0.14	NA	17.8±1.0
	Lake 120 06/10/2019	Whole Water (Lake)	290-550	2.02±0.14	2.01±0.16	NA	17.9±0.5
	Lake 120 08/19/2019	Whole Water (Lake)	290-550	2.43±0.17	2.14±0.15	NA	19.0±1.2
	Lake 126 07/08/2018	Whole Water (Lake)	290-550	2.39±0.16	1.96±0.15	NA	16.0±0.7
	Lake 126 07/21/2018	Whole Water (Lake)	290-550	2.39±0.17	2.12±0.16	NA	16.4±0.9

Table S37. Summary of literature $\Phi_{app,RI}$ data (continued)							
Source	Sample ID	Sample Classification	Wavelength Range (nm)	$\Phi_{app, ^3DOM^*_{TMP}}$ ($\times10^{-2}$ mol mol-photons^{-1})	$\Phi_{app, ^1O_2}$ ($\times10^{-2}$ mol mol-photons^{-1})	$\Phi_{app, ^3DOM^*_{Sorbate}}$ ($\times10^{-2}$ mol mol-photons^{-1})	$\Phi_{app, ^{\cdot}OH}$ ($\times10^{-6}$ mol mol-photons^{-1})
This work	Lake 126 08/17/2019	Whole Water (Lake)	290-550	2.17±0.15	2.06±0.15	NA	19.8±1.1
	Lake 130 08/25/2019	Whole Water (Lake)	290-550	2.22±0.15	2.04±0.15	NA	21.7±1.0
	Lake 130 09/22/2019	Whole Water (Lake)	290-550	2.10±0.14	2.12±0.18	NA	19.4±0.4
	Lake 132 06/10/2019	Whole Water (Lake)	290-550	1.97±0.14	1.71±0.12	NA	13.5±0.9
	Lake 132 09/03/2019	Whole Water (Lake)	290-550	2.62±0.18	2.44±0.17	NA	19.4±1.2
	Lake 133 06/04/2019	Whole Water (Lake)	290-550	1.80±0.12	1.89±0.15	NA	15.8±0.6
	Lake 133 08/19/2019	Whole Water (Lake)	290-550	2.41±0.16	2.17±0.16	NA	20.8±0.9
	Lake 135 05/27/2019	Whole Water (Lake)	290-550	2.02±0.14	1.89±0.12	NA	14.8±1.3
	Lake 135 08/31/2019	Whole Water (Lake)	290-550	2.23±0.15	2.13±0.15	NA	19.8±1.2
	Lake 136 06/08/2019	Whole Water (Lake)	290-550	2.13±0.15	1.92±0.15	NA	16.9±0.8
	Lake 136 09/20/2019	Whole Water (Lake)	290-550	2.63±0.18	2.44±0.17	NA	20.6±1.3
	Lake 137 08/18/2019	Whole Water (Lake)	290-550	2.99±0.20	3.13±0.24	NA	24.1±0.9
	Lake 139 08/06/2018	Whole Water (Lake)	290-550	1.55±0.11	1.47±0.12	NA	10.7±0.4
	Lake 139 09/19/2018	Whole Water (Lake)	290-550	2.44±0.17	2.38±0.18	NA	15.1±0.8
	Lake 145 06/06/2019	Whole Water (Lake)	290-550	2.00±0.14	1.76±0.13	NA	14.3±0.8
	Lake 145 08/15/2019	Whole Water (Lake)	290-550	1.92±0.13	1.84±0.14	NA	15.2±0.9
	Lake 149 06/10/2019	Whole Water (Lake)	290-550	2.16±0.15	1.95±0.14	NA	17.5±1.1
	Lake 149 09/21/2019	Whole Water (Lake)	290-550	2.45±0.17	2.42±0.19	NA	21.8±0.9
	Lake 153 08/25/2019	Whole Water (Lake)	290-550	1.96±0.13	1.72±0.13	NA	13.8±0.7
	Lake 153 09/08/2019	Whole Water (Lake)	290-550	2.19±0.15	1.92±0.14	NA	16.2±0.8
	Lake 164 06/23/2019	Whole Water (Lake)	290-550	2.24±0.15	2.00±0.14	NA	20.9±1.2
	Lake 164 08/27/2019	Whole Water (Lake)	290-550	2.46±0.17	2.03±0.16	NA	22.7±0.7
	Lake 166 07/14/2019	Whole Water (Lake)	290-550	2.37±0.16	2.28±0.18	NA	21.6±0.7
	Lake 166 09/29/2019	Whole Water (Lake)	290-550	2.49±0.17	2.01±0.16	NA	18.4±0.6
	Lake 169 07/22/2019	Whole Water (Lake)	290-550	2.59±0.18	2.38±0.17	NA	20.8±1.1
	Lake 169 08/18/2019	Whole Water (Lake)	290-550	2.50±0.17	2.35±0.18	NA	22.1±1.0
	Lake 176 07/15/2018	Whole Water (Lake)	290-550	2.10±0.15	1.85±0.13	NA	15.1±1.1
	Lake 176 06/15/2019	Whole Water (Lake)	290-550	1.93±0.14	1.72±0.12	NA	13.6±0.9
	Lake 176 08/19/2019	Whole Water (Lake)	290-550	1.99±0.14	1.80±0.14	NA	14.9±0.5
	Lake 177 08/19/2019	Whole Water (Lake)	290-550	2.05±0.14	1.89±0.14	NA	16.4±0.8
	Lake 178 08/06/2019	Whole Water (Lake)	290-550	2.24±0.15	2.01±0.15	NA	19.4±1.0
	Lake 182 07/17/2018	Whole Water (Lake)	290-550	2.25±0.15	2.10±0.16	NA	20.0±0.9
	Lake 182 07/31/2018	Whole Water (Lake)	290-550	2.49±0.17	2.46±0.19	NA	23.0±0.8
	Lake 182 08/14/2018	Whole Water (Lake)	290-550	3.52±0.24	3.38±0.24	NA	25.3±1.8
	Lake 182 09/11/2018	Whole Water (Lake)	290-550	3.82±0.27	3.57±0.24	NA	25.2±2.3
	Lake 182 06/18/2019	Whole Water (Lake)	290-550	3.53±0.24	3.25±0.24	NA	25.2±1.3
	Lake 182 06/18/2019	Whole Water (Lake)	290-550	2.44±0.17	2.40±0.17	NA	22.0±1.4
	Lake 182 07/01/2019	Whole Water (Lake)	290-550	2.35±0.16	2.17±0.15	NA	17.3±1.3
	Lake 182 07/08/2019	Whole Water (Lake)	290-550	2.03±0.14	1.89±0.14	NA	16.1±0.8
	Lake 182 08/12/2019	Whole Water (Lake)	290-550	2.10±0.14	1.93±0.16	NA	16.6±0.3
	Lake 182 08/12/2019	Whole Water (Lake)	290-550	2.27±0.15	2.14±0.15	NA	20.3±1.2
	Lake 182 08/27/2019	Whole Water (Lake)	290-550	2.29±0.16	2.05±0.15	NA	19.7±1.0

Table S37. Summary of literature $\Phi_{app,RI}$ data (continued)							
Source	Sample ID	Sample Classification	Wavelength Range (nm)	$\Phi_{app,\ ^3DOM^*_{TMP}}$ ($\times 10^{-2}$ mol mol-photons^{-1})	$\Phi_{app,\ ^1O_2}$ ($\times 10^{-2}$ mol mol-photons^{-1})	$\Phi_{app,\ ^3DOM^*_{Sorbate}}$ ($\times 10^{-2}$ mol mol-photons^{-1})	$\Phi_{app,\ ^{\cdot}OH}$ ($\times 10^{-6}$ mol mol-photons^{-1})
This work	Lake 183 08/25/2019	Whole Water (Lake)	290-550	1.89±0.13	1.91±0.15	NA	15.0±0.7
	Lake 183 09/10/2019	Whole Water (Lake)	290-550	2.15±0.15	1.97±0.15	NA	18.5±0.8
	Lake 190 06/17/2019	Whole Water (Lake)	290-550	3.20±0.22	3.26±0.24	NA	24.3±1.4
	Lake 190 08/19/2019	Whole Water (Lake)	290-550	3.29±0.23	3.26±0.24	NA	24.2±1.3
	Lake 192 09/25/2019	Whole Water (Lake)	290-550	2.05±0.14	1.89±0.13	NA	18.2±1.3
	Lake 194 06/26/2018	Whole Water (Lake)	290-550	2.13±0.15	1.97±0.15	NA	19.5±0.8
	Lake 194 07/09/2018	Whole Water (Lake)	290-550	2.17±0.15	1.89±0.14	NA	16.6±0.8
	Lake 194 07/23/2018	Whole Water (Lake)	290-550	2.43±0.17	2.25±0.17	NA	15.5±0.8
	Lake 194 08/07/2018	Whole Water (Lake)	290-550	2.38±0.17	2.27±0.16	NA	15.9±1.0
	Lake 194 08/20/2018	Whole Water (Lake)	290-550	2.28±0.16	2.09±0.16	NA	18.7±0.6
	Lake 194 09/09/2018	Whole Water (Lake)	290-550	2.32±0.16	2.18±0.17	NA	18.4±0.8
	Lake 194 09/22/2019	Whole Water (Lake)	290-550	2.45±0.17	2.47±0.18	NA	22.8±1.4
	Lake 195 06/03/2019	Whole Water (Lake)	290-550	1.81±0.13	1.58±0.12	NA	11.1±0.8
	Lake 199 06/19/2018	Whole Water (Lake)	290-550	2.69±0.18	2.39±0.19	NA	21.1±0.6
	Lake 199 07/03/2018	Whole Water (Lake)	290-550	1.71±0.12	1.62±0.12	NA	11.2±0.6
	Lake 199 07/18/2018	Whole Water (Lake)	290-550	4.22±0.28	4.15±0.30	NA	34.2±1.7
	Lake 199 07/31/2018	Whole Water (Lake)	290-550	3.02±0.20	2.64±0.20	NA	22.7±1.1
	Lake 199 08/14/2018	Whole Water (Lake)	290-550	1.54±0.11	1.49±0.11	NA	11.8±0.6
	Lake 199 08/28/2018	Whole Water (Lake)	290-550	2.17±0.15	1.92±0.15	NA	15.5±0.6
	Lake 199 09/11/2018	Whole Water (Lake)	290-550	3.14±0.21	3.04±0.22	NA	25.2±1.6
	Lake 199 06/23/2019	Whole Water (Lake)	290-550	3.67±0.25	3.43±0.24	NA	27.4±1.8
	Lake 199 07/08/2019	Whole Water (Lake)	290-550	1.86±0.13	1.73±0.13	NA	13.0±0.5
	Lake 199 07/30/2019	Whole Water (Lake)	290-550	3.14±0.21	2.71±0.21	NA	23.0±0.8
	Lake 199 08/18/2019	Whole Water (Lake)	290-550	2.50±0.17	2.29±0.18	NA	22.1±0.8
	Lake 199 09/15/2019	Whole Water (Lake)	290-550	4.24±0.29	3.79±0.29	NA	34.1±1.3
	Lake 203 06/16/2019	Whole Water (Lake)	290-550	1.81±0.13	1.73±0.13	NA	13.9±0.7
	Lake 203 09/22/2019	Whole Water (Lake)	290-550	2.15±0.15	1.98±0.15	NA	17.0±0.8
	Lake 205 06/08/2019	Whole Water (Lake)	290-550	2.16±0.15	1.97±0.15	NA	14.9±0.5
	Lake 205 08/06/2019	Whole Water (Lake)	290-550	2.08±0.14	1.99±0.15	NA	16.6±0.7
	Lake 209 08/18/2019	Whole Water (Lake)	290-550	2.01±0.14	1.80±0.13	NA	14.1±0.7
	Lake 210 06/02/2019	Whole Water (Lake)	290-550	1.72±0.12	1.56±0.12	NA	11.6±0.7
	Lake 210 08/18/2019	Whole Water (Lake)	290-550	1.56±0.11	1.50±0.11	NA	11.1±0.7
	Lake 212 06/16/2018	Whole Water (Lake)	290-550	2.43±0.17	2.12±0.14	NA	20.0±1.4
	Lake 212 07/14/2018	Whole Water (Lake)	290-550	2.29±0.16	2.34±0.18	NA	16.5±0.8
	Lake 212 07/28/2018	Whole Water (Lake)	290-550	2.42±0.17	2.47±0.18	NA	16.6±1.0
	Lake 212 08/12/2018	Whole Water (Lake)	290-550	2.34±0.16	2.14±0.15	NA	17.5±1.2
	Lake 212 06/17/2019	Whole Water (Lake)	290-550	2.18±0.15	2.46±0.19	NA	15.1±0.5
	Lake 213 08/18/2019	Whole Water (Lake)	290-550	2.67±0.18	2.30±0.16	NA	22.3±1.4
	Lake 215 06/09/2019	Whole Water (Lake)	290-550	2.45±0.17	2.67±0.20	NA	16.4±0.9
	Lake 215 09/15/2019	Whole Water (Lake)	290-550	2.86±0.20	2.36±0.18	NA	19.5±0.8
	Lake 223 08/19/2019	Whole Water (Lake)	290-550	2.64±0.19	2.48±0.19	NA	17.1±0.6
	Lake 225 07/28/2019	Whole Water (Lake)	290-550	1.98±0.14	2.03±0.16	NA	14.3±0.5

Table S37. Summary of literature $\Phi_{app,RI}$ data (continued)							
Source	Sample ID	Sample Classification	Wavelength Range (nm)	$\Phi_{app, {}^3DOM^*_{TMP}}$ ($\times10^{-2}$ mol mol-photons^{-1})	$\Phi_{app, {}^1O_2}$ ($\times10^{-2}$ mol mol-photons^{-1})	$\Phi_{app, {}^3DOM^*_{Sorbate}}$ ($\times10^{-2}$ mol mol-photons^{-1})	$\Phi_{app, {}^{\bullet}OH}$ ($\times10^{-6}$ mol mol-photons^{-1})
This work	Lake 229 06/29/2019	Whole Water (Lake)	290-550	1.91±0.13	1.93±0.14	NA	15.8±1.0
	Lake 230 06/08/2019	Whole Water (Lake)	290-550	2.44±0.17	2.26±0.17	NA	23.5±0.9
	Lake 231 06/04/2019	Whole Water (Lake)	290-550	2.10±0.15	1.93±0.14	NA	15.6±1.0
	Lake 234 06/20/2018	Whole Water (Lake)	290-550	3.35±0.23	2.91±0.21	NA	24.0±1.3
	Lake 234 07/15/2018	Whole Water (Lake)	290-550	3.24±0.22	3.11±0.22	NA	25.1±1.6
	Lake 234 06/09/2019	Whole Water (Lake)	290-550	1.97±0.14	1.84±0.14	NA	14.6±0.8
	Lake 234 08/12/2019	Whole Water (Lake)	290-550	1.97±0.14	1.93±0.14	NA	14.6±0.8
	Lake 235 07/14/2019	Whole Water (Lake)	290-550	1.99±0.14	1.76±0.13	NA	14.5±0.6
	Lake 236 07/14/2019	Whole Water (Lake)	290-550	2.70±0.19	2.86±0.22	NA	21.1±1.0
	Lake 236 09/15/2019	Whole Water (Lake)	290-550	2.23±0.15	2.08±0.16	NA	24.2±0.9
	Lake 238 08/12/2019	Whole Water (Lake)	290-550	2.49±0.17	2.53±0.19	NA	21.2±1.0
	Lake 239 06/09/2019	Whole Water (Lake)	290-550	2.39±0.16	2.19±0.17	NA	22.9±0.9
	Lake 239 08/18/2019	Whole Water (Lake)	290-550	2.53±0.17	2.28±0.17	NA	19.7±1.0
	Lake 245 09/22/2019	Whole Water (Lake)	290-550	2.39±0.16	2.09±0.16	NA	17.4±0.8
	Lake 246 08/05/2019	Whole Water (Lake)	290-550	2.36±0.16	2.03±0.16	NA	21.3±0.8
	Lake 247 06/08/2019	Whole Water (Lake)	290-550	2.04±0.14	1.95±0.14	NA	18.2±1.1
	Lake 247 09/20/2019	Whole Water (Lake)	290-550	2.25±0.15	2.27±0.17	NA	21.7±0.9
	Lake 248 08/18/2019	Whole Water (Lake)	290-550	2.07±0.15	1.91±0.14	NA	16.4±1.1
	Lake 249 07/08/2019	Whole Water (Lake)	290-550	2.02±0.14	1.91±0.13	NA	17.8±1.4
	Lake 249 09/01/2019	Whole Water (Lake)	290-550	2.03±0.14	1.95±0.14	NA	15.4±0.9
	Lake 250 07/08/2019	Whole Water (Lake)	290-550	2.62±0.18	2.35±0.18	NA	22.4±1.0
	Lake 250 08/18/2019	Whole Water (Lake)	290-550	1.74±0.12	1.85±0.14	NA	15.2±0.6
	Lake 251 08/27/2019	Whole Water (Lake)	290-550	2.48±0.17	2.42±0.18	NA	20.6±1.0
	Lake 251 09/17/2019	Whole Water (Lake)	290-550	2.74±0.19	2.48±0.17	NA	20.5±1.6
	Lake 253 07/02/2019	Whole Water (Lake)	290-550	2.40±0.16	2.40±0.17	NA	21.0±1.1
	Lake 253 08/25/2019	Whole Water (Lake)	290-550	3.66±0.25	3.44±0.24	NA	28.3±2.0
	Lake 253 09/15/2019	Whole Water (Lake)	290-550	2.27±0.15	1.98±0.13	NA	20.0±1.4
	Lake 256 07/05/2018	Whole Water (Lake)	290-550	3.11±0.21	2.97±0.21	NA	23.4±1.4
	Lake 256 07/16/2018	Whole Water (Lake)	290-550	2.74±0.19	2.85±0.23	NA	20.2±0.7
	Lake 256 07/31/2018	Whole Water (Lake)	290-550	2.50±0.17	2.22±0.17	NA	17.2±0.8
	Lake 256 08/15/2018	Whole Water (Lake)	290-550	2.78±0.19	2.67±0.21	NA	16.9±0.8
	Lake 256 08/29/2018	Whole Water (Lake)	290-550	2.62±0.18	2.58±0.20	NA	18.5±0.8
	Lake 256 09/14/2018	Whole Water (Lake)	290-550	2.42±0.16	2.35±0.17	NA	22.3±1.1
	Lake 256 09/28/2018	Whole Water (Lake)	290-550	2.53±0.17	2.34±0.17	NA	24.0±1.2
	Lake 256 06/18/2019	Whole Water (Lake)	290-550	1.73±0.12	1.60±0.12	NA	12.1±0.5
	Lake 256 06/18/2019	Whole Water (Lake)	290-550	2.33±0.16	2.21±0.15	NA	16.6±1.1
	Lake 256 08/13/2019	Whole Water (Lake)	290-550	2.39±0.16	2.10±0.15	NA	19.7±1.1
	Lake 257 08/24/2019	Whole Water (Lake)	290-550	2.42±0.17	2.24±0.16	NA	22.0±1.4
	Lake 258 06/16/2019	Whole Water (Lake)	290-550	2.27±0.16	2.39±0.17	NA	20.5±1.4
	Lake 258 08/11/2019	Whole Water (Lake)	290-550	2.35±0.16	2.19±0.15	NA	20.1±1.4
	Lake 259 09/01/2019	Whole Water (Lake)	290-550	4.08±0.29	3.63±0.26	NA	29.1±2.1
	Lake 260 06/01/2019	Whole Water (Lake)	290-550	1.96±0.14	1.72±0.13	NA	14.0±0.6

**Table S37.** Summary of literature $\Phi_{app,RI}$ data (continued)							
Source	Sample ID	Sample Classification	Wavelength Range (nm)	$\Phi_{app,\ ^3DOM^*_{TMP}}$ ($\times10^{-2}$ mol mol-photons^{-1})	$\Phi_{app,\ ^1O_2}$ ($\times10^{-2}$ mol mol-photons^{-1})	$\Phi_{app,\ ^3DOM^*_{Sorbate}}$ ($\times10^{-2}$ mol mol-photons^{-1})	$\Phi_{app,\ ^\bullet OH}$ ($\times10^{-6}$ mol mol-photons^{-1})
This work	Lake 260 08/12/2019	Whole Water (Lake)	290-550	2.47±0.17	2.10±0.15	NA	18.8±1.0
	Lake 261 06/17/2019	Whole Water (Lake)	290-550	2.66±0.19	2.31±0.17	NA	20.4±1.2
	Lake 261 08/12/2019	Whole Water (Lake)	290-550	2.35±0.16	2.14±0.17	NA	22.0±0.5
	Lake 262 06/17/2019	Whole Water (Lake)	290-550	1.83±0.13	1.81±0.14	NA	11.9±0.7
	Lake 262 09/21/2019	Whole Water (Lake)	290-550	1.78±0.12	1.92±0.15	NA	14.7±0.5
	Lake 261 09/04/2021_Lysate A	Bloom Lysate	290-550	1.00±0.07	0.92±0.09	0.62±0.05	6.3±0.8
	Lake 238 09/07/2021 Lysate B	Bloom Lysate	290-550	1.02±0.06	0.93±0.09	0.62±0.04	6.3±0.7
	Lake 147 08/30/2021 Lysate C	Bloom Lysate	290-550	1.07±0.08	1.00±0.08	0.65±0.05	6.2±0.5
	Lake 138 09/07/2021 Lysate D	Bloom Lysate	290-550	1.05±0.07	0.99±0.09	0.57±0.05	6.5±0.7
	Lake 33 09/04/2021 Lysate E	Bloom Lysate	290-550	0.96±0.08	0.84±0.07	0.55±0.04	6.4±0.6
	Lake 37 08/20/2021_Lysate F	Bloom Lysate	290-550	0.87±0.01	0.82±0.07	0.51±0.03	6.1±0.4
	Lake 38 08/29/2021 Lysate G	Bloom Lysate	290-550	1.21±0.06	1.19±0.10	0.78±0.06	7.8±1.1
	Lake 40 09/16/2021 Lysate H	Bloom Lysate	290-550	1.22±0.05	1.23±0.12	0.78±0.06	7.7±0.9
	Lake 78 08/22/2021 Lysate I	Bloom Lysate	290-550	1.21±0.06	1.14±0.12	0.79±0.06	7.1±0.9
	Lake 82 08/17/2021 Lysate J	Bloom Lysate	290-550	1.29±0.04	1.30±0.14	0.74±0.05	8.7±0.9
	Lake 93 08/29/2021_Lysate K	Bloom Lysate	290-550	0.89±0.10	0.88±0.07	0.50±0.05	6.3±0.7
	Lake 221 09/06/2021 Lysate L	Bloom Lysate	290-550	1.32±0.11	1.35±0.11	0.75±0.06	9.3±1.1
	Lake 256 10/12/2021 Otisco	Whole Water (Lake)	290-550	1.75±0.01	1.72±0.07	1.28±0.11	13.9±0.8
	Lysate A 25% + Otisco 75%	Whole Water (Lake, Bloom Lysate)	290-550	1.34±0.03	1.25±0.12	0.91±0.07	9.8±0.6
	Lysate A 50% + Otisco 50%	Whole Water (Lake, Bloom Lysate)	290-550	1.14±0.11	1.03±0.09	0.72±0.05	8.1±0.7
	Lysate A 75% + Otisco 25%	Whole Water (Lake, Bloom Lysate)	290-550	1.00±0.05	0.88±0.03	0.59±0.04	6.9±0.6
	Lysate B 25% + Otisco 75%	Whole Water (Lake, Bloom Lysate)	290-550	1.34±0.04	1.29±0.11	0.90±0.07	10.1±0.7
	Lysate B 50% + Otisco 50%	Whole Water (Lake, Bloom Lysate)	290-550	1.12±0.04	1.07±0.10	0.71±0.05	8.3±0.7
	Lysate B 75% + Otisco 25%	Whole Water (Lake, Bloom Lysate)	290-550	0.94±0.05	0.89±0.09	0.56±0.04	6.8±0.5
	Lysate C 25% + Otisco 75%	Whole Water (Lake, Bloom Lysate)	290-550	1.37±0.04	1.31±0.14	0.95±0.06	9.8±0.8
	Lysate C 50% + Otisco 50%	Whole Water (Lake, Bloom Lysate)	290-550	1.18±0.04	1.11±0.09	0.78±0.04	8.0±0.8
	Lysate C 75% + Otisco 25%	Whole Water (Lake, Bloom Lysate)	290-550	1.05±0.03	0.97±0.09	0.66±0.04	6.8±0.8
	Lysate D 25% + Otisco 75%	Whole Water (Lake, Bloom Lysate)	290-550	1.40±0.05	1.37±0.13	0.95±0.08	9.7±0.7
	Lysate D 50% + Otisco 50%	Whole Water (Lake, Bloom Lysate)	290-550	1.22±0.03	1.18±0.11	0.78±0.06	7.9±0.8
	Lysate D 75% + Otisco 25%	Whole Water (Lake, Bloom Lysate)	290-550	1.10±0.06	1.05±0.02	0.66±0.05	6.7±0.5
	Lysate E 25% + Otisco 75%	Whole Water (Lake, Bloom Lysate)	290-550	1.38±0.06	1.31±0.11	0.97±0.09	9.8±0.9
	Lysate E 50% + Otisco 50%	Whole Water (Lake, Bloom Lysate)	290-550	1.19±0.05	1.11±0.06	0.80±0.07	8.0±0.8
	Lysate E 75% + Otisco 25%	Whole Water (Lake, Bloom Lysate)	290-550	1.07±0.02	0.97±0.09	0.69±0.06	6.7±0.4
	Lysate F 25% + Otisco 75%	Whole Water (Lake, Bloom Lysate)	290-550	1.41±0.05	1.37±0.11	0.92±0.08	10.2±0.7
	Lysate F 50% + Otisco 50%	Whole Water (Lake, Bloom Lysate)	290-550	1.23±0.05	1.18±0.09	0.75±0.06	8.4±0.5
	Lysate F 75% + Otisco 25%	Whole Water (Lake, Bloom Lysate)	290-550	1.09±0.06	1.04±0.09	0.61±0.05	7.1±0.5
	SRNOM	IHSS Isolate (SRNOM)	290-550	2.68±0.14	2.57±0.23	1.26±0.10	21.5±1.3
	Lysate A 25% + SRNOM 75%	IHSS Isolate (SRNOM, Bloom Lysate)	290-550	1.99±0.15	1.90±0.12	0.95±0.07	15.0±1.4
	Lysate A 50% + SRNOM 50%	IHSS Isolate (SRNOM, Bloom Lysate)	290-550	1.57±0.16	1.46±0.08	0.79±0.06	11.7±0.9
	Lysate A 75% + SRNOM 25%	IHSS Isolate (SRNOM, Bloom Lysate)	290-550	1.18±0.13	1.05±0.04	0.62±0.05	8.6±0.6
	Lysate B 25% + SRNOM 75%	IHSS Isolate (SRNOM, Bloom Lysate)	290-550	1.91±0.09	1.93±0.10	0.93±0.07	15.0±1.3
	Lysate B 50% + SRNOM 50%	IHSS Isolate (SRNOM, Bloom Lysate)	290-550	1.46±0.03	1.44±0.07	0.77±0.05	11.2±1.0

**Table S37.** Summary of literature $\Phi_{app,RI}$ data (continued)							
Source	Sample ID	Sample Classification	Wavelength Range (nm)	$\Phi_{app,\ ^3DOM^*_{TMP}}$ ($\times10^{-2}$ mol mol-photons^{-1})	$\Phi_{app,\ ^1O_2}$ ($\times10^{-2}$ mol mol-photons^{-1})	$\Phi_{app,\ ^3DOM^*_{Sorbate}}$ ($\times10^{-2}$ mol mol-photons^{-1})	$\Phi_{app,\ ^\bullet OH}$ ($\times10^{-6}$ mol mol-photons^{-1})
This work	Lysate B 75% + SRNOM 25%	IHSS Isolate (SRNOM, Bloom Lysate)	290-550	1.08±0.04	1.02±0.05	0.60±0.04	8.3±0.3
	Lysate C 25% + SRNOM 75%	IHSS Isolate (SRNOM, Bloom Lysate)	290-550	1.95±0.11	1.90±0.16	1.00±0.06	15.1±1.1
	Lysate C 50% + SRNOM 50%	IHSS Isolate (SRNOM, Bloom Lysate)	290-550	1.53±0.08	1.55±0.08	0.85±0.05	11.9±1.2
	Lysate C 75% + SRNOM 25%	IHSS Isolate (SRNOM, Bloom Lysate)	290-550	1.16±0.08	1.15±0.04	0.69±0.04	8.8±0.5
	Lysate D 25% + SRNOM 75%	IHSS Isolate (SRNOM, Bloom Lysate)	290-550	1.99±0.14	1.96±0.11	1.03±0.08	15.0±1.3
	Lysate D 50% + SRNOM 50%	IHSS Isolate (SRNOM, Bloom Lysate)	290-550	1.60±0.05	1.57±0.07	0.87±0.07	11.4±0.8
	Lysate D 75% + SRNOM 25%	IHSS Isolate (SRNOM, Bloom Lysate)	290-550	1.24±0.08	1.18±0.04	0.70±0.05	8.3±0.5
	Lysate E 25% + SRNOM 75%	IHSS Isolate (SRNOM, Bloom Lysate)	290-550	1.94±0.09	1.90±0.11	1.01±0.08	15.2±1.3
	Lysate E 50% + SRNOM 50%	IHSS Isolate (SRNOM, Bloom Lysate)	290-550	1.54±0.07	1.44±0.08	0.84±0.07	11.8±0.8
	Lysate E 75% + SRNOM 25%	IHSS Isolate (SRNOM, Bloom Lysate)	290-550	1.17±0.04	1.07±0.11	0.68±0.06	8.7±0.4
	Lysate F 25% + SRNOM 75%	IHSS Isolate (SRNOM, Bloom Lysate)	290-550	1.98±0.11	1.98±0.14	0.92±0.07	15.4±1.6
	Lysate F 50% + SRNOM 50%	IHSS Isolate (SRNOM, Bloom Lysate)	290-550	1.58±0.09	1.53±0.10	0.78±0.06	12.2±1.2
	Lysate F 75% + SRNOM 25%	IHSS Isolate (SRNOM, Bloom Lysate)	290-550	1.22±0.08	1.15±0.07	0.64±0.05	9.3±0.9
	Lake 261 09/04/2021_Supernatant A_1	Bloom Supernatant	290-550	2.58±0.13	2.95±0.11	NA	20.9±1.8
	Lake 261 09/04/2021_Supernatant A_2	Bloom Supernatant	290-550	3.06±0.10	3.48±0.14	NA	24.5±1.3
	Lake 261 09/04/2021_Supernatant A_3	Bloom Supernatant	290-550	3.47±0.12	3.92±0.27	NA	26.8±1.5
	Lake 261 09/04/2021_Supernatant A_4	Bloom Supernatant	290-550	3.83±0.19	4.23±0.30	NA	28.9±1.8
	Lake 261 09/04/2021_Supernatant A_5	Bloom Supernatant	290-550	4.02±0.17	4.44±0.31	NA	30.5±1.1
	Lake 261 09/04/2021_Supernatant A_6	Bloom Supernatant	290-550	4.12±0.22	4.56±0.32	NA	31.3±1.9
	Lake 261 09/04/2021_Supernatant A_7	Bloom Supernatant	290-550	4.18±0.18	4.62±0.35	NA	31.6±1.4
	Lake 261 09/04/2021_Supernatant A_8	Bloom Supernatant	290-550	4.20±0.21	4.63±0.29	NA	31.7±0.9
	Lake 261 09/04/2021_Supernatant A_9	Bloom Supernatant	290-550	4.21±0.27	4.63±0.33	3.71±0.37	31.7±1.4
	Lake 238 09/07/2021_Supernatant B_1	Bloom Supernatant	290-550	2.54±0.03	2.98±0.26	NA	21.3±1.9
	Lake 238 09/07/2021_Supernatant B_2	Bloom Supernatant	290-550	3.04±0.03	3.52±0.25	NA	23.5±1.4
	Lake 238 09/07/2021_Supernatant B_3	Bloom Supernatant	290-550	3.50±0.04	4.02±0.30	NA	25.5±1.4
	Lake 238 09/07/2021_Supernatant B_4	Bloom Supernatant	290-550	3.88±0.16	4.42±0.30	NA	27.3±1.3
	Lake 238 09/07/2021_Supernatant B_5	Bloom Supernatant	290-550	4.10±0.03	4.65±0.31	NA	28.8±1.1
	Lake 238 09/07/2021_Supernatant B_6	Bloom Supernatant	290-550	4.22±0.09	4.81±0.39	NA	30.2±0.7
	Lake 238 09/07/2021_Supernatant B_7	Bloom Supernatant	290-550	4.30±0.06	4.84±0.39	NA	30.5±1.3
	Lake 238 09/07/2021_Supernatant B_8	Bloom Supernatant	290-550	4.34±0.09	4.85±0.38	NA	30.7±1.0
	Lake 238 09/07/2021_Supernatant B_9	Bloom Supernatant	290-550	4.35±0.01	4.86±0.38	3.61±0.30	30.7±1.3
	Lake 147 08/30/2021_Supernatant C_1	Bloom Supernatant	290-550	2.95±0.18	3.36±0.23	NA	21.5±1.3
	Lake 147 08/30/2021_Supernatant C_2	Bloom Supernatant	290-550	3.81±0.20	4.39±0.26	NA	26.2±1.6
	Lake 147 08/30/2021_Supernatant C_3	Bloom Supernatant	290-550	4.44±0.20	4.98±0.33	NA	28.7±1.7
	Lake 147 08/30/2021_Supernatant C_4	Bloom Supernatant	290-550	4.78±0.16	5.40±0.32	NA	30.9±1.2
	Lake 147 08/30/2021_Supernatant C_5	Bloom Supernatant	290-550	5.00±0.22	5.56±0.34	NA	31.9±1.0
	Lake 147 08/30/2021_Supernatant C_6	Bloom Supernatant	290-550	5.10±0.23	5.65±0.32	NA	32.6±0.8
	Lake 147 08/30/2021_Supernatant C_7	Bloom Supernatant	290-550	5.18±0.29	5.69±0.34	NA	32.9±0.9
	Lake 147 08/30/2021_Supernatant C_8	Bloom Supernatant	290-550	5.21±0.29	5.71±0.39	NA	33.1±0.9
	Lake 147 08/30/2021_Supernatant C_9	Bloom Supernatant	290-550	5.22±0.27	5.73±0.41	4.00±0.37	33.1±0.6
	Lake 138 09/07/2021_Supernatant D_1	Bloom Supernatant	290-550	2.86±0.14	3.28±0.26	NA	21.1±1.0
	Lake 138 09/07/2021_Supernatant D_2	Bloom Supernatant	290-550	3.72±0.09	4.30±0.33	NA	27.6±1.4

Table S37. Summary of literature $\Phi_{app,RI}$ data (continued)

Source	Sample ID	Sample Classification	Wavelength Range (nm)	$\Phi_{app,\ ^3DOM^*_{TMP}}$ ($\times10^{-2}$ mol mol-photons^{-1})	$\Phi_{app,\ ^1O_2}$ ($\times10^{-2}$ mol mol-photons^{-1})	$\Phi_{app,\ ^3DOM^*_{Sorbate}}$ ($\times10^{-2}$ mol mol-photons^{-1})	$\Phi_{app,\ ^{\bullet}OH}$ ($\times10^{-6}$ mol mol-photons^{-1})
This work	Lake 138 09/07/2021_Supernatant D_3	Bloom Supernatant	290-550	4.36±0.13	4.90±0.36	NA	30.2±1.9
	Lake 138 09/07/2021_Supernatant D_4	Bloom Supernatant	290-550	4.72±0.11	5.19±0.38	NA	31.7±1.2
	Lake 138 09/07/2021_Supernatant D_5	Bloom Supernatant	290-550	4.90±0.17	5.34±0.43	NA	32.7±0.9
	Lake 138 09/07/2021_Supernatant D_6	Bloom Supernatant	290-550	5.00±0.15	5.42±0.42	NA	33.2±1.5
	Lake 138 09/07/2021_Supernatant D_7	Bloom Supernatant	290-550	5.06±0.17	5.45±0.32	NA	33.5±1.0
	Lake 138 09/07/2021_Supernatant D_8	Bloom Supernatant	290-550	5.08±0.14	5.47±0.31	NA	33.7±1.0
	Lake 138 09/07/2021_Supernatant D_9	Bloom Supernatant	290-550	5.09±0.18	5.48±0.33	3.75±0.33	33.8±1.4
	Lake 33 09/04/2021_Supernatant E	Bloom Supernatant	290-550	3.83±0.48	3.84±0.25	3.48±0.35	31.6±1.7
	Lake 37 08/20/2021_Supernatant F	Bloom Supernatant	290-550	3.49±0.01	3.36±0.29	3.29±0.33	27.4±2.3
	Lake 38 08/29/2021_Supernatant G	Bloom Supernatant	290-550	4.86±0.31	5.10±0.31	4.34±0.44	35.2±2.5
	Lake 40 09/16/2021_Supernatant H	Bloom Supernatant	290-550	4.89±0.15	5.34±0.32	3.91±0.36	39.0±3.1
	Lake 78 08/22/2021_Supernatant I	Bloom Supernatant	290-550	4.71±0.21	5.01±0.32	4.13±0.39	36.0±2.3
	Lake 82 08/17/2021_Supernatant J	Bloom Supernatant	290-550	5.17±0.07	5.66±0.39	4.00±0.34	41.9±2.9
	Lake 93 08/29/2021_Supernatant K	Bloom Supernatant	290-550	3.51±0.03	3.48±0.18	3.18±0.31	28.4±2.3
	Lake 221 09/06/2021_Supernatant L	Bloom Supernatant	290-550	6.58±0.44	6.83±0.43	4.95±0.45	48.8±2.7
	Lake 256 10/12/2021_Otisco_1-1	Whole Water (Lake)	290-550	1.87±0.04	1.82±0.08	NA	13.9±0.8
	Lake 256 10/12/2021_Otisco_1-2	Whole Water (Lake)	290-550	1.85±0.02	1.81±0.05	NA	13.8±0.9
	Lake 256 10/12/2021_Otisco_1-3	Whole Water (Lake)	290-550	1.84±0.01	1.80±0.04	NA	13.8±0.5
	Lake 256 10/12/2021_Otisco_1-4	Whole Water (Lake)	290-550	1.83±0.03	1.79±0.06	NA	13.8±0.7
	Lake 256 10/12/2021_Otisco_1-5	Whole Water (Lake)	290-550	1.81±0.01	1.78±0.06	NA	13.7±0.3
	Lake 256 10/12/2021_Otisco_1-6	Whole Water (Lake)	290-550	1.79±0.01	1.77±0.07	NA	13.6±0.3
	Lake 256 10/12/2021_Otisco_1-7	Whole Water (Lake)	290-550	1.77±0.03	1.75±0.09	NA	13.6±0.4
	Lake 256 10/12/2021_Otisco_1-8	Whole Water (Lake)	290-550	1.76±0.01	1.74±0.08	NA	13.5±0.9
	Lake 256 10/12/2021_Otisco_1-9	Whole Water (Lake)	290-550	1.75±0.02	1.72±0.08	1.28±0.11	13.5±1.1
	Lake 256 10/12/2021_Otisco_2-1	Whole Water (Lake)	290-550	1.87±0.05	1.83±0.08	NA	13.9±0.8
	Lake 256 10/12/2021_Otisco_2-2	Whole Water (Lake)	290-550	1.86±0.02	1.81±0.06	NA	13.9±0.8
	Lake 256 10/12/2021_Otisco_2-3	Whole Water (Lake)	290-550	1.84±0.02	1.80±0.08	NA	13.8±1.1
	Lake 256 10/12/2021_Otisco_2-4	Whole Water (Lake)	290-550	1.83±0.02	1.79±0.06	NA	13.8±1.1
	Lake 256 10/12/2021_Otisco_2-5	Whole Water (Lake)	290-550	1.81±0.03	1.78±0.09	NA	13.7±0.4
	Lake 256 10/12/2021_Otisco_2-6	Whole Water (Lake)	290-550	1.79±0.04	1.77±0.07	NA	13.7±0.4
	Lake 256 10/12/2021_Otisco_2-7	Whole Water (Lake)	290-550	1.77±0.04	1.75±0.08	NA	13.6±1.0
	Lake 256 10/12/2021_Otisco_2-8	Whole Water (Lake)	290-550	1.76±0.01	1.73±0.07	NA	13.6±0.5
	Lake 256 10/12/2021_Otisco_2-9	Whole Water (Lake)	290-550	1.75±0.01	1.72±0.06	1.28±0.11	13.5±1.1

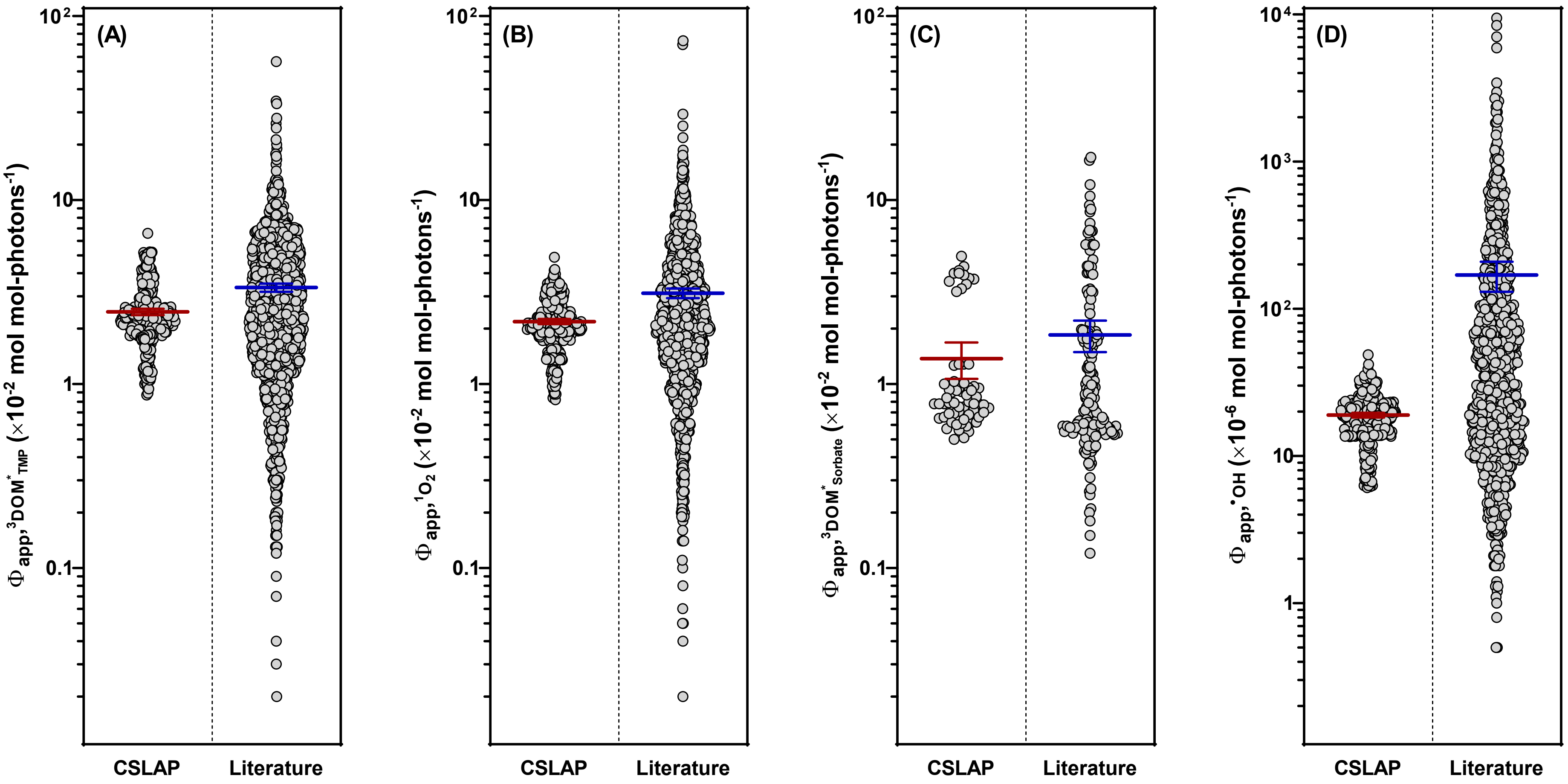

Figure S30. Comparison of $\Phi_{app,RI}$ for CSLAP samples (CSLAP; *n*=369 including lysate and supernatant samples) with those reported in the literature (i.e., $\Phi_{app,RI}$ summarized in Table S37 plus $\Phi_{app,RI}$ summarized in Wasswa *et al.*[7] Table S16 and Wasswa *et al.*[136] Table S18): **(A)** Scatter dot plot of $\Phi_{app,\,^3DOM^*_{TMP}}$ for CSLAP samples (red centerline at mean with 95% confidence interval) and $\Phi_{app,\,^3DOM^*_{TMP}}$ reported in the literature (blue centerline at mean with 95% confidence interval; *n*=1480). **(B)** Scatter dot plot of $\Phi_{app,\,^1O_2}$ for CSLAP samples and $\Phi_{app,\,^1O_2}$ reported in the literature (*n*=1492). **(C)** Scatter dot plot of $\Phi_{app,\,^3DOM^*_{HDO}}$ for CSLAP samples and $\Phi_{app,\,^3DOM^*_{Sorbate}}$ (i.e., the Φ_{app} of high-energy $^3DOM^*$ measured by sorbate probes such as sorbic acid, sorbic alcohol, and sorbic amine) reported in the literature (*n*=197). **(D)** Scatter dot plot of $\Phi_{app,\,\cdot OH}$ for CSLAP samples and $\Phi_{app,\,\cdot OH}$ reported in the literature (*n*=911). Note that Table S37 complements Table S16 in Wasswa *et al.*[7] and Table S18 in Wasswa *et al.*[136] $\Phi_{app,RI}$ values not directly reported in the references or associated supplementary documents were digitized from raw figures using *Plot Digitizer 2.6.8*. f_{TMP} were converted to $\Phi_{app,\,^3DOM^*_{TMP}}$ using $k_{TMP,\,^3DOM^*_{TMP}}$ values reported in Erickson *et al.*[86] when applicable.

References

(1) Armarego, W. L. F. *Purification of Laboratory Chemicals*; Butterworth-Heinemann, 2017.
(2) Dulin, D.; Mill, T. Development and evaluation of sunlight actinometers. *Environmental Science & Technology* **1982**, *16* (11), 815-820.
(3) Laszakovits, J. R.; Berg, S. M.; Anderson, B. G.; O'Brien, J. E.; Wammer, K. H.; Sharpless, C. M. *p*-Nitroanisole/pyridine and *p*-nitroacetophenone/pyridine actinometers revisited: Quantum yield in comparison to ferrioxalate. *Environmental Science & Technology Letters* **2017**, *4* (1), 11-14.
(4) Wang, S.; Matt, M.; Murphy, B. L.; Perkins, M.; Matthews, D. A.; Moran, S. D.; Zeng, T. Organic micropollutants in New York lakes: A statewide citizen science occurrence study. *Environmental Science & Technology* **2020**, *54* (21), 13759-13770.
(5) Prestigiacomo, A. R.; June, S. G.; Gorney, R. M.; Smith, A. J.; Clinkhammer, A. C. An evaluation of a spectral fluorometer for monitoring chlorophyll *a* in New York State lakes. *Lake and Reservoir Management* **2022**, *38* (4), 318-333.
(6) Gorney, R. M.; June, S. G.; Stainbrook, K. M.; Smith, A. J. Detections of cyanobacteria harmful algal blooms (cyanoHABs) in New York State, United States (2012–2020). *Lake and Reservoir Management* **2023**, *39* (1), 21-36.
(7) Wasswa, J.; Driscoll, C. T.; Zeng, T. Photochemical characterization of surface waters from lakes in the Adirondack Region of New York. *Environmental Science & Technology* **2020**, *54* (17), 10654-10667.
(8) Cory, R. M.; Miller, M. P.; McKnight, D. M.; Guerard, J. J.; Miller, P. L. Effect of instrument-specific response on the analysis of fulvic acid fluorescence spectra. *Limnology and Oceanography: Methods* **2010**, *8* (2), 67-78.
(9) Ohno, T. Fluorescence inner-filtering correction for determining the humification index of dissolved organic matter. *Environmental Science & Technology* **2002**, *36* (4), 742-746.
(10) Kothawala, D. N.; Murphy, K. R.; Stedmon, C. A.; Weyhenmeyer, G. A.; Tranvik, L. J. Inner filter correction of dissolved organic matter fluorescence. *Limnology and Oceanography: Methods* **2013**, *11* (12), 616-630.
(11) Lawaetz, A. J.; Stedmon, C. A. Fluorescence intensity calibration using the Raman scatter peak of water. *Applied Spectroscopy* **2009**, *63* (8), 936-940.
(12) Murphy, K. R.; Stedmon, C. A.; Graeber, D.; Bro, R. Fluorescence spectroscopy and multi-way techniques. PARAFAC. *Analytical Methods* **2013**, *5* (23), 6557-6566.
(13) Cuthbert, I. D.; del Giorgio, P. Toward a standard method of measuring color in freshwater. *Limnology and Oceanography* **1992**, *37* (6), 1319-1326.
(14) Weishaar, J. L.; Aiken, G. R.; Bergamaschi, B. A.; Fram, M. S.; Fujii, R.; Mopper, K. Evaluation of specific ultraviolet absorbance as an indicator of the chemical composition and reactivity of dissolved organic carbon. *Environmental Science & Technology* **2003**, *37* (20), 4702-4708.
(15) De Haan, H.; De Boer, T. Applicability of light absorbance and fluorescence as measures of concentration and molecular size of dissolved organic carbon in humic Lake Tjeukemeer. *Water Research* **1987**, *21* (6), 731-734.
(16) Moran, M. A.; Sheldon, W. M., Jr.; Zepp, R. G. Carbon loss and optical property changes during long-term photochemical and biological degradation of estuarine dissolved organic matter. *Limnology and Oceanography* **2000**, *45* (6), 1254-1264.
(17) Twardowski, M. S.; Boss, E.; Sullivan, J. M.; Donaghay, P. L. Modeling the spectral shape of absorption by chromophoric dissolved organic matter. *Marine Chemistry* **2004**, *89* (1), 69-88.
(18) Helms, J. R.; Stubbins, A.; Ritchie, J. D.; Minor, E. C.; Kieber, D. J.; Mopper, K. Absorption spectral slopes and slope ratios as indicators of molecular weight, source, and photobleaching of chromophoric dissolved organic matter. *Limnology and Oceanography* **2008**, *53* (3), 955-969.
(19) McKnight, D. M.; Boyer, E. W.; Westerhoff, P. K.; Doran, P. T.; Kulbe, T.; Andersen, D. T. Spectrofluorometric characterization of dissolved organic matter for indication of precursor organic material and aromaticity. *Limnology and Oceanography* **2001**, *46* (1), 38-48.

(20) Zsolnay, A.; Baigar, E.; Jimenez, M.; Steinweg, B.; Saccomandi, F. Differentiating with fluorescence spectroscopy the sources of dissolved organic matter in soils subjected to drying. *Chemosphere* **1999**, *38* (1), 45-50.
(21) Wilson, H. F.; Xenopoulos, M. A. Effects of agricultural land use on the composition of fluvial dissolved organic matter. *Nature Geoscience* **2008**, *2*, 37-41.
(22) Coble, P. G.; Del Castillo, C. E.; Avril, B. Distribution and optical properties of CDOM in the Arabian Sea during the 1995 Southwest Monsoon. *Deep Sea Research Part II: Topical Studies in Oceanography* **1998**, *45* (10), 2195-2223.
(23) Re, R.; Pellegrini, N.; Proteggente, A.; Pannala, A.; Yang, M.; Rice-Evans, C. Antioxidant activity applying an improved ABTS radical cation decolorization assay. *Free Radical Biology and Medicine* **1999**, *26* (9), 1231-1237.
(24) Rimmer, D. L.; Abbott, G. D. Phenolic compounds in NaOH extracts of UK soils and their contribution to antioxidant capacity. *European Journal of Soil Science* **2011**, *62* (2), 285-294.
(25) Walpen, N.; Schroth, M. H.; Sander, M. Quantification of phenolic antioxidant moieties in dissolved organic matter by flow-injection analysis with electrochemical detection. *Environmental Science & Technology* **2016**, *50* (12), 6423-6432.
(26) Haag, W. R.; Hoigné, J.; Gassman, E.; Braun, A. M. Singlet oxygen in surface waters - Part I: Furfuryl alcohol as a trapping agent. *Chemosphere* **1984**, *13* (5-6), 631-640.
(27) Appiani, E.; Ossola, R.; Latch, D. E.; Erickson, P. R.; McNeill, K. Aqueous singlet oxygen reaction kinetics of furfuryl alcohol: Effect of temperature, pH, and salt content. *Environmental Science: Processes & Impacts* **2017**, *19* (4), 507-516.
(28) Canonica, S.; Freiburghaus, M. Electron-rich phenols for probing the photochemical reactivity of freshwaters. *Environmental Science & Technology* **2001**, *35* (4), 690-695.
(29) McCabe, A. J.; Arnold, W. A. Multiple linear regression models to predict the formation efficiency of triplet excited states of dissolved organic matter in temperate wetlands. *Limnology and Oceanography* **2018**, *63* (5), 1992-2014.
(30) Zhou, H.; Yan, S.; Ma, J.; Lian, L.; Song, W. Development of novel chemical probes for examining triplet natural organic matter under solar illumination. *Environmental Science & Technology* **2017**, *51* (19), 11066-11074.
(31) Leifer, A. *The Kinetics of Environmental Aquatic Photochemistry: Theory and Practice*; American Chemical Society, 1988.
(32) Schwarzenbach, R. P.; Gschwend, P. M.; Imboden, D. M. *Environmental Organic Chemistry*; John Wiley & Sons, Inc., 2016.
(33) Partanen, S. B.; Erickson, P. R.; Latch, D. E.; Moor, K. J.; McNeill, K. Dissolved organic matter singlet oxygen quantum yields: Evaluation using time-resolved singlet oxygen phosphorescence. *Environmental Science & Technology* **2020**, *54* (6), 3316-3324.
(34) Wu, B.; Liu, T.; Wang, Y.; Zhao, G.; Chen, B.; Chu, C. High sample throughput LED reactor for facile characterization of the quantum yield spectrum of photochemically produced reactive intermediates. *Environmental Science & Technology* **2021**, DOI: 10.1021/acs.est.1021c04608.
(35) O'Connor, M.; Helal, S. R.; Latch, D. E.; Arnold, W. A. Quantifying photo-production of triplet excited states and singlet oxygen from effluent organic matter. *Water Research* **2019**, *156*, 23-33.
(36) Sharpless, C. M.; Aeschbacher, M.; Page, S. E.; Wenk, J.; Sander, M.; McNeill, K. Photooxidation-induced changes in optical, electrochemical, and photochemical properties of humic substances. *Environmental Science & Technology* **2014**, *48* (5), 2688-2696.
(37) McCabe, A. J.; Arnold, W. A. Seasonal and spatial variabilities in the water chemistry of prairie pothole wetlands influence the photoproduction of reactive intermediates. *Chemosphere* **2016**, *155*, 640-647.
(38) Pochon, A.; Vaughan, P. P.; Gan, D.; Vath, P.; Blough, N. V.; Falvey, D. E. Photochemical oxidation of water by 2-methyl-1,4-benzoquinone: Evidence against the formation of free hydroxyl radical. *The Journal of Physical Chemistry A* **2002**, *106* (12), 2889-2894.

(39) Gan, D.; Jia, M.; Vaughan, P. P.; Falvey, D. E.; Blough, N. V. Aqueous photochemistry of methyl-benzoquinone. *The Journal of Physical Chemistry A* **2008**, *112* (13), 2803-2812.
(40) Page, S. E.; Arnold, W. A.; McNeill, K. Assessing the contribution of free hydroxyl radical in organic matter-sensitized photohydroxylation reactions. *Environmental Science & Technology* **2011**, *45* (7), 2818-2825.
(41) Liu, Y.; Yan, S.; Lian, L.; Ma, J.; Zhou, H.; Song, W. Assessing the contribution of hydroxylation species in the photochemical transformation of primidone (pharmaceutical). *Science of the Total Environment* **2019**, *696*, 133826.
(42) Page, S. E.; Arnold, W. A.; McNeill, K. Terephthalate as a probe for photochemically generated hydroxyl radical. *Journal of Environmental Monitoring* **2010**, *12*, 1658-1665.
(43) Yuan, C.; Chin, Y.-P.; Weavers, L. K. Photochemical acetochlor degradation induced by hydroxyl radical in Fe-amended wetland waters: Impact of pH and dissolved organic matter. *Water Research* **2018**, *132*, 52-60.
(44) Brezonik, P. L.; Fulkerson-Brekken, J. Nitrate-induced photolysis in natural waters: Controls on concentrations of hydroxyl radical photo-intermediates by natural scavenging agents. *Environmental Science & Technology* **1998**, *32* (19), 3004-3010.
(45) Vaughan, P. P.; Blough, N. V. Photochemical formation of hydroxyl radical by constituents of natural waters. *Environmental Science & Technology* **1998**, *32* (19), 2947-2953.
(46) Vione, D.; Falletti, G.; Maurino, V.; Minero, C.; Pelizzetti, E.; Malandrino, M.; Ajassa, R.; Olariu, R.-I.; Arsene, C. Sources and sinks of hydroxyl radicals upon irradiation of natural water samples. *Environmental Science & Technology* **2006**, *40* (12), 3775-3781.
(47) Zepp, R. G.; Faust, B. C.; Hoigné, J. Hydroxyl radical formation in aqueous reactions (pH 3-8) of iron(II) with hydrogen peroxide: The photo-Fenton reaction. *Environmental Science & Technology* **1992**, *26* (2), 313-319.
(48) Southworth, B. A.; Voelker, B. M. Hydroxyl radical production via the photo-Fenton reaction in the presence of fulvic acid. *Environmental Science & Technology* **2003**, *37* (6), 1130-1136.
(49) Vermilyea, A. W.; Voelker, B. M. Photo-Fenton reaction at near neutral pH. *Environmental Science & Technology* **2009**, *43* (18), 6927-6933.
(50) Charbouillot, T.; Brigante, M.; Mailhot, G.; Maddigapu, P. R.; Minero, C.; Vione, D. Performance and selectivity of the terephthalic acid probe for $^{\bullet}OH$ as a function of temperature, pH and composition of atmospherically relevant aqueous media. *Journal of Photochemistry and Photobiology A: Chemistry* **2011**, *222* (1), 70-76.
(51) Miller, P. L.; Chin, Y.-P. Photoinduced degradation of carbaryl in a wetland surface water. *Journal of Agricultural and Food Chemistry* **2002**, *50* (23), 6758-6765.
(52) Yuan, C.; Sleighter, R. L.; Weavers, L. K.; Hatcher, P. G.; Chin, Y.-P. Fast photomineralization of dissolved organic matter in acid mine drainage impacted waters. *Environmental Science & Technology* **2019**, *53* (11), 6273-6281.
(53) Matthews, R. W. The radiation-chemistry of the terephthalate dosimeter. *Radiation Research* **1980**, *83* (1), 27-41.
(54) Mark, G.; Tauber, A.; Laupert, R.; Schuchmann, H.-P.; Schulz, D.; Mues, A.; von Sonntag, C. OH-radical formation by ultrasound in aqueous solution – Part II: Terephthalate and Fricke dosimetry and the influence of various conditions on the sonolytic yield. *Ultrasonics Sonochemistry* **1998**, *5* (2), 41-52.
(55) Gonzalez, D. H.; Kuang, X. M.; Scott, J. A.; Rocha, G. O.; Paulson, S. E. Terephthalate probe for hydroxyl radicals: Yield of 2-hydroxyterephthalic acid and transition metal interference. *Analytical Letters* **2018**, *51* (15), 2488-2497.
(56) Buxton, G. V.; Greenstock, C. L.; Helman, W. P.; Ross, A. B. Critical review of rate constants for reactions of hydrated electrons and hydroxyl radicals ($^{\bullet}OH/^{\bullet}O^{-}$) in aqueous solution. *Journal of Physical and Chemical Reference Data* **1988**, *17* (2), 513-886.
(57) Larson, R. A.; Zepp, R. G. Reactivity of the carbonate radical with aniline derivatives. *Environmental Toxicology and Chemistry* **1988**, *7* (4), 265-274.
(58) Westerhoff, P.; Song, R.; Amy, G.; Minear, R. NOM's role in bromine and bromate formation during ozonation. *Journal - American Water Works Association* **1998**, *90* (2), 82-94.

(59) Westerhoff, P.; Aiken, G.; Amy, G.; Debroux, J. Relationships between the structure of natural organic matter and its reactivity towards molecular ozone and hydroxyl radicals. *Water Research* **1999**, *33* (10), 2265-2276.
(60) Goldstone, J. V.; Pullin, M. J.; Bertilsson, S.; Voelker, B. M. Reactions of hydroxyl radical with humic substances: Bleaching, mineralization, and production of bioavailable carbon substrates. *Environmental Science & Technology* **2002**, *36* (3), 364-372.
(61) Westerhoff, P.; Mezyk, S. P.; Cooper, W. J.; Minakata, D. Electron pulse radiolysis determination of hydroxyl radical rate constants with Suwannee River fulvic acid and other dissolved organic matter isolates. *Environmental Science & Technology* **2007**, *41* (13), 4640-4646.
(62) Nagarnaik, P. M.; Boulanger, B. Advanced oxidation of alkylphenol ethoxylates in aqueous systems. *Chemosphere* **2011**, *85* (5), 854-860.
(63) Katsoyiannis, I. A.; Canonica, S.; von Gunten, U. Efficiency and energy requirements for the transformation of organic micropollutants by ozone, O_3/H_2O_2 and UV/H_2O_2. *Water Research* **2011**, *45* (13), 3811-3822.
(64) McKay, G.; Kleinman, J.; Johnston, K.; Dong, M.; Rosario-Ortiz, F.; Mezyk, S. Kinetics of the reaction between the hydroxyl radical and organic matter standards from the International Humic Substance Society. *Journal of Soils and Sediments* **2014**, *14* (2), 298-304.
(65) Appiani, E.; Page, S. E.; McNeill, K. On the use of hydroxyl radical kinetics to assess the number-average molecular weight of dissolved organic matter. *Environmental Science & Technology* **2014**, *48* (20), 11794-11802.
(66) Page, S. E.; Logan, J. R.; Cory, R. M.; McNeill, K. Evidence for dissolved organic matter as the primary source and sink of photochemically produced hydroxyl radical in arctic surface waters. *Environmental Science: Processes & Impacts* **2014**, *16* (4), 807-822.
(67) Donham, J. E.; Rosenfeldt, E. J.; Wigginton, K. R. Photometric hydroxyl radical scavenging analysis of standard natural organic matter isolates. *Environmental Science: Processes & Impacts* **2014**, *16* (4), 764-769.
(68) McKay, G.; Rosario-Ortiz, F. L. Temperature dependence of the photochemical formation of hydroxyl radical from dissolved organic matter. *Environmental Science & Technology* **2015**, *49* (7), 4147-4154.
(69) Lee, D.; Kwon, M.; Ahn, Y.; Jung, Y.; Nam, S.-N.; Choi, I.-H.; Kang, J.-W. Characteristics of intracellular algogenic organic matter and its reactivity with hydroxyl radicals. *Water Research* **2018**, *144*, 13-25.
(70) Ossola, R.; Jönsson, O. M.; Moor, K.; McNeill, K. Singlet oxygen quantum yields in environmental waters. *Chemical Reviews* **2021**, *121* (7), 4100-4146.
(71) Mostafa, S.; Rosario-Ortiz, F. L. Singlet oxygen formation from wastewater organic matter. *Environmental Science & Technology* **2013**, *47* (15), 8179-8186.
(72) Manfrin, A.; Nizkorodov, S. A.; Malecha, K. T.; Getzinger, G. J.; McNeill, K.; Borduas-Dedekind, N. Reactive oxygen species production from secondary organic aerosols: The importance of singlet oxygen. *Environmental Science & Technology* **2019**, *53* (15), 8553-8562.
(73) Dalrymple, R. M.; Carfagno, A. K.; Sharpless, C. M. Correlations between dissolved organic matter optical properties and quantum yields of singlet oxygen and hydrogen peroxide. *Environmental Science & Technology* **2010**, *44* (15), 5824-5829.
(74) Zepp, R. G.; Schlotzhauer, P. F.; Sink, R. M. Photosensitized transformations involving electronic energy transfer in natural waters: Role of humic substances. *Environmental Science & Technology* **1985**, *19* (1), 74-81.
(75) Zhou, H.; Yan, S.; Lian, L.; Song, W. Triplet-state photochemistry of dissolved organic matter: Triplet-state energy distribution and surface electric charge conditions. *Environmental Science & Technology* **2019**, *53* (5), 2482-2490.
(76) Aguer, J.-P.; Mailhot, G.; Bolte, M. Unexpected 2,4,6-trimethylphenol oxidation in the presence of Fe(III) aquacomplexes. *New Journal of Chemistry* **2006**, *30* (2), 191-196.
(77) Faust, B. C.; Hoigné, J. Sensitized photooxidation of phenols by fulvic acid and in natural waters. *Environmental Science & Technology* **1987**, *21* (10), 957-964.

(78) Canonica, S.; Jans, U.; Stemmler, K.; Hoigné, J. Transformation kinetics of phenols in water: Photosensitization by dissolved natural organic material and aromatic ketones. *Environmental Science & Technology* **1995**, *29* (7), 1822-1831.
(79) Chin, Y.-P.; Miller, P. L.; Zeng, L.; Cawley, K.; Weavers, L. K. Photosensitized degradation of bisphenol A by dissolved organic matter. *Environmental Science & Technology* **2004**, *38* (22), 5888-5894.
(80) Aguer, J.-P.; Tetegan, D.; Richard, C. Humic substances mediated phototransformation of 2,4,6-trimethylphenol: A catalytic reaction. *Photochemical & Photobiological Sciences* **2005**, *4* (6), 451-453.
(81) Halladja, S.; ter Halle, A.; Aguer, J.-P.; Boulkamh, A.; Richard, C. Inhibition of humic substances mediated photooxygenation of furfuryl alcohol by 2,4,6-trimethylphenol. Evidence for reactivity of the phenol with humic triplet excited states. *Environmental Science & Technology* **2007**, *41* (17), 6066-6073.
(82) Bodhipaksha, L. C.; Sharpless, C. M.; Chin, Y.-P.; Sander, M.; Langston, W. K.; MacKay, A. A. Triplet photochemistry of effluent and natural organic matter in whole water and isolates from effluent-receiving rivers. *Environmental Science & Technology* **2015**, *49* (6), 3453-3463.
(83) McCabe, A. J.; Arnold, W. A. Reactivity of triplet excited states of dissolved natural organic matter in stormflow from mixed-use watersheds. *Environmental Science & Technology* **2017**, *51* (17), 9718-9728.
(84) Tenorio, R.; Fedders, A. C.; Strathmann, T. J.; Guest, J. S. Impact of growth phases on photochemically produced reactive species in the extracellular matrix of algal cultivation systems. *Environmental Science: Water Research & Technology* **2017**, *3* (6), 1095-1108.
(85) Stirchak, L. T.; Moor, K. J.; McNeill, K.; Donaldson, D. J. Differences in photochemistry between seawater and freshwater for two natural organic matter samples. *Environmental Science: Processes & Impacts* **2019**, *21* (1), 28-39.
(86) Erickson, P. R.; Moor, K. J.; Werner, J. J.; Latch, D. E.; Arnold, W. A.; McNeill, K. Singlet oxygen phosphorescence as a probe for triplet-state dissolved organic matter reactivity. *Environmental Science & Technology* **2018**, *52* (16), 9170-9178.
(87) Kroflič, A.; Schaefer, T.; Huš, M.; Phuoc Le, H.; Otto, T.; Herrmann, H. OH radicals reactivity towards phenol-related pollutants in water: Temperature dependence of the rate constants and novel insights into the [OH–phenol]$^{\cdot}$ adduct formation. *Physical Chemistry Chemical Physics* **2020**, *22* (3), 1324-1332.
(88) Canonica, S.; Hellrung, B.; Wirz, J. Oxidation of phenols by triplet aromatic ketones in aqueous solution. *The Journal of Physical Chemistry A* **2000**, *104* (6), 1226-1232.
(89) Canonica, S.; Laubscher, H. U. Inhibitory effect of dissolved organic matter on triplet-induced oxidation of aquatic contaminants. *Photochemical and Photobiological Sciences* **2008**, *7* (5), 547-551.
(90) Wenk, J.; von Gunten, U.; Canonica, S. Effect of dissolved organic matter on the transformation of contaminants induced by excited triplet states and the hydroxyl radical. *Environmental Science & Technology* **2011**, *45* (4), 1334-1340.
(91) Wenk, J.; Canonica, S. Phenolic antioxidants inhibit the triplet-induced transformation of anilines and sulfonamide antibiotics in aqueous solution. *Environmental Science & Technology* **2012**, *46* (10), 5455-5462.
(92) Wenk, J.; Eustis, S. N.; McNeill, K.; Canonica, S. Quenching of excited triplet states by dissolved natural organic matter. *Environmental Science & Technology* **2013**, *47* (22), 12802-12810.
(93) Maizel, A. C.; Remucal, C. K. The effect of probe choice and solution conditions on the apparent photoreactivity of dissolved organic matter. *Environmental Science: Processes & Impacts* **2017**, *19* (8), 1040-1050.
(94) Baird, R. B.; Eaton, A. D.; Rice, E. W. *Standard Methods For the Examination of Water and Wastewater*; American Public Health Association (APHA), American Water Works Association (AWWA), Water Environment Federation (WEF), 2017.
(95) Al Housari, F.; Vione, D.; Chiron, S.; Barbati, S. Reactive photoinduced species in estuarine waters. Characterization of hydroxyl radical, singlet oxygen and dissolved organic matter triplet state in natural oxidation processes. *Photochemical and Photobiological Sciences* **2010**, *9* (1), 78-86.
(96) Golanoski, K. S.; Fang, S.; Del Vecchio, R.; Blough, N. V. Investigating the mechanism of phenol photooxidation by humic substances. *Environmental Science & Technology* **2012**, *46* (7), 3912-3920.

(97) Wenk, J.; Nguyen, M. T.; Nelson, K. L. Natural photosensitizers in constructed unit process wetlands: Photochemical characterization and inactivation of pathogen indicator organisms. *Environmental Science & Technology* **2019**, *53* (13), 7724-7735.
(98) McKay, G.; Couch, K. D.; Mezyk, S. P.; Rosario-Ortiz, F. L. Investigation of the coupled effects of molecular weight and charge-transfer interactions on the optical and photochemical properties of dissolved organic matter. *Environmental Science & Technology* **2016**, *50* (15), 8093-8102.
(99) McKay, G.; Huang, W.; Romera-Castillo, C.; Crouch, J. E.; Rosario-Ortiz, F. L.; Jaffé, R. Predicting reactive intermediate quantum yields from dissolved organic matter photolysis using optical properties and antioxidant capacity. *Environmental Science & Technology* **2017**, *51* (10), 5404-5413.
(100) Maizel, A. C.; Remucal, C. K. Molecular composition and photochemical reactivity of size-fractionated dissolved organic matter. *Environmental Science & Technology* **2017**, *51* (4), 2113-2123.
(101) Maizel, A. C.; Li, J.; Remucal, C. K. Relationships between dissolved organic matter composition and photochemistry in lakes of diverse trophic status. *Environmental Science & Technology* **2017**, *51* (17), 9624-9632.
(102) Berg, S. M.; Whiting, Q. T.; Herrli, J. A.; Winkels, R.; Wammer, K. H.; Remucal, C. K. The role of dissolved organic matter composition in determining photochemical reactivity at the molecular level. *Environmental Science & Technology* **2019**, *53* (20), 11725-11734.
(103) McNeill, K.; Canonica, S. Triplet state dissolved organic matter in aquatic photochemistry: Reaction mechanisms, substrate scope, and photophysical properties. *Environmental Science: Processes & Impacts* **2016**, *18* (11), 1381-1399.
(104) Grebel, J. E.; Pignatello, J. J.; Mitch, W. A. Sorbic acid as a quantitative probe for the formation, scavenging and steady-state concentrations of the triplet-excited state of organic compounds. *Water Research* **2011**, *45* (19), 6535-6544.
(105) Chen, Y.; Hu, C.; Hu, X.; Qu, J. Indirect photodegradation of amine drugs in aqueous solution under simulated sunlight. *Environmental Science & Technology* **2009**, *43* (8), 2760-2765.
(106) Chen, Y.; Liang, J.; Liu, L.; Lu, X.; Deng, J.; Pozdnyakov, I. P.; Zuo, Y. Photosensitized degradation of amitriptyline and its active metabolite nortriptyline in aqueous fulvic acid solution. *Journal of Environmental Quality* **2017**, *46* (5), 1081-1087.
(107) Pozdnyakov, I. P.; Tyutereva, Y. E.; Parkhats, M. V.; Grivin, V. P.; Fang, Y.; Liu, L.; Wan, D.; Luo, F.; Chen, Y. Mechanistic investigation of humic substances assisted photodegradation of imipramine under simulated sunlight. *Science of the Total Environment* **2020**, *738*, 140298.
(108) Saunders, D. G.; Mosier, J. W. Photolysis of the aquatic herbicide fluridone in aqueous solution. *Journal of Agricultural and Food Chemistry* **1983**, *31* (2), 237-241.
(109) Niu, X.-Z.; Croué, J.-P. Photochemical production of hydroxyl radical from algal organic matter. *Water Research* **2019**, *161*, 11-16.
(110) Zeng, Y.; Fang, G.; Fu, Q.; Peng, F.; Wang, X.; Dionysiou, D. D.; Guo, J.; Gao, J.; Zhou, D.; Wang, Y. Mechanistic study of the effects of agricultural amendments on photochemical processes in paddy water during rice growth. *Environmental Science & Technology* **2022**, *56* (7), 4221-4230.
(111) Xu, H.; Li, X.; Guo, M.; Li, F.; Yang, K.; Liu, X. Dissolved organic matters with low molecular weight fractions exhibit high photochemical potential for reactive oxygen formation. *Chemosphere* **2022**, *305*, 135542.
(112) Zhang, T.; Ma, H.; Hong, Z.; Fu, G.; Zheng, Y.; Li, Z.; Cui, F. Photo-reactivity and photo-transformation of algal dissolved organic matter unraveled by optical spectroscopy and high-resolution mass spectrometry analysis. *Environmental Science & Technology* **2022**, *56* (18), 13439-13448.
(113) Wan, D.; Wang, J.; Chen, T.; Xiang, W.; Selvinsimpson, S.; Chen, Y. Effect of disinfection on the photoreactivity of effluent organic matter and photodegradation of organic contaminants. *Water Research* **2022**, *219*, 118552.
(114) Wan, D.; Yang, J.; Wang, X.; Xiang, W.; Selvinsimpson, S.; Chen, Y. Wavelength-dependent photoreactivity of root exudates from aquatic plants under UV-LED irradiation. *ACS ES&T Water* **2022**, *2* (12), 2613-2622.

(115) Song, N.; Wu, D.; Xu, H.; Jiang, H. Integrated evaluation of the reactive oxygen species (ROS) production characteristics in one large lake under alternating flood and drought conditions. *Water Research* **2022**, *225*, 119136.
(116) Hu, A.; Li, L.; Huang, Y.; Fu, Q.-L.; Wang, D.; Zhang, W. Photochemical transformation mechanisms of dissolved organic matters (DOM) derived from different bio-stabilization sludge. *Environment International* **2022**, *169*, 107534.
(117) Liu, Y.; Wang, M.; Yin, S.; Xie, L.; Qu, X.; Fu, H.; Shi, Q.; Zhou, F.; Xu, F.; Tao, S.; Zhu, D. Comparing photoactivities of dissolved organic matter released from rice straw-pyrolyzed biochar and composted rice straw. *Environmental Science & Technology* **2022**, *56* (4), 2803-2815.
(118) Chen, X.; Deng, L.; Chen, Y.; Wan, D. Impact of ozonation on the optical properties and photo-reactivity of dissolved organic matter. *Journal of Environmental Chemical Engineering* **2023**, *11* (1), 109251.
(119) Guo, Z.; Wang, T.; Chen, G.; Wang, J.; Fujii, M.; Yoshimura, C. Apparent quantum yield for photo-production of singlet oxygen in reservoirs and its relation to the water matrix. *Water Research* **2023**, *244*, 120456.
(120) Berg, S. M.; Wammer, K. H.; Remucal, C. K. Dissolved organic matter photoreactivity is determined by its optical properties, redox activity, and molecular composition. *Environmental Science & Technology* **2023**, *57* (16), 6703-6711.
(121) Kong, S.; Liu, X.; Jiang, H.; Hong, W.; Zhang, J.; Song, W.; Yan, S. Photobleaching-induced changes in the optical and photochemical properties of algal organic matter. *Water Research* **2023**, *243*, 120395.
(122) Allen, A.; Cheng, K.; McKay, G. Evaluating the pH-dependence of DOM absorbance, fluorescence, and photochemical production of singlet oxygen. *Environmental Science: Processes & Impacts* **2023**, *25* (12), 1974-1985.
(123) Buckley, S.; Leresche, F.; Hanson, B.; Rosario-Ortiz, F. L. Decoupling optical response and photochemical formation of singlet oxygen in size isolated fractions of ozonated dissolved organic matter. *Environmental Science & Technology* **2023**, *57* (14), 5603-5610.
(124) Wang, Y.; Wu, B.; Zheng, X.; Chen, B.; Chu, C. Assessing the quantum yield spectrum of photochemically produced reactive intermediates from black carbon of various sources and properties. *Water Research* **2023**, *229*, 119450.
(125) Zhou, Z.; Yang, L.; Qu, X.; Fu, H. Dissolved black carbon mediated photo-oxidation of arsenic(III) to arsenic(V) in water: The key role of triplet states. *Chemosphere* **2024**, *347*, 140718.
(126) Li, L.; Cheng, W.; Xie, X.; Zhao, R.; Wang, Y.; Wang, Z. Photo-Reactivity of dissolved black carbon unveiled by combination of optical spectroscopy and FT-ICR MS analysis: Effects of pyrolysis temperature. *Water Research* **2024**, *251*, 121138.
(127) Li, Y.; Zhang, K.; Apell, J.; Ruan, Y.; Huang, X.; Nah, T. Photoproduction of reactive intermediates from dissolved organic matter in coastal seawater around an urban metropolis in South China: Characterization and predictive modeling. *Science of the Total Environment* **2024**, *921*, 170998.
(128) Wan, D.; Song, G.; Mi, W.; Tu, X.; Zhao, Y.; Bi, Y. Insights into the enhanced photogeneration of hydroxyl radicals from chlorinated dissolved organic matter. *Environmental Science & Technology* **2024**, *58* (1), 805-815.
(129) Madhiyan, M.; Moor, K. J. Singlet oxygen quantum yields of pyrogenic dissolved organic matter from lab-prepared and wildfire chars. *Environmental Science & Technology* **2024**, *58* (2), 1265-1273.
(130) He, H.; Sun, N.; Li, L.; Zhou, H.; Hu, A.; Yang, X.; Ai, J.; Jiao, R.; Yang, X.; Wang, D.; Zhang, W. Photochemical transformation of dissolved organic matter in surface water augmented the formation of disinfection byproducts. *Environmental Science & Technology* **2024**, *58* (7), 3399-3411.
(131) Guo, Z.; Wang, T.; Ichiyanagi, H.; Ateia, M.; Chen, G.; Wang, J.; Fujii, M.; En, K.; Li, T.; Sohrin, R.; Yoshimura, C. Photo-production of excited triplet-state of dissolved organic matters in inland freshwater and coastal seawater. *Water Research* **2024**, *253*, 121260.
(132) Guo, Z.-C.; Zhang, L.; Chen, Y.; Huang, C.; Liao, Z.-M. Effect of UV-LED wavelength on reactive species photogeneration from dissolved organic matter. *Water* **2024**, *16* (5), 635.

(133) Xie, H.; Li, Q.; Wang, M.; Feng, Y.; Wang, B. Unraveling the photochemical behavior of dissolved organic matter derived from hydrothermal carbonization process water: Insights from molecular transformation and photoactive species. *Journal of Hazardous Materials* **2024**, *469*, 133946.
(134) Du, P.; Tang, K.; Yang, B.; Mo, X.; Wang, J. Reassessing the quantum yield and reactivity of triplet-state dissolved organic matter via global kinetic modeling. *Environmental Science & Technology* **2024**, *58* (13), 5856-5865.
(135) Du, R.; Wen, J.; Huang, J.; Zhang, Q.; Shi, X.; Wang, B.; Deng, S.; Yu, G. Dissolved organic matter isolates obtained by solid phase extraction exhibit higher absorption and lower photo-reactivity: Effect of components. *Water Research* **2024**, 121604.
(136) Wasswa, J.; Driscoll, C. T.; Zeng, T. Contrasting impacts of photochemical and microbial processing on the photoreactivity of dissolved organic matter in an Adirondack Lake watershed. *Environmental Science & Technology* **2022**, *56* (3), 1688-1701.
